# Multi-omic analysis of deep learning-derived phenotypes links ophthalmic imaging to cardiovascular and neurological traits

**DOI:** 10.1038/s44161-026-00815-5

**Published:** 2026-06-16

**Authors:** Thomas H. Julian, Haoran Dou, Jinming Duan, Jinghan Huang, Esther Yoo, David J. Green, Andrew Strange, Elham Alhathli, Matthew Sperrin, Pearse A. Keane, Emily Y. Chew, Bernard Keavney, Tomas W. Fitzgerald, Johnathan Cooper-Knock, Ewan Birney, Alejandro F. Frangi, Panagiotis I. Sergouniotis

**Affiliations:** 1 Division of Evolution, Infection and Genomics, School of Biological Sciences, Faculty of Biology, Medicine and Health, University of Manchester, Manchester, UK; 2 Centre for Computational Imaging and Modelling in Medicine (CIMIM), The Christabel Pankhurst Institute, The University of Manchester, Manchester, UK; 3 Manchester Royal Eye Hospital, Manchester University NHS Foundation Trust, Manchester, UK; 4 European Molecular Biology Laboratory, European Bioinformatics Institute (EMBL-EBI), Wellcome Genome Campus, Cambridge, UK; 5 Department of Computer Science, School of Engineering, Faculty of Science and Engineering, University of Manchester, Manchester, UK; 6 Division of Informatics, Imaging, and Data Sciences, School of Health Sciences, Faculty of Biology, Medicine and Health, University of Manchester, Manchester, UK; 7 BHF Centre of Research Excellence, University of Manchester, Manchester, UK; 8 Sheffield Institute for Translational Neuroscience (SITraN), University of Sheffield, Sheffield, UK; 9 NIHR Sheffield Biomedical Research Centre, Sheffield, UK; 10 NIHR Biomedical Research Centre at Moorfields Eye Hospital NHS Foundation Trust, London, UK; 11 Institute of Ophthalmology, University College London, London, UK; 12 Division of Epidemiology and Clinical Applications, National Eye Institute/National Institutes of Health, Bethesda, MD USA; 13 Division of Cardiovascular Sciences, School of Medical Sciences, Faculty of Biology, Medicine, and Health, The University of Manchester, Manchester, UK; 14 NIHR Manchester Biomedical Research Centre, Manchester Academic Health Sciences Centre, University of Manchester, Manchester, UK; 15 Medical Imaging Research Center (MIRC), University Hospital Gasthuisberg. Cardiovascular Sciences and Electrical Engineering, KU Leuven, Leuven, Belgium

**Keywords:** Machine learning, Vascular diseases, Image processing, Genome-wide association studies, Neurodegenerative diseases

## Abstract

The eye is a recognized source of biomarkers for cardiovascular and neurodegenerative disease risk. Here we characterize the breadth of these associations and identify biological axes that may mediate them. Using UK Biobank data, we developed a multi-omic analysis pipeline integrating physiological, radiomic, metabolomic and genomic information. We trained retinal adversarial autoencoders to represent optical coherence tomography images and color fundus photographs as 256-dimensional embeddings. Retinal adversarial autoencoder-derived embeddings were associated with a range of cardiovascular and neurodegenerative diseases, including ischemic heart disease, cerebrovascular disease, Parkinson’s disease and dementia. Examining associations across diverse omics datasets, we provide evidence linking ophthalmic imaging features to neurological and cardiovascular anatomy and function, lipid metabolism and gene sets associated with neurodegenerative pathology. Collectively, our findings show that ophthalmic features reflect complex, multisystem biological processes and reinforce the role of the eye as a composite indicator of systemic health.

## Abstract (web summary)

Julian et al. combine machine learning and multi-omic analyses using UK Biobank data to link ophthalmic image features to cardiovascular and neurodegenerative diseases and identify relevant pathways.

## Main

The eye is uniquely positioned among human organs in that it permits direct, noninvasive visualization of the vasculature and central nervous system^1–3^. Given this property, ophthalmic structures have long been recognized as potential indicators of systemic health, particularly in relation to neurodegenerative and cardiovascular disease.

In clinical practice, this relationship is well established and materially important. Ophthalmic findings often prompt investigations into systemic conditions: retinal features may lead to assessment of blood pressure or diabetic status, papilledema to neuroimaging, and signs of ocular ischemia to evaluation of carotid artery patency^4^. These associations, however, reflect overt disease, manifestations that are already visible to the human eye and reflect established disease.

The role of the eye in public health is now increasingly recognized as being substantially broader than previously understood. Several developments have elevated interest in the field: (1) advances in ophthalmic imaging technologies have offered better resolution of disease-relevant tissues^5^; (2) retinal imaging is becoming increasingly widespread—with modalities such as color fundus photography (CFP) and optical coherence tomography (OCT) now available not only in specialist eye clinics but also in many optometry practices and community-based healthcare settings; and (3) machine learning models have become increasingly effective at representing complex structures in medical imaging data^6^. Inspired by these advances, deep learning models using ophthalmic imaging—developed by our group and others—have been applied to predict a range of neurodegenerative and cardiovascular conditions including myocardial infarction, heart failure, Alzheimer’s disease, stroke and Parkinson’s disease^2,7–11^.

Despite significant progress in this area, several key challenges remain. These include providing explainability of the relationship between the eye, heart and brain; determining the breadth of disease that can be predicted; incorporating multimodal data in models; integrating temporally embedded, sequential imaging data to enable modeling of longitudinal health trajectories; improving model validity across diverse populations; exploring the cost-effectiveness of these technologies for primary prevention; and solving logistical technology implementation challenges required for deployment. In this study, we address two of these challenges: (1) delineating the spectrum of neurodegenerative and cardiovascular diseases that are linked to ophthalmic imaging features and (2) characterizing the aberrant biological processes detectable in ophthalmic images that may be relevant to systemic disease risk. Previous work has principally focused on exploring the mechanisms linking ophthalmic features to systemic disease using a narrow range of expert-defined imaging traits, such as OCT layer thicknesses, and vessel widths and tortuosity measures in CFPs. This approach leads to repeated study of features we already know are important and may neglect more complex or abstract structural features that are challenging to define using human labels. Accordingly, we introduce the retinal adversarial autoencoder (Ret-AAE), an autoencoder framework that compresses OCT and CFP images into 256-dimensional vector embeddings (hereafter, ‘Ret-AAE embeddings’), allowing exploration of oculo-systemic associations in a manner free of previous assumptions.

**Fig. 1:**
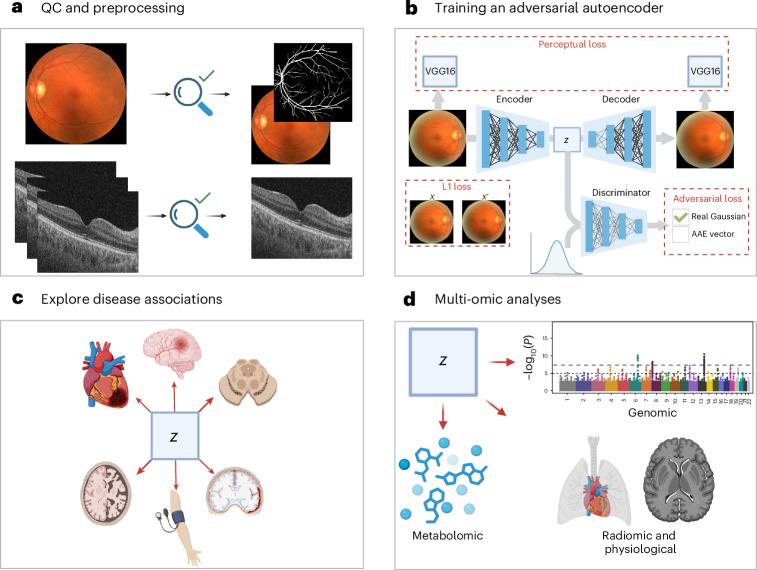
An overview of the four key components of this analysis. **a**, Data are subjected to quality control before analysis. **b**, The OCT and CFP images are then used to train an adversarial autoencoder. **c**,**d**, The latent features (‘embeddings’) projected by the encoder (denoted *z*) are then used to explore ophthalmic associations with disease (**c**) and study potential biological and genetic mechanisms that link ophthalmic features to systemic disease (**d**). QC, quality control; *z*, the latent representation (embeddings); *X*, the original image; *X’*, the reconstructed image; VGG16, a convolutional neural network developed by the Visual Geometry Group. Figure created in BioRender; Julian, T. https://biorender.com/qxpq8e6 (2026).

To address challenge (1), we investigated the breadth of cardiovascular and neurodegenerative disease associations with ophthalmic features using two complementary analyses: (a) exploration of the association between multimodality Ret-AAE embeddings and baseline neurodegenerative and cardiovascular conditions, in which variations probably reflect the presence of diagnosed disease or its associated systemic correlates and (b) prospective associations between multimodality Ret-AAE embeddings and incident disease, establishing the extent to which retinal features may serve as early indicators of future pathology. With a view to addressing challenge (2), we explored the plethora of factors that may mediate the link between retinal imaging features and neurological and cardiovascular health, generating mechanistic hypotheses for further validation. To this end, we developed a hypothesis-free multi-omic pipeline leveraging UK Biobank (UKB) data, integrating physiological, functional, radiomic, metabolomic and genomic data to interrogate the architecture of these oculo-systemic associations (Fig. 1).

## Results

### Ret-AAE maps ophthalmic images to a low-dimensional vector while preserving critical anatomical features

**Extended Data Fig. 1:**
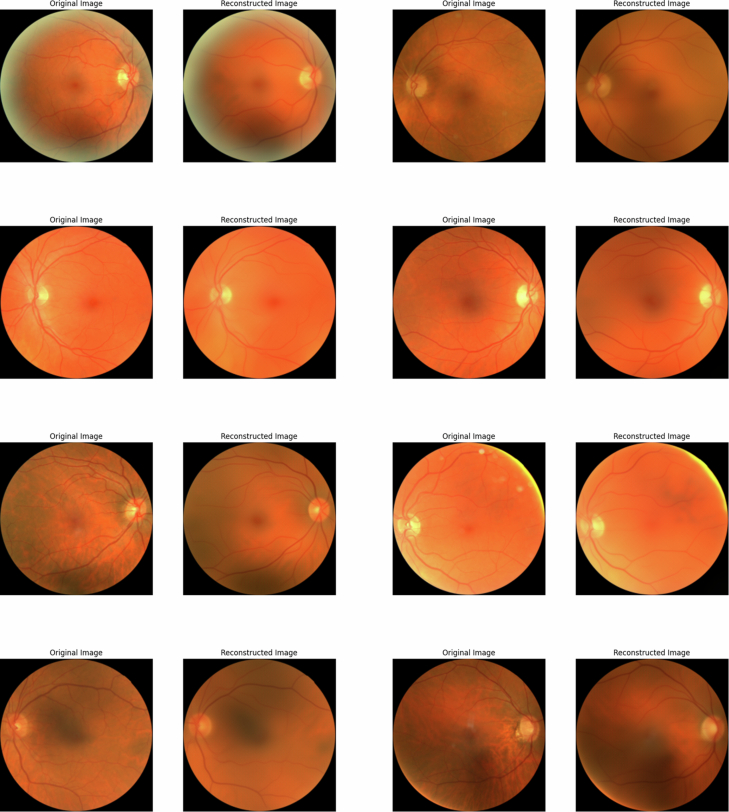
Example Ret-AAE CFP reconstructions. Eight randomly selected examples of CFP reconstructions (images unseen during training). Reproduced with the permission of UK Biobank.

**Extended Data Fig. 2:**
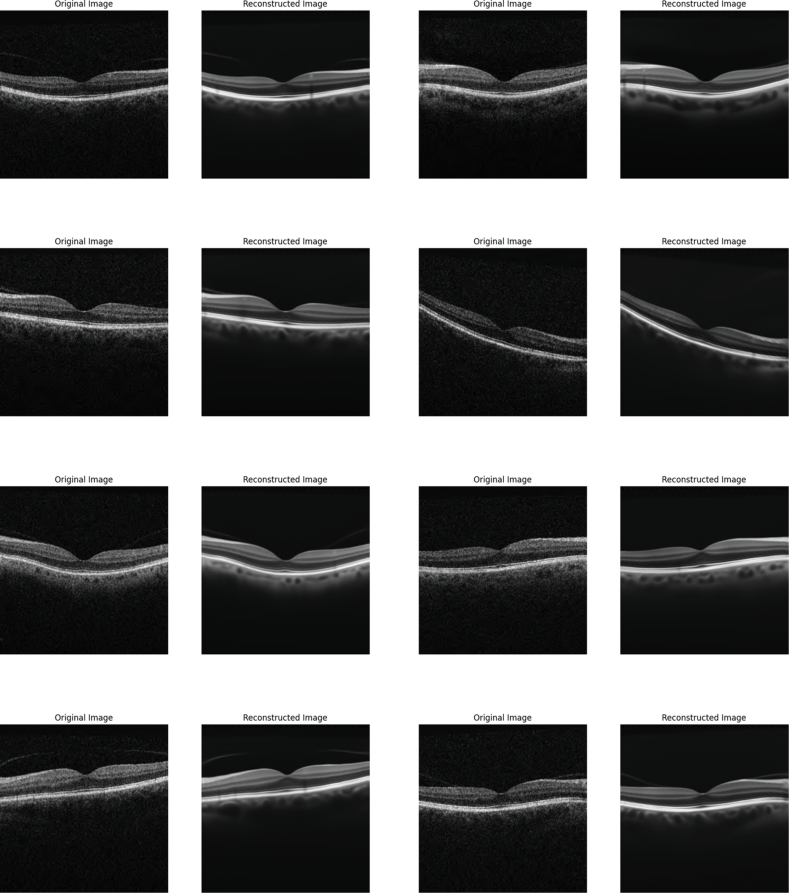
Example Ret-AAE OCT reconstructions. Eight randomly selected examples of OCT reconstructions (images unseen during training). Reproduced with the permission of UK Biobank.

After quality control, a total of 63,946 images were available to train the CFP-AAE (13,570 and 14,024 for test and validation, respectively). Furthermore, a total of 88,972 images were available to train the OCT-AAE (19,018 and 19,621 for test and validation, respectively). The structural similarity index metric (SSIM) for CFP reconstructions was 0.89, and the peak signal to noise ratio (PSNR) was 30.65 dB. The SSIM for the OCT images was 0.72 while the PSNR was 29.19 dB. The speckled (noisy) nature of OCT images was not preserved during reconstruction, and images were smoothed, which may account for the lower reconstruction metrics relative to CFPs despite perceptually superior anatomical structure preservation. Eight randomly sampled example reconstructions for OCTs and CFPs are presented in Extended Data Figs. 1 and 2. Most ophthalmic imaging embeddings (latent features) approximated a Gaussian distribution (Supplementary Figs. 1 and 2).

### Population characteristics of those with sufficiently high-quality images for phenotypic and genotypic analysis

Phenotypic and genotypic associations were conducted in study participants with sufficiently high-quality left eye CFP (*n* = 42,001) or OCT (*n* = 68,942) images, although the total number of study participants for each analysis varied from test to test owing to missingness. The overall cohort with such images were significantly younger than those with no ophthalmic imaging (day 1 of the imaging study median ages: OCT imaging = 58, CFP imaging = 55, no imaging = 59, Kruskal–Wallis *P* value ≈ 0), differed in terms of genetic sex (OCT imaging = 52.8% female, CFP imaging = 54.8% female, no imaging = 54.4% female, *χ*^2^ *P* value = 2.62 × 10^−14^), were less likely to identify as White ethnicity (OCT imaging = 92%, CFP imaging = 94%, no imaging = 96%, *χ*^2^ *P* value ≈ 0) and were less wealthy (median Townsend score for OCT = −1.84, CFP = −1.89, no imaging = −2.27, Kruskal–Wallis *P* value = 1.83 × 10^−175^). Visualizations of the population characteristics are presented in Supplementary Figs. 3–6.

### Ret-AAE embeddings are associated with ophthalmic anatomy, function, genetics and disease

We performed three ‘positive control’ studies to explore the representation of retinal features within Ret-AAE embeddings (denoted ‘*z*_*i*_’, where *i* is the relevant latent dimension): (1) Pearson correlation analysis to assess associations between ophthalmic imaging embeddings and ophthalmic anatomical or functional features, (2) Welch’s *t*-test to compare ophthalmic imaging embeddings based on the presence or absence of ophthalmic disease and (3) Cox proportional hazard modeling to examine whether the embeddings predict future ophthalmic disease. In addition, expecting to reproduce the findings of genome-wide-association studies (GWAS) for conventional ophthalmic imaging traits, we conducted GWAS of the 512 Ret-AAE embeddings.

These ‘positive control’ studies yielded results showing that our models effectively capture and represent key ophthalmic features. See the ‘Controlling for Type 1 Error Risk’ section of this paper for details of our approach to multiple comparisons.

The Pearson correlation study was performed between latent features and 50 ophthalmic traits (for example, conventional ophthalmic imaging-derived traits, visual acuity, refractive error, intraocular pressure and corneal biomechanics). Reassuringly, we detected extensive multiple-testing-corrected significant associations between Ret-AAE embeddings and the breadth of ophthalmic features analyzed (Supplementary Tables 1–3 and Supplementary Figs. 7–9). Exploring the association between Ret-AAE embeddings and baseline ophthalmic disease, we revealed that Ret-AEE embeddings are associated with all ophthalmic diseases tested (Supplementary Figs. 10 and 11). The largest number of associations exceeding a Bonferroni significance threshold was observed for cataracts, vitreous disorders, glaucoma, retinal detachments and breaks, and other retinal disorders. In addition, we found that CFP- and OCT-derived embeddings predicted post-imaging ophthalmic disease, including disorders of the lens, choroid, retina and vitreous humor (Supplementary Figs. 12 and 13). Complete positive control analyses are available in Supplementary Tables 2–7.

With a view to exploring the biology captured by Ret-AAE embeddings, we conducted a series of genetic analyses. The OCT-derived embedding discovery GWAS contained 30,863 individuals, with replication in 6,847 individuals (Methods). The CFP-derived embedding discovery GWAS included 19,109 individuals, with replication in 4,402 individuals. These GWAS detected significant associations with numerous genetic variants identified in studies of conventional ophthalmic imaging traits and revealed previously unreported variants of interest.

**Extended Data Fig. 3:**
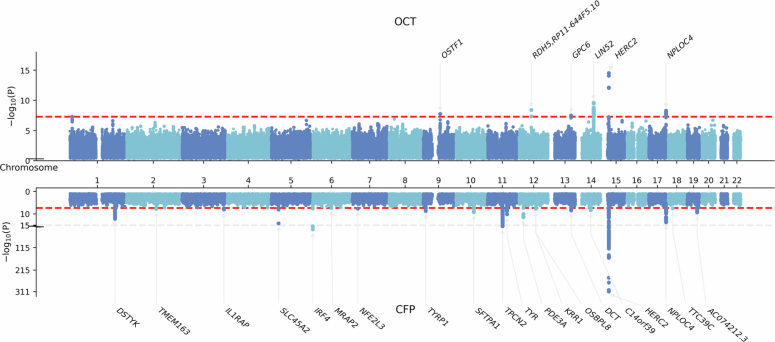
GWAS Miami plot. A Miami plot of GWAS results for both CFP and OCT embeddings. In the interest of creating a single illustrative figure, this Miami plot contains the top SNPs from all embeddings identified to have a polygenic architecture using LD score regression. The superior plot is for OCT-derived embedding associations, whilst the inferior plot is for CFP-derived embedding associations. Note that due to the asymmetry in the axis, the CFP plot has been truncated, with the point of truncation indicated on the y axis. The red line indicates the genome-wide significance threshold, and all annotated variants exceed this threshold.

Following fine mapping Genome-wide Complex Trait Analysis Conditional and Joint Analysis (GCTA COJO), the GWAS of the 256 OCT-derived embeddings highlighted genome-wide significant associations for 33 different single nucleotide variants across 39 embeddings^12^. Of these, 9 replicated in the validation study (across 13 embeddings; Extended Data Fig. 3 and Supplementary Table 8). Several GWAS hits are implicated in pigmentation, including *TYR*, *OCA2*, *DCT* and *TSPAN10* (refs. ^13–16^). In addition, we identified significant signals in genes with core roles in retinal development and physiology, namely *RDH5* (which encodes an enzyme with roles in the visual cycle) and *VSX2* (a retinal transcription factor). Most detected variants have been previously identified in conventional ophthalmic trait GWAS, with the exception of *OSTF1* (a protein produced by osteoclasts, which has previously been linked to retinitis pigmentosa risk) and *DCT* (previously linked to albinism)^17,18^. Linkage disequilibrium (LD) score regression indicated that just seven (2.73%) OCT-derived embeddings had a polygenic architecture, with a heritable component (range = 0.39%–5.13%, median = 3.46%), which may reflect statistical power, multifactorial inheritance patterns, environmental influence, technical factors and/or that some embeddings are distributed according to right eye features (note that the GWAS was conducted using left eye features).

GWAS of the 256 CFP-derived embeddings identified 51 independent genome-wide significant variants across 80 embeddings. Of these, 13 replicated in the validation study (across 53 embeddings) (Supplementary Table 9). Again, several of these variants were in genes with key roles in pigmentation (*TYR*, *OCA2*, *DCT* and *IRF4*)^19^. In addition, we identified a vasculature-associated signal in *PDE3A*. This gene encodes a protein that regulates vascular smooth muscle contraction and relaxation, which was previously linked to retinal vasculature features, aortic root size and blood pressure^20^. There were structural imaging–genetics associations with *TMCC2* (a gene associated with machine learning-derived age-gap measures), *RXFP3* (a gene associated with aging-related disorders) and *IL1RAP* (a component of the interleukin-1 receptor complex, associated with ocular disease)^21–23^. Overall, 23 embeddings (8.98%) had a polygenic architecture with the heritability of polygenic CFP-derived embeddings exceeding that of OCTs, and ranging from 3.12% to 15.5% (median = 5.75%).

**Extended Data Fig. 4:**
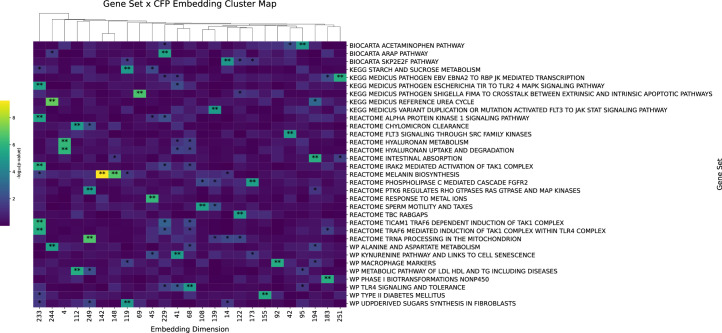
CFP gene set analysis cluster plot. A cluster plot of MAGMA results for our CFP-derived embedding analysis. A single asterisk indicates nominal significance, whilst a double asterisk indicates multiple testing corrected significance. The figure contains gene sets that did not replicate in the validation study (that is these are discovery results). Only gene sets and embeddings with at least one multiple testing significant results are plotted.

**Extended Data Fig. 5:**
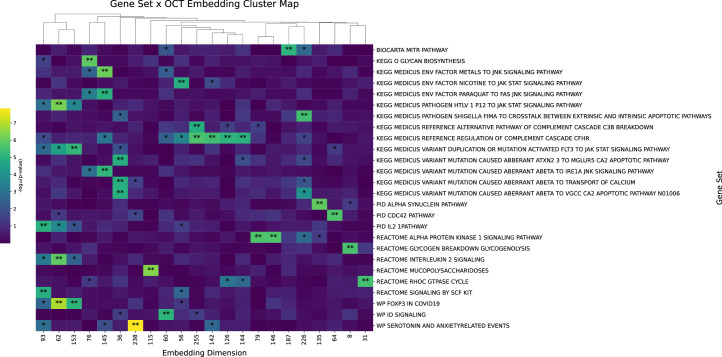
OCT gene set analysis cluster plot. A cluster plot of MAGMA results for our OCT-derived embedding analysis. A single asterisk indicates nominal significance, whilst a double asterisk indicates multiple testing corrected significance. The figure contains gene sets that did not replicate in the validation study (that is these are discovery results). Only gene sets and embeddings with at least one multiple testing significant results are plotted.

Gene set analysis using multi-marker analysis of genomic annotation (MAGMA) revealed 33 multiple-testing-corrected significant gene sets with associations spanning 25 CFP-derived embeddings and 26 gene sets with associations spanning 22 OCT-derived embeddings^24^. Of these, four CFP- and six OCT-associated gene sets replicated in the validation study. For OCT-derived embeddings, replicated gene sets included ‘KEGG MEDICUS variant duplication or mutation activated Fms-like tyrosine kinase 3 (FLT3) to Jak-STAT signaling pathway’, associated with a choroid-localized embedding (*z*_153_, beta = 1.71, *P* value = 6.00 × 10^−6^); ‘KEGG MEDICUS reference regulation of complement cascade *CFHR*’, associated with a choroid- and retinal pigment epithelium-localized embedding (*z*_126_, beta = 2.23, *P* value = 9.00 × 10^−6^); and ‘WikiPathways (WP) FOXP3 in COVID19’, also associated with a choroid-localized embedding (*z*_153_, beta = 0.86, *P* value = 9.00 × 10^−6^). The gene sets identified fit with our understanding of the importance of pigmentation, the complement system and fibroblast growth factor receptors in ophthalmic biology, as well as revealed pathways warranting further exploration in dedicated studies^15,25^. For CFP-derived embeddings, the replicated pathways included ‘Reactome melanin biosynthesis’, which was associated with fundal pigmentation and illuminance, and optic nerve head-localized embeddings (*z*_142_, and *z*_148_, beta = 1.86–2.27, *P* value = 5.56 × 10^−10^ to 1.81 × 10^−7^), and ‘Reactome phospholipase C mediated cascade fibroblast growth factor receptor 2 (FGFR2)’, associated with a vasculature branching complexity and optic nerve head color or pallor-localized embedding (*z*_173_, beta = 0.9, *P* value = 6.66 × 10^−6^). Cluster maps of MAGMA results are presented in Extended Data Figs. 4 and 5, and the complete results are presented in Supplementary Tables 10 and 11. Further Ret-AAE embedding-associated pathways with relevance to neurodegenerative disease were identified and are discussed in the relevant section below.

Collectively, our GWAS findings illustrate that Ret-AAE embeddings are associated with established ophthalmic disease-related genes as well as genes associated with biological age discrepancies, pigmentation and vascular physiology. The complete GCTA COJO results can be viewed in Supplementary Tables 12 and 13. Heritability analyses are presented in Supplementary Tables 14 and 15. Gradient-weighted class activation mapping (Grad-CAM) and latent feature traversals linking embeddings to human-interpretable features are presented in Supplementary Figs. 14–68.

### Associations between ophthalmic imaging features and cardiovascular disease

**Fig. 2:**
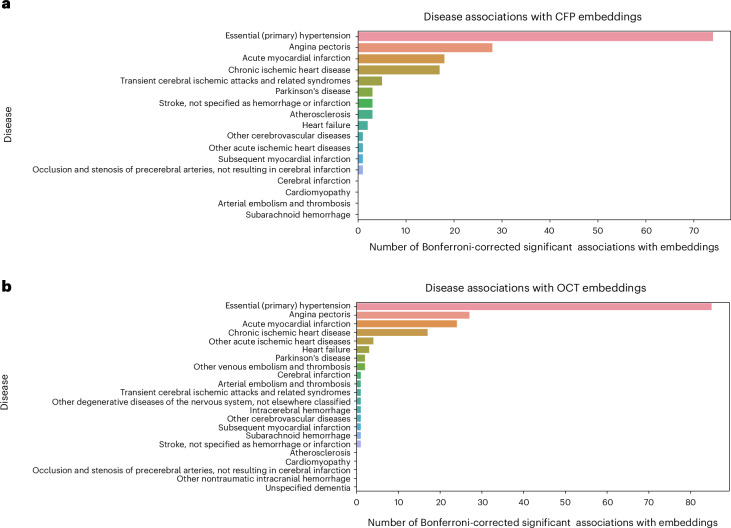
Ret-AAE embeddings and established disease. **a**,**b**, Bar plots showing the number of multiple-testing-corrected significant associations between CFP-derived (**a**) and OCT-derived (**b**) embeddings and neurodegenerative and cardiovascular diseases at the time of baseline imaging (two-sided *t*-test). Individual *P* values are presented in Supplementary Tables 16 and 17 and can be referred to for appraisal of specific tests and the strength of significance.

CFP- and OCT-derived embeddings were associated with numerous cardiovascular diseases at baseline (that is, at the time of first ophthalmic imaging), following correction for age and genetic sex (Supplementary Tables 16 and 17). The largest numbers of significant associations were seen for ischemic heart disease and hypertension (Fig. 2). Smaller numbers of significant associations were seen for cerebrovascular disease and heart failure. There were no significant associations between Ret-AAE and cardiomyopathy. Grad-CAM and latent feature traversals suggested that cardiovascular disease-associated embeddings (for example, CFP *z*_9_, *z*_20_, *z*_63_, *z*_148_, *z*_154_ and *z*_218_; OCT *z*_173_, *z*_209_ and *z*_218_) localized to the retinal vasculature, optic nerve head (for example, cup to disc ratio), the choroidal layer, neurosensory retina and background features (potentially pigmentation or choroidal features).

**Fig. 3:**
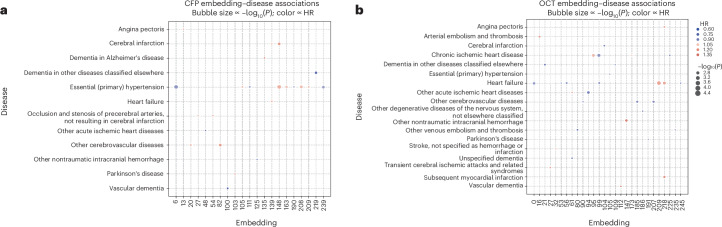
Ret-AAE embeddings and future disease risk. **a**,**b**, Bubble plots illustrating the results of our CFP-derived (**a**) and OCT-derived (**b**) embedding Cox proportional hazards analysis. Only embeddings and disorders with at least one multiple testing-corrected significant result are plotted here. The size of the bubbles indicates the level of significance (larger bubbles = greater significance, unadjusted). The bubbles are color coded according to the hazards ratio (HR). Individual *P* values are presented in Supplementary Tables 18 and 19 and can be referred to for appraisal of specific tests and the strength of significance.

CFP- and OCT-derived embeddings were predictive of future risk of a wide range of cardiovascular diseases including hypertension, stroke, angina pectoris, ischemic heart disease and heart failure (Fig. 3 and Supplementary Tables 18 and 19). Although essential hypertension was associated with numerous CFP-derived embeddings localizing to the vasculature, optic nerve head, fovea and fundal pigmentation (for example, *z*_6_, *z*_148_ and *z*_208_), it was associated with just one OCT-derived embedding, which appeared to localize to the borders of the macula and the choroidal vessels (*z*_105_). Among OCT-derived embeddings, heart failure emerged as the most frequently associated disorder. This was most strongly associated with embeddings localizing to the ellipsoid zone, retinal pigment epithelium, ganglion cell layer, retinal nerve fiber layer, inner nuclear layer and, to some extent, the choroid (*z*_209_ and *z*_218_). The contrasting disease prediction profiles of CFP- and OCT-derived embeddings bring attention to the value that multimodal disease prediction models may hold as a means of measuring diverse cardiovascular health outcomes.

Using LD score regression, we estimated the extent of genetic correlation between Ret-AAE embeddings and cardiovascular disease. This analysis has substantial limitations, given that it can only be conducted on the minority of embeddings with a polygenic genetic architecture and only for traits for which there are well-powered, publicly available GWAS summary statistics. Accordingly, genetic correlation analyses were undertaken against angina, myocardial infarction, heart failure, stroke and systolic blood pressure. We found a few CFP embeddings that were at least nominally associated with cardiovascular disease. All of these localized to the vascular tree and included embedding *z*_220_, which was moderately genetically correlated with myocardial infarction after multiple testing correction (*r*_*xy*_ = 0.38, *P* value = 0.003); embedding *z*_72_, which was nominally significantly genetically correlated with myocardial infarction (*r*_*xy*_ = 0.22, *P* value = 0.01); and embeddings *z*_68_ and *z*_111_, which were nominally genetically correlated with heart failure (*r*_*xy*_ = −0.32 and 0.34, *P* value = 0.04 and 0.04, respectively). These correlations offer some evidence that there is genetic overlap between ophthalmic features and cardiovascular disease, and latent traversals suggested that the genetically correlated ophthalmic features may relate to retinal branching complexity (for example, Supplementary Figs. 24–27). The complete LD score regression results are available in Supplementary Tables 20 and 21.

**Fig. 4:**
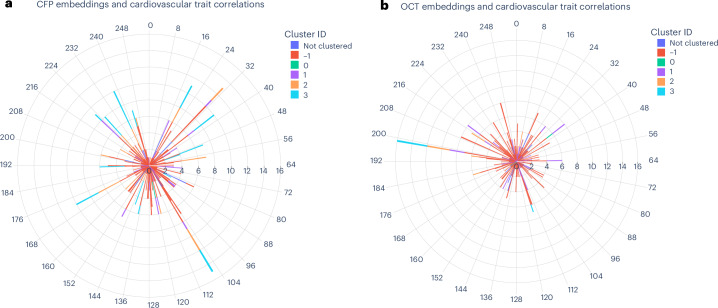
Ret-AAE embeddings and cardiovascular traits. **a**,**b**, Polar plots showing the number of significant relationships (corrected for multiple comparisons) between CFP-derived embeddings (**a**), OCT-derived embeddings (**b**) and cardiovascular traits. The circumferential axis indicates the embedding number. The radial axis indicates the number of multiple testing-corrected significant two-sided Pearson correlations that the embedding has with cardiovascular traits. The bars are color coded according to the cluster of traits the significant relationship belongs to. ‘Cluster 0’ was composed of electrocardiogram intervals (‘PP interval’, ‘RR interval’, ‘QT interval’); ‘cluster 1’ grouped heart rate measures; ‘cluster 2’ included central pulse pressure, peripheral pulse pressure and stroke volume; and ‘cluster 3’ encompassed blood pressure measures. Individual *P* values are presented in Supplementary Tables 23 and 24 and can be referred to for appraisal of specific tests and the strength of significance. HDBSCAN clustering has been used to simplify the presentation of results by grouping traits into statistically defined categories, but readers may wish to explore the more granular trait-wise results in the supplementary content.

Next, we sought to explore how Ret-AEE embeddings relate to cardiovascular anatomy, physiology and function. To that end, we calculated the Pearson correlation coefficient of these embeddings against cardiovascular traits. Hierarchical Density-Based Spatial Clustering of Applications with Noise (HDBSCAN) clustering revealed four cardiovascular trait groups (Supplementary Table 22). ‘Cluster 0’ was composed of electrocardiogram intervals (‘PP interval’, ‘RR interval’, ‘QT interval’); ‘cluster 1’ grouped heart rate measures; ‘cluster 2’ included central pulse pressure, peripheral pulse pressure and stroke volume; and ‘cluster 3’ encompassed blood pressure measures (Supplementary Fig. 69). Associations were of low magnitude but high significance (CFP: 0.06 > *r*_*xy*_ > −0.12; OCT: 0.08 > *r*_*xy*_ > −0.20, excluding small sample sizes). The embeddings were principally associated with ‘noise’-defined features (those that do not fit into a cluster, coded −1), ‘cluster 2’ features and ‘cluster 3’ features (Fig. 4). The significant ‘noise’ results were led by the pulse wave arterial stiffness index, pulse wave reflection index and blood pressure measurements. Notable non-clustered associations included body surface area, left ventricular stroke volume, end-diastolic volume and end-systolic volume. Overall, 24 cardiovascular traits were associated with at least one CFP-derived embedding, and 21 with OCT-derived embeddings. The embeddings with the most cardiovascular associations were vascular traits (OCT embedding *z*_199_, choroid localized; and CFP embedding *z*_106_, vascular tree localized). The traits with the most significant associations with embeddings are presented in Supplementary Figs. 70 and 71. For the full breadth of results, see Supplementary Tables 23 and 24.

To identify ophthalmic imaging-associated metabolomic signatures, which may be of relevance to the association between ophthalmic anatomy and systemic disease, we performed canonical correlation analysis (CCA) between Ret-AAE embeddings and 251 circulating metabolites. The CFP-metabolomic CCA included 16,917 individuals while the OCT-metabolomic analysis included 27,849 individuals. For CFP-derived embeddings, two canonical modes were retained following inspection of the Scree plot (Supplementary Figs. 72–74). Both modes showed moderate correlation between CFP-derived embeddings and metabolomic traits, with the analyses dominated by high-density lipoprotein (HDL) and low-density lipoprotein (LDL) compositional measures (mode 1: *r* = 0.44, permutation *P* value < 0.001; mode 2: *r* = 0.39, *P* value < 0.001). For the OCT embedding–metabolomics CCA, the first canonical mode was evaluated via permutation testing (Supplementary Figs. 75 and 76). This revealed moderate correlation (*r* = 0.36, *P* value < 0.001) and was again dominated by HDL-, LDL- and very low density lipoprotein-related features. Notably, ‘cholesterol in large HDL’ had the highest CCA weighting by a substantial margin. The complete results of CCA are presented in Supplementary Tables 25–27. Seeking to validate our results under statistical assumptions that allow for high correlation among metabolomic data, we conducted a further embedding–metabolic association study using canonical sparse partial least squares (SPLS). SPLS reaffirmed the association between CFP embeddings, and HDL and LDL compositional measures, while SPLS for OCT embeddings recapitulated associations between embeddings and HDL-, LDL- and very low density lipoprotein-related metabolites, while also indicating a substantial contribution of creatinine to the model that was not identified in CCA. Although we did not explore the relationship with renal parameters in this study, the creatinine feature importance is in keeping with a recognized link between OCT features and renal health. SPLS hyperparameter testing, loading and cluster plots are presented in Supplementary Figs. 77–84 (ref. ^26^). These results suggest that anatomical features represented by Ret-AAE embeddings are associated with lipid metabolism. Given the recognized centralism of lipid metabolism in cardiovascular disease, these findings highlight circulating lipoprotein composition as a shared biological axis between ophthalmic structure and systemic cardiometabolic health^27,28^.

### Associations between ophthalmic imaging features and neurodegenerative disease

Ret-AAE-derived embeddings were associated with Parkinson’s disease at baseline imaging, at a level exceeding a multiple-comparisons-corrected threshold (Supplementary Tables 16 and 17). The embeddings most strongly associated with Parkinson’s disease (for example, CFP *z*_148_, OCT *z*_15_) localized to the optic nerve head, retinal nerve fibre layer (RNFL) thickness and ellipsoid zone (photoreceptor layer).

Ret-AAE embeddings were also associated with future risk of several neurodegenerative diseases, after correction for age and genetic sex. Both CFP- and OCT-derived embeddings were predictive of future risk of Parkinson’s disease, vascular dementia and dementia in other diseases (inclusive of Pick disease, Creutzfeldt–Jakob disease, Parkinson’s disease, Huntington disease, human immunodeficiency virus and others). Alzheimer’s dementia was associated with CFP-derived embeddings (for example, vasculature- and optic nerve head-localized CFP-*z*_103_), but not OCT-derived embeddings. With a view to observing the sensitivity of our results to selection of latent dimension size, we analyzed the same associations in relation to 128-dimensional representations of OCT and CFP images. The analysis identified similar associations across neurodegenerative and cardiovascular disease, showing the consistency of association themes (Supplementary Figs. 85 and 86).

The LD score regression analysis was highly limited by the public availability of sufficiently polygenic neurodegenerative disease GWAS. However, it was possible to explore the genetic relationship between Alzheimer’s disease and polygenic Ret-AAE embeddings—finding that there was no evidence of significant genetic correlation. Conversely, our gene set analysis revealed interesting relationships between Ret-AAE embeddings and four pathways associated with neurodegenerative disorders. CFP-derived embeddings were associated with ‘WP kynurenine pathway and links to cell senescence’ (*z*_41_, an embedding localizing to numerous CFP features including vasculature branching patterns and optic nerve head morphology, beta = 0.71, *P* value = 8.31 × 10^−6^). OCT-derived embeddings were associated with the ‘Pathway Interaction Database alpha synuclein pathway’ (*z*_135_, loosely localizing to the outer retina, beta = 0.74, *P* value = 1.00 × 10^−6^), KEGG MEDICUS variant mutation-caused aberrant amyloid beta to VGCC-Ca^2+^ apoptotic pathway N01006 (*z*_36_, partially localized to retinal blood vessels and RNFL, beta = 1.25, *P* value = 1.10 × 10^−5^) and KEGG MEDICUS variant mutation-caused aberrant amyloid beta to transport of calcium (*z*_36_, beta = 1.02, *P* value = 1.10 × 10^−5^).

**Fig. 5:**
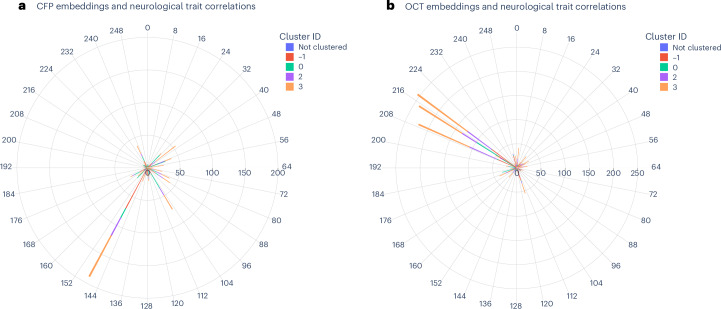
Ret-AAE embeddings and neurological traits. **a**,**b**, Polar plots showing the number of significant relationships (corrected for multiple comparisons) between CFP-derived embeddings (**a**), OCT-derived embeddings (**b**) and neurological traits. The circumferential axis indicates the embedding number. The radial axis indicates the number of multiple-testing-corrected significant two-sided Pearson correlations that the embedding has with neurological traits. The bars are color coded according to the cluster of traits the significant relationship belongs to. ‘Cluster 0’ was principally constituted of gray matter volumes assessed using MRI; ‘clusters 1–3’ were largely constituted of diffusion MRI values. The individual *P* values are presented in Supplementary Tables 29 and 30 and can be referred to for appraisal of specific tests and the strength of significance. HDBSCAN clustering has been used to simplify the presentation of results by grouping traits into statistically defined categories, but readers may wish to explore the more granular trait-wise results in the supplementary content.

HDBSCAN clustering identified four groups of neurological traits. ‘Cluster 0’ was principally constituted of gray matter volumes assessed using magnetic resonance imaging (MRI); ‘clusters 1–3’ were largely constituted of diffusion MRI values (Supplementary Fig. 87 and Supplementary Table 28). This analysis also revealed extensive low-magnitude, but highly statistically significant, correlations distributed across a small number of embeddings (CFP: 0.11 > *r*_*xy*_ > −0.12; OCT: 0.08 > *r*_*xy*_ > −0.23, excluding small sample sizes). The analysis was dominated by significant results in diffusion MRI-dominated ‘clusters 2–3’, with a smaller proportion of multiple testing significant associations with the gray matter volume-dominated MRI ‘cluster 0’ (Fig. 5). Key significant results included OCT-derived embedding *z*_209_ (principally localized to the inner retinal layers, ellipsoid zone and retinal pigment epithelium; 222 multiple-testing-corrected significant results), *z*_215_ (poor localization; 239 multiple-testing-corrected significant results) and *z*_218_ (associated with the thickness and pixel intensity of several retinal layers, including the retinal nerve fiber layer (Supplementary Fig. 68); 255 multiple-testing-corrected significant results), and for CFP-derived embedding *z*_148_ (optic nerve head, background feature and projection artifact localized; 189 multiple-testing-corrected significant associations). OCT-derived embeddings had substantially more significant associations with neurological traits than CFP-derived embeddings. Fluid intelligence score (the capacity to solve problems that require logic and reasoning ability, independent of acquired knowledge) and reaction times showed the largest number of significant associations with imaging latent features, followed by cerebral volume measures. Supplementary Figs. 88 and 89 illustrate the traits with the largest number of significant associations with embeddings. Overall, 459 neurological traits had significant associations with at least one OCT-derived embedding, and 390 with at least one CFP-derived embedding (Supplementary Tables 29 and 30).

As documented previously, Ret-AAE embeddings were also correlated with lipid-related metabolites. Although these data have been presented in relation to cardiovascular disease, lipids are crucial for the normal development and function of the central nervous system, and lipid metabolism has been implicated across the breadth of neurodegenerative disease^29,30^. Therefore, the associations between Ret-AAE embeddings and lipid metabolism are of equal interest as a potential axis of association between ophthalmic features and neurodegenerative disease.

## Discussion

In this study, we present an integrative multi-omic framework to investigate the links between deep learning-derived ophthalmic imaging features (Ret-AAE embeddings) and systemic disorders. Leveraging comprehensive physiological, anatomical, radiomic, metabolomic and genomic analyses, we provide converging evidence that the observed association between ophthalmic features and systemic disease reflects diverse processes.

We show that OCT and CFP features are associated with a range of cardiovascular and neurodegenerative disorders, both at the time of imaging and as predictors of future disease. Specifically, Ret-AAE embeddings are associated with future heart failure, hypertension, ischemic heart disease, cerebrovascular disease, Parkinson’s disease and dementia, mirroring existing evidence of the predictive power of ophthalmic imaging for neurodegenerative and cardiovascular diseases^1,2,8,9,31,32^. As such, this work builds upon evidence supporting the concept of the eye as a diagnostic window into cardiovascular and neurological health. Furthermore, our study corroborates and extends existing evidence that the ophthalmic features represented in OCT and CFP images offer distinct, complementary insights into systemic health. A previous study^9^ has indicated that CFP and OCT images do not necessarily predict systemic disease with equal accuracy. In this study (RETFound), it was shown that CFPs offered the most value with respect to stroke prediction, and OCTs in relation to Parkinson’s disease^9^. In our study, we showed that both CFP and OCT features are associated with cerebrovascular and cardiovascular outcomes, but they differ with regard to the breadth and distribution of these associations. This was apparent throughout our multi-omic analysis, in which OCT features had substantially more neurological trait associations, while CFPs had more cardiovascular associations, and with regard to future disease risk, in which OCTs led by heart failure associations and CFPs by essential hypertension associations.

Saliency maps and latent feature traversals principally implicated the choroid and vascular tree in relation to cardiovascular associations, and the optic nerve head, neurosensory retina and vasculature in relation to neurological associations. Notably, the prominence of choroidal features in our analysis contrasts with the relative paucity of research into choroidal biomarkers for cardiovascular disease^2^. This suggests that further study is warranted, particularly using imaging modalities that visualize the choroid with greater resolution (for example, swept-source OCT). In some cases, associations were mapped to projection artifacts (for example, cataract) and CFP background features (for example, pigmentation or the choroidal vasculature). These associations may reflect variation associated with ancestral background, sociodemographic factors and age. While such variation is of relevance to disease prediction, this finding highlights the importance of considering confounding anatomical factors when studying image-derived phenotypes^33^.

Genetic analyses revealed that Ret-AAE features are enriched for genes in pathways implicated in neurodegenerative disease, including those related to Parkinson’s disease (for example, alpha-synuclein), Alzheimer’s disease (for example, amyloid-beta-related pathways) and broader neurodegeneration (for example, the kynurenine pathway, linked to Huntington’s disease, amyotrophic lateral sclerosis and dementia)^34,35^. These findings are in keeping with postmortem studies demonstrating that amyloid and alpha-synuclein deposits can be shown in the retina, and may be correlated with the extent of neurodegenerative disease^36–38^. In addition, we observed a statistically significant genetic correlation between cardiovascular traits and vascular tree-localized ophthalmic features, suggesting that some ophthalmic features share a genetic architecture with cardiovascular health. Physiologically, our analyses indicated that ophthalmic features are associated with multiple cardiovascular phenotypes, including blood pressure, pulse pressures, arterial stiffness and cardiac cycle functional volumes. Radiomic analyses revealed that there are broad associations between ophthalmic features and both global and regional cerebral volumes, as well as with cerebral tissue microstructure assessed using diffusion MRI. Metabolomic analysis showed that both OCT- and CFP-derived features are correlated with lipid-related metabolites, suggesting a shared metabolomic axis between ophthalmic features and neurodegenerative and cardiovascular disease. Collectively, these findings are illustrative of the wide-ranging and complex potential intermediaries that may link ophthalmic features to systemic health. While few studies have taken a multi-omic approach to elucidate the biological underpinnings of deep learning-derived retinal phenotypes, our findings reinforce previous evidence linking ophthalmic features to neurological and cardiovascular structure, systemic physiology, metabolomic pathways and clinical risk factors^10,39–42^. Furthermore, this study advances the application of deep learning-derived phenotyping approaches in large-scale biological and clinical research^10,39,43,44^.

This study has several limitations. While the UKB was designed to be broadly representative of the ageing UK population, it is not without some inevitable selection biases. All reported associations should therefore be interpreted considering this limitation, which may apply to both the cohort as a whole and the missingness of individual measures within the population^45^. Specifically, it is possible that the relationship between the eye, heart and brain may differ in cohorts with a higher burden of disease, of more heterogeneous ancestry or of different age distribution (for example, those below 40 years of age, who are excluded from the UKB study). Secondly, Ret-AAE used a 2D representation (the central slice) of OCT images, rather than exploring relationships across the entire 3D macular representation. This simplification, necessitated by computational constraints, risks overlooking relationships with paracentral OCT features and macular asymmetries. We did not seek to explore pharmacological factors within this study, and it is therefore possible that some oculo-systemic associations are pharmacologically mediated, a line of inquiry that may be worthy of further study. This study sought to explore mechanistic links between ophthalmic features and systemic health, but the study methodology was not designed with clinical applications in mind, and the distinction between statistical and clinical significance should be noted. The study was conducted exclusively in the UKB population owing to the limited availability of multi-omic studies inclusive of ophthalmic imaging, and therefore, the generalizability of our findings to other populations has not been tested. Finally, with the intention of balancing type 1 and type 2 error rates, our multiple-testing correction was applied considering each embedding as a family (see section ‘Controlling for Type 1 Error Risk’); the results may therefore benefit from follow-up in external validation studies, although the risk of type 1 error is reduced by the convergence of evidence across the diverse analyses conducted in this study.

In summary, this study provides a framework for interpreting the specific systemic pathways that underpin observed imaging–disease associations with respect to their genetic, metabolic, anatomical and functional characteristics. Here this was achieved using AAE-derived imaging features, but future research may seek to compare the performance of this approach with alternative methods, such as the use of variational autoencoders or other state-of-the-art vision learners^11,46^. By offering enhanced biological interpretability of these associations, our findings can inform future studies aiming to model cardiovascular and neurological disease using ophthalmic imaging, and support the development of explainable deep learning tools for personalized medicine approaches. Finally, through delineation of the spectrum of neurodegenerative and cardiovascular disorders associated with ophthalmic imaging features, we expect this study to guide future models with respect to the diversity of health outcomes that can be modeled.

## Methods

### Ethics statement

The UKB study was conducted with the approval of the North-West Research Ethics Committee (ref 06/MRE08/65), in accordance with the principles of the Declaration of Helsinki; all study participants gave written informed consent and were free to withdraw at any time^47^. This project used data from the UKB study under approved project numbers 53144, 49978 and 11350. All retinal images shown in this paper are reproduced with the permission of UKB.

### Cohort characteristics

UKB is a prospective population-based study of 502,355 participants. Baseline examinations were carried out at 22 study assessment centers between January 2006 and October 2010. Study participants have undergone a detailed assessment of demographic, lifestyle and clinical measures; provided DNA samples via blood tests; and provided a range of physical measures. A substantial subset of volunteers underwent an ophthalmic assessment (23%, *n* = 117,279). Of these participants, 67,664 underwent spectral domain OCT between 2006 and 2010 (‘instance 0’) and an additional 17,090 participants were imaged for the first time during the first repeat assessment between 2012 and 2013 (‘instance 1’)^48^. UKB data are organized into data categories and fields, and we provide data category and field ID numbers in brackets for reproducibility. The characteristics of those with and without imaging were compared using the Kruskal–Wallis and *χ*^2^ tests.

### Imaging quality control

Low-quality OCT scans were excluded on the basis of an image quality score below 40 (ref. ^15^). The quality score used was that which is native to the Topcon OCT device. CFPs were excluded if they were of ‘reject’ quality grading assessed using a deep learning method, ‘Automorph’ (‘usable’ and ‘good’ images were retained)^49^. Automorph was trained using the EyePACS-Q dataset, using grading provided by two experts according to image illumination, artifacts and diagnosability of ocular diseases; the full method is documented in the referenced publication.

### Image preprocessing

For OCTs, FDS files were converted to an analyzable format using the oct-converter python package^50^. OCT slice 64 was retained for analysis. CFPs were cropped and segmented using Automorph, and the binary vasculature segmented images were retained for the purpose of refining the autoencoder loss function defined below. Imaging data were randomly split approximately 70:15:15 into train:test:validate allocations for the purpose of training deep learning models. Images were partitioned so that each study participant’s scans were paired within the same allocation.

### Autoencoder model architecture and training

#### Image transformations

Several transformations were applied to OCTs and CFPs during the autoencoder training process. To reduce computational burden, images were resized to 224 × 224 using bicubic interpolation. To improve model generalizability, several further transformations were applied during training, namely, random horizontal flips, random rotations (0–15°), random color jitter (brightness = 0.9–1.1, contrast = 0.9–1.1, saturation = 0.9–1.1).

#### Model architecture

We designed an AAE for the purpose of creating compressed representations of retinal images (CFP and OCT). The AAE architecture is regularized by matching the aggregated posterior, *q*(*z*), to an arbitrary prior, *p*(*z*). We selected a Gaussian prior *N*(0,1), and the resulting vector (*z*) approximates a multivariate Gaussian distribution^51^. This property is beneficial for downstream analyses, many of which have a normality assumption. Our AAE was trained in a laterality agnostic manner, but separate AAEs were trained for OCTs and CFPs. The model encoder consisted of each layer of the encoder consisting of a 2D convolution, a batch normalization layer, a Leaky Rectified Linear Unit (ReLU), a multi-scale residual block (kernel sizes 3 and 5) and an efficient multi-scale attention module^52,53^. We projected our image to a 256-dimensional vector using a linear layer at the bottleneck. Our decoder consisted of a single linear layer for up sampling our vector, followed by 2D transposed convolutions, batch normalization, Leaky ReLU activations, multi-scale residual blocks and efficient multi-scale attention modules^52,53^. Our adversarial discriminator was composed of three linear layers and used Leaky ReLU activation functions.

#### Loss functions

For training both the OCT- and CFP-AAE, we used the mean absolute error (also known as L1 loss) and adversarial loss. CFPs are relatively low contrast images, which are dominated by a narrow background color distribution (the retina). For our purposes, the retinal vasculature is an important structure that must be represented in the latent space. In our first iterations of training our model, the AAE failed to reconstruct the vasculature to an acceptable extent, owing to the vasculature contributing little to the L1 loss (for example, Supplementary Fig. 90). Using Automorph, we segmented the CFP images and produced binary masks of the vasculature^49^. On average, the vasculature represented just 4.07% of the pixels in CFPs. Accordingly, for the CFP-AAE, we weighted the L1 loss to attribute 12.29× more weight to pixels containing blood vessels on Automorph binary masks. In doing so, CFP vasculature structures then contributed 50% of the L1 Loss on average. In addition, we implemented a perceptual loss function for the CFP-AAE. In a previous systematic comparison of deep perceptual loss functions, the 4th ReLU of VGG16 (without batch normalization) was shown to contribute to the best performance in autoencoding tasks, and, accordingly, this model was selected for our perceptual loss function^54^. To calculate a perceptual loss, original images and AAE reconstructions were normalized according to ImageNet parameters before being passed to VGG16 without batch normalization^55^. The mean squared error loss of the feature maps of the reconstructed and original images then served as the perceptual loss. Finally, with a view to normalizing the latent vectors produced by the model, we used an adversarial loss function. Briefly, this was calculated by training a discriminator network to distinguish between the latent vectors of the AAE model and a standard normal distribution. To improve the stability of the adversarial network, we used one-sided label smoothing^56^. The weight provided to each constituent of the loss function was determined using a Bayesian hyperparameter sweep as described below. In our CFP-AAE, terminal arterioles and venules were lost in reconstruction. Although we identified that the SSIM improved with expansion of the latent space (for example, 128-dimensional vectors resulted in an SSIM of 0.876, and 256-dimensional vectors in 0.891), in the interest of preserving the statistical and computational feasibility of downstream tasks, we did not expand the latent space dimensionality further in the pursuit of terminal vascular reconstruction.

#### Image quality metrics

Image quality metrics used included the PSNR, SSIM and the L1 loss^57^.

#### Hyperparameter search

We performed a Bayesian hyperparameter sweep using the Weights and Biases platform (version 0.19.8)^58^. The SSIM in the test dataset guided the Bayesian optimization. For both CFP and OCT AAEs, the search was used to select the discriminator learning rate, the autoencoder learning rate, the number of convolutional layers, the optimizer weight decay and the weighting of the adversarial loss. For the CFP sweep, we additionally included the perceptual loss weighting. The sweep was conducted for 35 epochs per hyperparameter combination. For the CFP-AAE, the sweep-identified optimal hyperparameters included 5 convolutional layers, a weight decay of 0.01 (AdamW optimizer), an autoencoder learning rate of 0.0005, a discriminator learning rate of 0.0001, a weighting of 0.2 for the perceptual loss and a weighting of 0.001 to the discriminator loss^59^. For the OCT-AAE, the optimal hyperparameters included 4 convolutional layers, a weight decay of 0.001 (AdamW optimizer), an autoencoder learning rate of 0.0001, a discriminator learning rate of 0.00005 and a weighting of 0.0001 for the discriminator loss.

#### Additional AAE experimental details

Experiments were conducted using PyTorch^60^. We used a learning rate to warm up over the first 15 epochs. The batch size was 32. A random seed of value 42 was used. The CFP model was trained for 400 epochs, and the OCT model was trained for 250. For sensitivity analysis, we trained a second OCT and CFP model with 128 dimensions, using the same hyperparameters as those used in our main analysis. Our models were trained using 4 tesla T4 graphics processing units (GPUs). Randomly selected reconstructions were examined by two ophthalmologists (T.H.J. and P.I.S.) to ensure that critical structures were adequately represented in reconstructed images.

### OCT- and CFP-AAE phenotypic association tests

We used latent vectors representing the left eye (OCT and CFP) of UKB study participants as a means of reducing the dimensionality of the analysis. In keeping with previous work, the left eye was selected because the relevant images are systematically of higher quality than those from the right eye^11,15^. The total number of study participants varied according to the requirements of the association test, and are reported in text where possible, or in the supplementary content in which the study participant number varied according to individual phenotype. Both instance 0 and 1 ophthalmic images were used in these analyses (except where explicitly stated otherwise), and where a study participant had undergone imaging during both instances, only their first set of images were considered.

#### Pearson correlation analyses

The ‘positive control’ Pearson correlation analyses included all traits in the UKB eye measures category (category 100013), excluding right eye measures.

The experimental tests featured a combination of anatomical and functional cardiovascular and neurological traits. Specifically, this featured all traits in the following UKB catalogs: brain MRI (category 1014), cognitive function (category 1005), heart MRI (category 1015) carotid ultrasound (category 101) and selected traits from the physical measures category (category 1006). There was a total of 50 cardiovascular traits and 892 neurological traits. The large number of neurological traits reflects the scale of derived neuroimaging features available in UKB. To enhance theme-wise interpretability in plots, we used HDBSCAN clustering^61^. Traits present in an overlapping, large number of study participants were clustered, and the remainder of the traits were assigned to a ‘not clustered’ category—but were still considered in subsequent analyses. Data were scaled according to the interquartile range using ‘RobustScaler’ in SciKit Learn^62^. Clusterable features included 35 ophthalmic traits (*n* = 62,729), 889 neurological features (*n* = 12,219) and 37 cardiovascular traits (*n* = 11,295). A leaf clustering method was used. Cardiovascular and ophthalmic traits were fewer in number, so a minimum cluster size of 3 was used. Neurological traits were assigned a minimum cluster size of 10 given the larger number of traits.

For each analysis, outlier cardiovascular, neurological or ophthalmic values were excluded. This was achieved by excluding data greater than 2.5 standard deviations from the mean. Pearson correlation coefficients and accompanying *P* values were then calculated using the SciPy python package^63^.

#### Cox proportional hazards and Welch’s *t*-test analyses

Disease outcomes were derived from the ‘First Occurrences’ UKB catalog (category 1712). The UKB first occurrences were generated by mapping read code information in the ‘Primary Care data’ (category 3000), ICD-9 and ICD-10 codes in the ‘Hospital inpatient data’ (category 2000), ICD-10 codes in ‘Death Register records’ (field 40001, field 40002) and ‘self-reported medical condition codes’ (field 20002) reported at the baseline or subsequent UKB assessment center visit. The UKB estimates that hospital inpatient data and death are mostly complete up to 31 May 2022, after which data may be incomplete^64^. Accordingly, we used 31 May 2022 as the censoring date for our time-to-event analysis. For the Cox proportional hazards, study participants who suffered a given disease event before the date of retinal imaging were excluded from analysis. Meanwhile, for our *t*-test, the objective was to determine whether latent features differed significantly according to the presence and absence of disease at the time of scan, and so, for this analysis, those with disease events at the time of scan were included. Disease outcomes included (1) a positive control disease dataset inclusive of diseases that were determined to be identifiable on retinal imaging according to two ophthalmologists (T.H.J. and P.I.S.), including disorders of the retina, choroid, optic nerve and lens (19 diseases); (2) all available neurodegenerative diseases (10 diseases); and (3) all available cardiovascular disorders (23 diseases). The complete list of diseases considered is presented in Supplementary Table 31.

Cox proportional hazards modeling was conducted using the lifelines Python package^65^. The primary outcome of interest was the relationship between left eye Ret-AAE embeddings and time-to-event analyses against ophthalmic, cardiovascular and neurodegenerative diseases. Covariates included genetic sex and age at the time of scan. *t*-tests were conducted using the SciPy package in Python^63^. We elected to use the Welch’s *t*-test, a modification of the Student’s *t*-test, which is more reliable in the context of unequal sample sizes of variances between two populations^66^. Results with fewer than 30 samples per group were excluded from the final analysis in both analyses. We were unable to test the baseline relationship between Ret-AAE embeddings and Huntington’s disease, spinal muscular atrophy and related syndromes, Alzheimer’s disease and vascular dementia owing to insufficient cases (<30) at baseline imaging. Huntington’s disease was additionally excluded from Cox proportional hazards analysis owing to insufficient cases.

#### CCA

CCA was used to explore the relationship between CFP–OCT-derived embeddings and 251 metabolic biomarkers generated by Nightingale Health (category 220)^67^. Owing to study participant overlap in instance 0 and 1 metabolic sampling, only instance 0 data were used in this analysis. Study participants with missing imaging or missing metabolite samples were excluded. Extreme values that may unduly influence the results were managed using Winsorization (applied to the most extreme upper and lower 1% of values)^68^. The analysis was conducted using the SciKit learn CCA model. Embeddings and metabolomic data were scaled using the native CCA scaler. To assess the statistical significance of each canonical variate, we used permutation testing to create a null distribution of correlations. The embedding matrices were held constant, while the metabolomic matrix was substituted for randomly permuted sequences over 1,000 iterations^69,70^. The *P* value was calculated using the number of null correlations that exceeded the average CCA estimated on the original dataset. The number of modes to test in our permutation test was determined by constructing a Scree plot of the first 10 modes and then truncating where there is a substantial reduction to the regression coefficient.

#### SPLS

In addition to CCA, we explored metabolomic data using a canonical SPLS analysis using the mixOmics (version 6.32.0) R library^71^. SPLS is a variant of partial least squares in which a subset of important features is selected for inclusion in the model according to sparsity hyperparameters, rather than including all traits, which may be inappropriate in the context of highly correlated covariates (as is the case for metabolomic data). SPLS was therefore implemented as a means of validating our CCA results in a manner more robust to the structure of our data. Data were prepared in line with the CCA analysis. Lasso penalization was optimized using fivefold cross-validation with five repeats, optimized to maximize correlation between predicted and actual components. Following this, we implemented CFP SPLS with 2 components, 30 embedding variables in component 1 and 10 in component 2, and 25 metabolite variables in component 1 and 10 in component 2. OCT SPLS was implemented with 1 component, with 10 embedding variables and 10 metabolite variables. The analysis was undertaken using the canonical mode, and plots presented with respect to SPLS were all generated using mixOmics.

### Genetic analyses

#### Choice of GWAS method

REGENIE version 4.1 was selected as the method for GWAS^72^. For step 1, we used the UKB microarray data (field 22418). For step 2, we used the imputed data (field 22828).

#### Phenotypes and covariate data

The phenotypes studied were latent features for each study participant’s left eye OCT and CFP images. Therefore, 256 GWAS studies were performed per study participant per scan type (that is, 512 GWAS studies in total). Covariates for GWAS were sex (field 31), age at date of scan (field 22006) and spherical equivalent refractive error. Spherical equivalent refractive error was calculated by identifying the most reliable refractometry result (field 5276), identifying the spherical refractive error (field 5085) and cylindrical refractive error (field 5086), and performing the calculation: spherical equivalent = spherical refractive error + 0.5 × cylindrical refractive error^15^.

#### Quality control for genetic analyses

The genetic analyses in the discovery cohort were conducted in study participants in instance 0 (initial assessment visit, 2006–2010). The genetic analyses in the replication cohort were conducted in study particpants who attended within instance 1 (first repeat assessment visit, 2012–2013). Any overlapping study participants were removed (that is, the cohorts are entirely independent). Analysis was limited to study participants of genetically determined European ancestry according to their principal components (field 22006).

Study-participant-level genetic quality control metrics were applied, including exclusion of participants whose genetic sex (field 22001) did not match their reported sex (field 31), exclusion of participants with sex chromosome aneuploidy (field 22019), exclusion of participants with genetic kinship to other UKB participants (field 2202), exclusion of participants who were outliers for heterozygosity or missing rate (field 22027) and removal of individuals with more than 10% missing genotype data^73^.

Variant-level quality control was conducted using plink2 (ref. ^74^). For the microarray data used in REGENIE step 1, quality control included minimum minor allele frequency of 0.01, minimum minor allele count of 20, removal of variants with >10% missingness and removal of SNPs failing the Hardy–Weinberg equilibrium test at *P* < 1 × 10^−15^. For imputed data used in step 2, quality control included minimum minor allele frequency of 0.01, minimum minor allele count of 20, removal of variants with >10% missingness, removal of SNPs failing the Hardy–Weinberg equilibrium test at *P* < 1 × 10^−6^ and removal of duplicate variants. In addition, following GWAS, any variants with an imputed information (INFO) score below 0.8 were removed.

#### GCTA COJO

To refine the obtained association signals, further analyses were performed using GCTA-COJO^12^. Genetic variants in loci that were on different chromosomes or more than 1,500 kb distant from each other were assumed to be uncorrelated. Otherwise, GCTA COJO default parameters were implemented.

#### Gene set analysis

Gene set analysis was performed using MAGMA^24^. Gene sets were derived from the Human MSigDB Collections^75^. Specifically, we elected to use the canonical pathways from the curated gene sets. The canonical pathways include the BioCarta pathways database (292 gene sets), the KEGG MEDICUS pathway database (658 gene sets), the PID pathway database (196 gene sets), the Reactome pathway database (1,787 gene sets), the WikiPathways pathway database (885 gene sets) and the KEGG (legacy) pathway database (186 gene sets). Variants were annotated to genes based on dbSNP version 135 variant locations and NCBI 37.3 gene definitions. The LD reference file was derived from the ‘European’ subset of the 1000 Genomes Project dataset^76^.

#### Global genetic correlation analysis

The global genetic correlation between OCT and CFP latent features and cardiovascular and neurological disease was assessed using LD score regression (LDSC)^77^. The GWAS summary statistics for cardiovascular and neurological disease were sourced from publicly available studies in the same genome build used in this study (GRCh37) and containing the required information for genetic correlation^78–83^. Only latent features with a mean chi^2^ > 1.02 were considered for LD score regression (the threshold for polygenicity). Our analysis was consistent with the methodology outlined in a previous study^77^, in which genetic variants were filtered according to presence in HapMap3, minor allele frequency > 0.01, INFO score > 0.9 and removal of strand-ambiguous variants and duplicated variants. LD scores were derived from the European subset of the 1000 Genomes Project dataset^76^. The intercept was not constrained in this analysis. LD score regression was also used to determine the heritability of the polygenic latent features, but not an estimate of statistical significance is not available as this is not an output of the LDSC method.

### Grad-CAM

To enhance the interpretability of our learned embeddings, we applied Grad‑CAM^84^. Consistent with previous work, we observed that visualizations from the earliest convolutional layers lacked semantic relevance, whereas those from the deepest layers were overly coarse to localize meaningful image features^85^. Consequently, we focused our primary analyses on the third convolutional layer for both CFP and OCT modalities, which provided the optimal balance between spatial resolution and semantic content. In instances in which Grad‑CAM failed to yield an interpretable map, we instead used Layer‑CAM or, alternatively, targeted adjacent convolutional layers; these exceptions are clearly noted in each figure legend^86^. Saliency maps are only presented for embeddings that are referenced in text. Where possible, we describe embeddings with respect to their anatomical localization to enhance interpretability. Given the abstract nature of embeddings, this is not possible in all cases.

### Latent feature traversals

In addition to Grad-CAM, we performed systematic latent feature traversals of the trained AAE^87^. For each latent dimension (embedding) *z*_*i*_, we first identified a representative reference image whose latent coordinate in that dimension was closest to the mean (that is, approximately zero). This reference latent vector was then held fixed except for *z*_*i*_, which was perturbed independently by ±4 standard deviations. The modified latent vectors were decoded using the AAE decoder to visualize the effect of isolated variation along each latent axis on the reconstructed image.

Because the resulting changes can be visually subtle, we complemented qualitative inspection with quantitative difference maps. For CFPs (three-channel RGB images), we computed the mean change in pixel intensity per color channel relative to the reference reconstruction. For OCT images (single-channel), we computed the absolute per-pixel intensity difference. These visualizations highlight regions most sensitive to variation in each latent dimension and facilitate interpretation of the image features encoded by the learned representation.

The power of autoencoding for feature extraction is that image patterns can be identified that are not necessarily visually apparent to the human eye. Accordingly, many latent traversals result in diffuse pixel intensity changes that are challenging to interpret biologically and clinically. Only figures with human-interpretable features are presented in (Supplementary Figs. 14–68).

Annotations of latent traversals and Grad-CAM saliency maps were provided following independent review by two ophthalmologists (T.H.J. and P.I.S.).

### Controlling for type 1 error risk

We elected to implement family-wise error rate control (Bonferroni-corrected threshold for significance) in this analysis. We examined whether autoencoder embeddings were substantially correlated to guide our definition of a family. As can be seen in Supplementary Figs. 91 and 92, the embeddings showed little meaningful correlation. Accordingly, each embedding was treated as its own family, and multiple testing correction was applied according to the total number of tests within each embedding. Given that this is not a study-wide correction (that is, we do not also correct according to the number of embeddings, which would be prohibitively strict and cause excessive type 2 error given the study scale and hypothesis-free nature of this work), we assess our results and the risk of type 1 error in the context of the convergent evidence presented across analyses, present raw *P* values to allow interpretation by readers, interpret results in the context of their biological plausibility and, where possible, validate results in a separate UKB instance. Although this approach offers a more nuanced perspective on the results, type 1 error remains an area of concern, and the key study findings will benefit from external experimental validation when appropriate external multi-omic datasets become available. The Bonferroni-corrected *P* value thresholds for significance for all other analyses are presented in the appropriate supplementary table legends.

### Statistics and reproducibility

All methodological approaches are described in the relevant sections of the paper. Sample sizes varied by analysis and are reported alongside each result where possible or otherwise provided in the Supplementary Data. No statistical methods were used to predetermine sample size. Instead, all available data passing analysis-specific quality control criteria were included. All analyses were performed using reproducible computational pipelines, as described in the Methods.

### Reporting summary

Further information on research design is available in the Nature Portfolio Reporting Summary linked to this article.

## Supplementary information

Supplementary Information Supplementary Figs. 1–92.

Reporting Summary

Peer Review File

Supplementary Tables Supplementary Tables 1–31.

## Data Availability

The UKB dataset is available under restricted access through a procedure described at http://www.ukbiobank.ac.uk/using-the-resource/. All other data supporting the findings of this study are available in the Article and its online Supplementary Information.

## Code availability

UKB guidance does not permit researchers ‘to (directly or indirectly) feed or enable UK Biobank participant level data to be incorporated into a publicly available Generative AI or similar models’. We are therefore unable to share the Ret-AAE decoder weights. However, we are permitted to share the model encoder and its associated weights, and we have made these publicly available via GitHub at https://github.com/TomJulian/Ret-AAE.
