# Supplementary material for: Multi-omic analysis of deep learning-derived phenotypes links ophthalmic imaging to cardiovascular and neurological traits
Source: Nat Cardiovasc Res. 2026 Jun 16;5(6):541–54. doi: 10.1038/s44161-026-00815-5 (PMC13271892; doi:10.1038/s44161-026-00815-5)
Supplement: Supplementary file 1 — Supplementary Figs. 1–92. [file 44161_2026_815_MOESM1_ESM.pdf]

nature cardiovascular research

Supplementary information

https://doi.org/10.1038/s44161-026-00815-5

Multi-omic analysis of deep learning-derived phenotypes links ophthalmic imaging to cardiovascular and neurological traits

In the format provided by the authors and unedited

Supplementary Figures:

Table of Contents

Embedding Distributions *1*

Population Characteristics Plots *2*

Positive Control Plots *6*

Embedding Feature Visualisations / Saliency Mapping *12*

Cardiovascular Trait Associations *67*

Metabolomic Analyses *69*

Sensitivity Analyses *81*

Neurological Trait Analyses *83*

Early Reconstruction Example *85*

Embedding-Embedding Correlations *86*

UK Biobank Eye and Vision Consortium Membership: *88*

Embedding Distributions

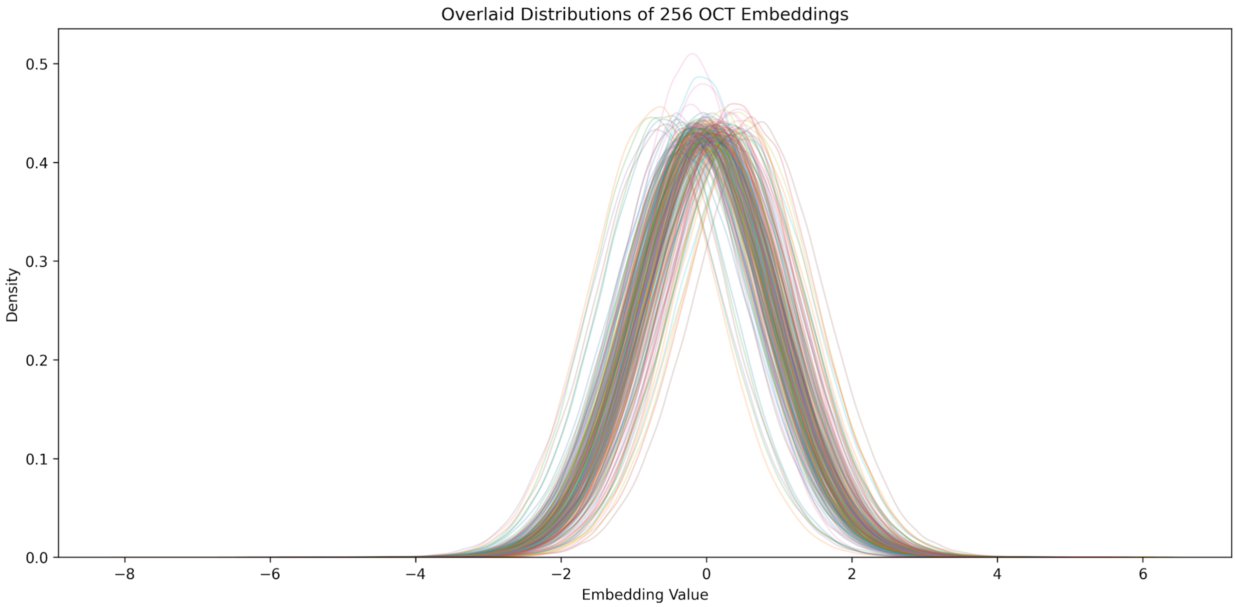

Supplementary Figure 1: A density plot, illustrating that most OCT embeddings approximated a Gaussian distribution.

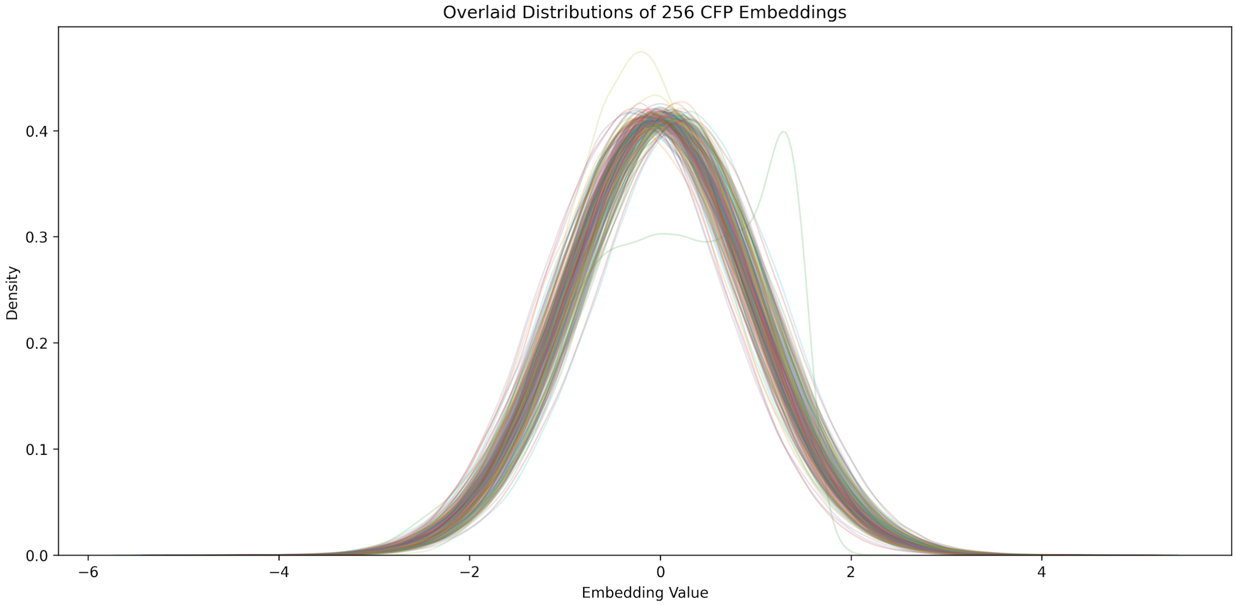

Supplementary Figure 2: A density plot, illustrating that most CFP embeddings approximated a Gaussian distribution.

Population Characteristics Plots

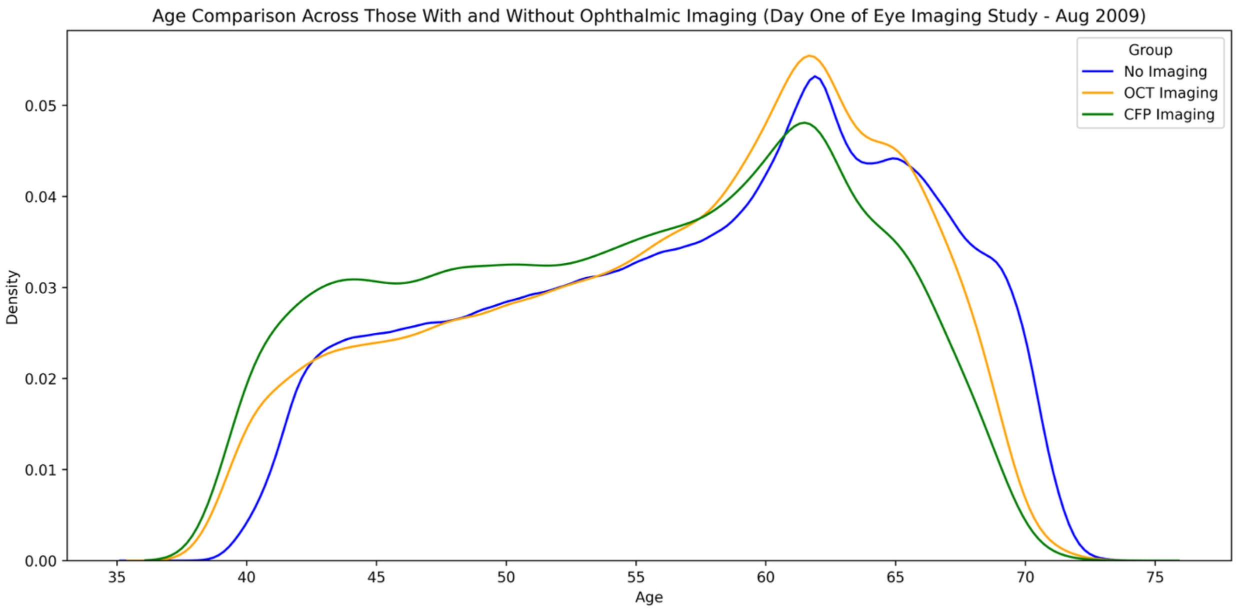

Supplementary Figure 3: A density plot showing the distribution of age of subjects at the commencement of the UK Biobank Eye Imaging Study. The plot compares those without imaging, those with sufficiently high-quality left eye colour fundus photograph (CFP) images, and those with sufficiently high-quality left eye optical coherence tomography (OCT) images. Quality criteria are described in the methods section of the manuscript.

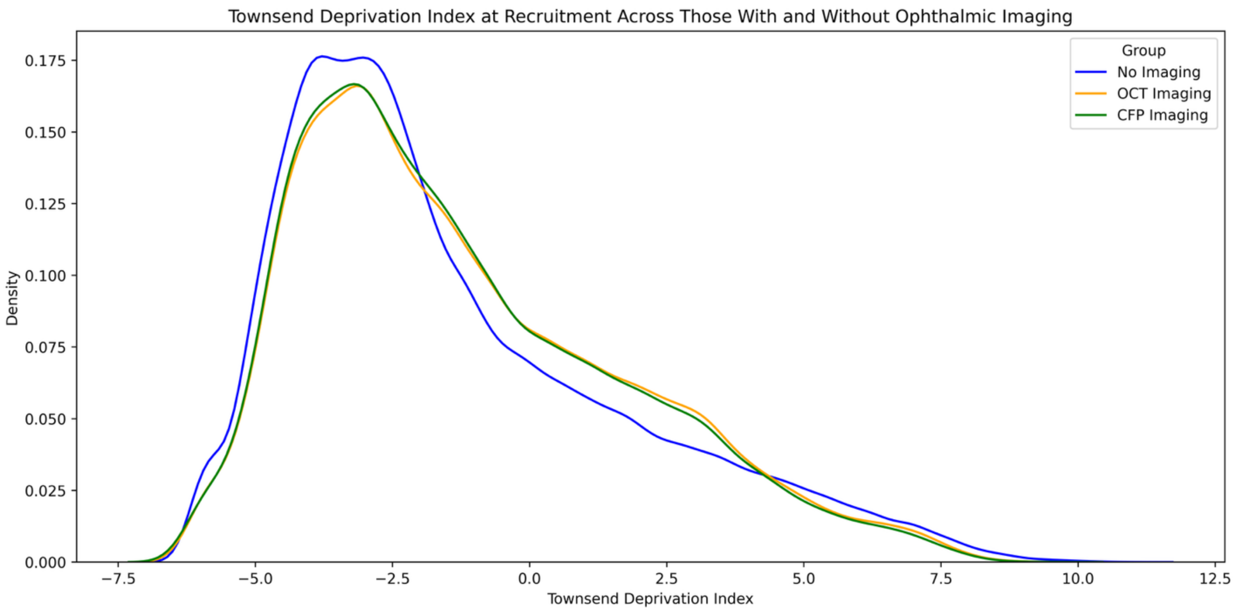

Supplementary Figure 4: A density plot showing the distribution of Townsend deprivation index of UK Biobank subjects. The plot compares those without imaging, those with sufficiently high-quality left eye colour fundus photograph (CFP) images, and those with sufficiently high-quality left eye optical coherence tomography (OCT) images. Quality criteria are described in the methods section of the manuscript.

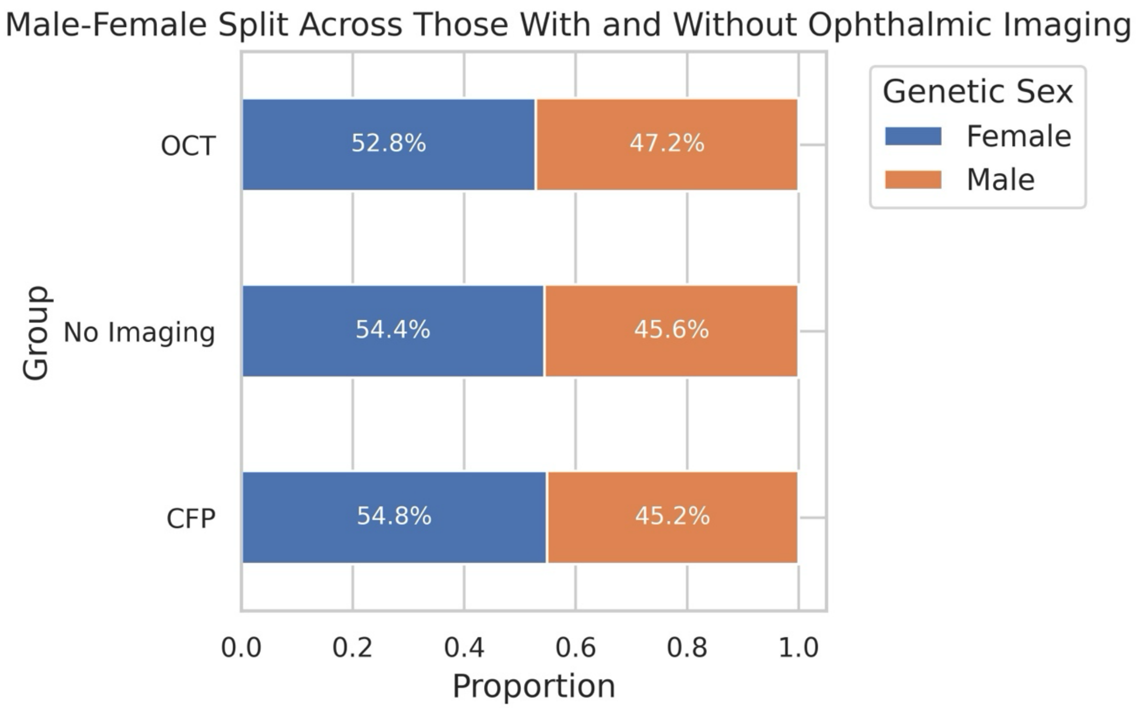

Supplementary Figure 5: A bar plot showing the male : female (genetic sex) split of UK Biobank subjects. The plot compares those without imaging, those with sufficiently high-quality left eye colour fundus photograph (CFP) images, and those with sufficiently high-quality left eye optical coherence tomography (OCT) images. Quality criteria are described in the methods section of the manuscript.

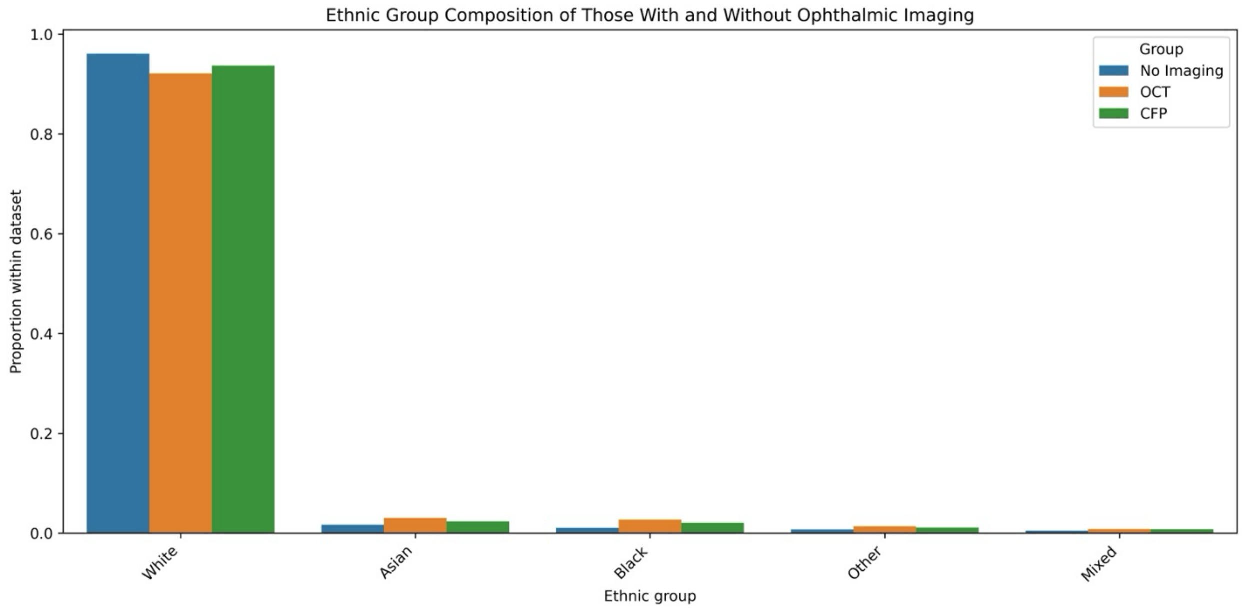

Supplementary Figure 6: A bar plot showing the self-reported ethnicity split of UK Biobank subjects. The plot compares those without imaging, those with sufficiently high-quality left eye colour fundus photograph (CFP) images, and those with sufficiently high-quality left eye optical coherence tomography (OCT) images. Quality criteria are described in the methods section of the manuscript. Ethnic groups were defined as per the UK 2021 census (with some truncation of full group names to allow them to fit on the plot axis). The ethnic groups as defined by the UK government are available here: https://www.ethnicity-facts-figures.service.gov.uk/style-guide/ethnic-groups/

Positive Control Plots

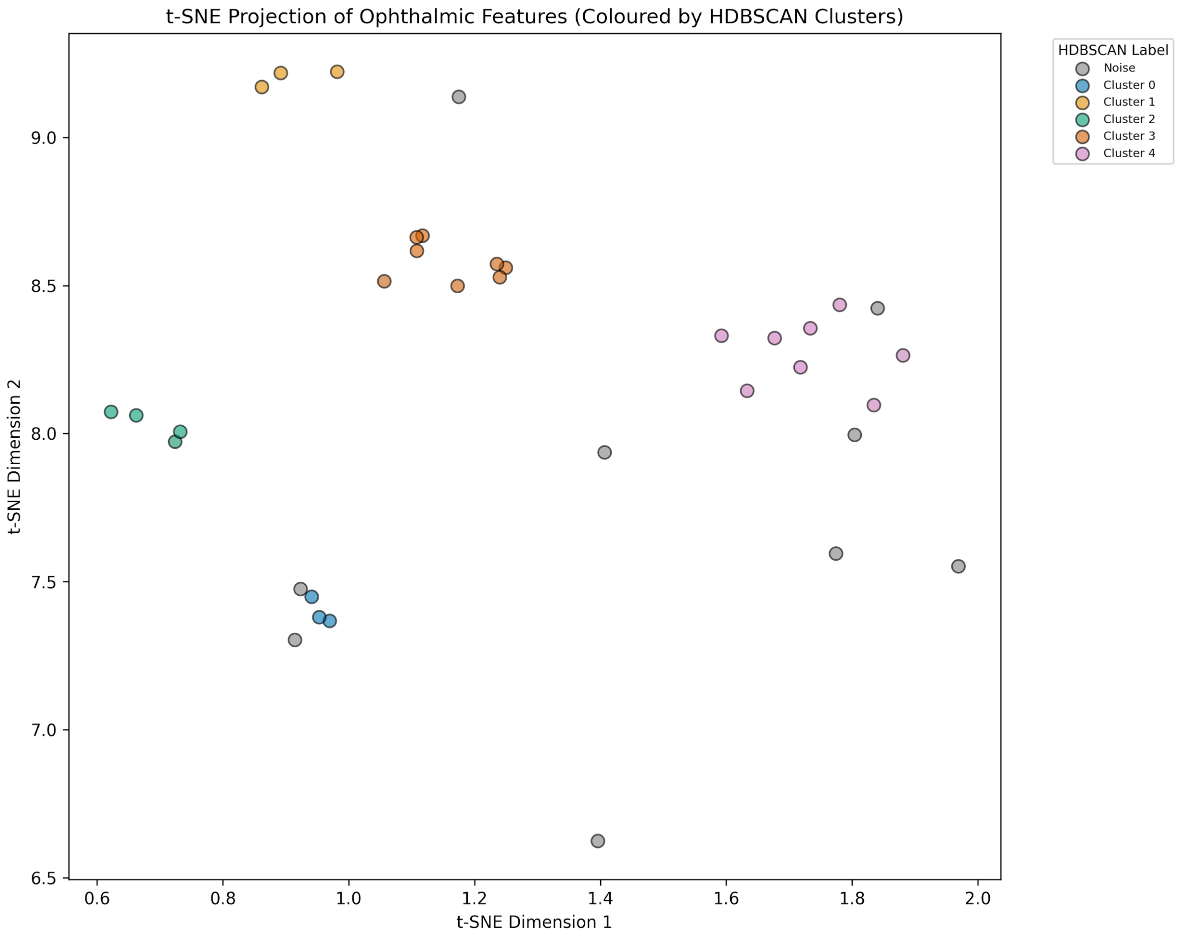

Supplementary Figure 7: A t-SNE projection of ophthalmic features clustered using HDBSCAN. The clusters have been colour coded according to the HDBSCAN cluster.

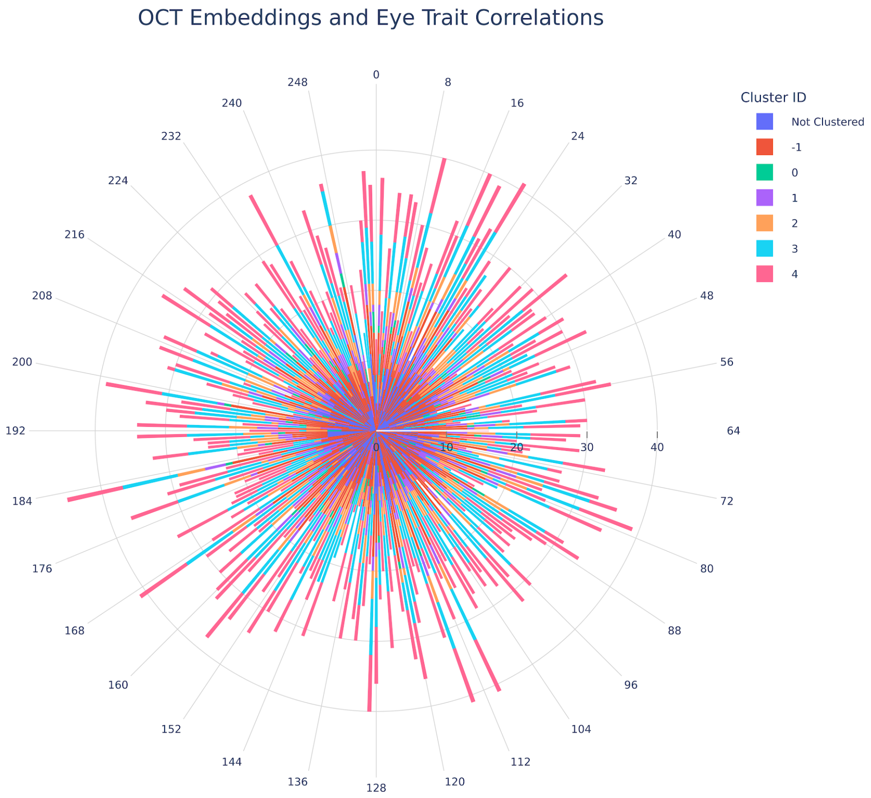

Supplementary Figure 8: A polar plot illustrating the relationships between ophthalmic traits and OCT embeddings. HDBSCAN identified four data clusters: 'cluster 0' included intraocular pressure and corneal hysteresis measures; 'cluster 1' included OCT derived photoreceptor layer thickness measures; 'cluster 2' included OCT derived thickness of the photoreceptor & retinal pigment epithelium layers; 'cluster 3' included a number of OCT derived thickness measures including the inner nuclear layer, outer plexiform layer, photoreceptors and retinal pigment epithelium; and 'cluster 4' contained OCT derived macular thickness measures. Inner retinal thickness measures (e.g. retinal nerve fibre layer and ganglion cell layer thickness). 15 traits could not be clustered due to a high level of missingness across the population. and visual acuity did not fall into HDBSCAN clusters, and were categorized as noise (cluster -1). The circumferential axis indicates the embedding number. The radial axis indicates the number of multiple testing corrected significant Pearson correlations that the embedding has with Ophthalmic traits. The bars are colour coded according to the cluster of traits the significant relationship belongs to. There were more significant relationships between OCT embeddings and Ophthalmic traits than CFP embeddings and Ophthalmic traits. This is expected given that most UKB ocular traits were derived from OCT scans.

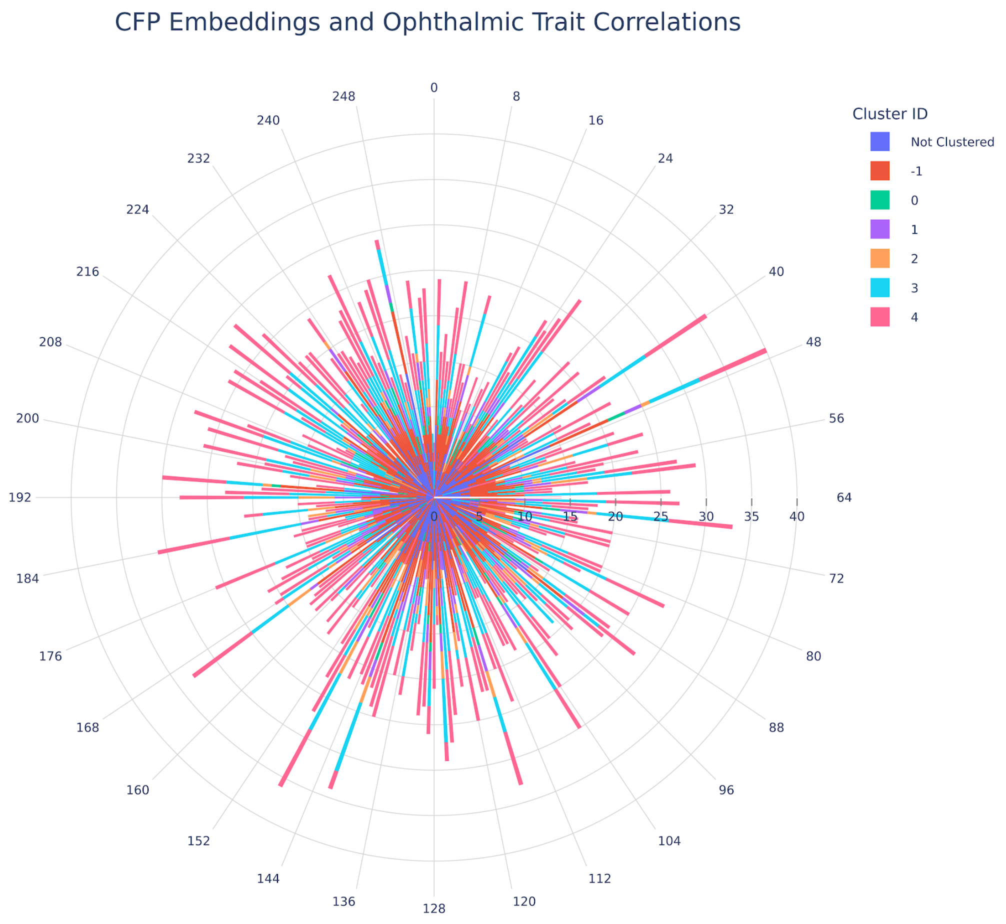

Supplementary Figure 9: A polar plot illustrating the relationships between ophthalmic traits and CFP embeddings. HDBSCAN identified four data clusters: ‘cluster 0’ included intraocular pressure and corneal hysteresis measures; ‘cluster 1’ included OCT derived photoreceptor layer thickness measures; ‘cluster 2’ included OCT derived thickness of the photoreceptor & retinal pigment epithelium layers; ‘cluster 3’ included a number of OCT derived thickness measures including the inner nuclear layer, outer plexiform layer, photoreceptors and retinal pigment epithelium; and ‘cluster 4’ contained OCT derived macular thickness measures. Inner retinal thickness measures (e.g. retinal nerve fibre layer and ganglion cell layer thickness). 15 traits could not be clustered due to a high level of missingness across the population. and visual acuity did not fall into HDBSCAN clusters, and were categorized as noise (cluster -1). The circumferential axis indicates the embedding number. The radial axis indicates the number of multiple testing corrected significant Pearson correlations that the embedding has with Ophthalmic traits. The bars are colour coded according to the cluster of traits the significant relationship belongs to.

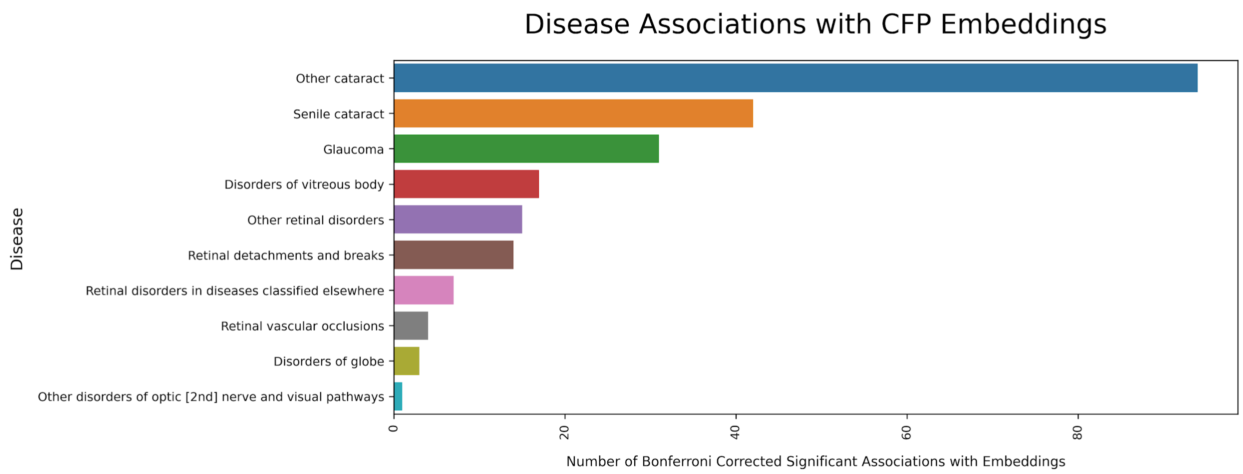

Supplementary Figure 10: The results of Welch's t-test. This bar plot shows the total number of multiple testing corrected significant associations between ocular diseases and CFP embedding values. As can be seen, all ophthalmic disorders tested demonstrated associations with embeddings values. The fewest multiple testing significant results were seen for non-glaucomatous optic nerve disorders, an expected result given the retrobulbar nature of these conditions and the absence of dedicated optic nerve imaging in this study.

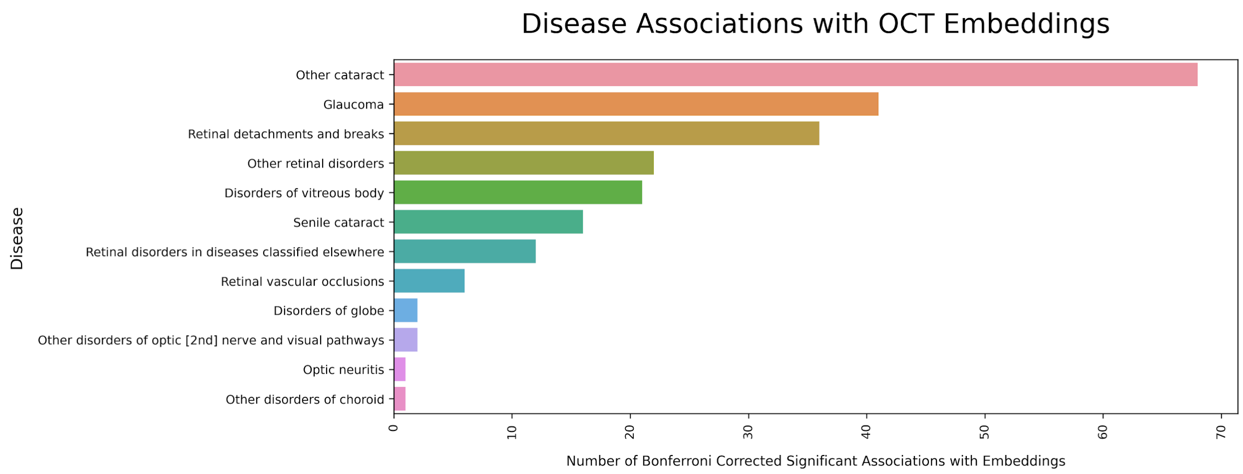

Supplementary Figure 11: The results of Welch's t-test. This bar plot shows the total number of multiple testing corrected significant associations between ocular diseases and OCT embedding values. As can be seen, all ophthalmic disorders tested demonstrated associations with embeddings values. The fewest multiple testing significant results were seen for non-glaucomatous optic nerve disorders, an expected result given the retrobulbar nature of these conditions and the absence of dedicated optic nerve imaging in this study.

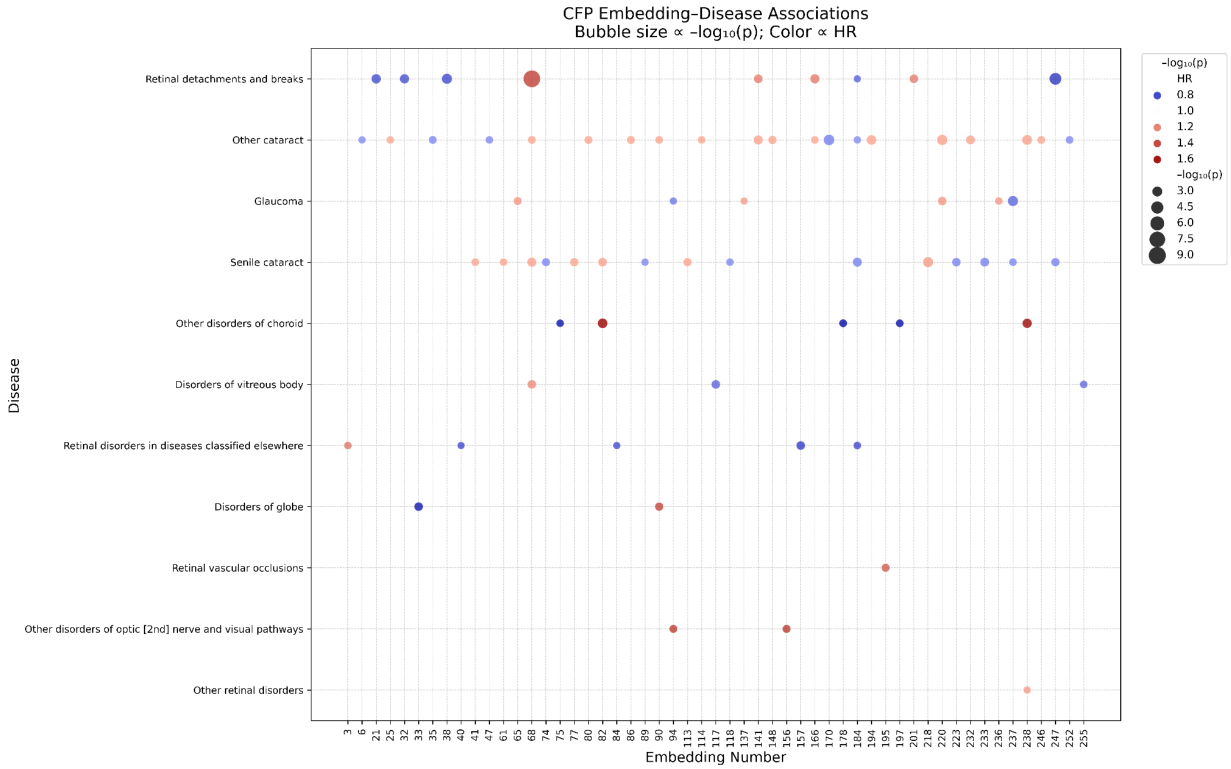

Supplementary Figure 12: A bubble plot illustrating the results of our CFP-ocular disease Cox Proportional Hazards analysis. Only embeddings and disorders with at least one multiple testing corrected significant result are plotted here. The size of the bubbles indicates the level of significance (larger bubbles = more significant). The bubbles are colour coded according to the hazards ratio (HR).

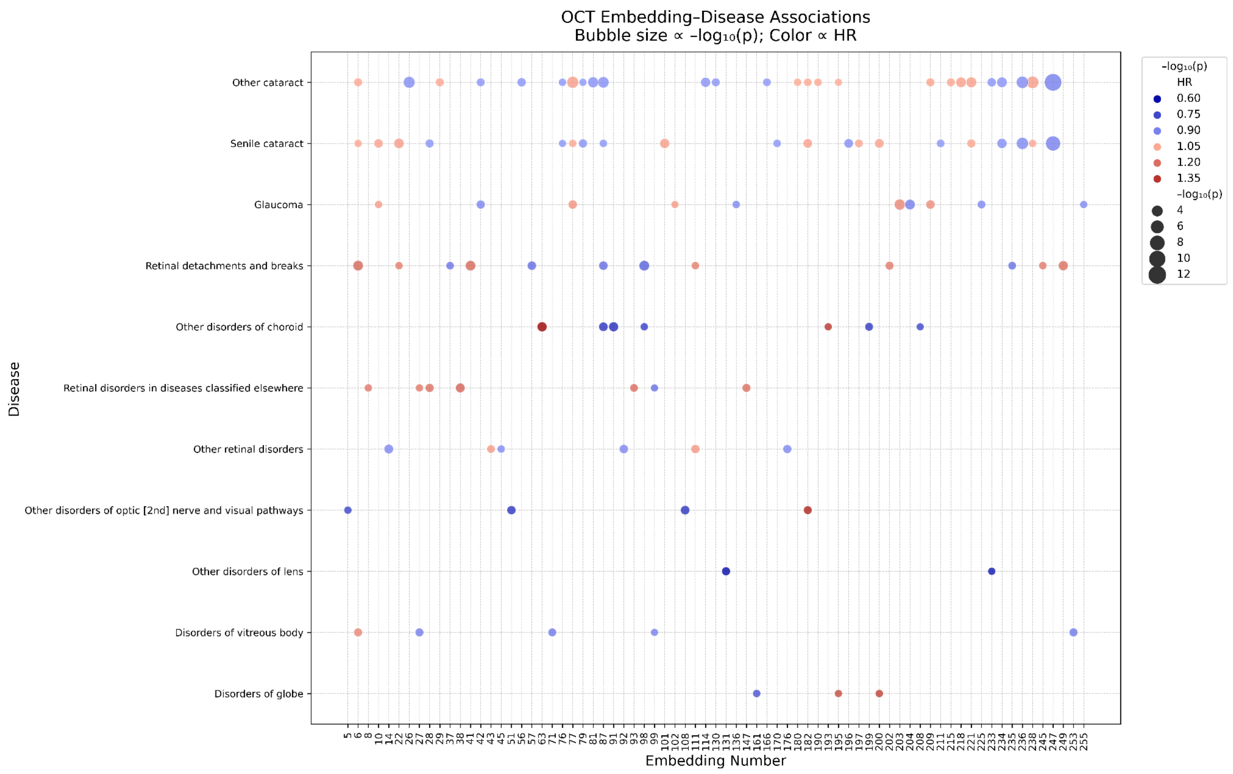

Supplementary Figure 13: A bubble plot illustrating the results of our OCT-ocular disease Cox Proportional Hazards analysis. Only embeddings and disorders with at least one multiple testing corrected significant result are plotted here. The size of the bubbles indicates the level of significance (larger bubbles = more significant). The bubbles are colour coded according to the hazards ratio (HR).

Embedding Feature Visualisations / Saliency Mapping

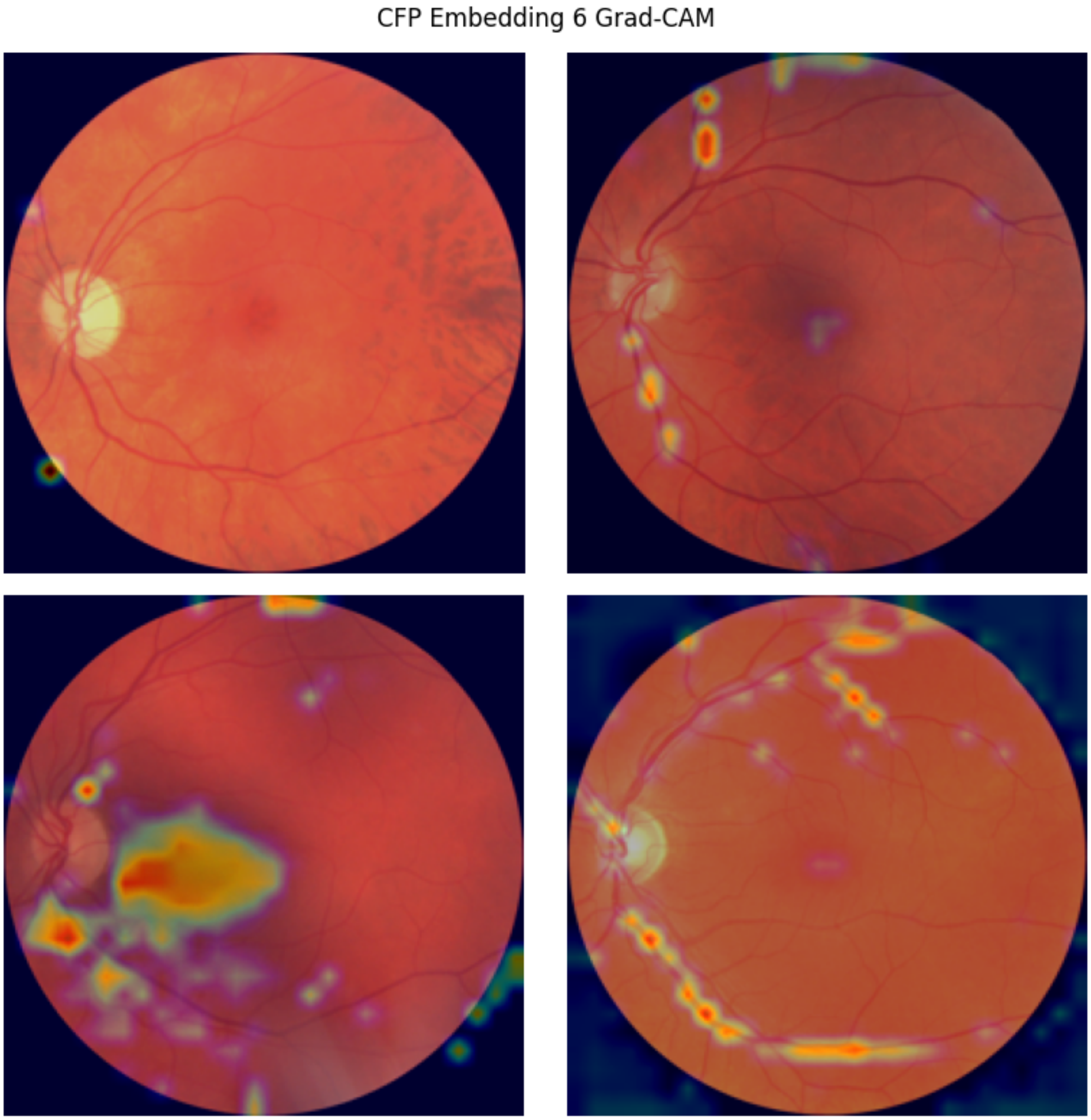

Supplementary Figure 14: Grad-CAM saliency map for CFP embedding 6. This was the second most strongly associated embedding with future hypertension risk. The embedding seems to localise to the vasculature, fovea, and projection artifacts. Reproduced with the permission of UK Biobank.

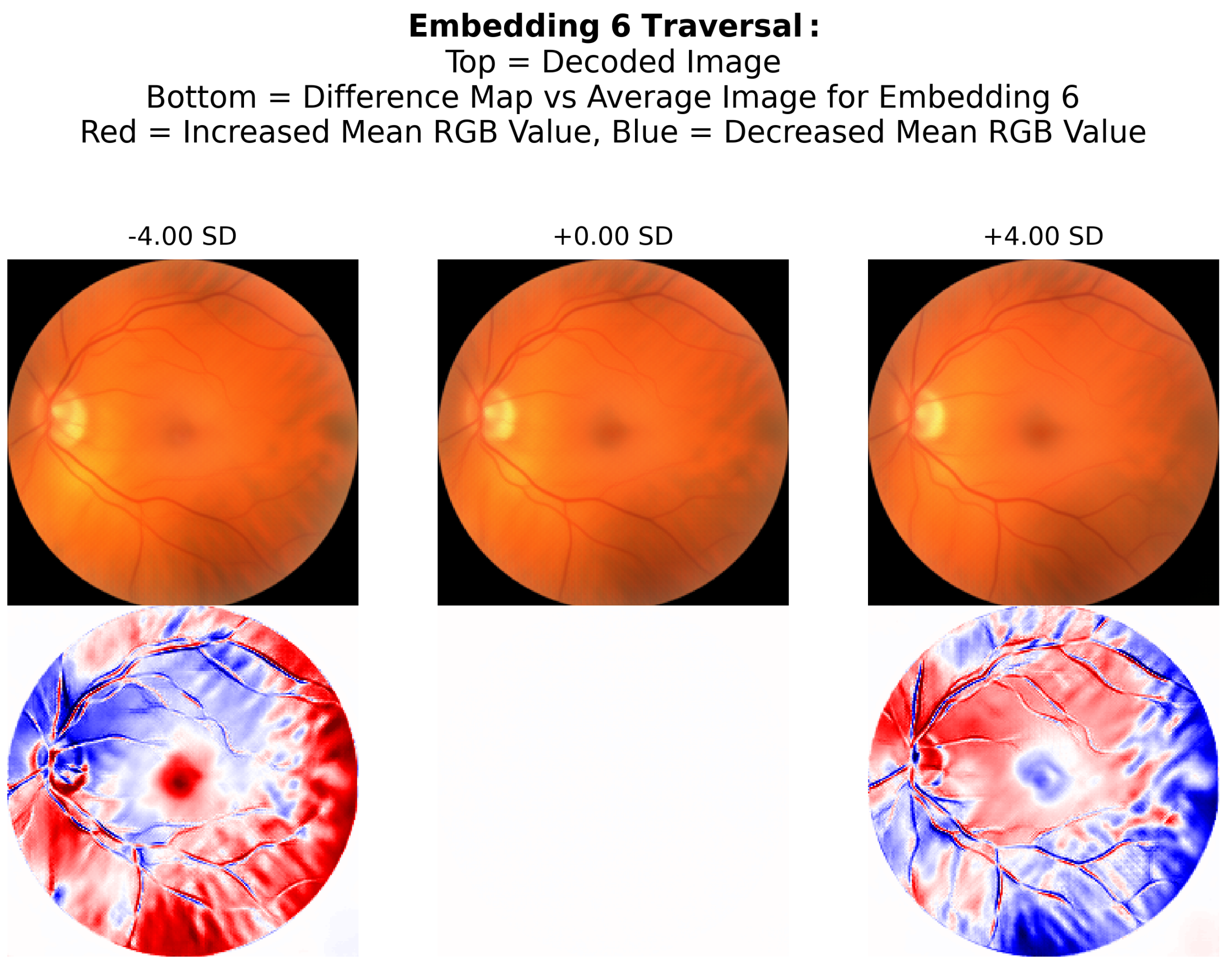

Supplementary Figure 15: Top row - reconstructions obtained by perturbing latent dimension 6 by ±4 standard deviations (SD) around its mean, while holding all other latent dimensions constant. This was the second most strongly associated embedding with future hypertension risk. The central image corresponds to the reference latent vector. Bottom row - difference maps showing the mean per-pixel change in RGB intensity relative to the reference reconstruction. Red denotes increased mean RGB intensity, and blue denotes decreased intensity.

Perturbation of this latent dimension primarily modulates the spatial extent and intensity of darker regions in the CFP, including the foveal region (which expands progressively from −4 SD to +4 SD) and peripheral choroidal patterns. This suggests that embedding dimension 6 captures variation related to global and regional image darkness/illuminance rather than a single anatomically localised feature. Reproduced with the permission of UK Biobank.

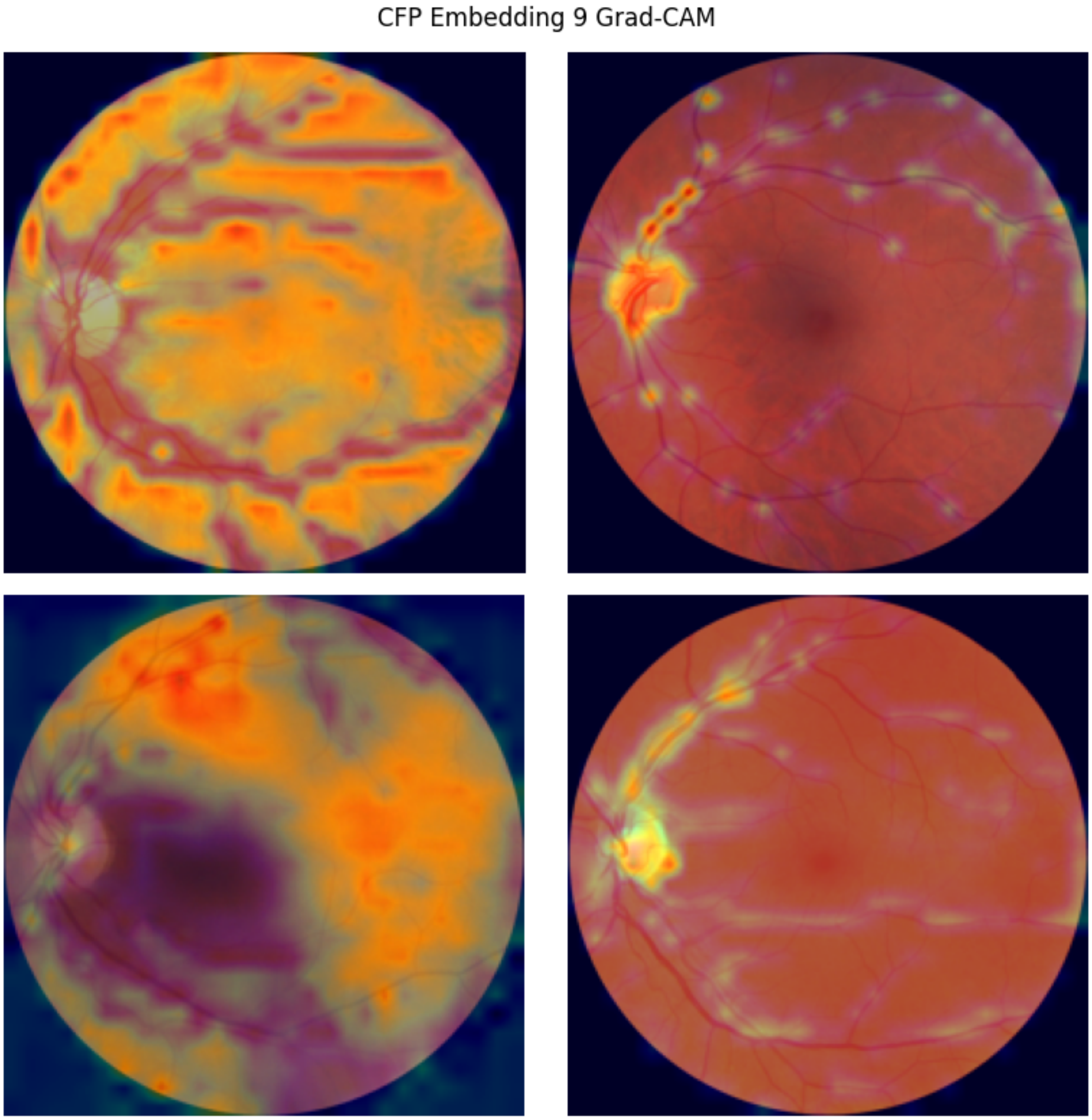

Supplementary Figure 16: Grad-CAM saliency map for CFP embedding 9. This was the embedding most strongly associated with heart failure at the time of imaging (baseline). The embedding seems to represent the vasculature, and perhaps to some extent the fundal pigmentation or choroidal features (given the background feature saliency). Reproduced with the permission of UK Biobank.

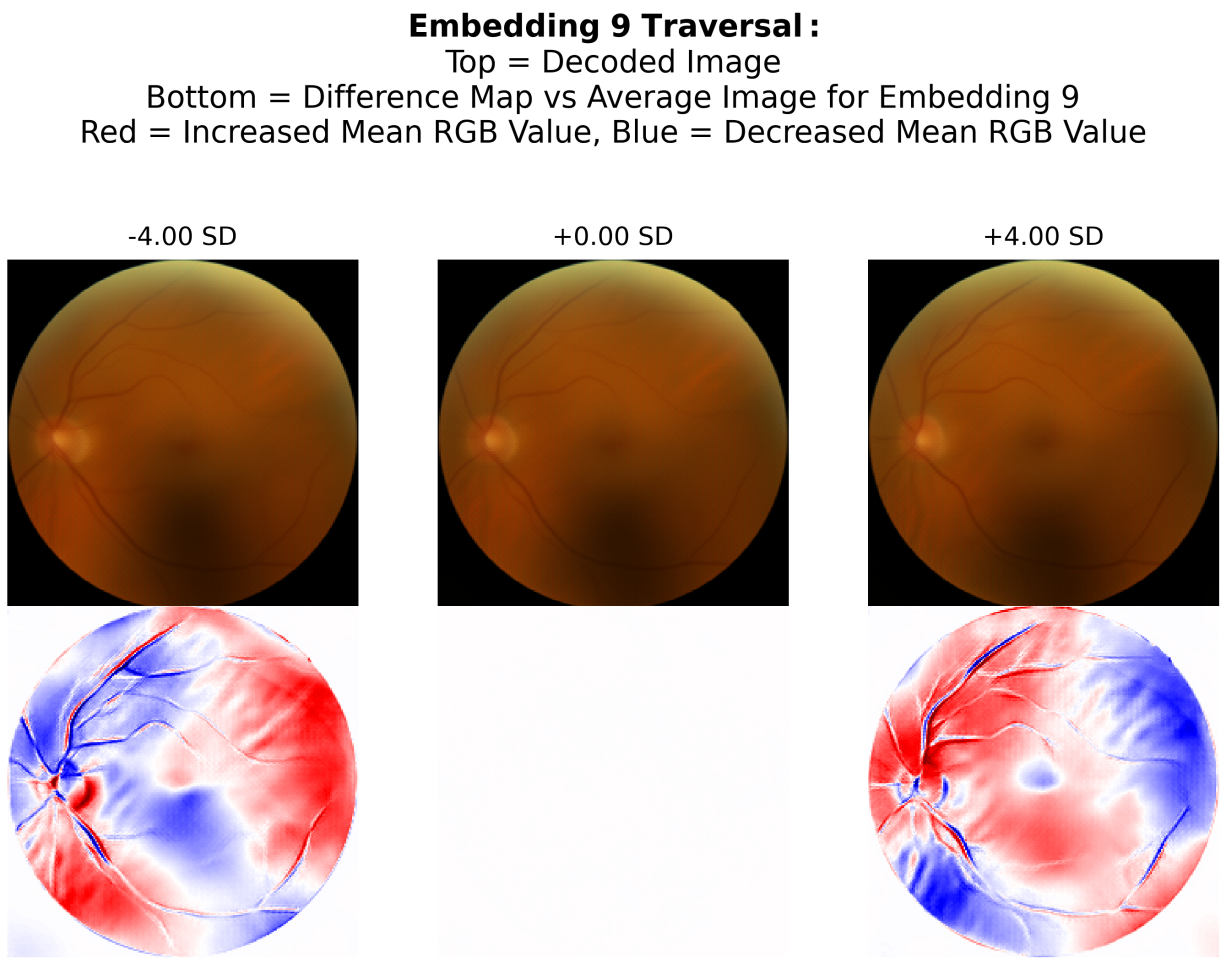

Supplementary Figure 17: Top row - reconstructions obtained by perturbing latent dimension 9 by ±4 standard deviations (SD) around its mean, while holding all other latent dimensions constant. This was the embedding most strongly associated with heart failure at the time of imaging (baseline). The central image corresponds to the reference latent vector. Bottom row - difference maps showing the mean per-pixel change in RGB intensity relative to the reference reconstruction. Red denotes increased mean RGB intensity, and blue denotes decreased intensity.

Perturbation of this latent dimension appears to diffusely affect overall RGB values across the CFP. Reproduced with the permission of UK Biobank.

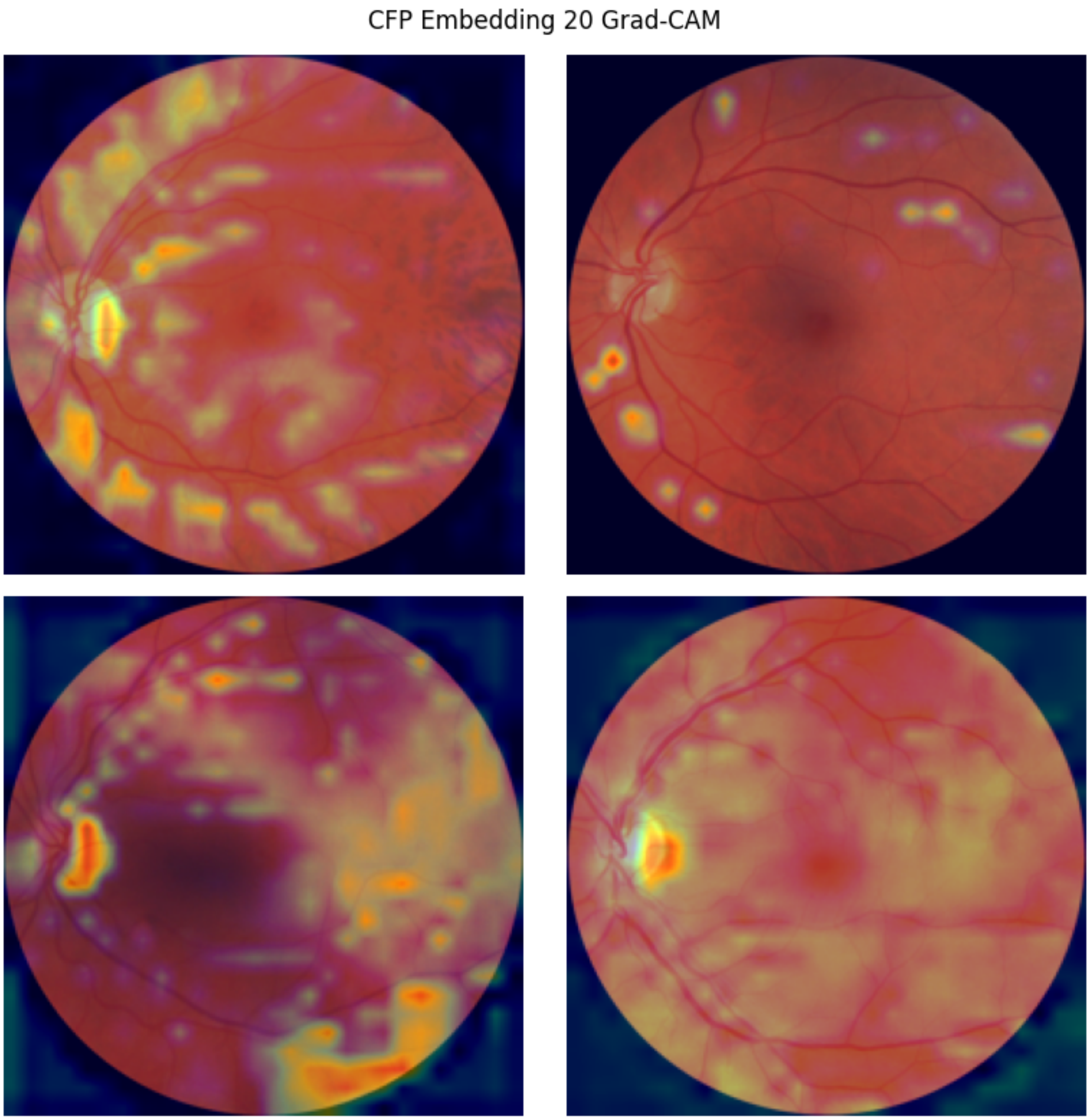

Supplementary Figure 18: Grad-CAM saliency map for CFP embedding 20. This was the embedding most strongly associated with chronic ischaemic heart disease at the time of imaging (baseline). The embedding seems to represent the optic nerve head, as well as some scattered background features, which we speculate could be the choroidal vasculature. Reproduced with the permission of UK Biobank.

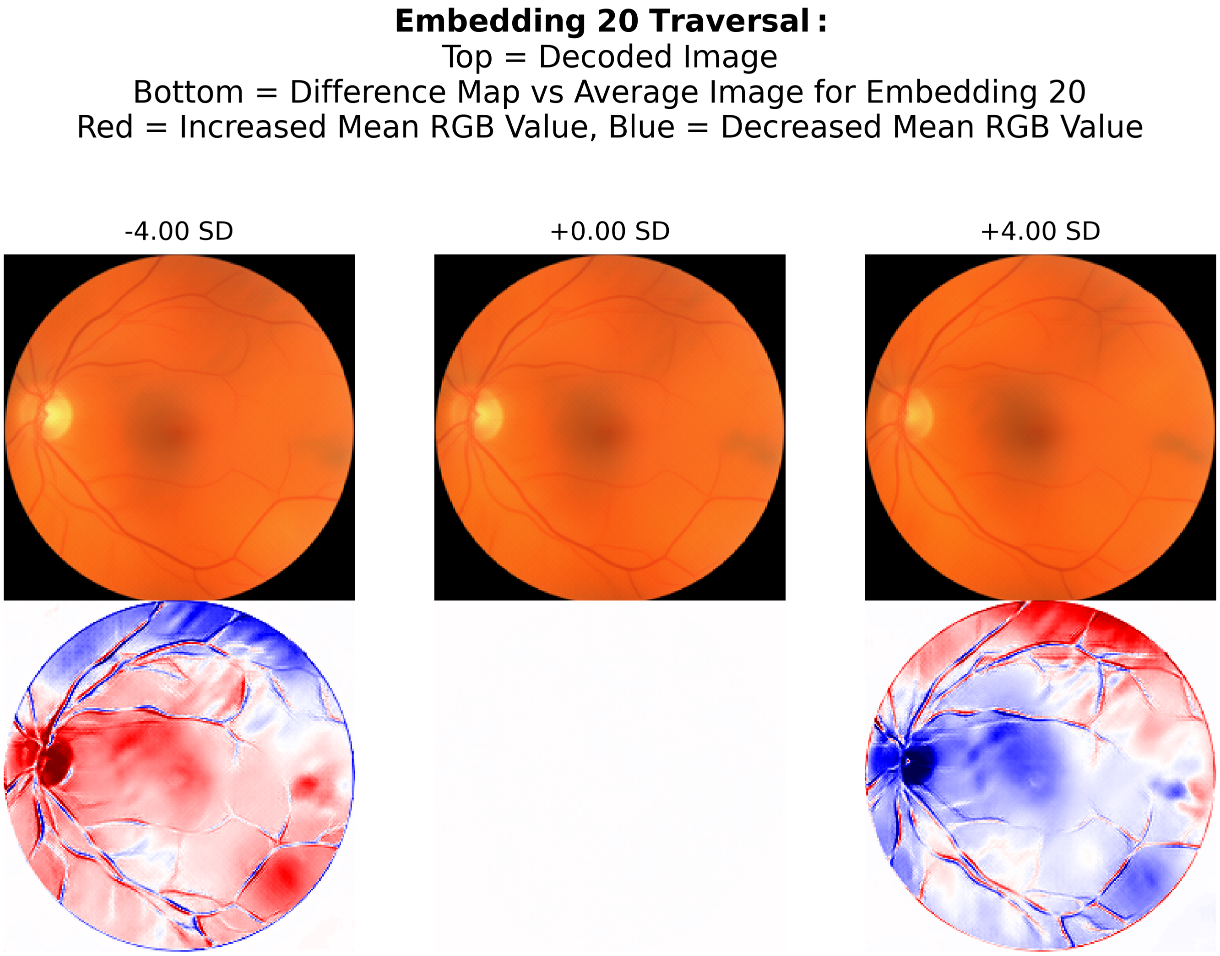

Supplementary Figure 19: Top row - reconstructions obtained by perturbing latent dimension 20 by ±4 standard deviations (SD) around its mean, while holding all other latent dimensions constant. This was the embedding most strongly associated with chronic ischaemic heart disease at the time of imaging (baseline). The central image corresponds to the reference latent vector. Bottom row - difference maps showing the mean per-pixel change in RGB intensity relative to the reference reconstruction. Red denotes increased mean RGB intensity, and blue denotes decreased intensity.

Perturbation of this latent dimension appears to modulate the appearance of the optic nerve head. The cup-disc ratio reduces from -4.00 to +4.00 SD from the mean. Reproduced with the permission of UK Biobank.

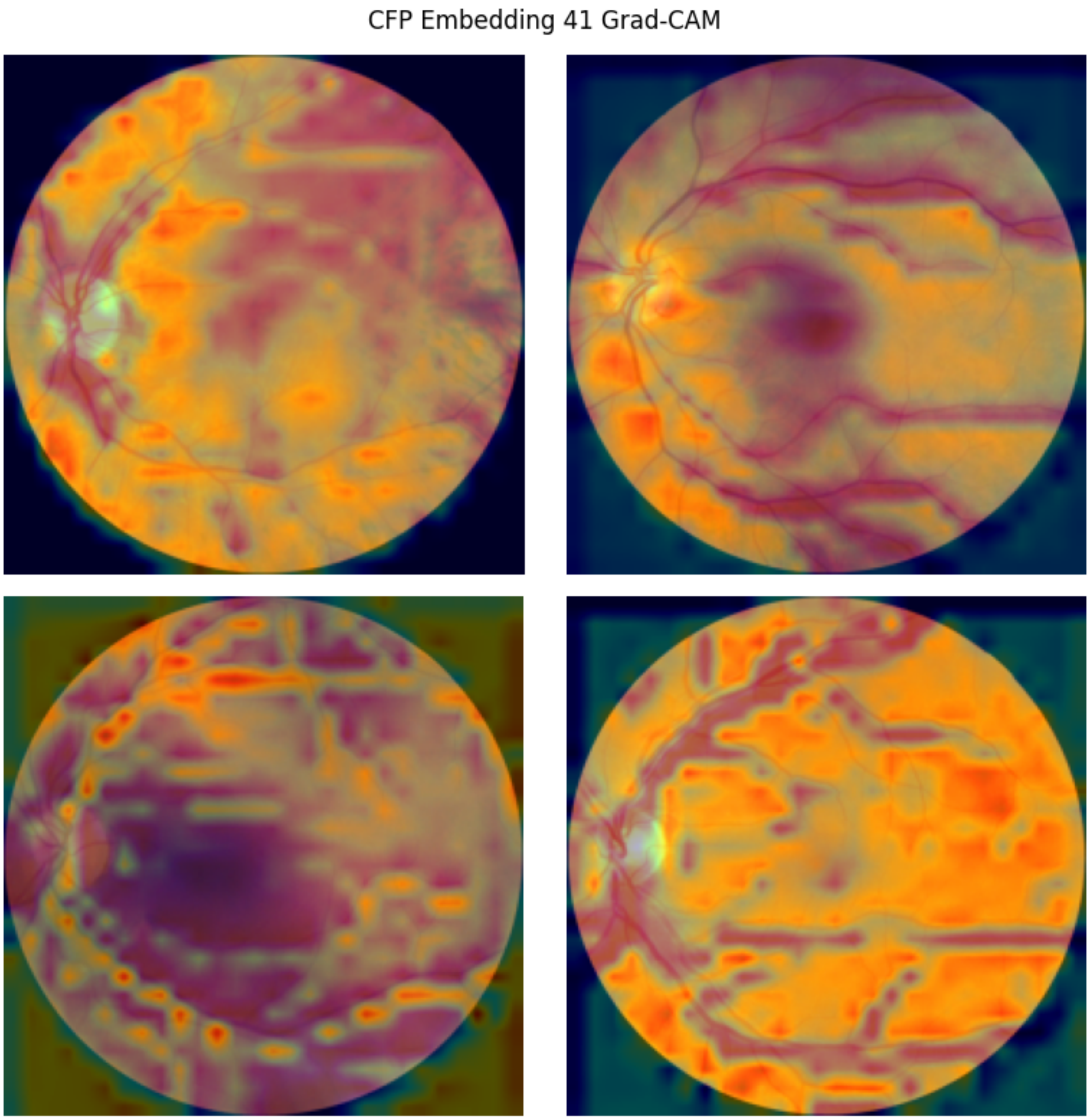

Supplementary Figure 20: Grad-CAM saliency map for CFP embedding 41. This was the CFP feature most strongly associated with the gene set ‘WikiPathways kynurenine pathway and links to cell senescence’. As can be seen, the embedding has a diffuse saliency map, and it is not clear what specific trait is represented by the latent feature. Whilst some maps correspond to the vasculature (bottom left), others correspond to background fundal features. Reproduced with the permission of UK Biobank.

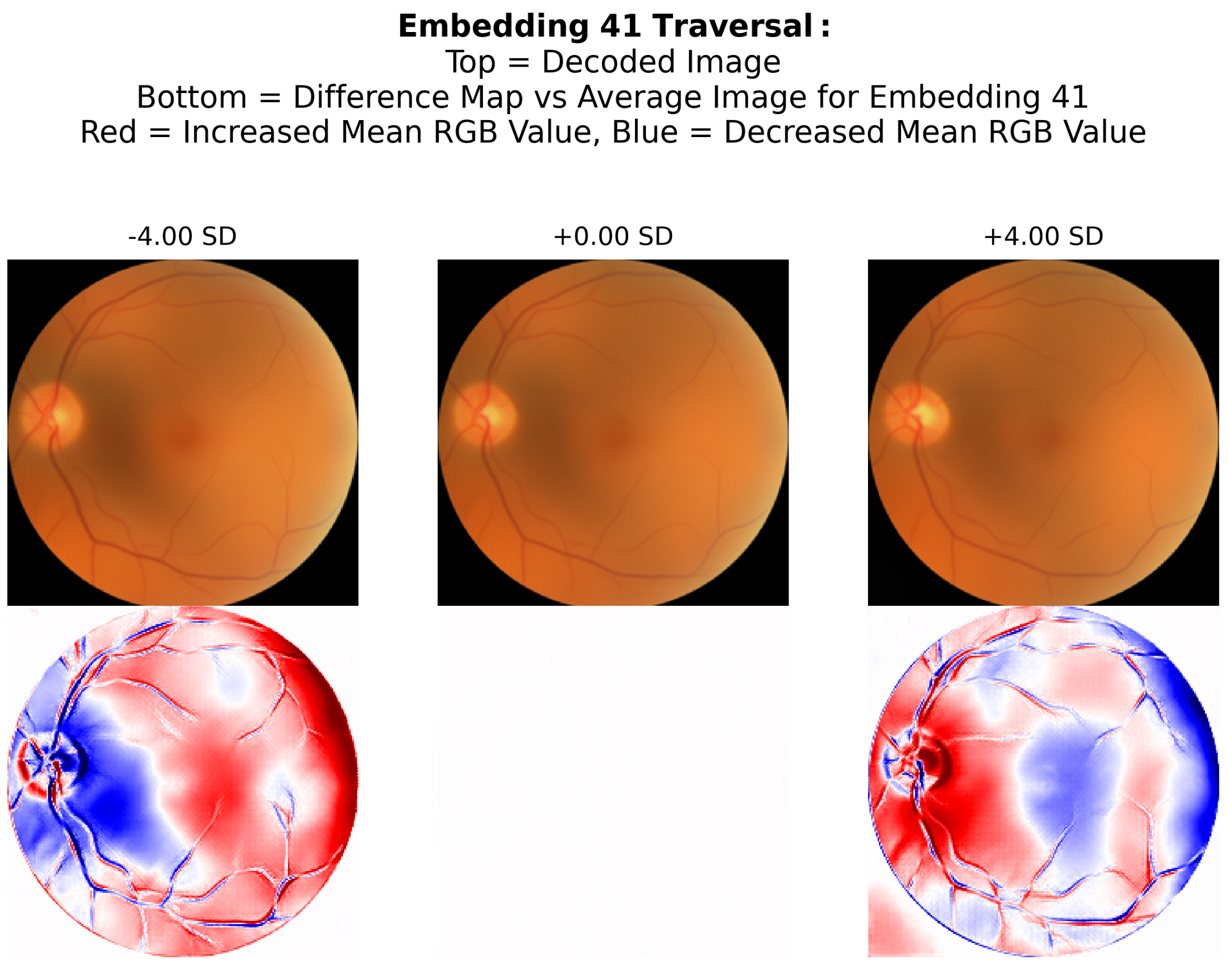

Supplementary Figure 21: Top row - reconstructions obtained by perturbing latent dimension 41 by ±4 standard deviations (SD) around its mean, while holding all other latent dimensions constant. This was the CFP feature most strongly associated with the gene set 'WikiPathways kynurenine pathway and links to cell senescence'. The central image corresponds to the reference latent vector. Bottom row - difference maps showing the mean per-pixel change in RGB intensity relative to the reference reconstruction. Red denotes increased mean RGB intensity, and blue denotes decreased intensity.

Perturbation of this latent dimension appears to modulate the appearance of the inferior vascular arcades and optic nerve head appearance (note the cup:disc ratio) but also has a more diffuse impact on the fundal illuminance. Reproduced with the permission of UK Biobank.

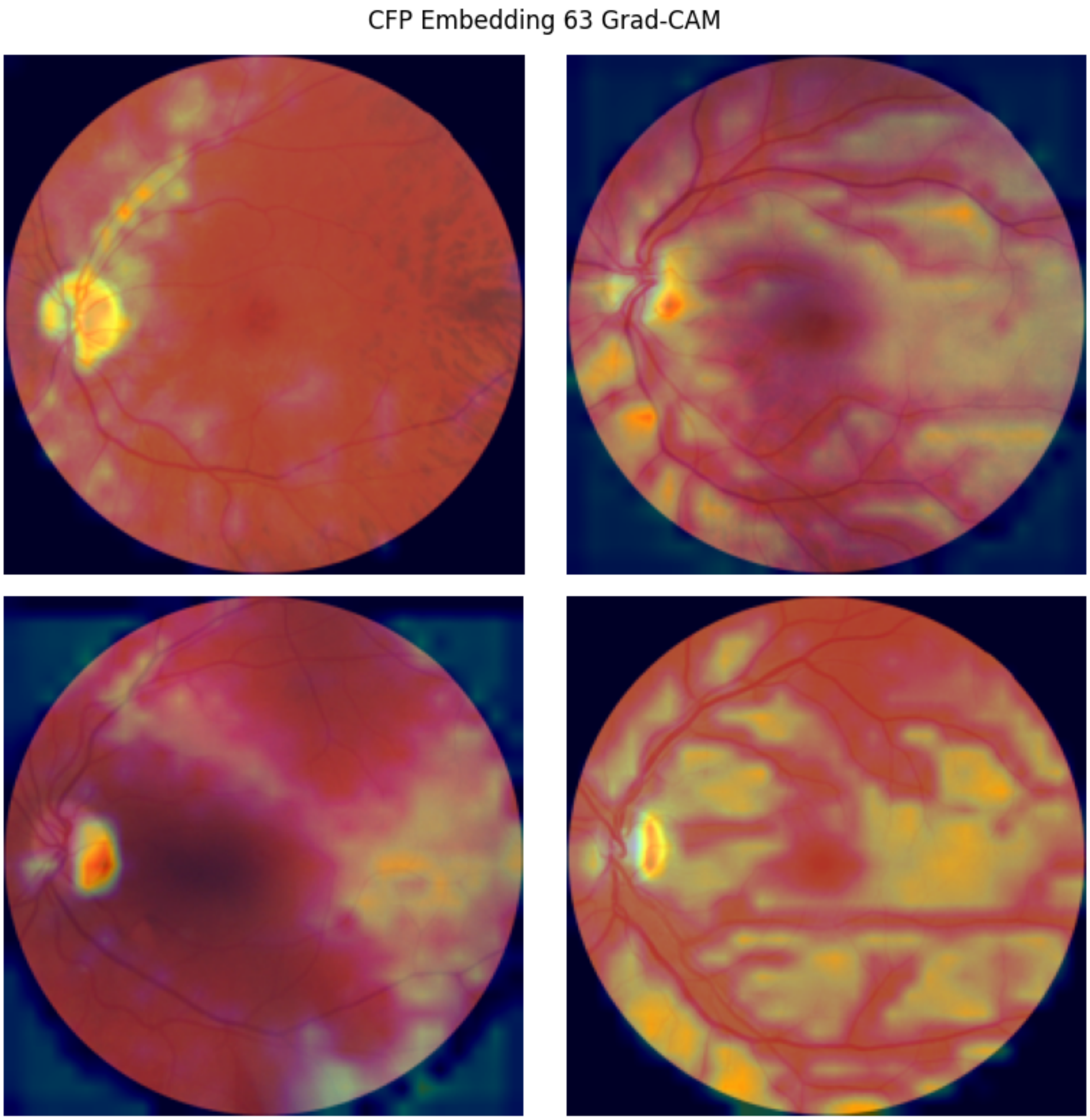

Supplementary Figure 22: Grad-CAM saliency map for CFP embedding 63. This was the embedding most strongly associated with acute myocardial infarction at the time of imaging (baseline). As can be seen, the embedding seems to localise to the optic nerve head, with some less consistent signals around the vasculature and more diffusely throughout the background. Reproduced with the permission of UK Biobank.

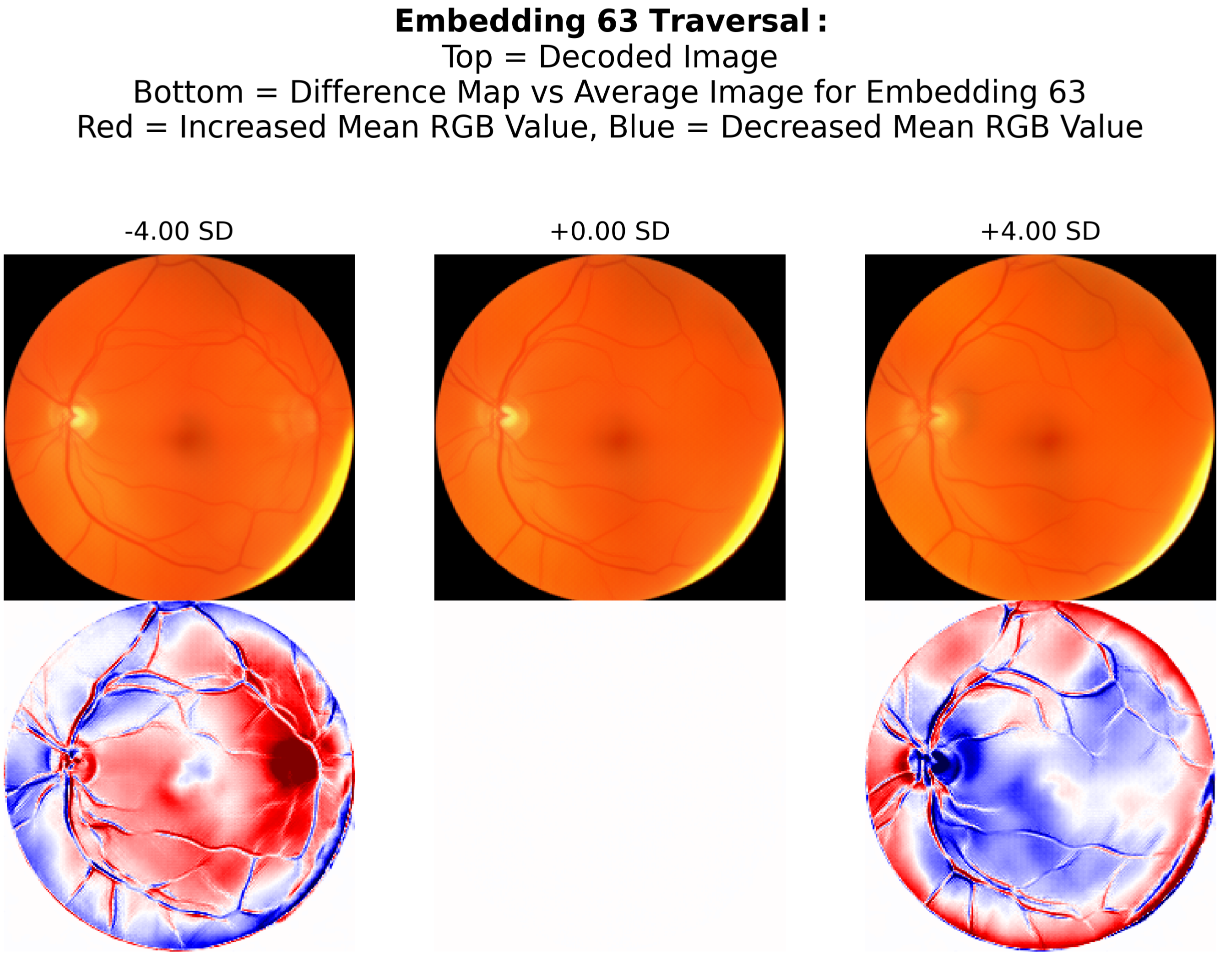

Supplementary Figure 23: Top row - reconstructions obtained by perturbing latent dimension 63 by ±4 standard deviations (SD) around its mean, while holding all other latent dimensions constant. This was the embedding most strongly associated with acute myocardial infarction at the time of imaging (baseline). The central image corresponds to the reference latent vector. Bottom row - difference maps showing the mean per-pixel change in RGB intensity relative to the reference reconstruction. Red denotes increased mean RGB intensity, and blue denotes decreased intensity.

Perturbation of this latent dimension appears to modulate the appearance of the optic nerve head. The -4.00SD image appears to fall outside of the image manifold, with the decoded image having an implausible anatomy. The 0.00SD - +4.00SD images however illustrate that the perturbation affects the colour of the optic nerve head. Reproduced with the permission of UK Biobank.

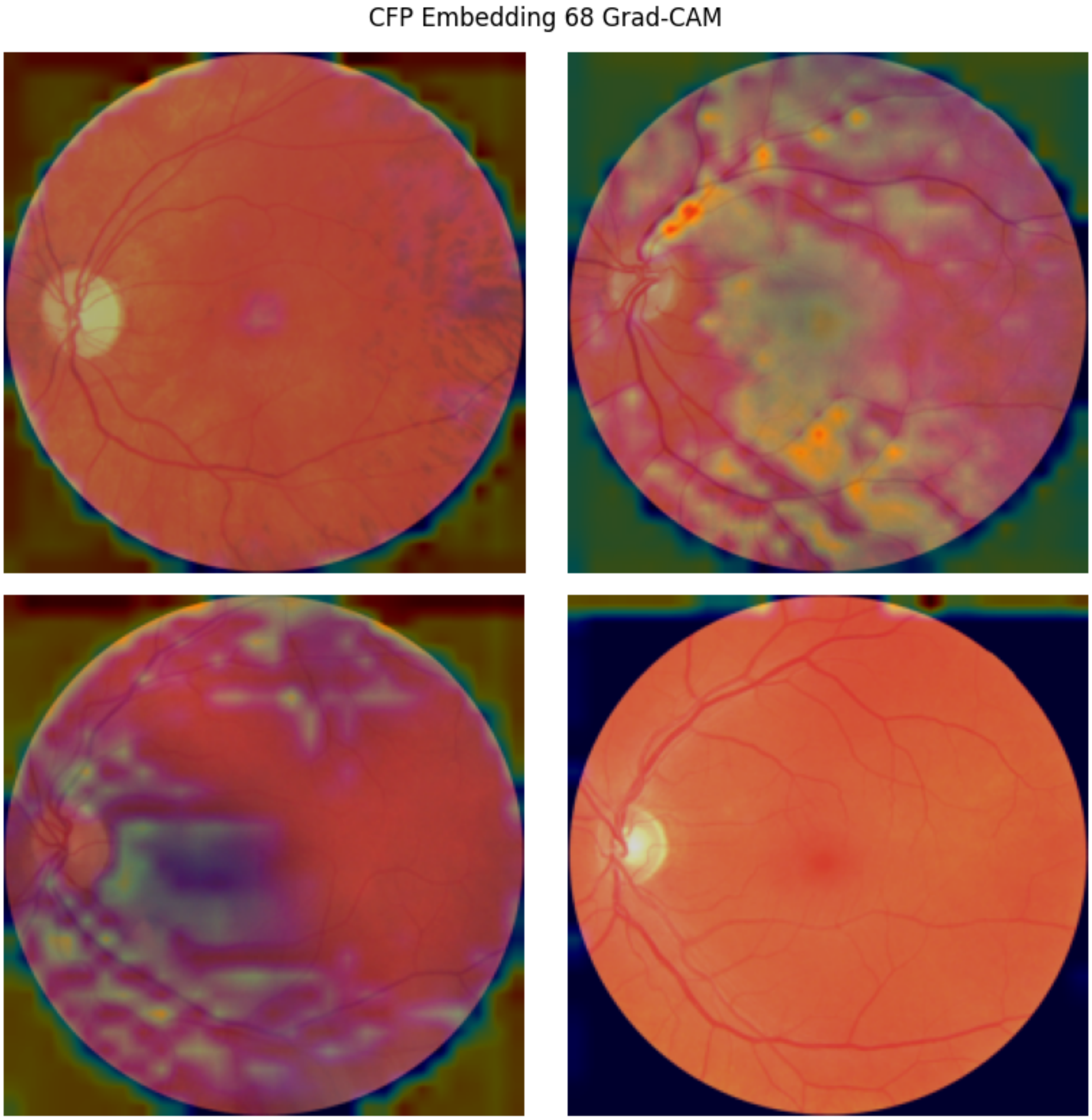

Supplementary Figure 24: Grad-CAM saliency map for CFP embedding 68. This embedding was genetically correlated with heart failure (nominally). The feature appears to localise to the vasculature. Reproduced with the permission of UK Biobank.

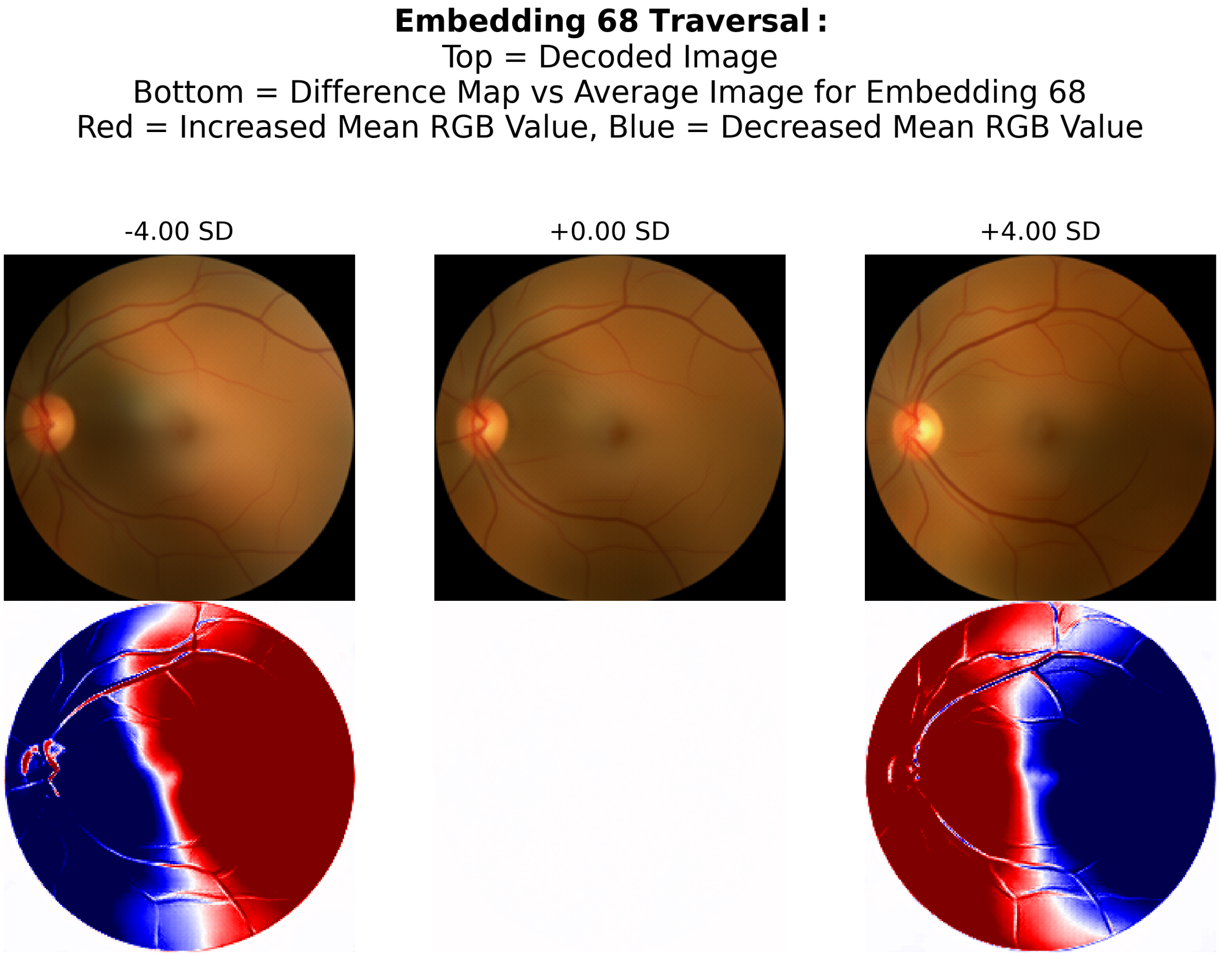

Supplementary Figure 25: Top row - reconstructions obtained by perturbing latent dimension 68 by ±4 standard deviations (SD) around its mean, while holding all other latent dimensions constant. This embedding was genetically correlated with heart failure (nominally). The central image corresponds to the reference latent vector. Bottom row - difference maps showing the mean per-pixel change in RGB intensity relative to the reference reconstruction. Red denotes increased mean RGB intensity, and blue denotes decreased intensity.

Perturbation of this latent dimension appears to modulate the appearance of the vasculature – this is seen most obviously when comparing the simple branching complexity of image -4.00 compared against the more complex branching patterns of the +4.00 image. Additionally, the embedding appears to represent shadows/dark regions of the CFP, with the most luminous aspects of the image inverting from -4.00SD - +4.00SD. Reproduced with the permission of UK Biobank.

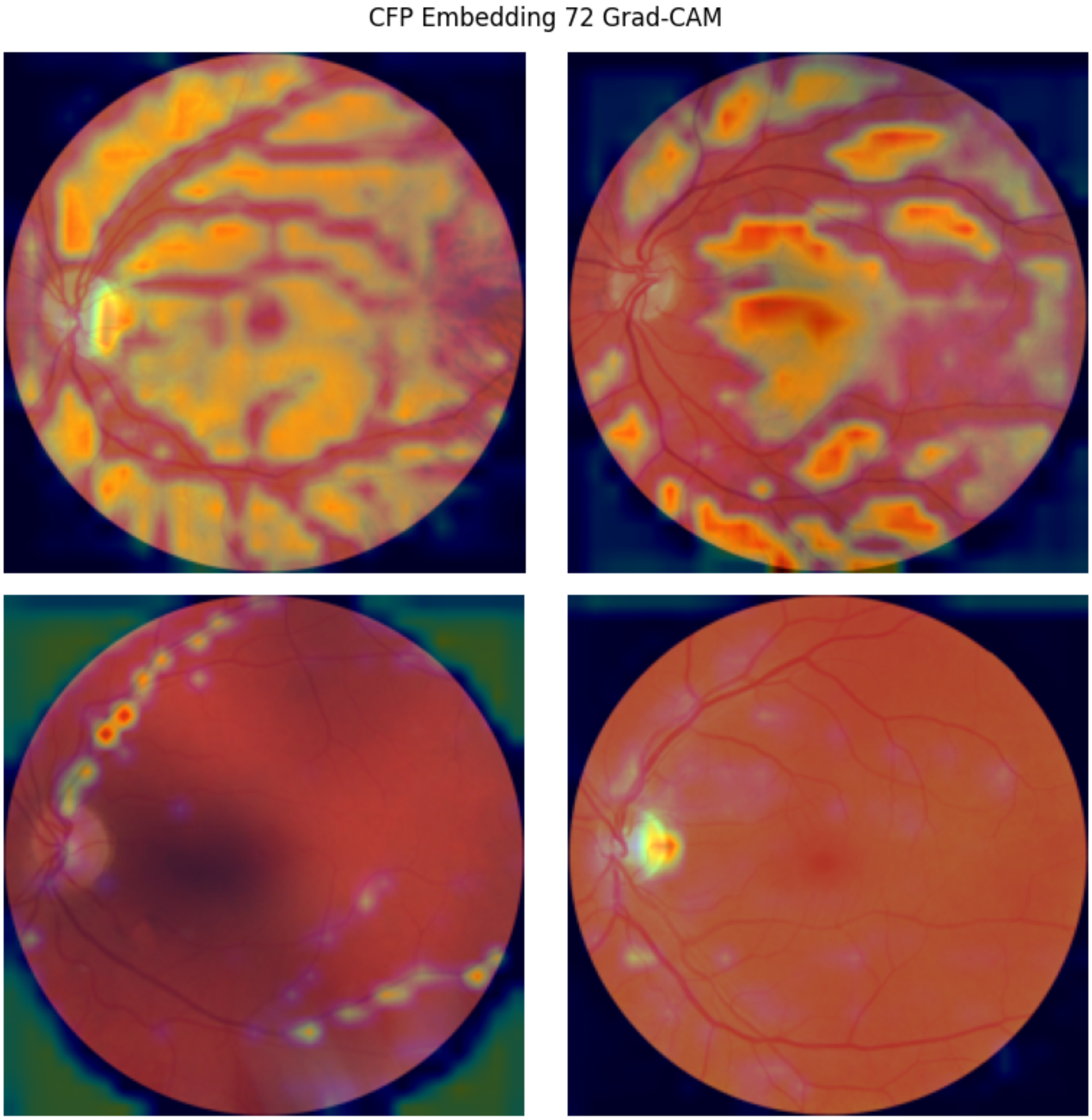

Supplementary Figure 26: Grad-CAM saliency map for CFP embedding 72. This embedding was genetically correlated with myocardial infarction (nominally). The feature appears to localise to the vasculature and the optic nerve head. Reproduced with the permission of UK Biobank.

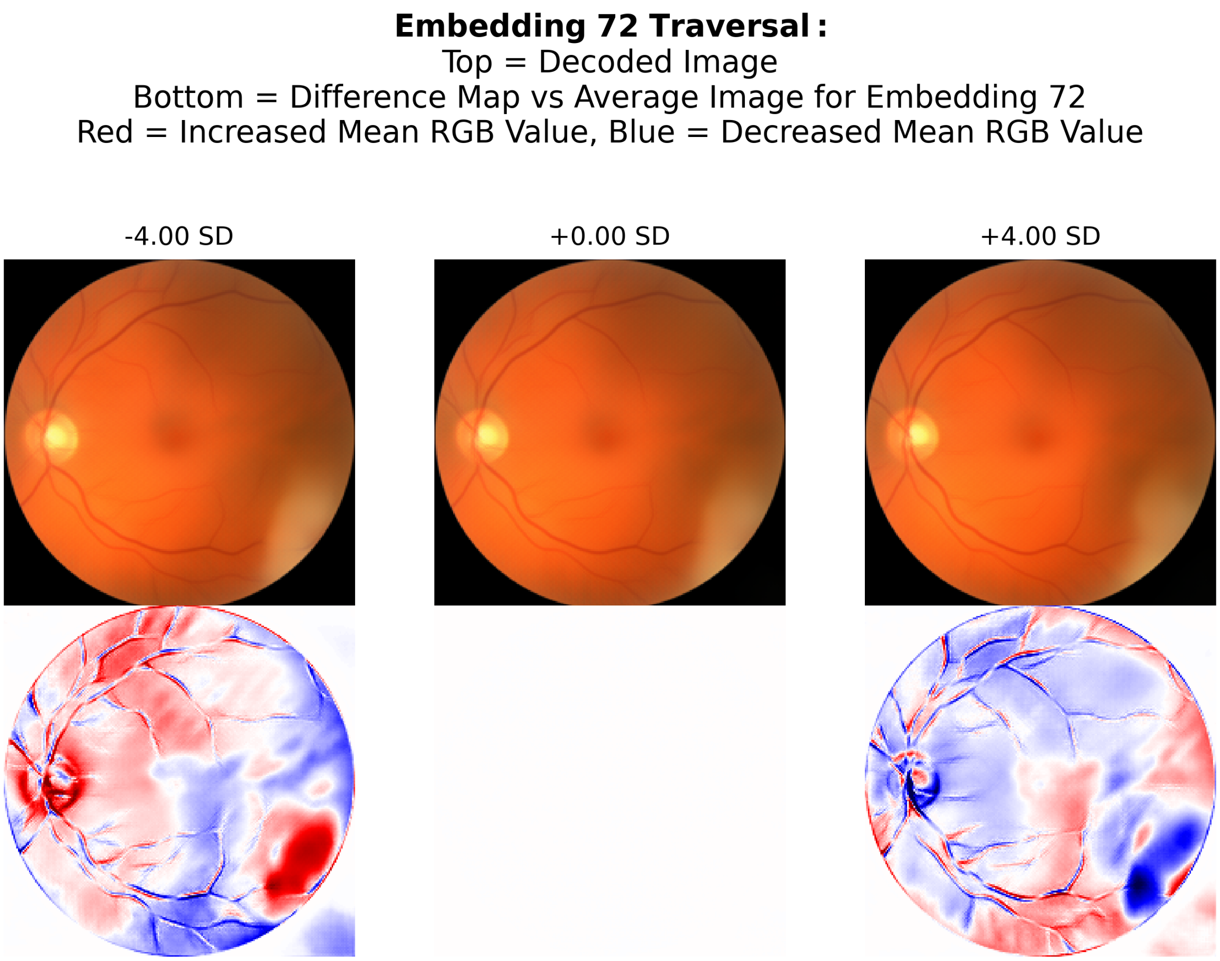

Supplementary Figure 27: Top row - reconstructions obtained by perturbing latent dimension 72 by ±4 standard deviations (SD) around its mean, while holding all other latent dimensions constant. This embedding was genetically correlated with myocardial infarction (nominally). The central image corresponds to the reference latent vector. Bottom row - difference maps showing the mean per-pixel change in RGB intensity relative to the reference reconstruction. Red denotes increased mean RGB intensity, and blue denotes decreased intensity.

Whilst there are changes to the vascular branching patterns across the perturbation, the difference maps suggest that the largest RGB pixel value changes are in the peripapillary region and in the infero-temporal region of relatively pale retina. Reproduced with the permission of UK Biobank.

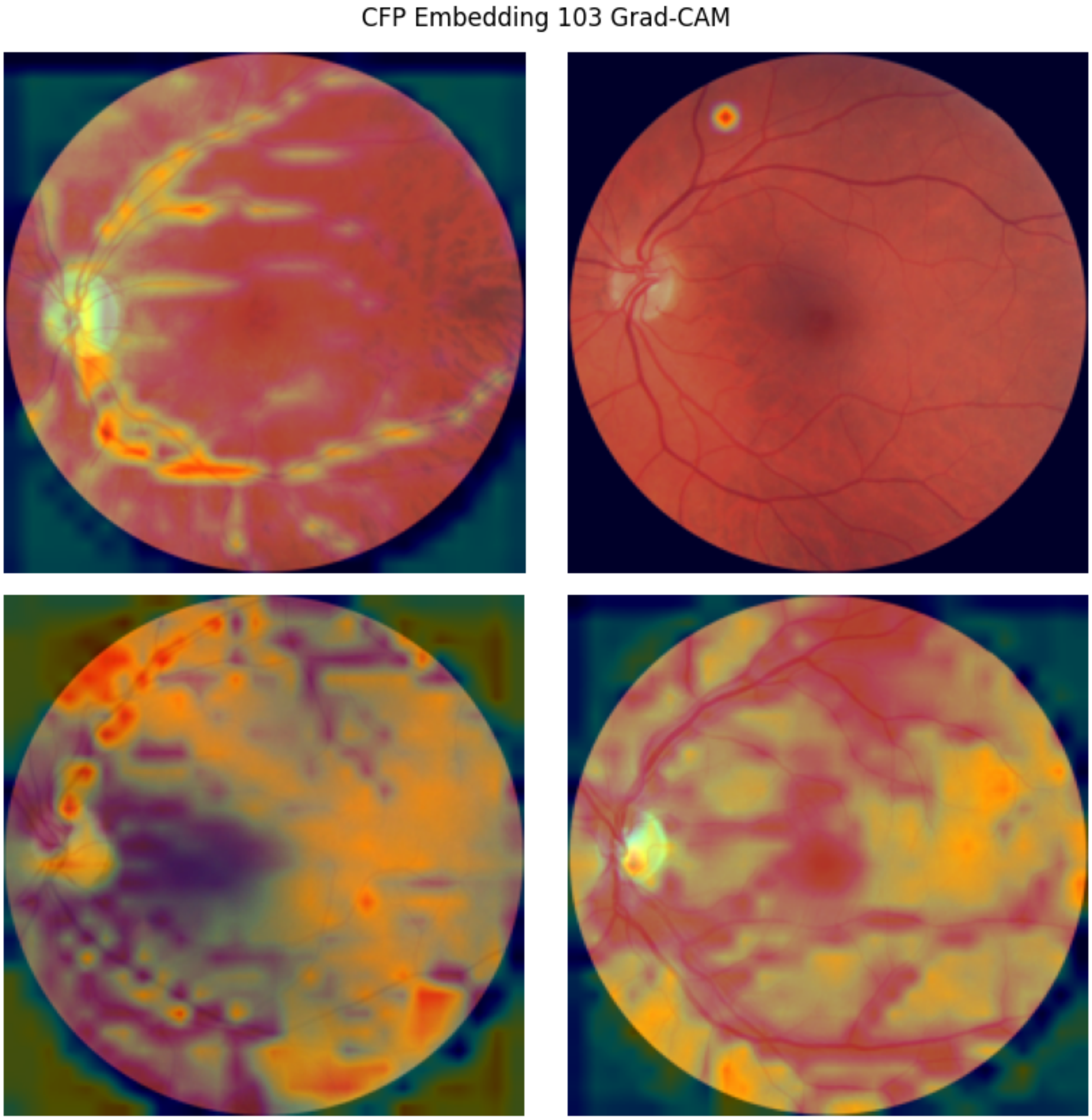

Supplementary Figure 28: Grad-CAM saliency map for CFP embedding 103. This was the CFP feature second most strongly associated with future Alzheimer's disease risk. The embedding seems to localise several features including the optic nerve head, vasculature, and to patchy background features. Reproduced with the permission of UK Biobank.

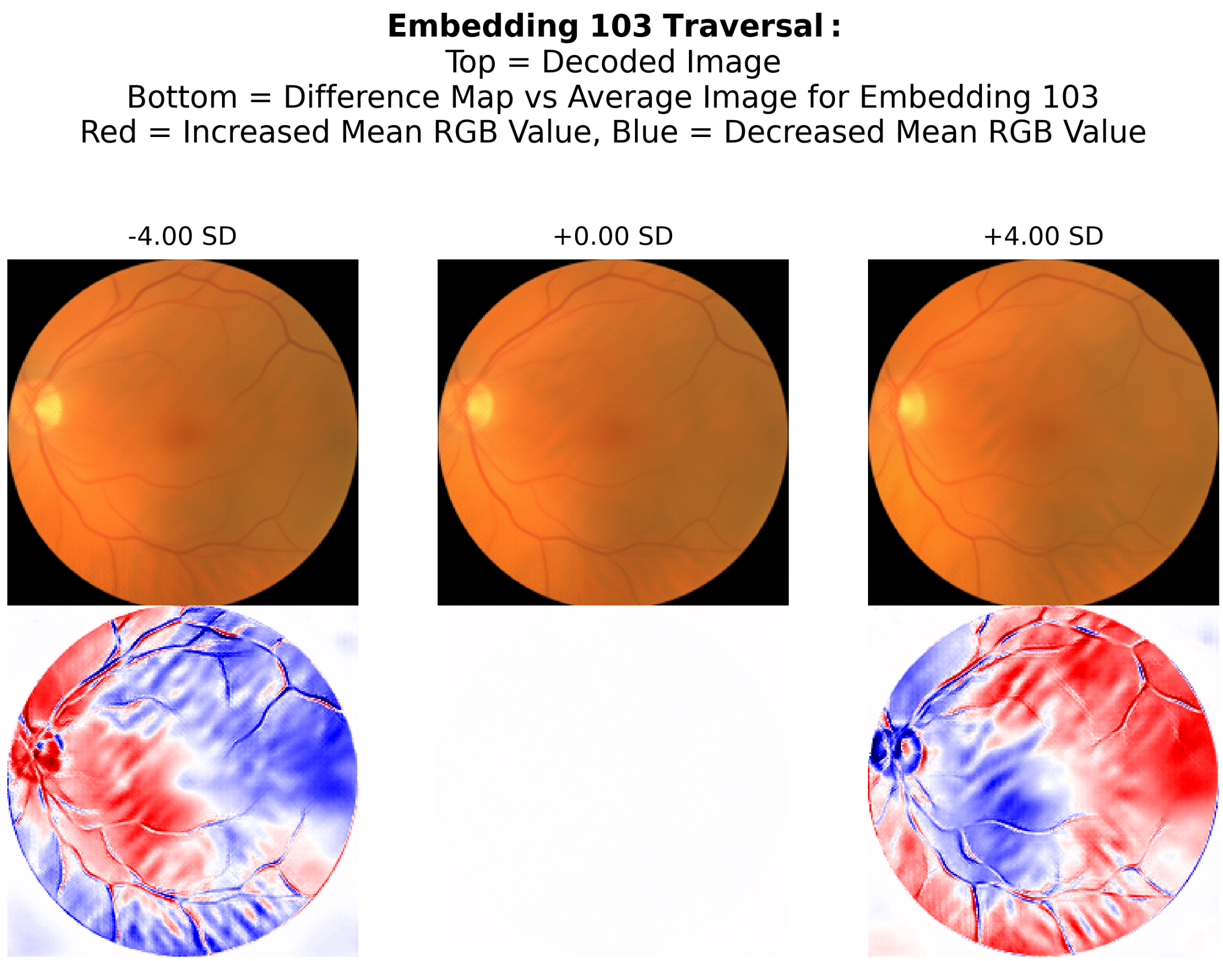

Supplementary Figure 29: Top row - reconstructions obtained by perturbing latent dimension 103 by ±4 standard deviations (SD) around its mean, while holding all other latent dimensions constant. This was the CFP feature second most strongly associated with future Alzheimer's disease risk. The central image corresponds to the reference latent vector. Bottom row - difference maps showing the mean per-pixel change in RGB intensity relative to the reference reconstruction. Red denotes increased mean RGB intensity, and blue denotes decreased intensity.

Perturbation of this latent dimension appears to modulate vascular widths (best seen inferiorly), the branching patterns, and the region occupied by the cup of the optic nerve head. Reproduced with the permission of UK Biobank.

CFP Embedding 106 Grad-CAM

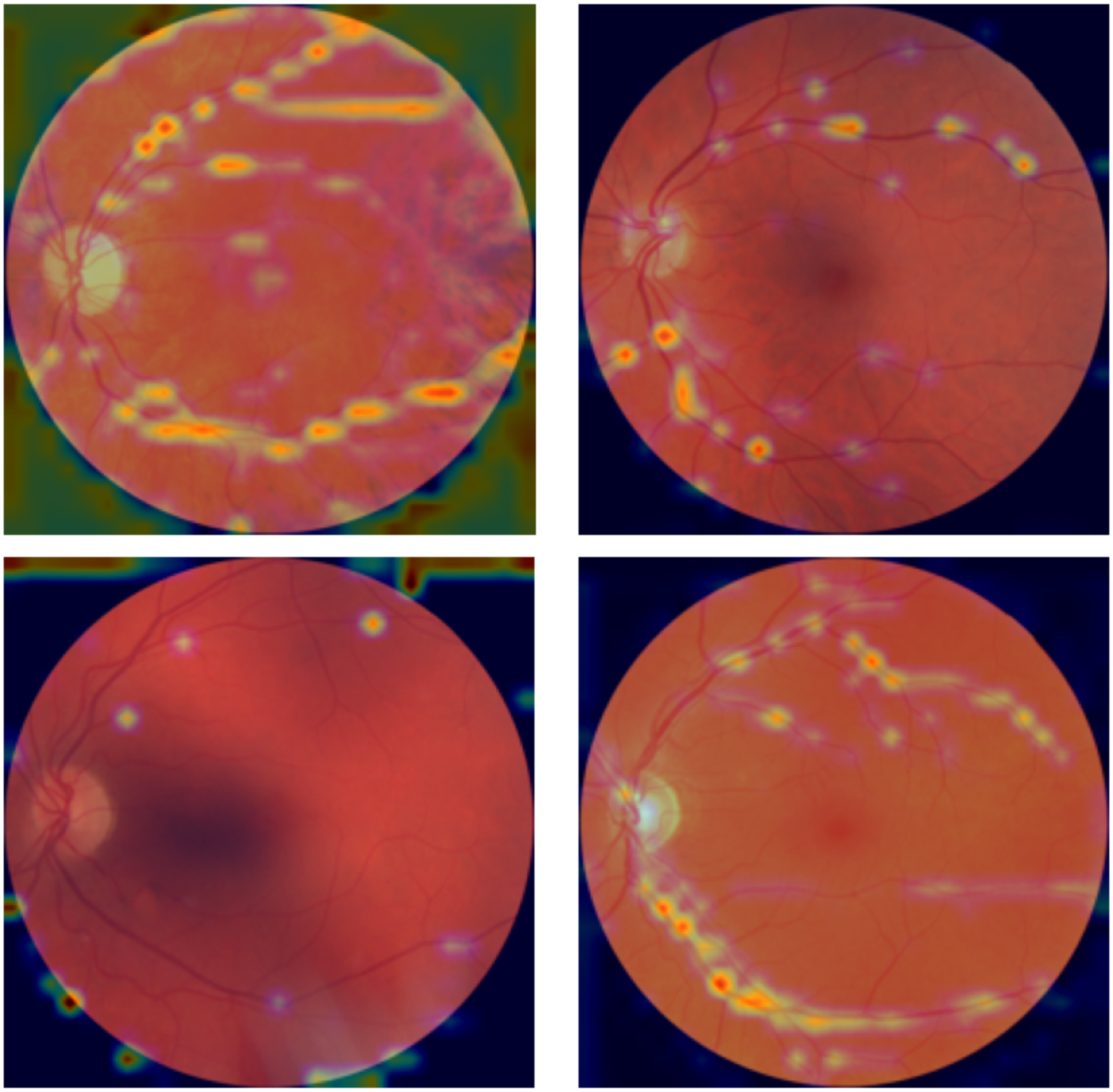

Supplementary Figure 30: Grad-CAM saliency map for CFP embedding 106. This embedding was associated with a large number of cardiovascular features in our Pearson correlation analysis. The feature appears to localise to the vascular tree. Reproduced with the permission of UK Biobank.

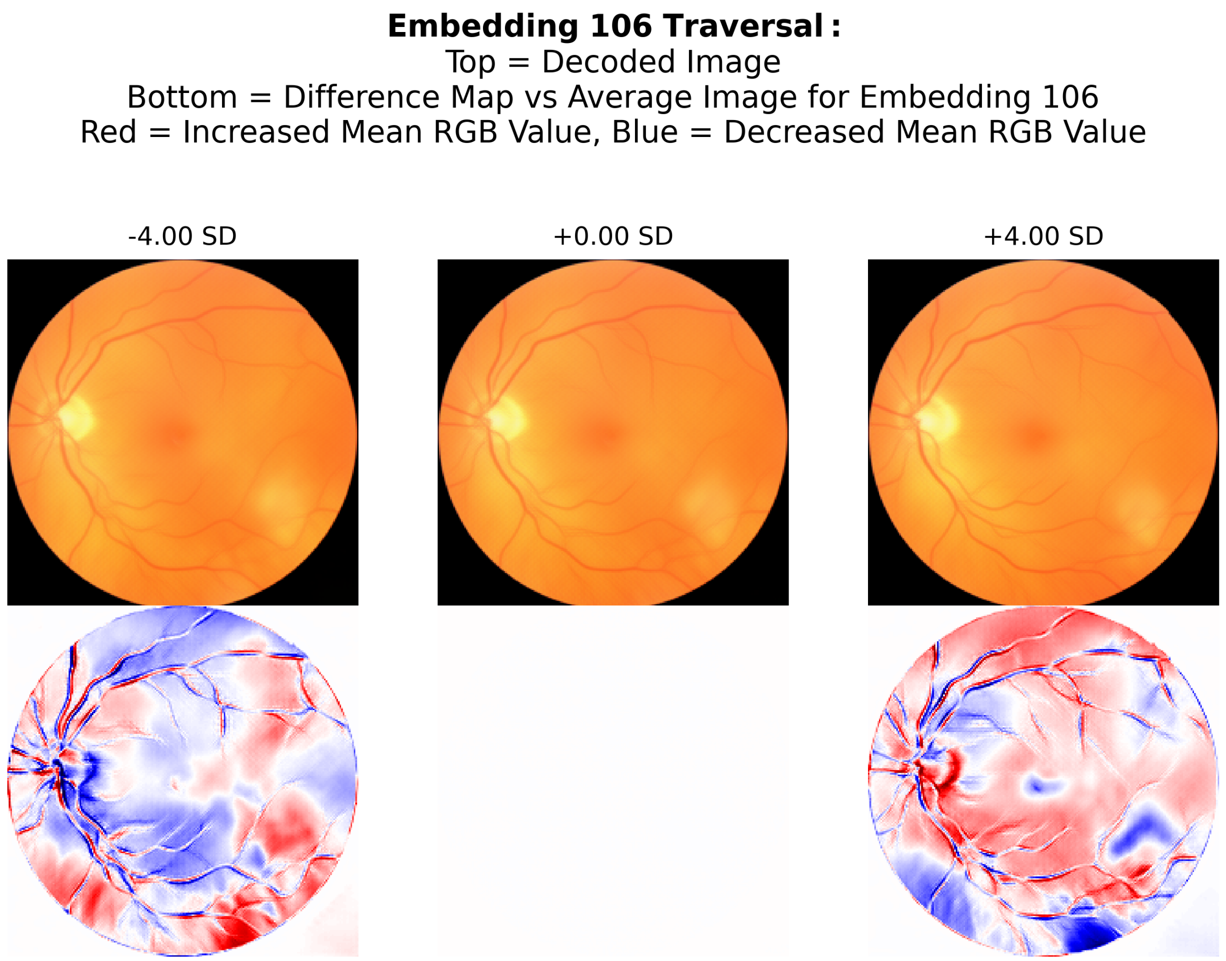

Supplementary Figure 31: Top row - reconstructions obtained by perturbing latent dimension 106 by ±4 standard deviations (SD) around its mean, while holding all other latent dimensions constant. This embedding was associated with many cardiovascular features in our Pearson correlation analysis. The central image corresponds to the reference latent vector. Bottom row - difference maps showing the mean per-pixel change in RGB intensity relative to the reference reconstruction. Red denotes increased mean RGB intensity, and blue denotes decreased intensity.

Perturbation of this latent dimension appears to have subtle effects on the vessel branching patterns and the shape of the optic nerve head. Reproduced with the permission of UK Biobank.

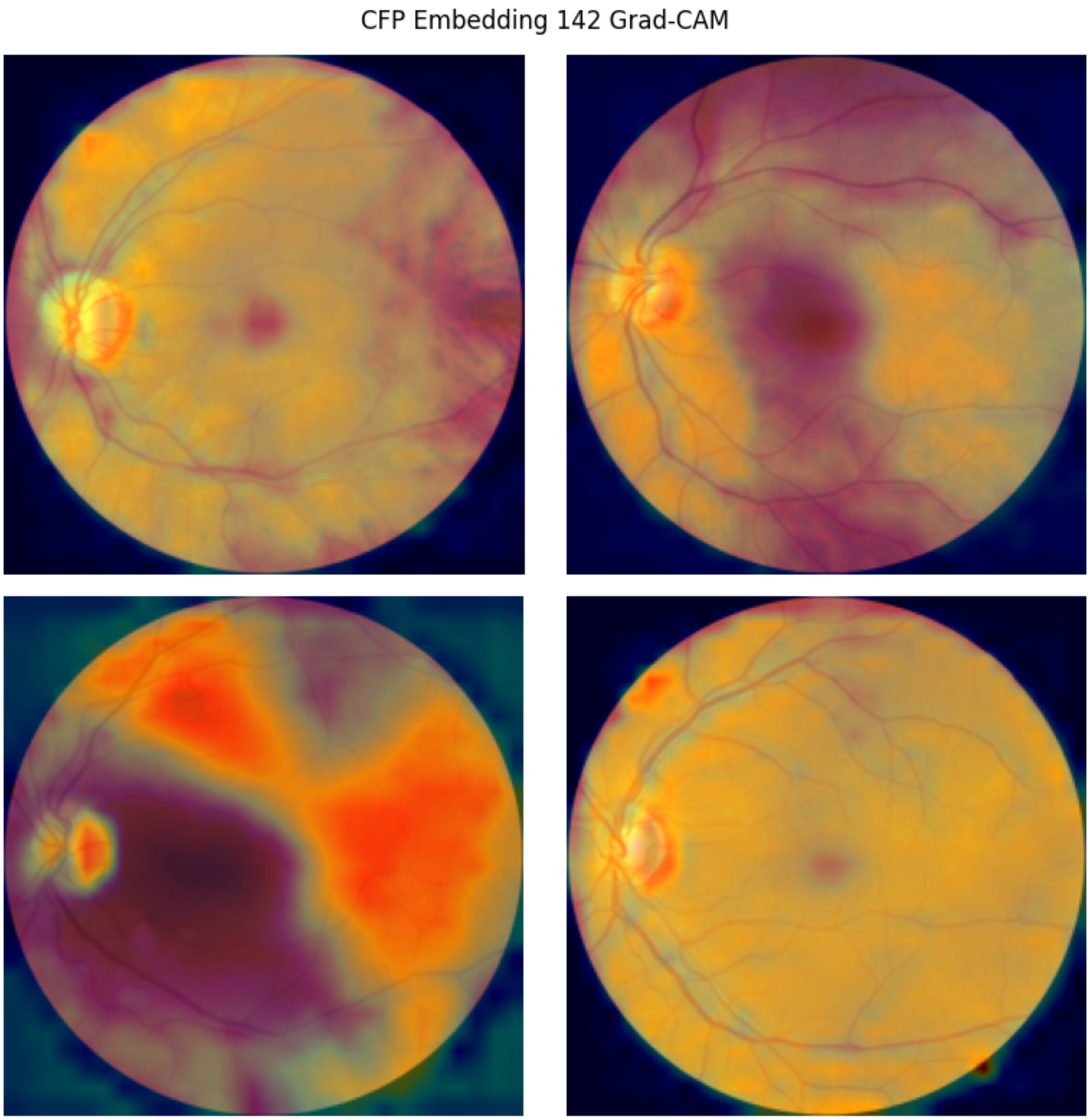

Supplementary Figure 32: Grad-CAM saliency map for CFP embedding 142. This was the CFP feature most strongly associated with the gene set for melanin biosynthesis and interestingly was also the embedding which dominated the correlation with lipids in our CCA analysis. The embedding seems to represent the background features in a relatively homogeneous manner and appears to correspond to the extent of fundal pigmentation. Reproduced with the permission of UK Biobank.

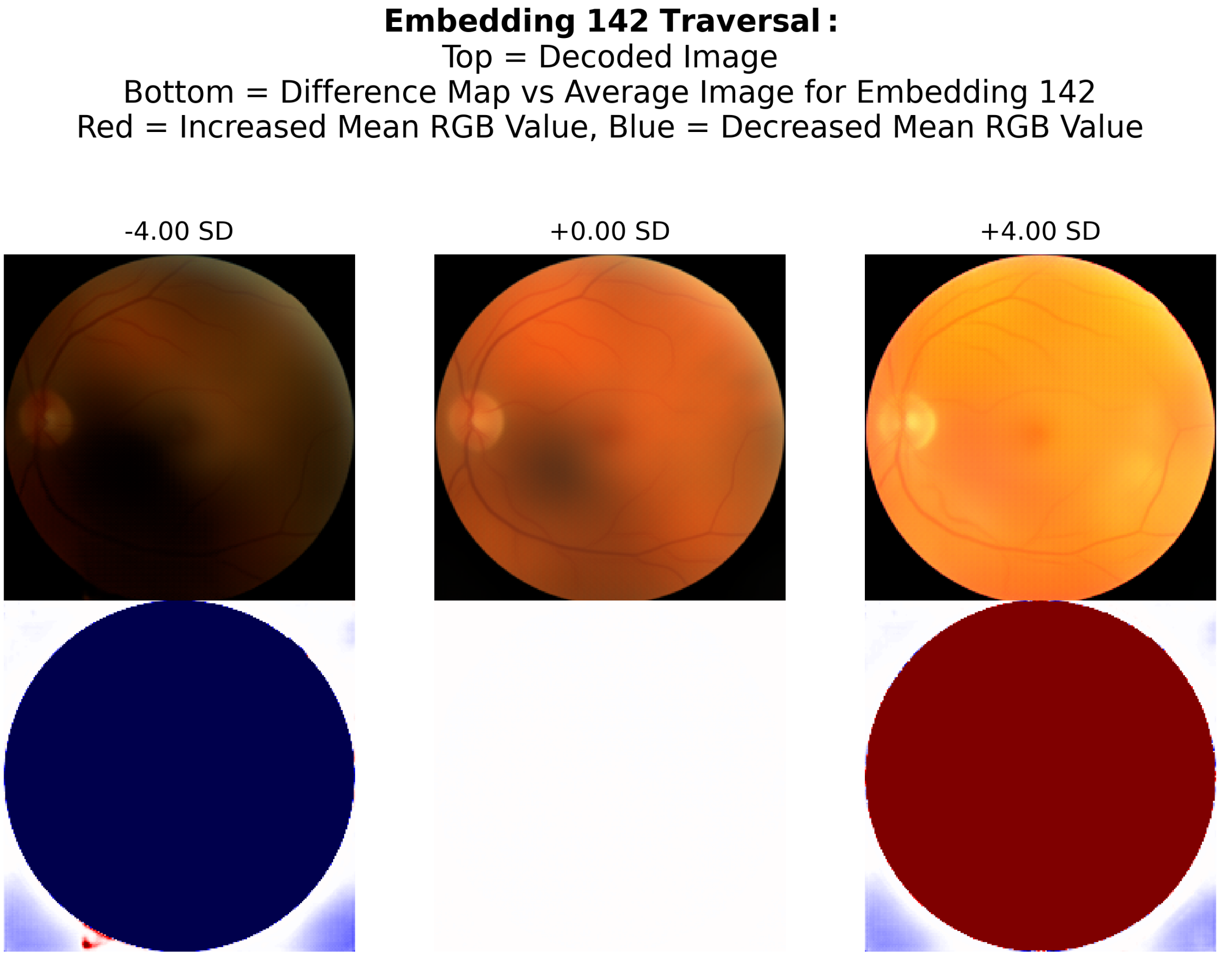

Supplementary Figure 33: Top row - reconstructions obtained by perturbing latent dimension 142 by ±4 standard deviations (SD) around its mean, while holding all other latent dimensions constant. This was the CFP feature most strongly associated with the gene set for melanin biosynthesis and interestingly was also the embedding which dominated the correlation with lipids in our CCA analysis. The central image corresponds to the reference latent vector. Bottom row - difference maps showing the mean per-pixel change in RGB intensity relative to the reference reconstruction. Red denotes increased mean RGB intensity, and blue denotes decreased intensity.

Perturbation of this latent dimension has a clear effect on fundal pigmentation and illuminance. Reproduced with the permission of UK Biobank.

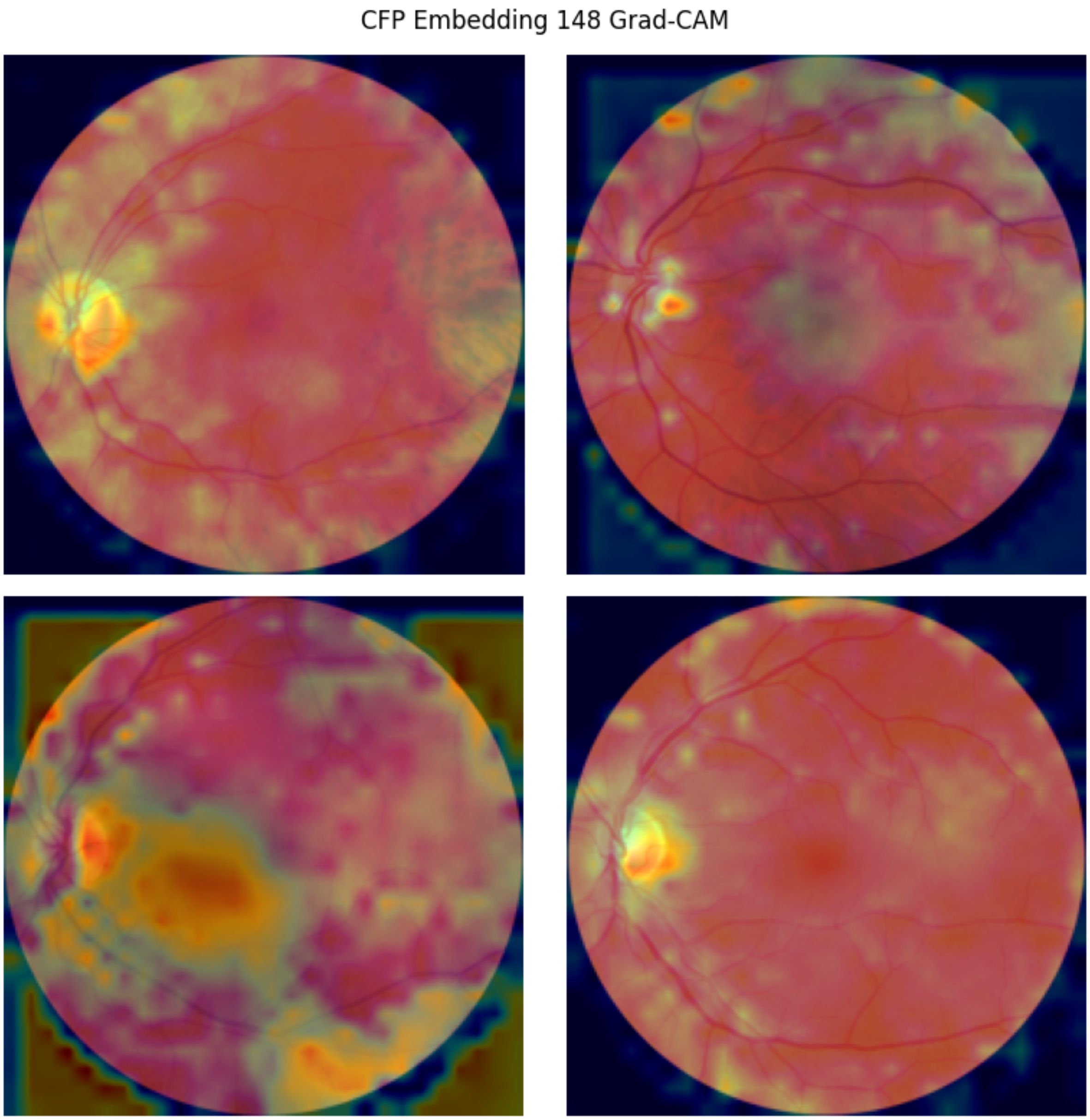

Supplementary Figure 34: Grad-CAM saliency map for CFP embedding 148. This was the embedding most strongly associated with hypertension and Parkinson's disease at the time of imaging (baseline). This was the embedding most strongly associated with future risk of hypertension. Our radiomic analysis revealed this embedding was associated with a large number of cerebral volumes and diffusion MRI features. Genetic analyses revealed that this embedding was associated with melanin biosynthesis. The embedding appears to localise to the optic nerve head, pigmented areas, and the projection artifacts. Reproduced with the permission of UK Biobank.

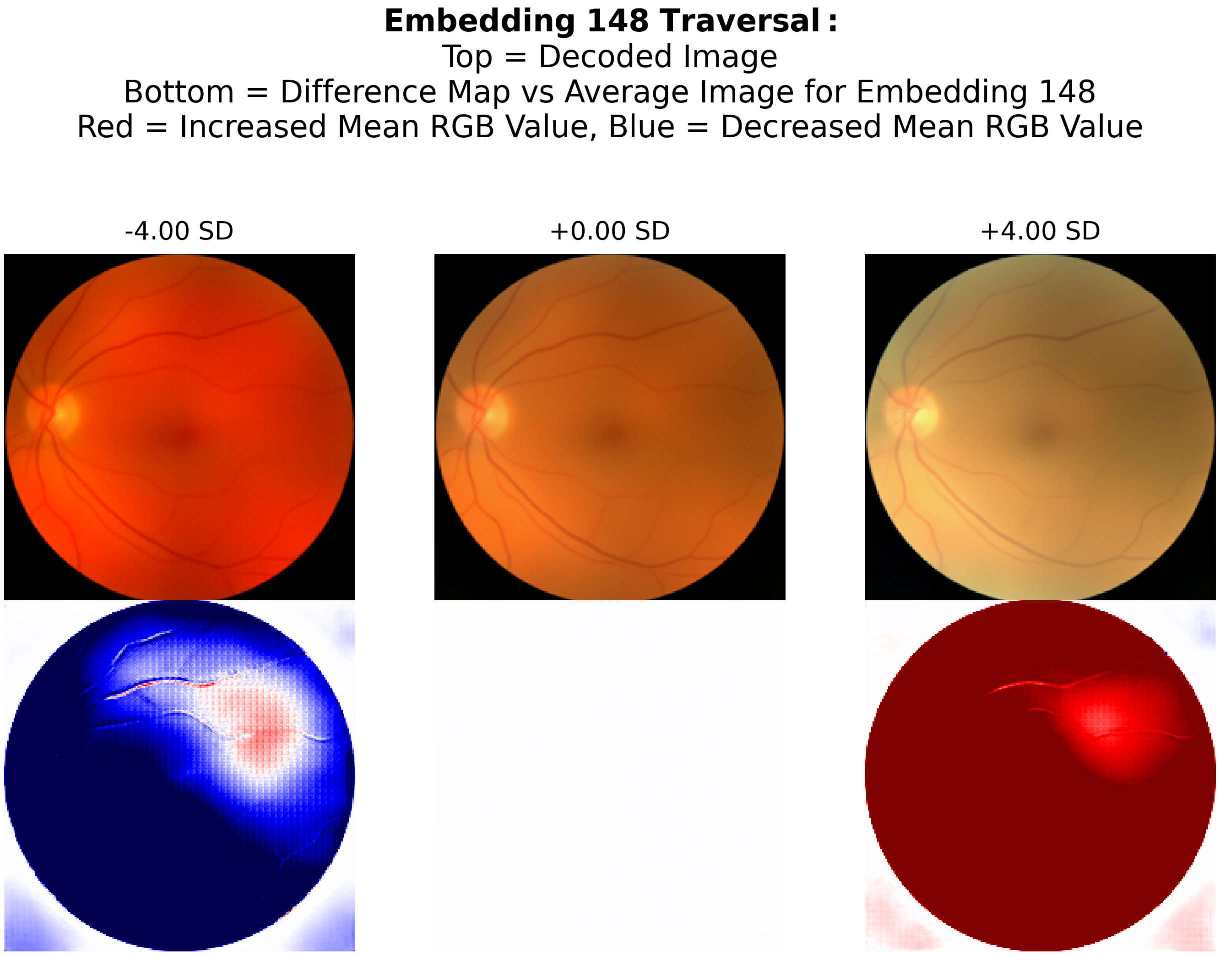

Supplementary Figure 35: Top row - reconstructions obtained by perturbing latent dimension 148 by ±4 standard deviations (SD) around its mean, while holding all other latent dimensions constant. This was the embedding most strongly associated with hypertension and Parkinson's disease at the time of imaging (baseline). This was the embedding most strongly associated with future risk of hypertension. Genetic analyses revealed that this embedding was associated with melanin biosynthesis. The central image corresponds to the reference latent vector. Bottom row - difference maps showing the mean per-pixel change in RGB intensity relative to the reference reconstruction. Red denotes increased mean RGB intensity, and blue denotes decreased intensity.

Perturbation of this latent dimension has a clear effect on fundal pigmentation. Reproduced with the permission of UK Biobank.

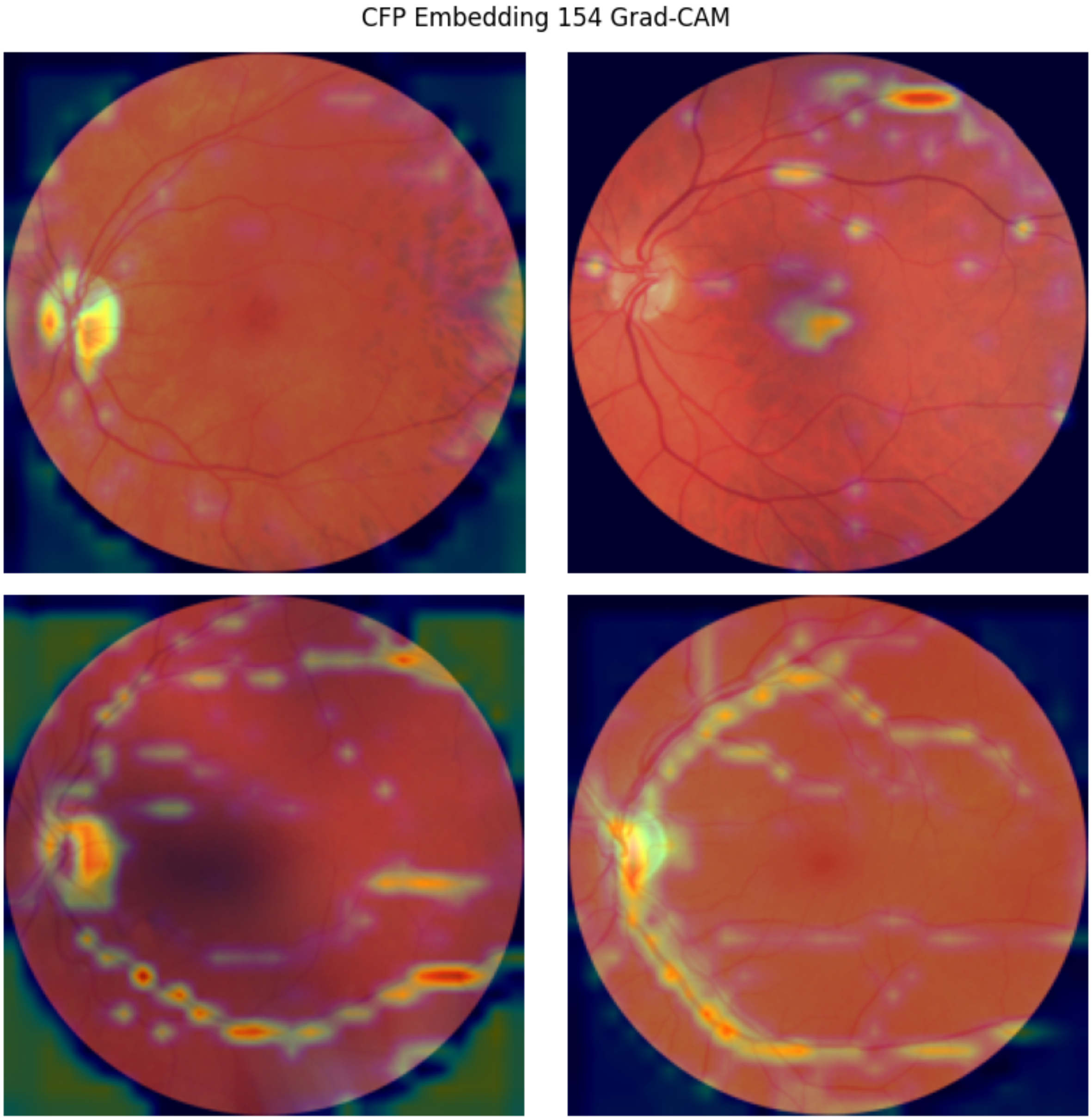

Supplementary Figure 36: Grad-CAM saliency map for CFP embedding 154. This was the embedding most strongly associated with transient ischaemic attack at the time of imaging (baseline). The embedding seems to localise to the optic nerve head and vasculature. Reproduced with the permission of UK Biobank.

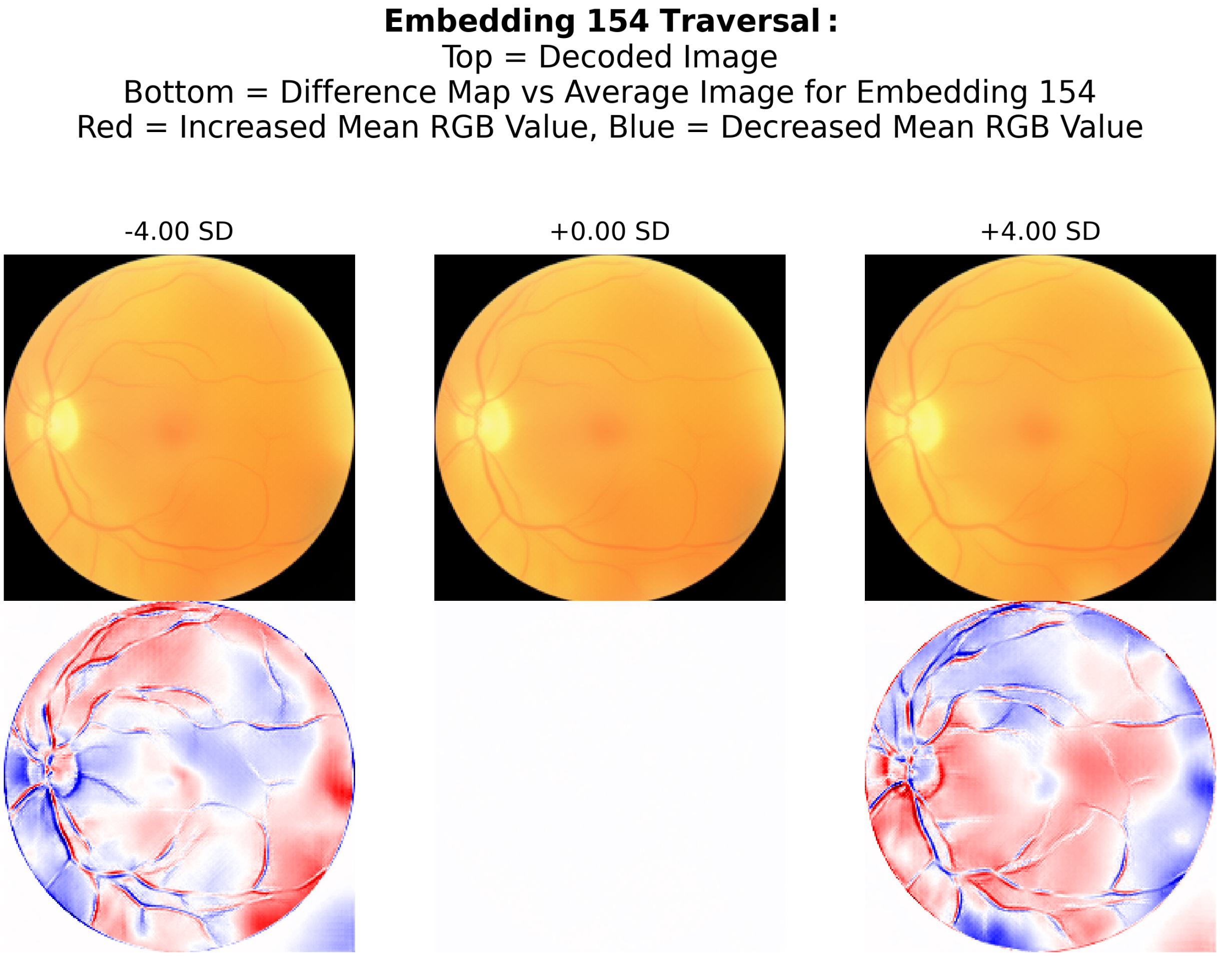

Supplementary Figure 37: Top row - reconstructions obtained by perturbing latent dimension 154 by ±4 standard deviations (SD) around its mean, while holding all other latent dimensions constant. This was the embedding most strongly associated with transient ischaemic attack at the time of imaging (baseline). The central image corresponds to the reference latent vector. Bottom row - difference maps showing the mean per-pixel change in RGB intensity relative to the reference reconstruction. Red denotes increased mean RGB intensity, and blue denotes decreased intensity.

Perturbation of this latent dimension subtly affects vascular branching patterns. Reproduced with the permission of UK Biobank.

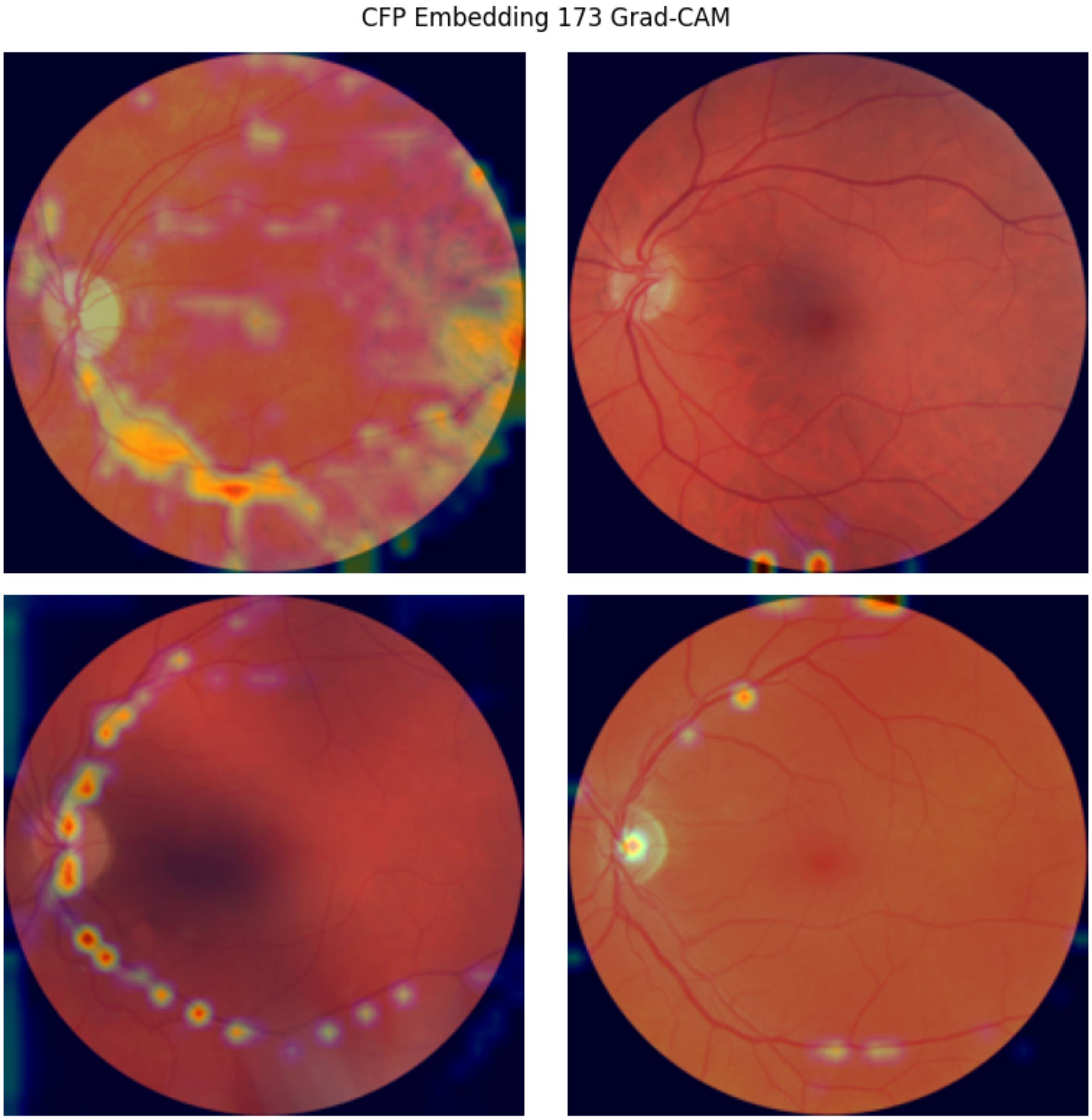

Supplementary Figure 38: Grad-CAM saliency map for CFP embedding 173. This was the CFP feature most strongly associated with the gene set ‘Reactome phospholipase C mediated cascade *FGFR2’*. The embedding seems to localise to the vasculature and optic nerve head. Reproduced with the permission of UK Biobank.

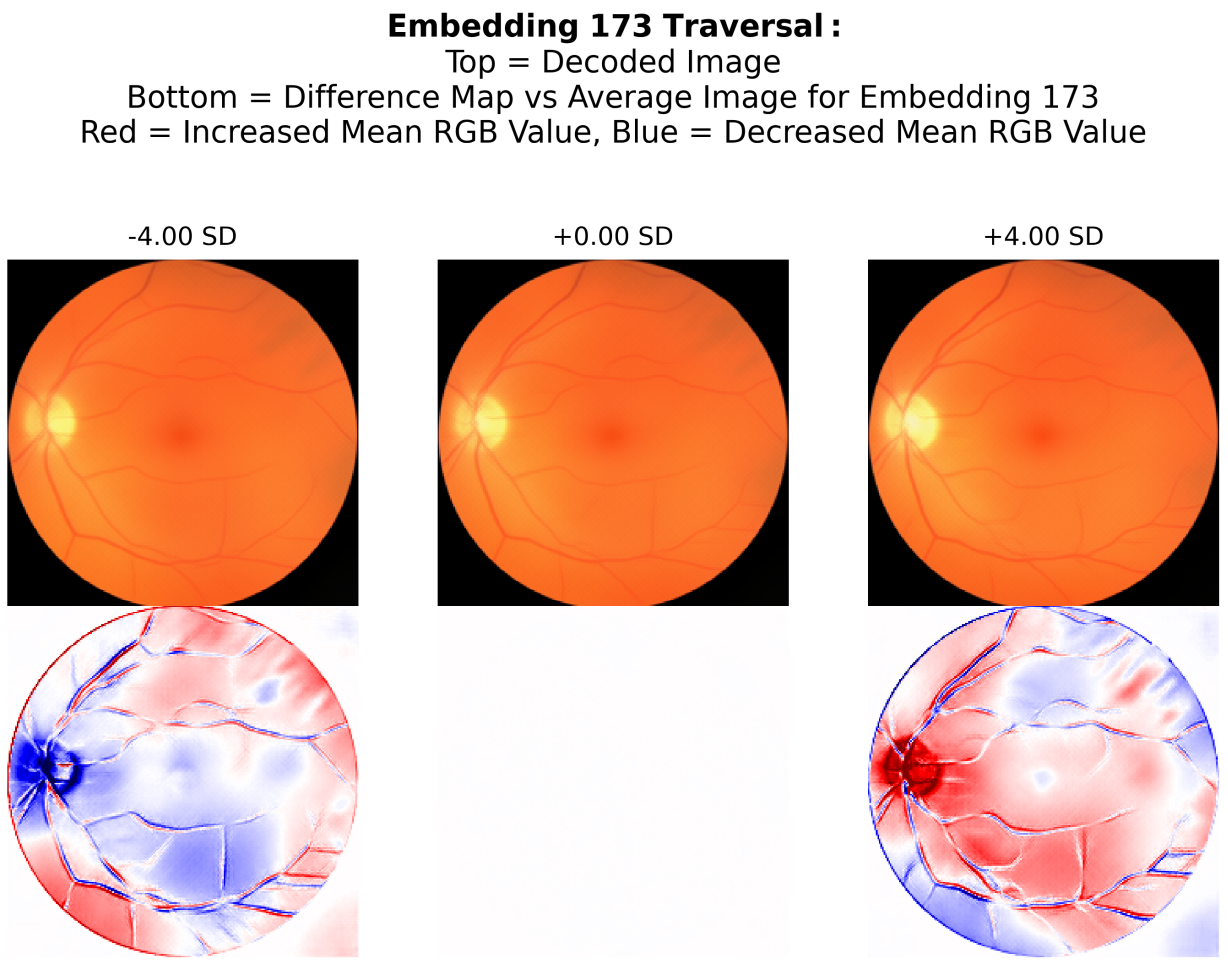

Supplementary Figure 39: Top row - reconstructions obtained by perturbing latent dimension 173 by ±4 standard deviations (SD) around its mean, while holding all other latent dimensions constant. This was the CFP feature most strongly associated with the gene set 'Reactome phospholipase C mediated cascade *FGFR2'*. The central image corresponds to the reference latent vector. Bottom row - difference maps showing the mean per-pixel change in RGB intensity relative to the reference reconstruction. Red denotes increased mean RGB intensity, and blue denotes decreased intensity.

Perturbation of this latent dimension appears to modulate the pallor of the optic nerve head and affects the branching pattern of the vasculature, best seen in the inferior arcade. Reproduced with the permission of UK Biobank.

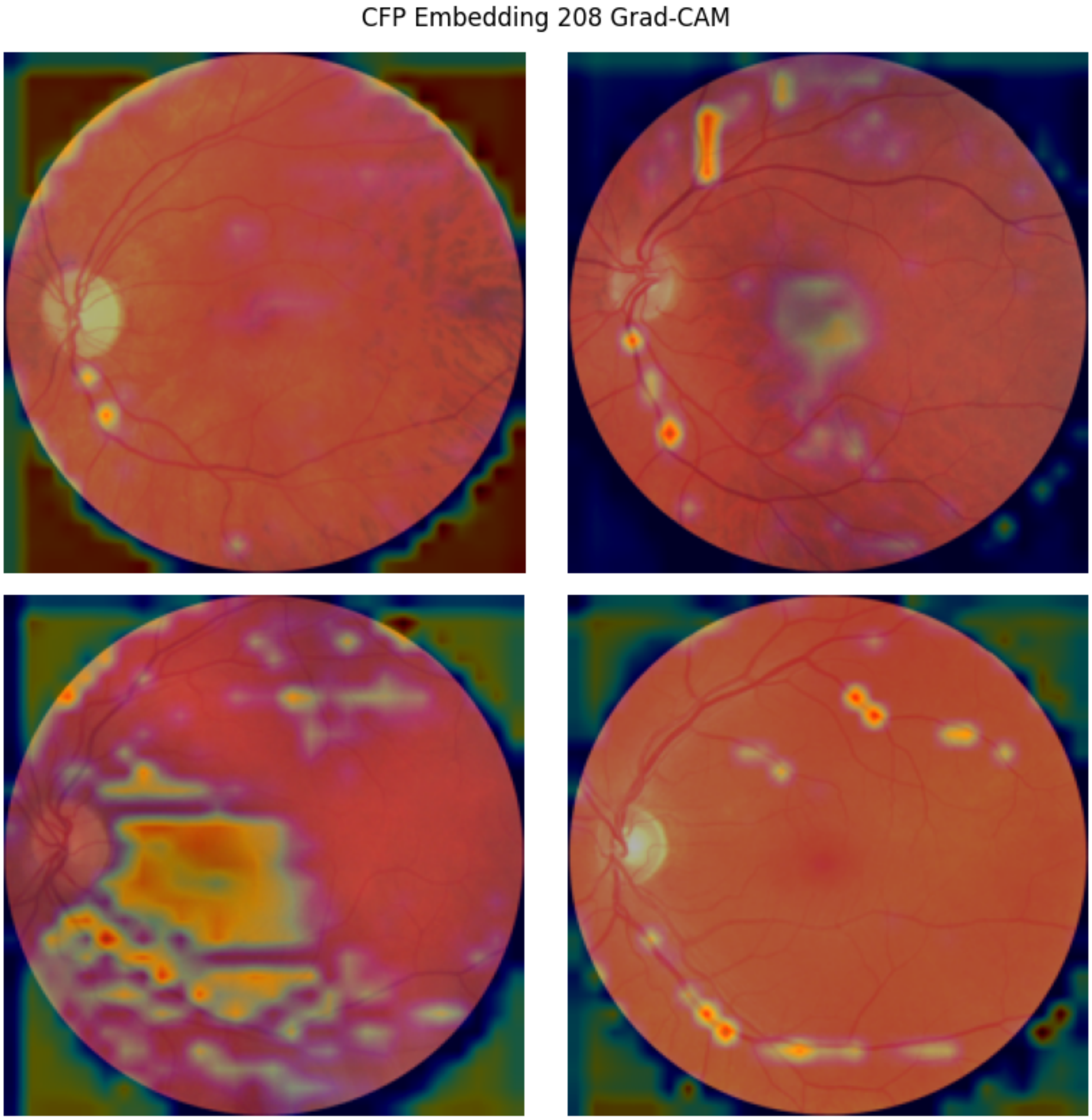

Supplementary Figure 40: Grad-CAM saliency map for CFP embedding 208. This was the third most strongly associated with future hypertension risk. The embedding seems to localise to the vasculature and the projection artifact in the bottom left image. Reproduced with the permission of UK Biobank.

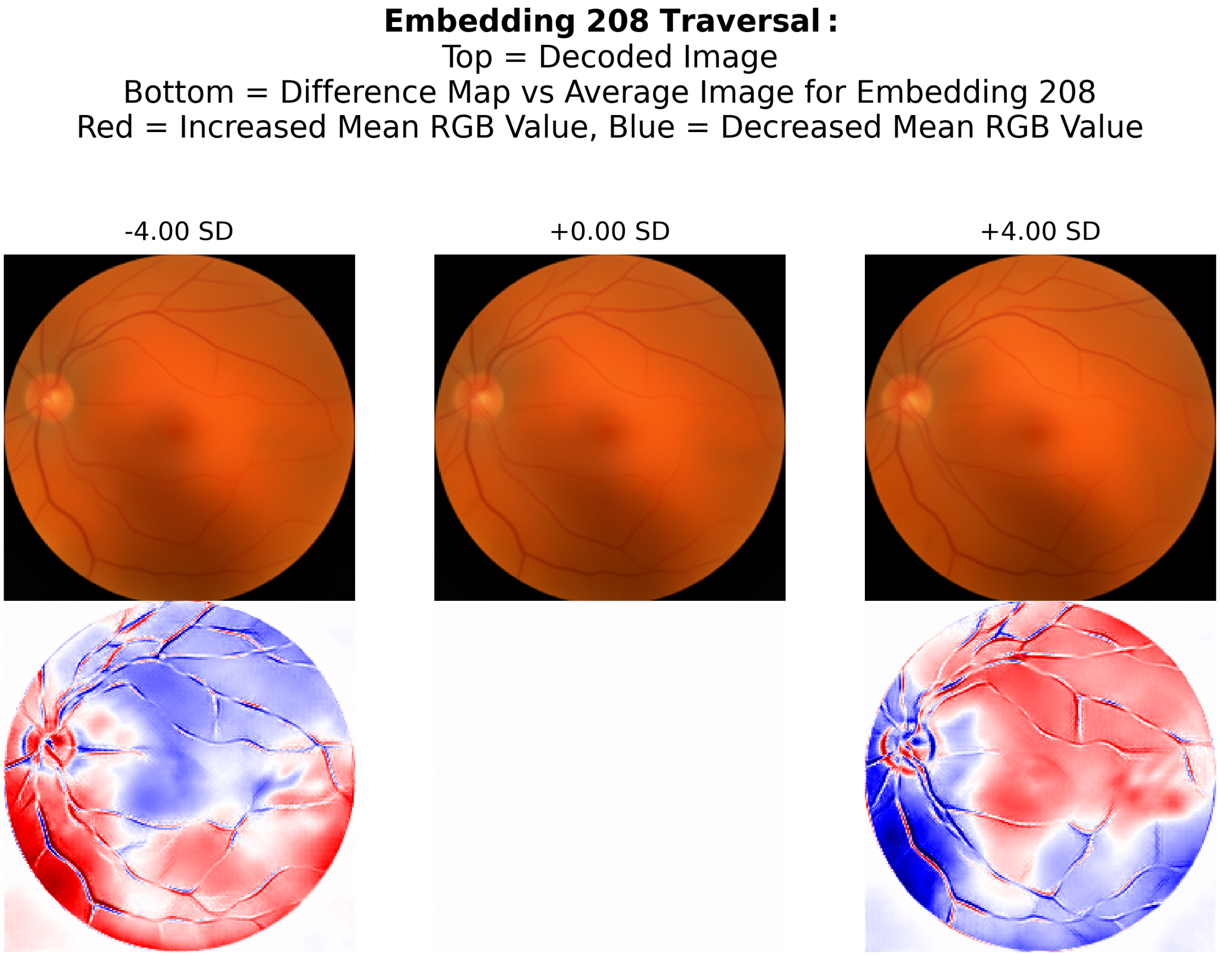

Supplementary Figure 41: Top row - reconstructions obtained by perturbing latent dimension 208 by ±4 standard deviations (SD) around its mean, while holding all other latent dimensions constant. This was the third most strongly associated with future hypertension risk. The central image corresponds to the reference latent vector. Bottom row - difference maps showing the mean per-pixel change in RGB intensity relative to the reference reconstruction. Red denotes increased mean RGB intensity, and blue denotes decreased intensity.

Perturbation of this latent feature influences the appearance of the vasculature both inferiorly and superiorly, with changes in branching complexity and the tortuosity of vessels. Reproduced with the permission of UK Biobank.

CFP Embedding 218 Grad-CAM

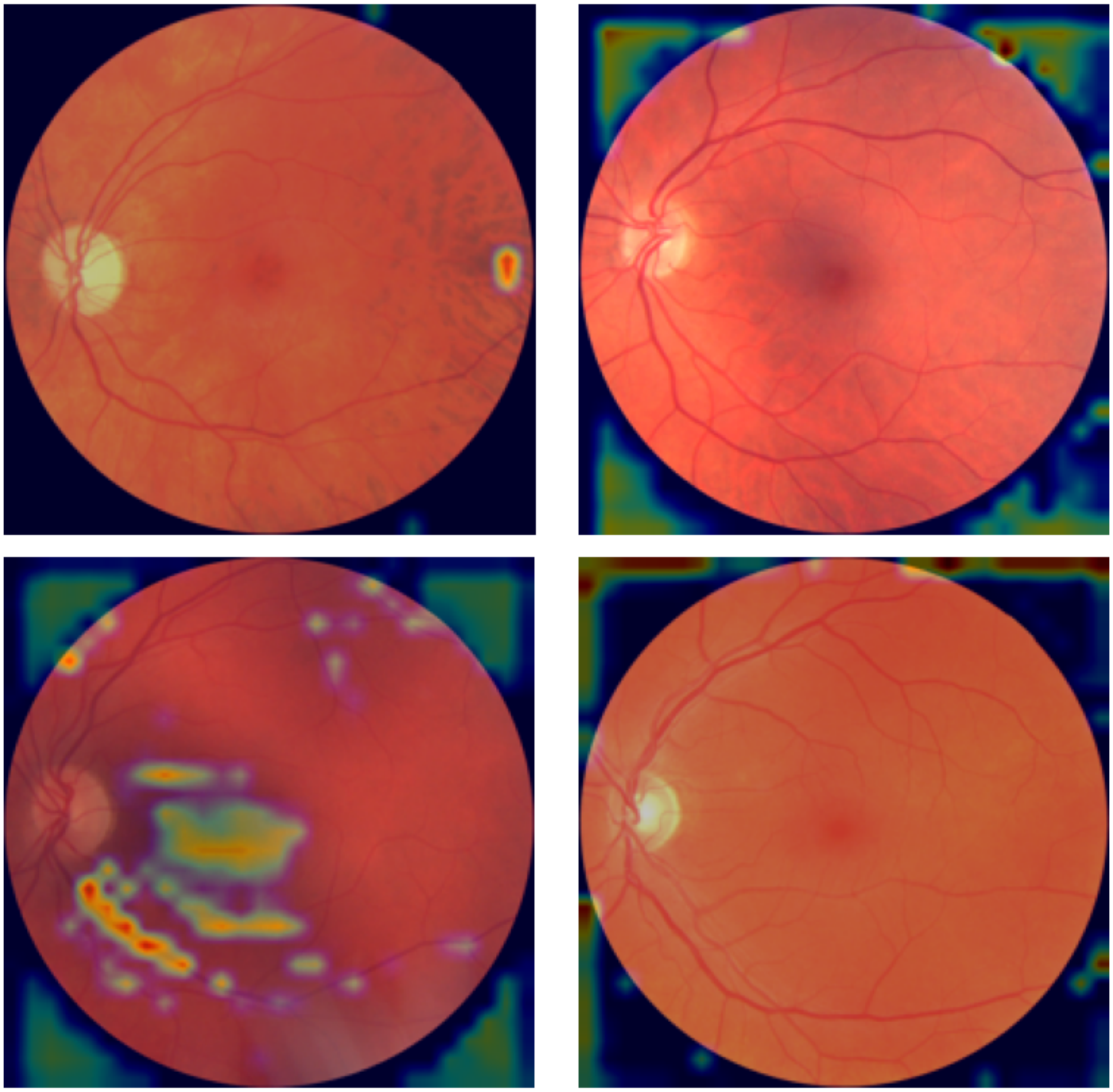

Supplementary Figure 42: Grad-CAM saliency map for CFP embedding 218. This was the embedding most strongly associated with angina at the time of imaging (baseline). The embedding seems to localise to the vasculature. Reproduced with the permission of UK Biobank.

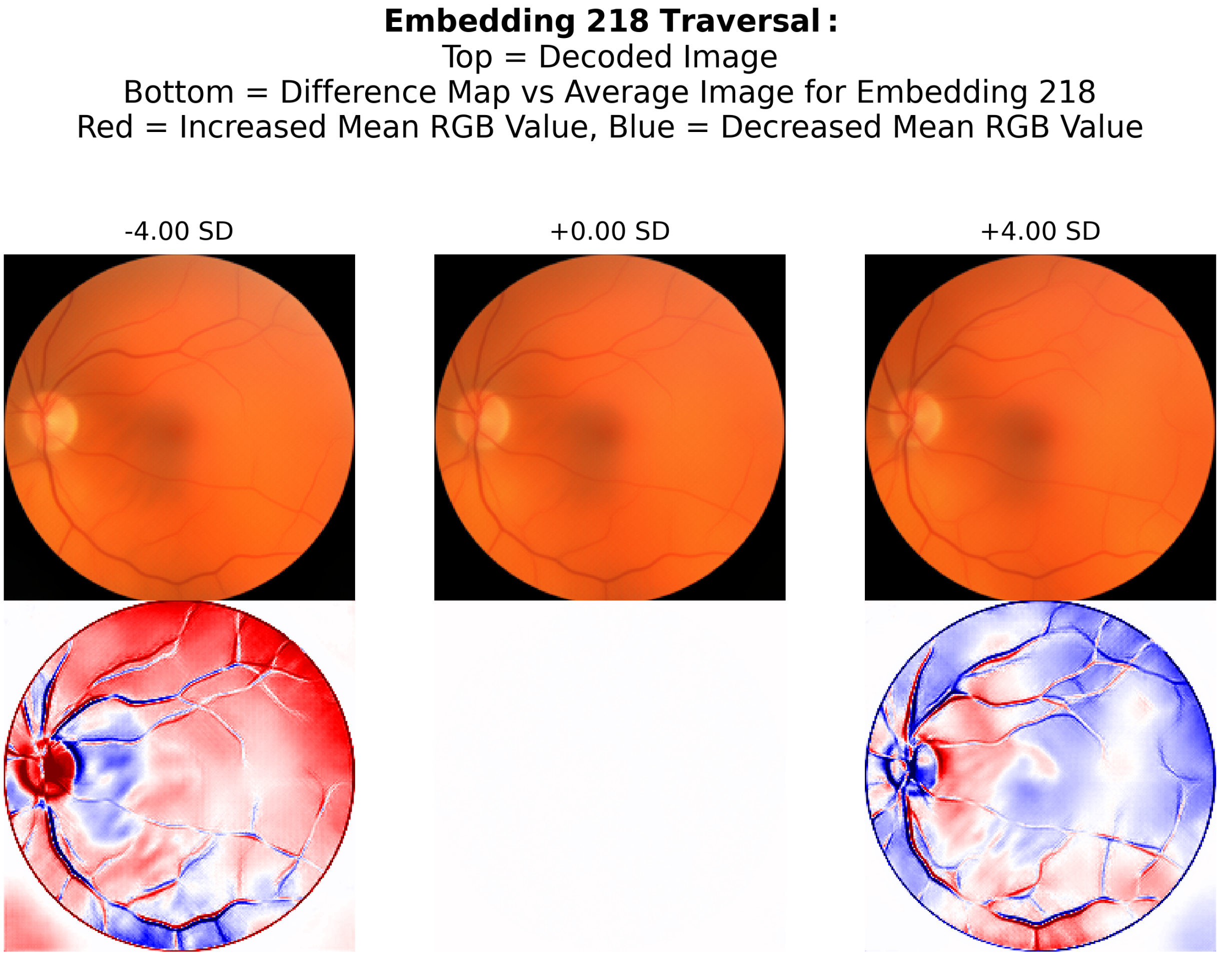

Supplementary Figure 43: Top row - reconstructions obtained by perturbing latent dimension 218 by ±4 standard deviations (SD) around its mean, while holding all other latent dimensions constant. This was the embedding most strongly associated with angina at the time of imaging (baseline). The central image corresponds to the reference latent vector. Bottom row - difference maps showing the mean per-pixel change in RGB intensity relative to the reference reconstruction. Red denotes increased mean RGB intensity, and blue denotes decreased intensity.

Perturbation of this latent dimension appears to subtly affect the vessel branching patterns and some vessel widths and colours, but the difference maps suggest that the optic nerve head RGB values are substantially altered by this embedding value in a manner that is challenging to appreciate with the human eye. Reproduced with the permission of UK Biobank.

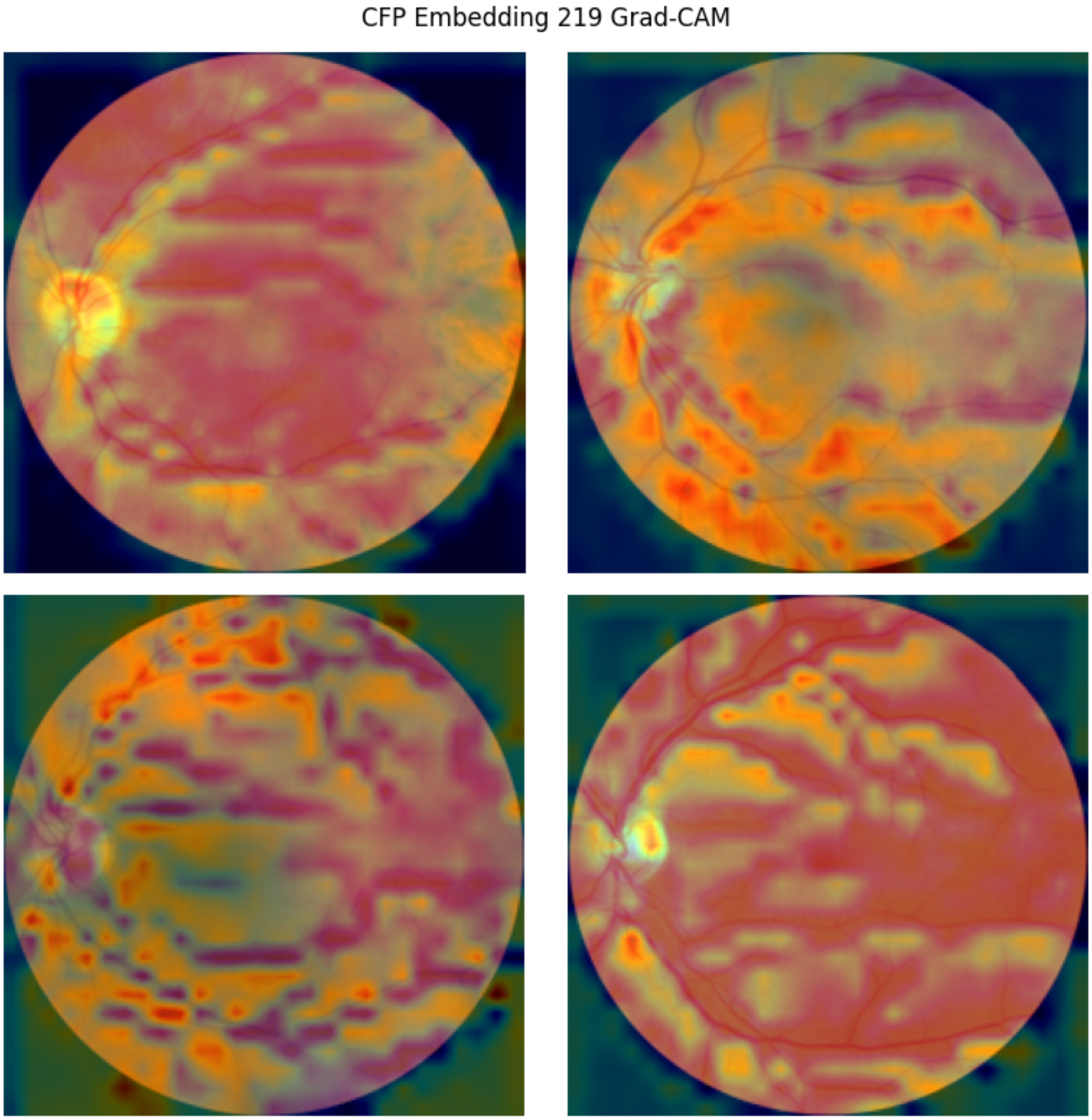

Supplementary Figure 44: Grad-CAM saliency map for CFP embedding 219. This embedding was associated with future risk of dementia in diseases ‘classified elsewhere’ (inclusive of Pick disease, Creutzfeldt-Jakob disease, Parkinson’s disease, Huntington disease, human immunodeficiency virus and others). This embedding appears to localise principally to perivascular regions. Reproduced with the permission of UK Biobank.

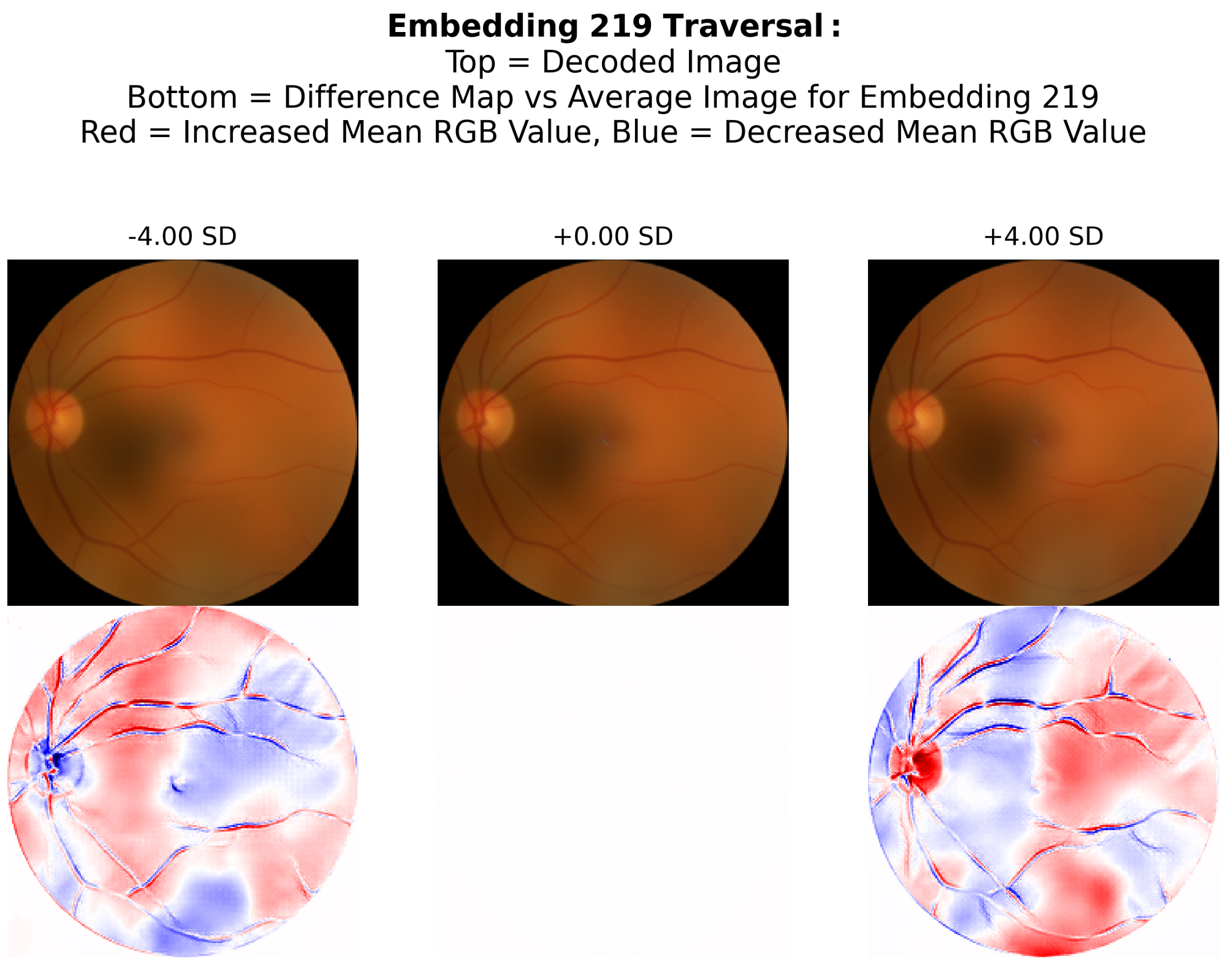

Supplementary Figure 45: Top row - reconstructions obtained by perturbing latent dimension 219 by ±4 standard deviations (SD) around its mean, while holding all other latent dimensions constant. This embedding was associated with future risk of dementia in diseases 'classified elsewhere' (inclusive of Pick disease, Creutzfeldt-Jakob disease, Parkinson's disease, Huntington disease, human immunodeficiency virus and others). The central image corresponds to the reference latent vector. Bottom row - difference maps showing the mean per-pixel change in RGB intensity relative to the reference reconstruction. Red denotes increased mean RGB intensity, and blue denotes decreased intensity. Perturbation of this latent dimension most obviously affects the tortuosity of the blood vessel closest to the fovea superiorly. However, the difference map suggests that the embedding also modulates the RGB values at the optic nerve head. Reproduced with the permission of UK Biobank.

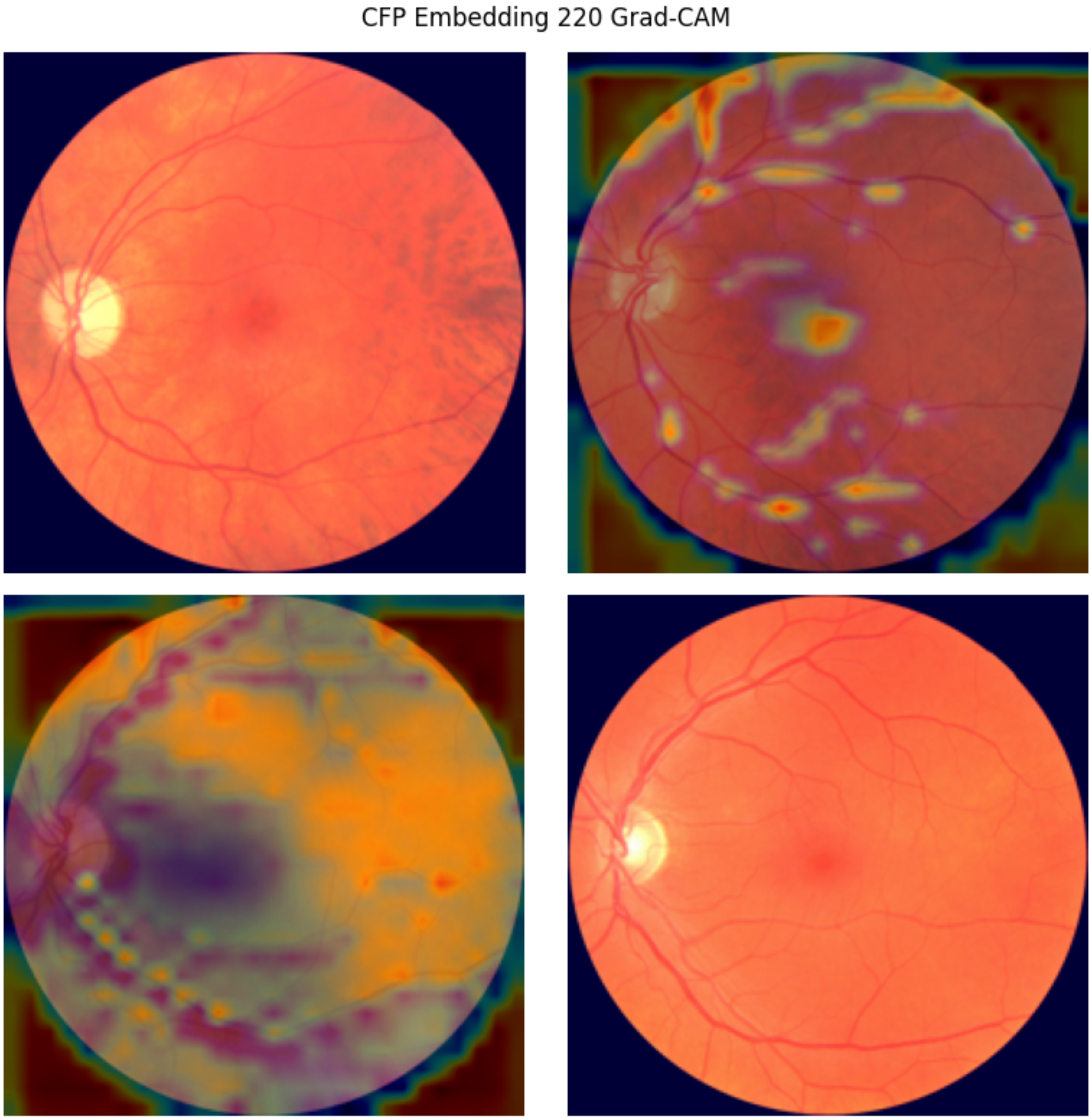

Supplementary Figure 46: Grad-CAM saliency map for CFP embedding 220. This embedding was genetically correlated with myocardial infarction. The feature appears to localise to vascular structures and perhaps the fovea.

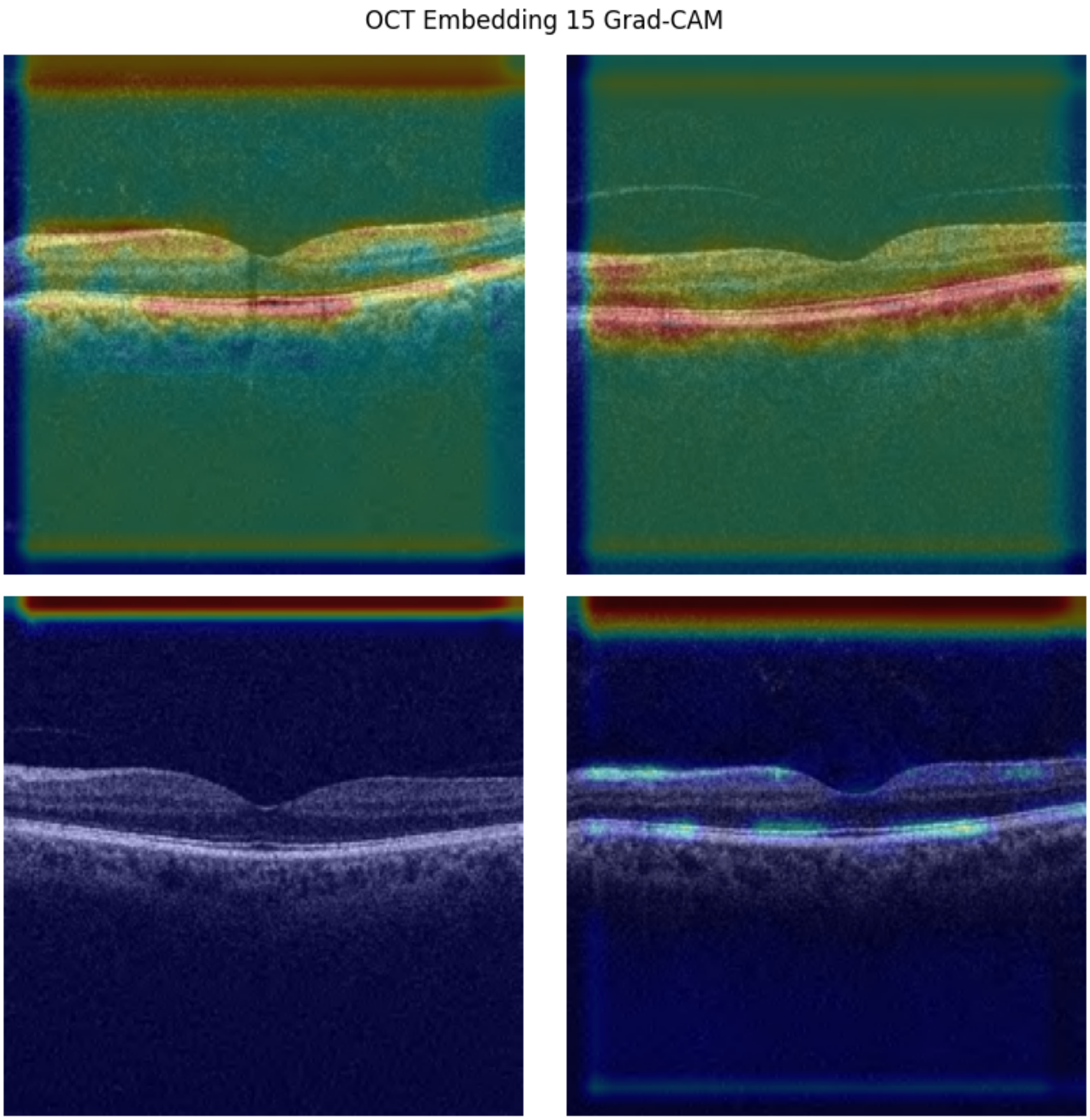

Supplementary Figure 47: Grad-CAM saliency map for OCT embedding 15. This embedding was the OCT embedding most strongly associated with baseline Parkinson's disease. The second convolutional layer was targeted due to poor localisation in the third convolution. The embedding appears to localise to the ellipsoid zone (the location of photoreceptors) and retinal nerve fibre layer. Reproduced with the permission of UK Biobank.

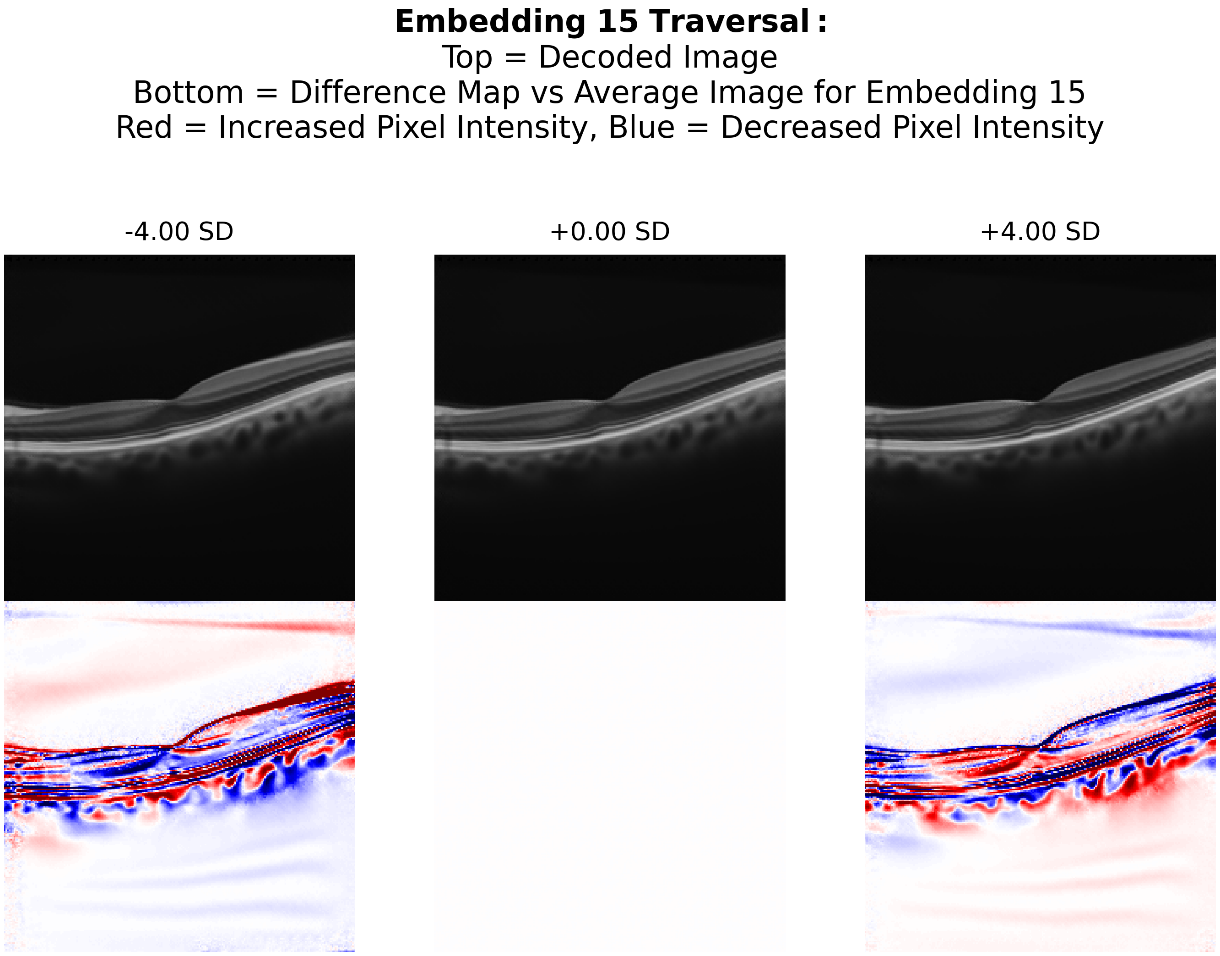

Supplementary Figure 48: Top row - reconstructions obtained by perturbing latent dimension 15 by ±4 standard deviations (SD) around its mean, while holding all other latent dimensions constant. This embedding was the OCT embedding most strongly associated with baseline Parkinson's disease. The central image corresponds to the reference latent vector. Bottom row - difference maps showing the per-pixel change in intensity relative to the reference reconstruction. Red denotes increased intensity, and blue denotes decreased intensity.

The most visually obvious impact of perturbation of this latent dimension is thinning of the temporal retinal nerve fibre layer (the superior, bright layer on the right side of the image) from -4.00SD to +4.00 SD. However, the difference map suggests there are broader pixel intensity differences across all layers that are more challenging to identify with the human eye. Reproduced with the permission of UK Biobank.

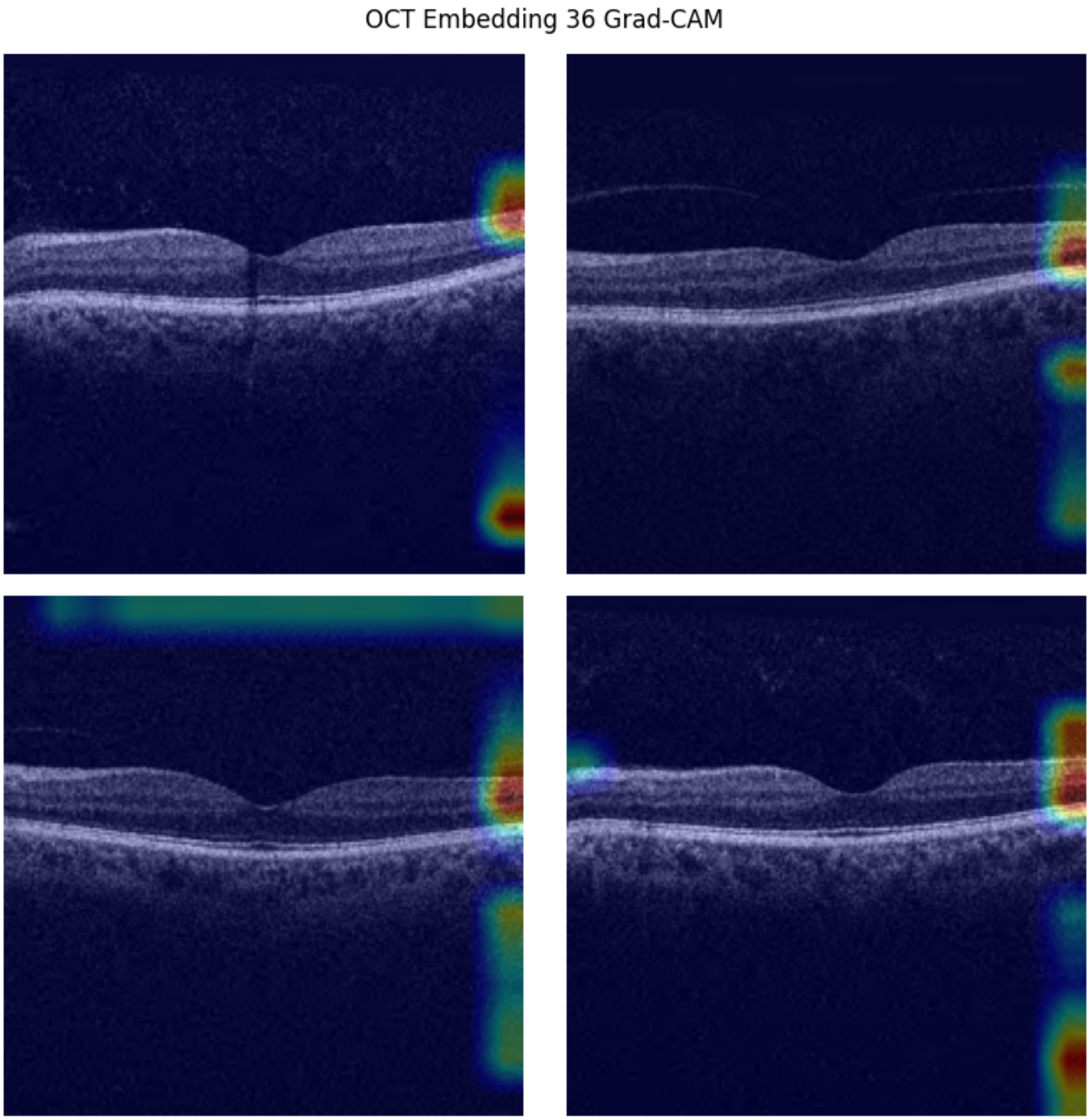

Supplementary Figure 49: Grad-CAM saliency map for OCT embedding 36. This was the OCT feature most strongly associated with dementia-related gene sets. The embedding does not clearly localise to a specific ophthalmic feature in Grad-CAM. Reproduced with the permission of UK Biobank.

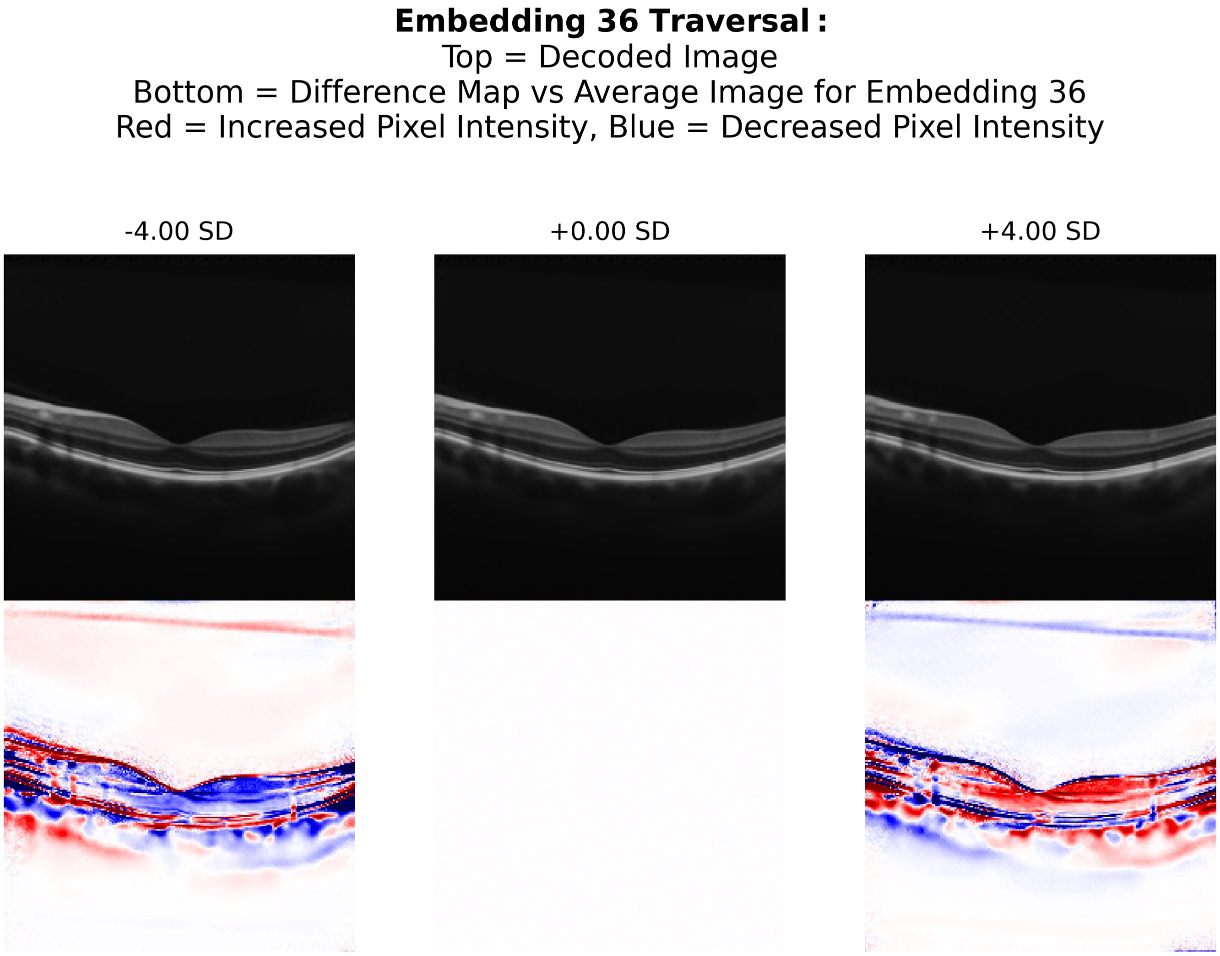

Supplementary Figure 50: Top row - reconstructions obtained by perturbing latent dimension 36 by ±4 standard deviations (SD) around its mean, while holding all other latent dimensions constant. This was the OCT feature most strongly associated with dementia-related gene sets. The central image corresponds to the reference latent vector. Bottom row - difference maps showing the per-pixel change in pixel intensity relative to the reference reconstruction. Red denotes increased intensity, and blue denotes decreased intensity.

Perturbation of this latent dimension appears to affect the thickness of the nasal retinal nerve fibre layer (the bright superior layer of the retina seen to the left side of each OCT) and affects the projection artifacts from retinal blood vessels (note the -4.00SD image casts three vessel shadows nasal [left] of the foveal depression, whilst +4.00SD image casts two). The difference maps suggest that there are also broader changes to pixel intensity distributions across the retina. Reproduced with the permission of UK Biobank.

OCT Embedding 105 Grad-CAM

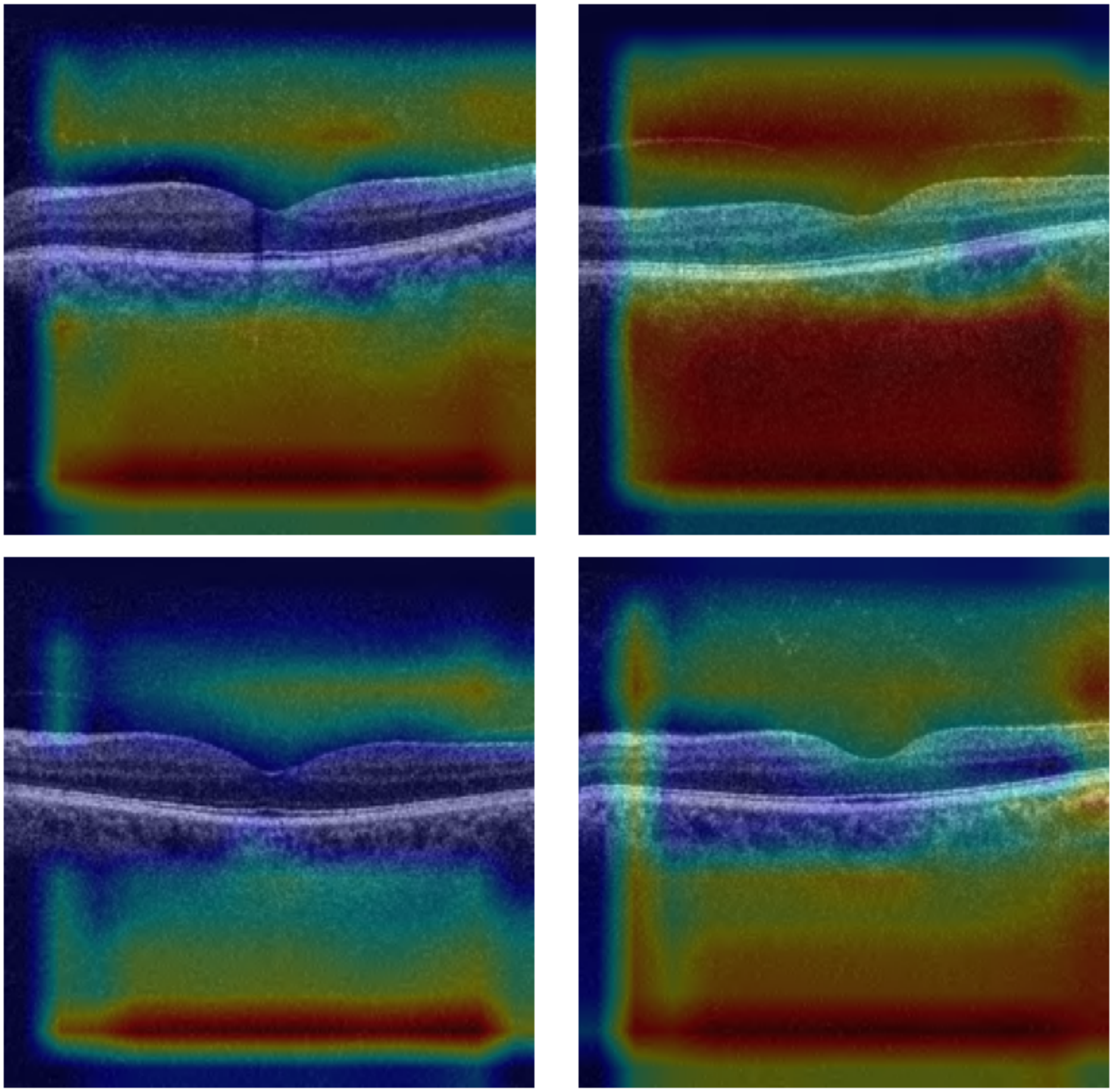

Supplementary Figure 51 Grad-CAM saliency map for OCT embedding 105. This was the OCT feature most strongly associated with future hypertension. As can be seen, the embedding seems to localise to superior and inferior borders of the retina. Reproduced with the permission of UK Biobank.

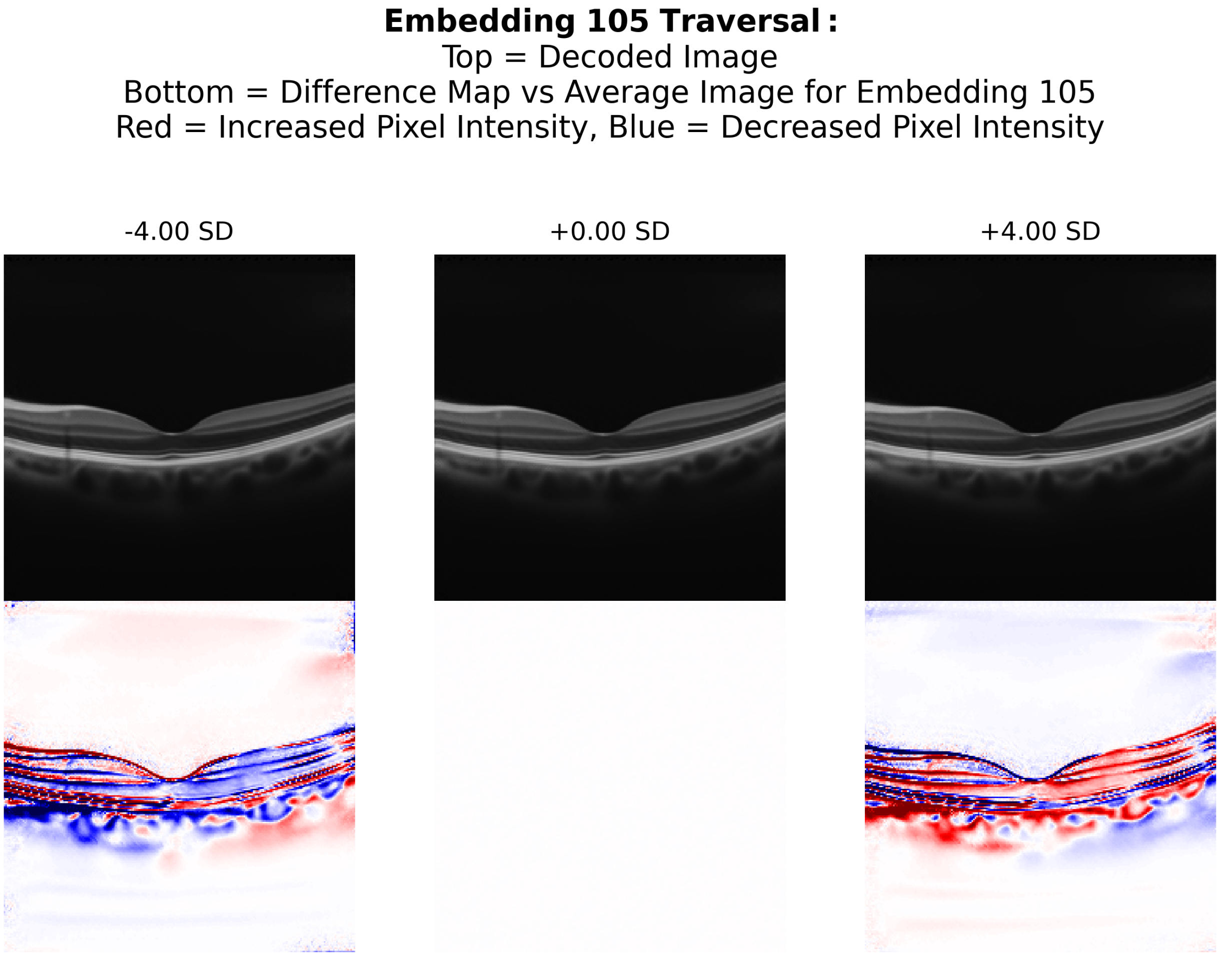

Supplementary Figure 52: Top row - reconstructions obtained by perturbing latent dimension 105 by ±4 standard deviations (SD) around its mean, while holding all other latent dimensions constant. This was the OCT feature most strongly associated with future hypertension. The central image corresponds to the reference latent vector. Bottom row - difference maps showing the per-pixel change in intensity relative to the reference reconstruction. Red denotes increased pixel intensity, and blue denotes decreased intensity.

Perturbation of this latent dimension appears to alter the morphology of the choroidal vessels (the most inferior layer), although the difference maps suggest broader pixel value changes. Reproduced with the permission of UK Biobank.

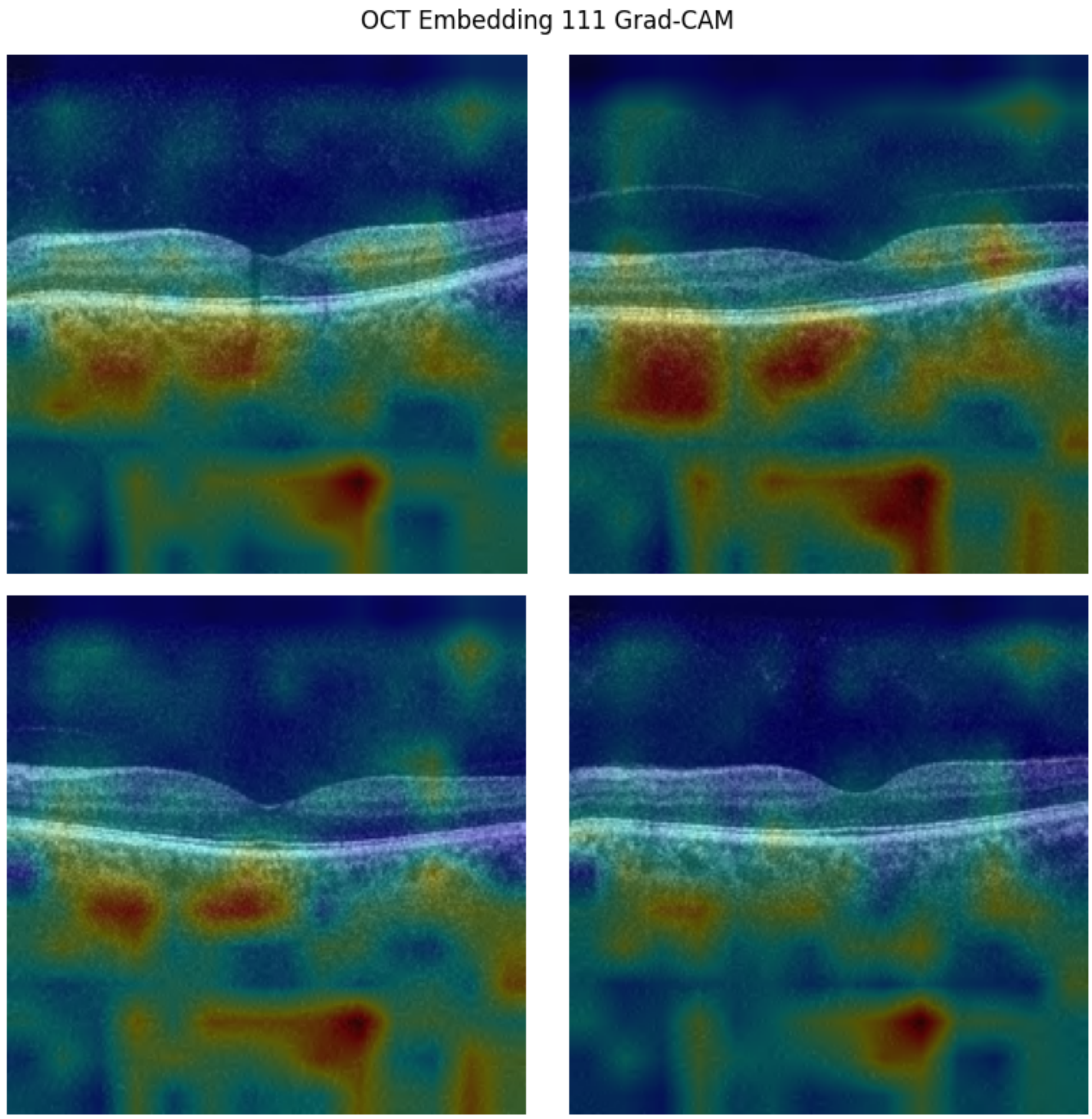

Supplementary Figure 53: Grad-CAM saliency map for OCT embedding 111. This embedding was genetically correlated with heart failure (nominally). Grad-CAM failed to localise the feature, and so Layer-CAM was used. The feature appears to localise to choroid. Reproduced with the permission of UK Biobank.

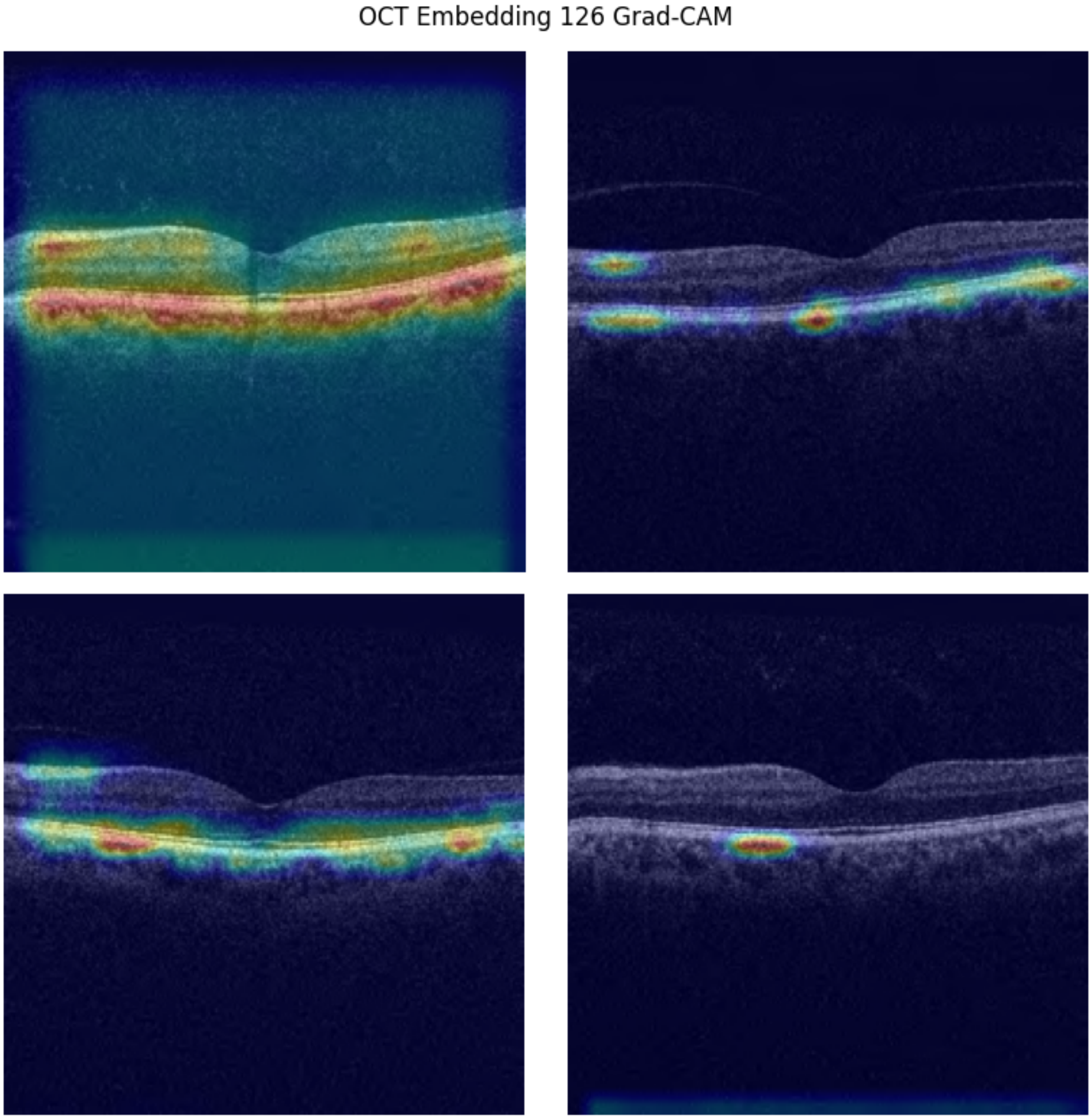

Supplementary Figure 54: Grad-CAM saliency map for OCT embedding 126. This was the OCT feature most strongly associated with the gene set 'KEGG medicus reference regulation of complement cascade *CFHR*'. The third convolutional layer saliency map did not localise clearly, therefore this analysis was performed on the second convolutional layer. The embedding appears to localise to the choroid and retinal pigment epithelium, in keeping with previous work

linking complement to disorders of these structures. Reproduced with the permission of UK Biobank.

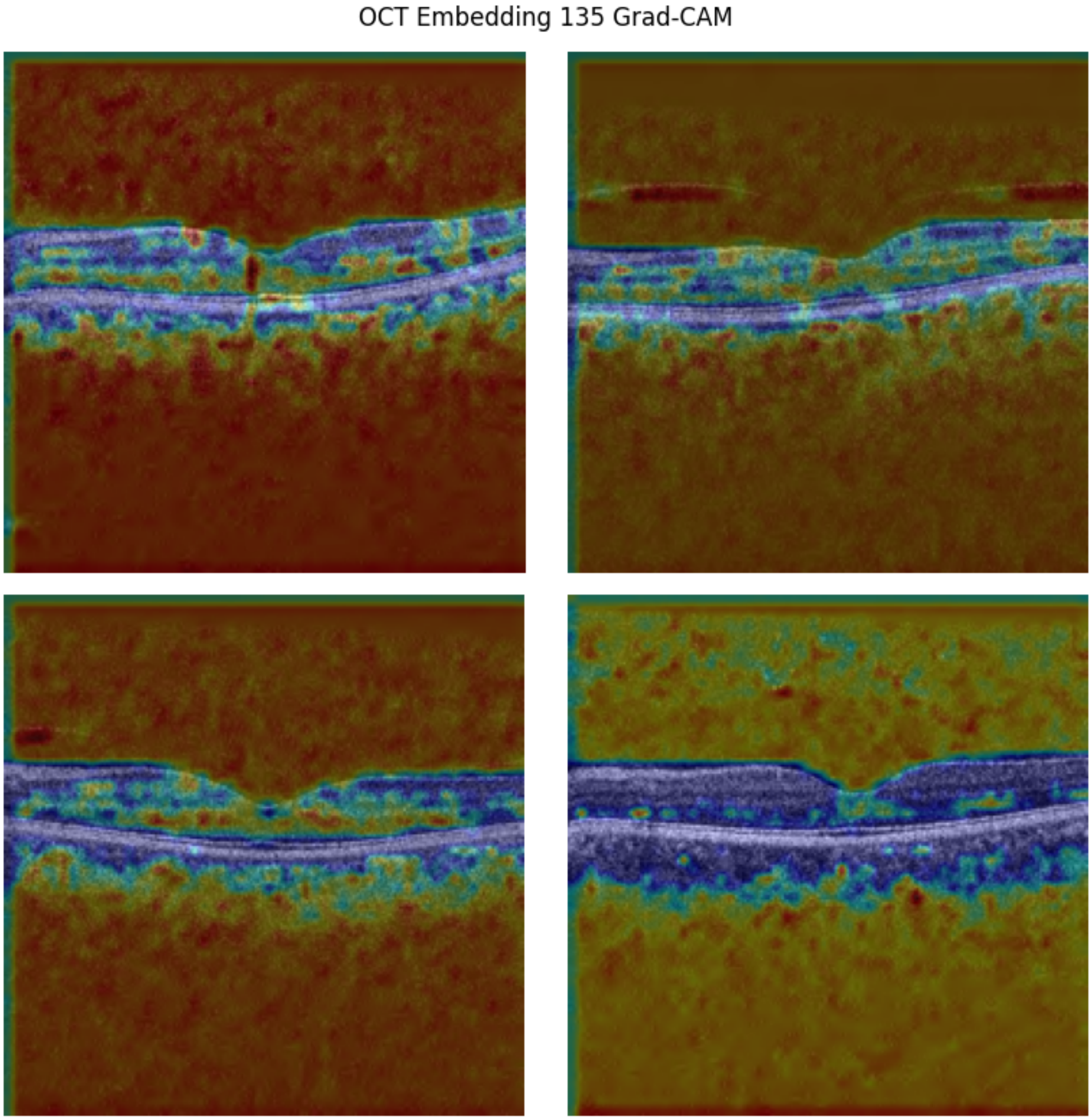

Supplementary Figure 55: Grad-CAM saliency map for OCT embedding 135. This was the OCT feature most strongly associated with the gene set 'Pathway Interaction Database (PID) alpha synuclein pathway'. The third and second convolutional layer saliency maps did not localise to any specific area and therefore this analysis was performed on the first convolutional layer. The embedding appears to loosely localise to the outer retinal layers. Reproduced with the permission of UK Biobank.

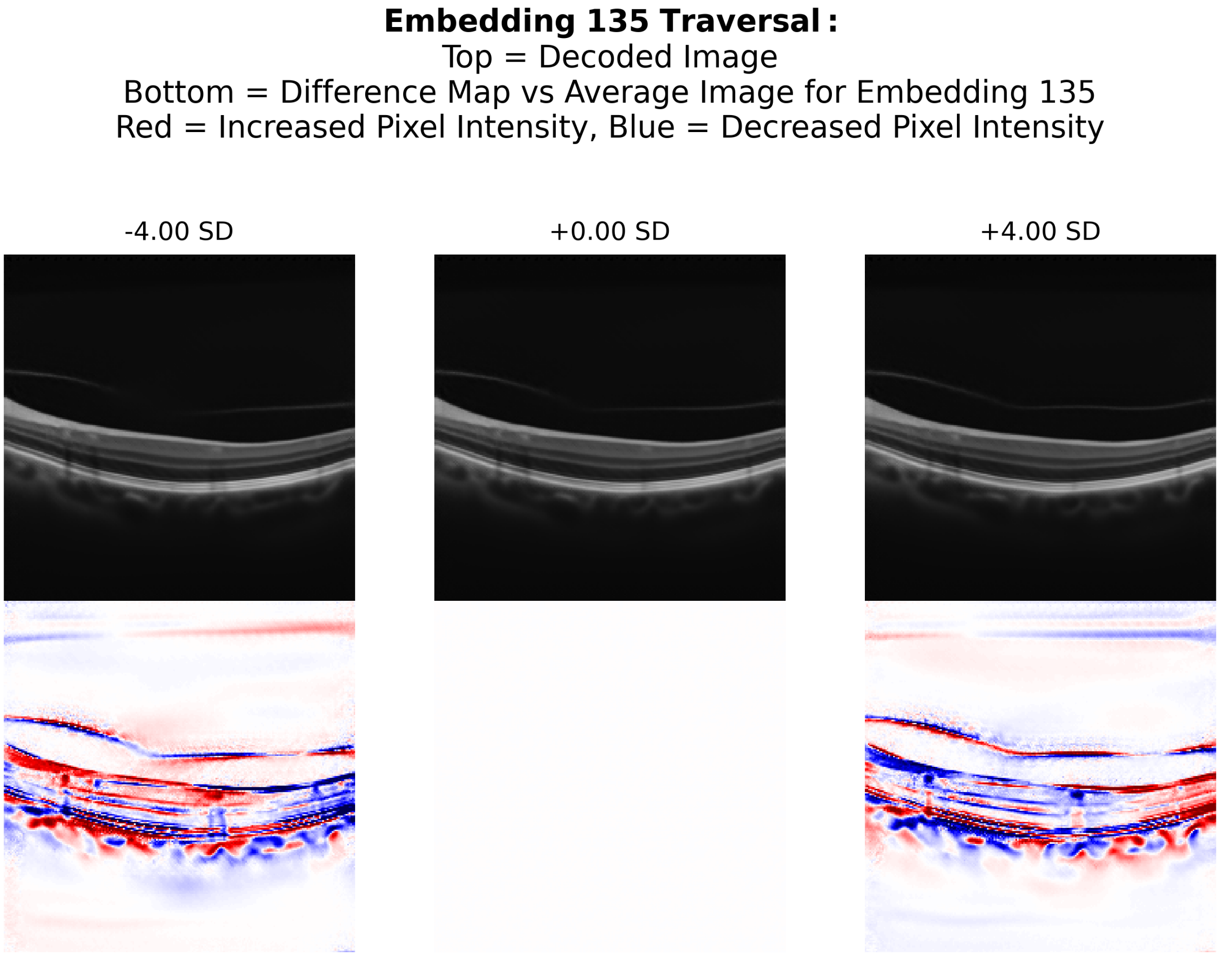

Supplementary Figure 56: Top row - reconstructions obtained by perturbing latent dimension 135 by ±4 standard deviations (SD) around its mean, while holding all other latent dimensions constant. This was the OCT feature most strongly associated with the gene set ‘Pathway Interaction Database (PID) alpha synuclein pathway’. The central image corresponds to the reference latent vector. Bottom row - difference maps showing the per-pixel change in intensity relative to the reference reconstruction. Red denotes increased intensity, and blue denotes decreased intensity.

The effect of perturbation is difficult to visually interpret, other than discontinuity of the vitreous face at -4.00SD (the superior-most layer sitting above the retina, representing a posterior vitreous detachment). Reproduced with the permission of UK Biobank.

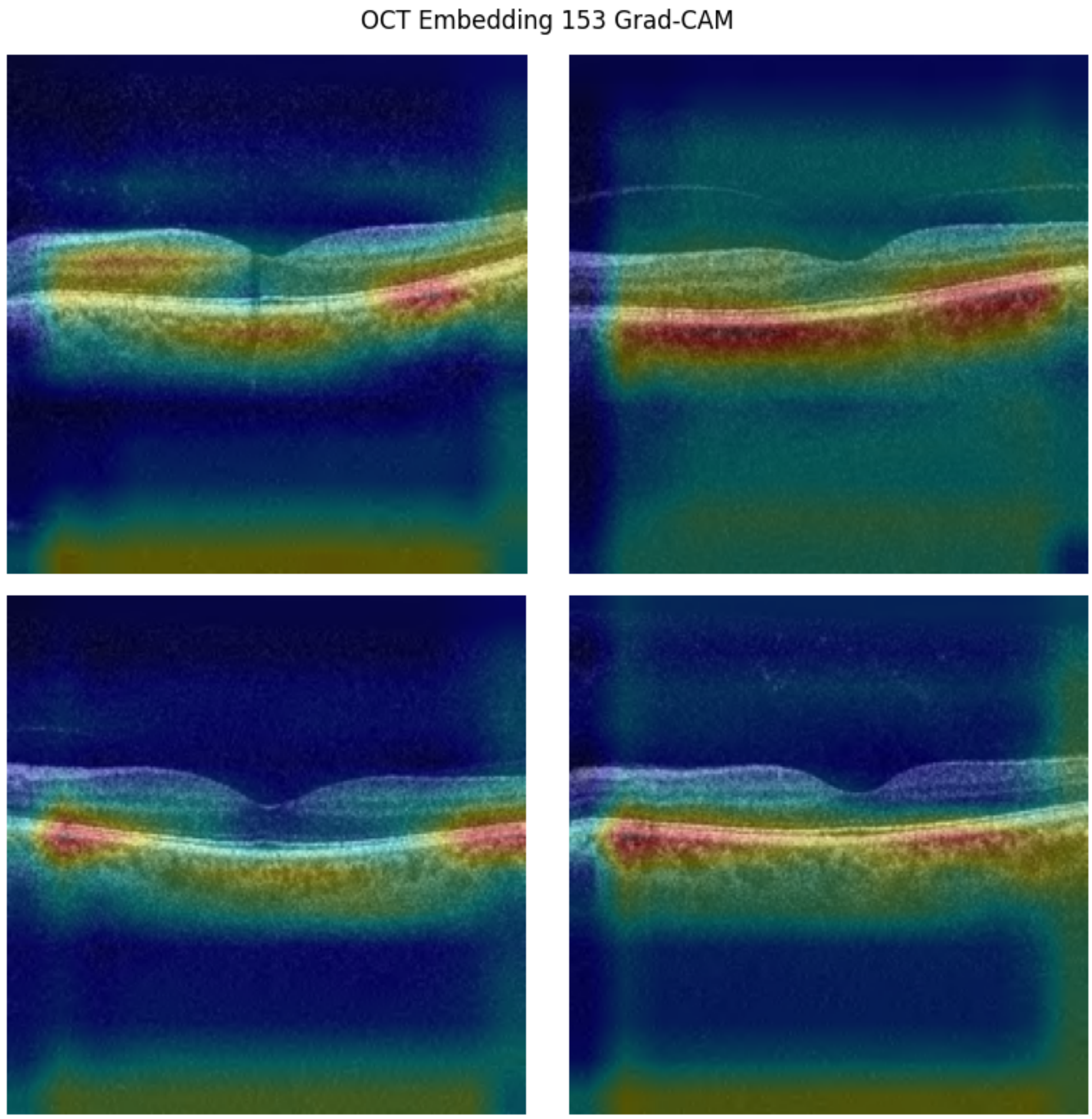

Supplementary Figure 57: Grad-CAM saliency map for OCT embedding 153. This was the OCT feature most strongly associated with the gene set 'KEGG medicus variant duplication or mutation activated *FLT3* to Jak-STAT signaling pathway'. The embedding appears to localise to the choroid. Reproduced with the permission of UK Biobank.

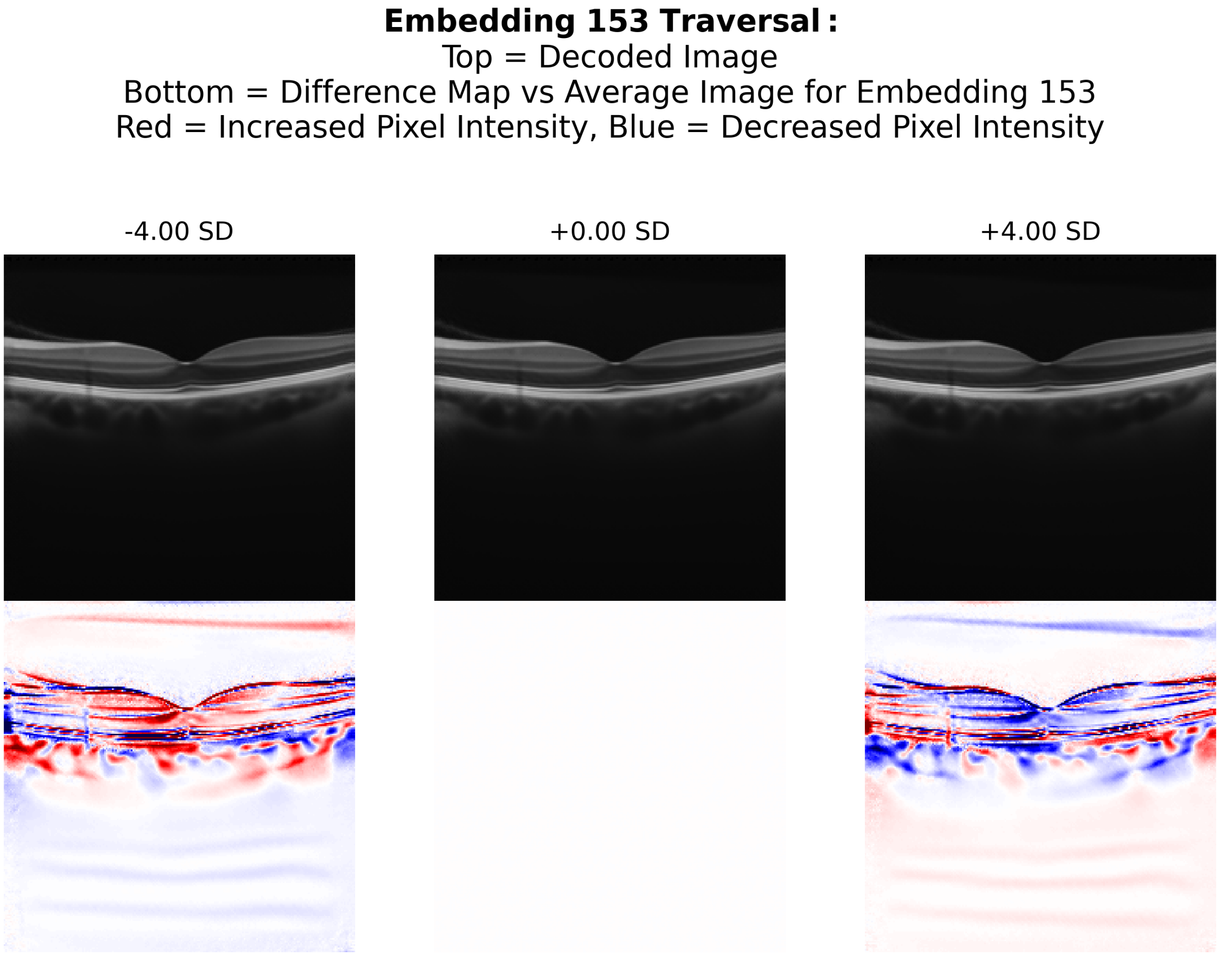

Supplementary Figure 58: Top row - reconstructions obtained by perturbing latent dimension 153 by ±4 standard deviations (SD) around its mean, while holding all other latent dimensions constant. This was the OCT feature most strongly associated with the gene set 'KEGG medicus variant duplication or mutation activated *FLT3* to Jak-STAT signaling pathway'. The central image corresponds to the reference latent vector. Bottom row - difference maps showing the per-pixel change in intensity relative to the reference reconstruction. Red denotes increased intensity, and blue denotes decreased intensity.

It is noted that the choroidal vessels are more well defined at -4.00SD than +4.00SD, and that there are subtle changes to pixel intensity across the image. Reproduced with the permission of UK Biobank.

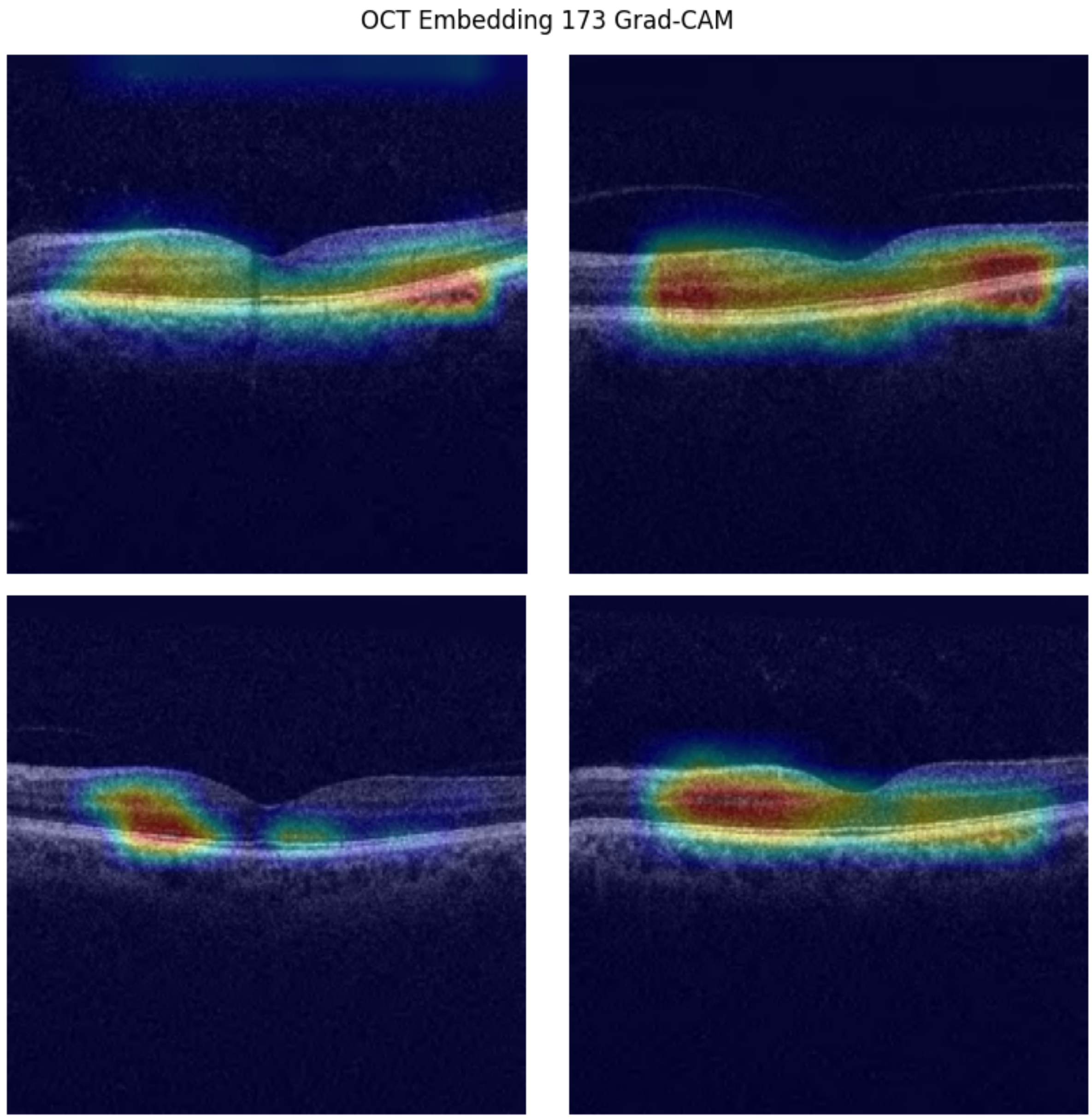

Supplementary Figure 59: Grad-CAM saliency map for OCT embedding 173. This was the embedding most strongly associated with acute myocardial infarction at the time of imaging (baseline). The embedding appears to localise to the neurosensory retina. Reproduced with the permission of UK Biobank.

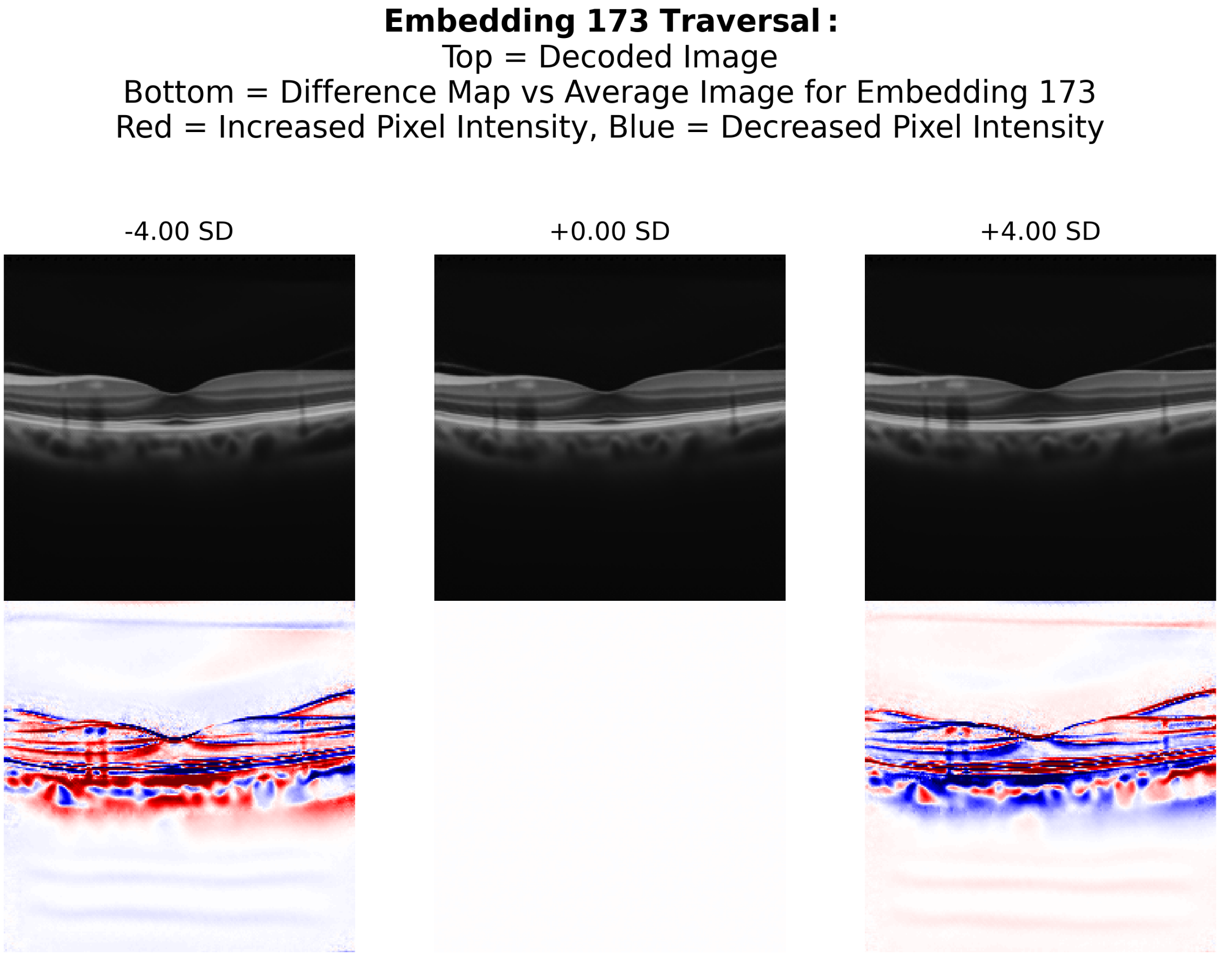

Supplementary Figure 60: Top row - reconstructions obtained by perturbing latent dimension 173 by ±4 standard deviations (SD) around its mean, while holding all other latent dimensions constant. This was the embedding most strongly associated with acute myocardial infarction at the time of imaging (baseline). The central image corresponds to the reference latent vector. Bottom row - difference maps showing the per-pixel change in intensity relative to the reference reconstruction. Red denotes increased pixel intensity, and blue denotes decreased intensity.

Perturbation of this latent dimension appears affect the pixel intensity at several layers, but the visually clear changes are reduction in the size of the ‘foveal bulge’ (the dome shared protrusion of the photoreceptor outer segments at the fovea) from -4.00 to +4.00SD, changes to the width of vessel projection artifacts nasally (left of the fovea on each OCT), and increased pixel intensity in brighter layers (e.g. the vitreous face and retinal nerve fibre layer) with increasing embedding value. Reproduced with the permission of UK Biobank.

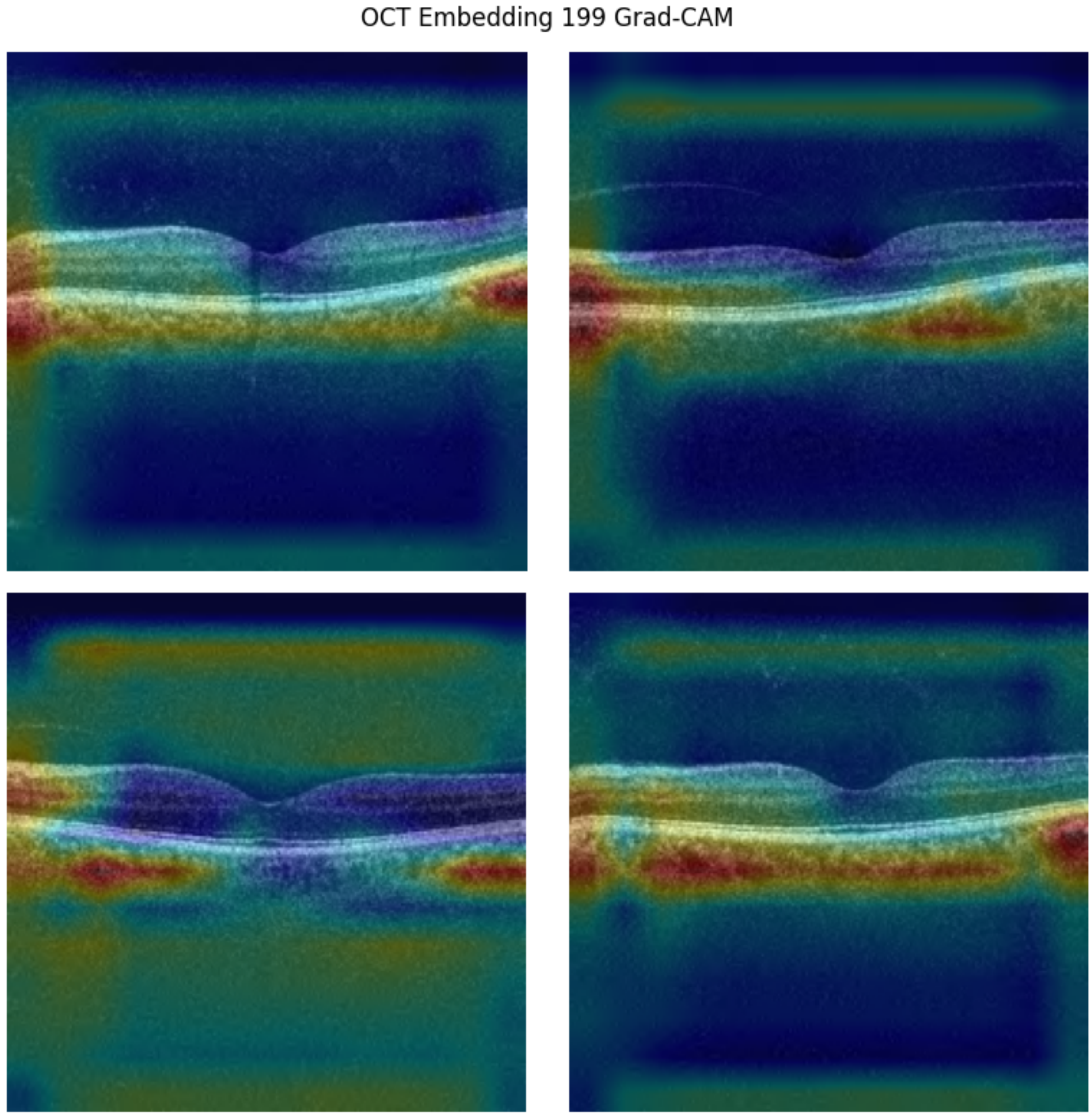

Supplementary Figure 61 caption follows.

Supplementary Figure 61: Grad-CAM saliency map for OCT embedding 199. This embedding was associated with many cardiovascular features in our Pearson correlation analysis. The feature appears to localise to the choroid. Reproduced with the permission of UK Biobank.

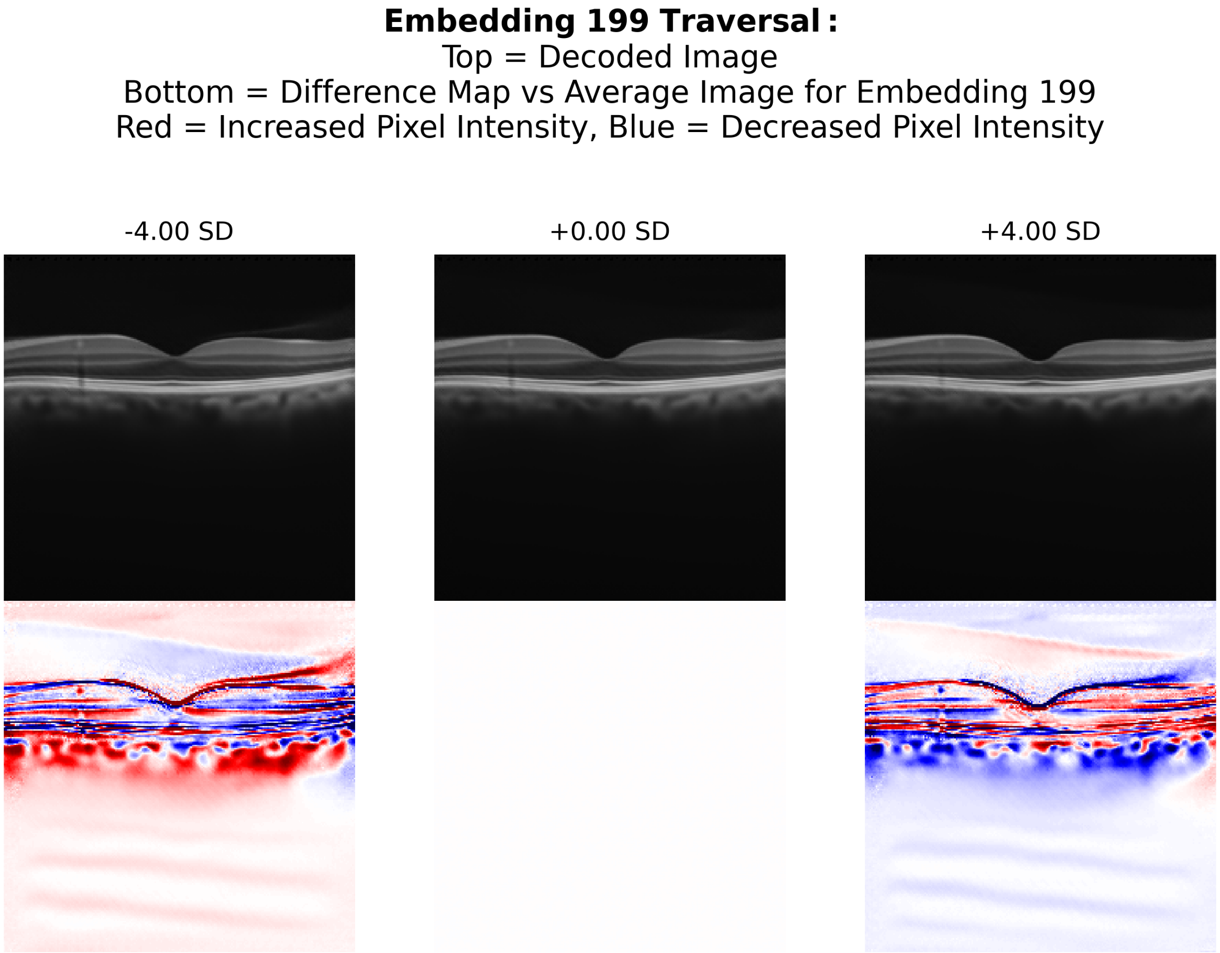

Supplementary Figure 62: Top row - reconstructions obtained by perturbing latent dimension 199 by ±4 standard deviations (SD) around its mean, while holding all other latent dimensions constant. This embedding was associated with many cardiovascular features in our Pearson correlation analysis. The central image corresponds to the reference latent vector. Bottom row - difference maps showing the per-pixel change in intensity relative to the reference reconstruction. Red denotes increased pixel intensity, and blue denotes decreased intensity.

The difference maps suggest there are pronounced changes to pixel intensity at the choroid with perturbation of this embedding, and visually the fovea becomes broader and more rounded at the pit base from 4.00SD to +4.00SD. Reproduced with the permission of UK Biobank.

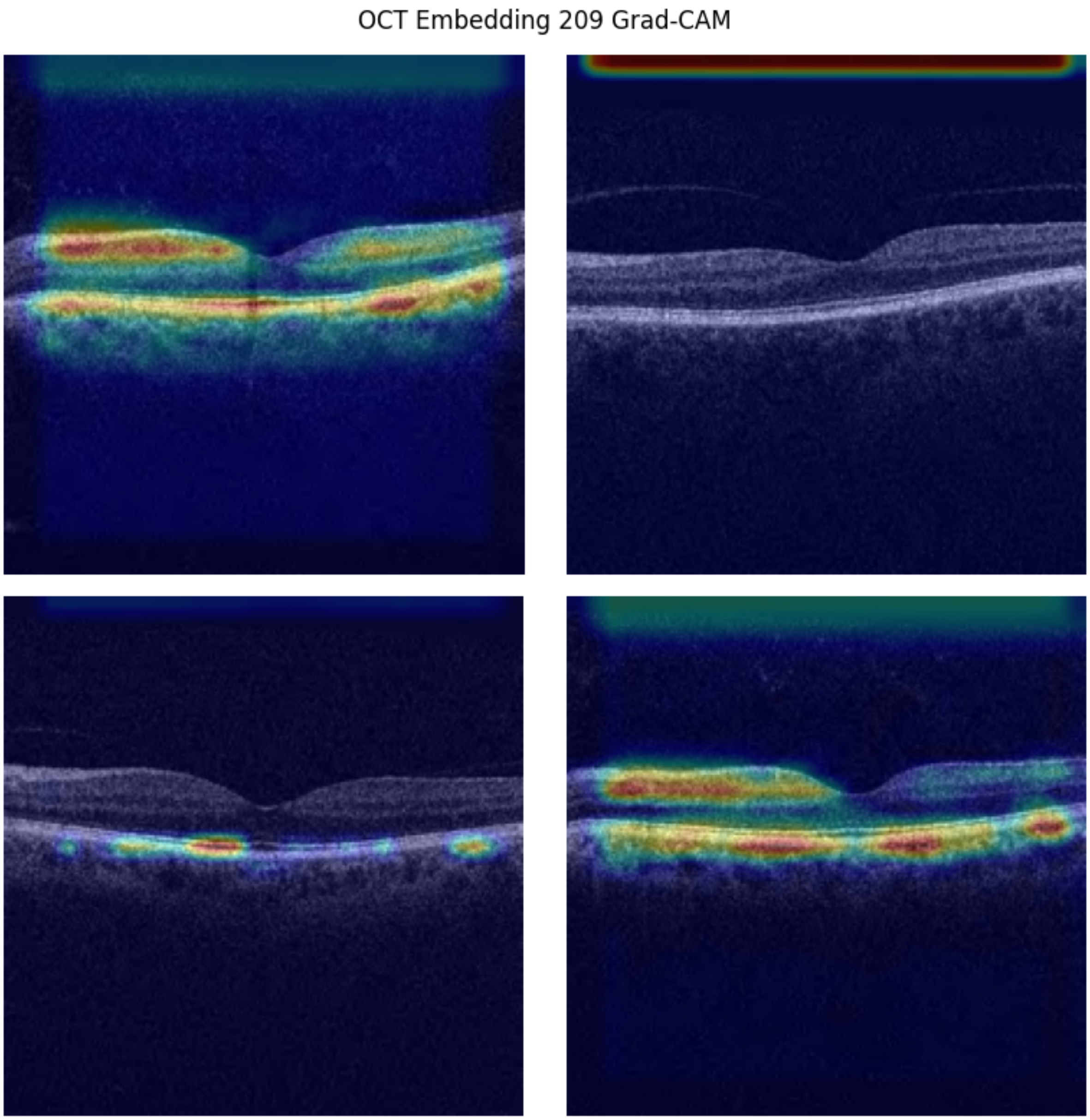

Supplementary Figure 63: Grad-CAM saliency map for OCT embedding 209. This was the OCT feature most strongly associated with baseline hypertension, angina, and chronic ischaemic heart disease, and future heart failure. The embedding appears to localise to ellipsoid zone, the retinal pigmental epithelium, retinal nerve fibre layer, the ganglion cell layer, and to some extent the choroid. Reproduced with the permission of UK Biobank.

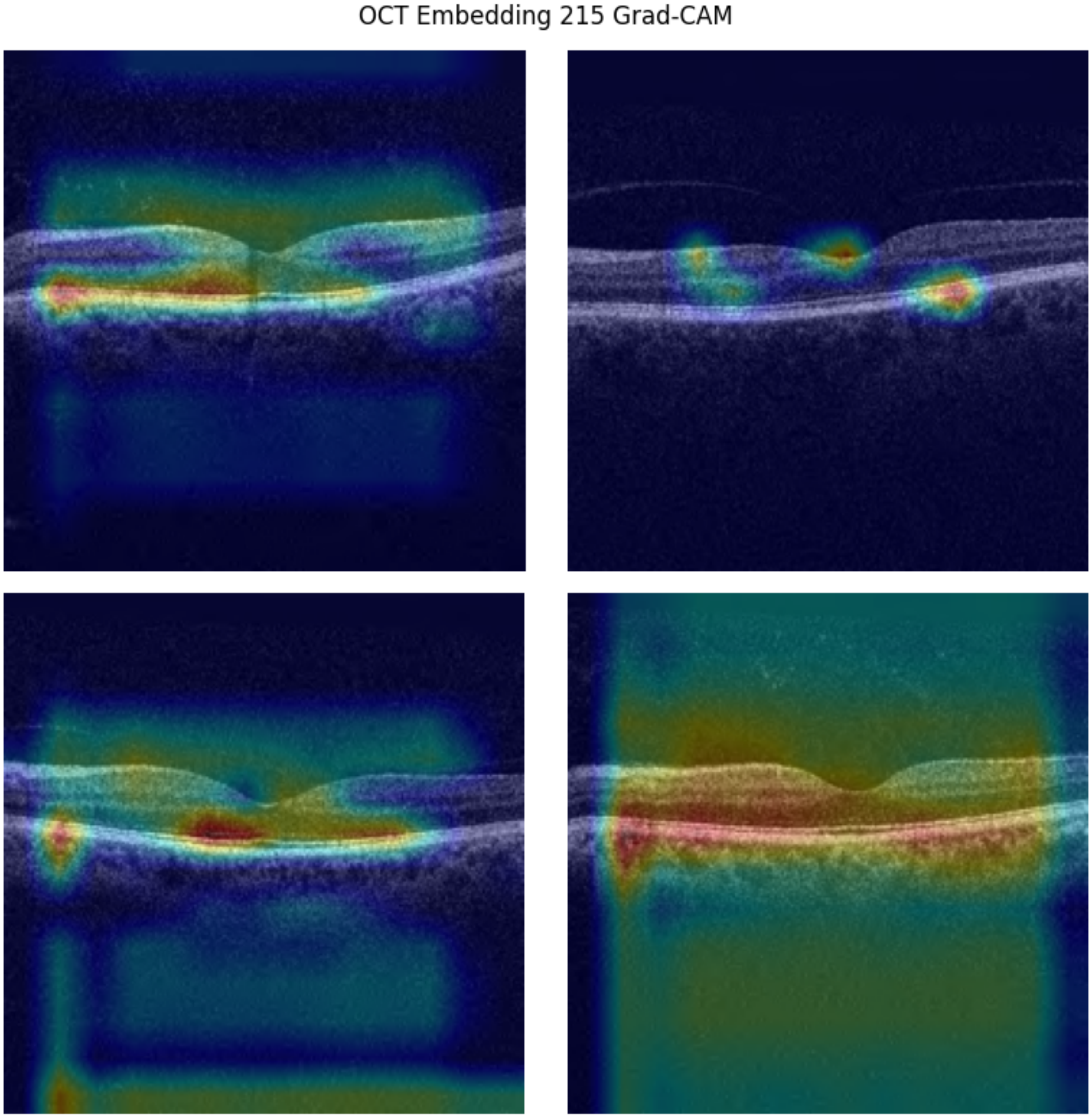

Supplementary Figure 64: Grad-CAM saliency map for OCT embedding 215. This embedding was associated with many cerebral radiomic features in our Pearson correlation analysis. The feature is poorly localised. Reproduced with the permission of UK Biobank.

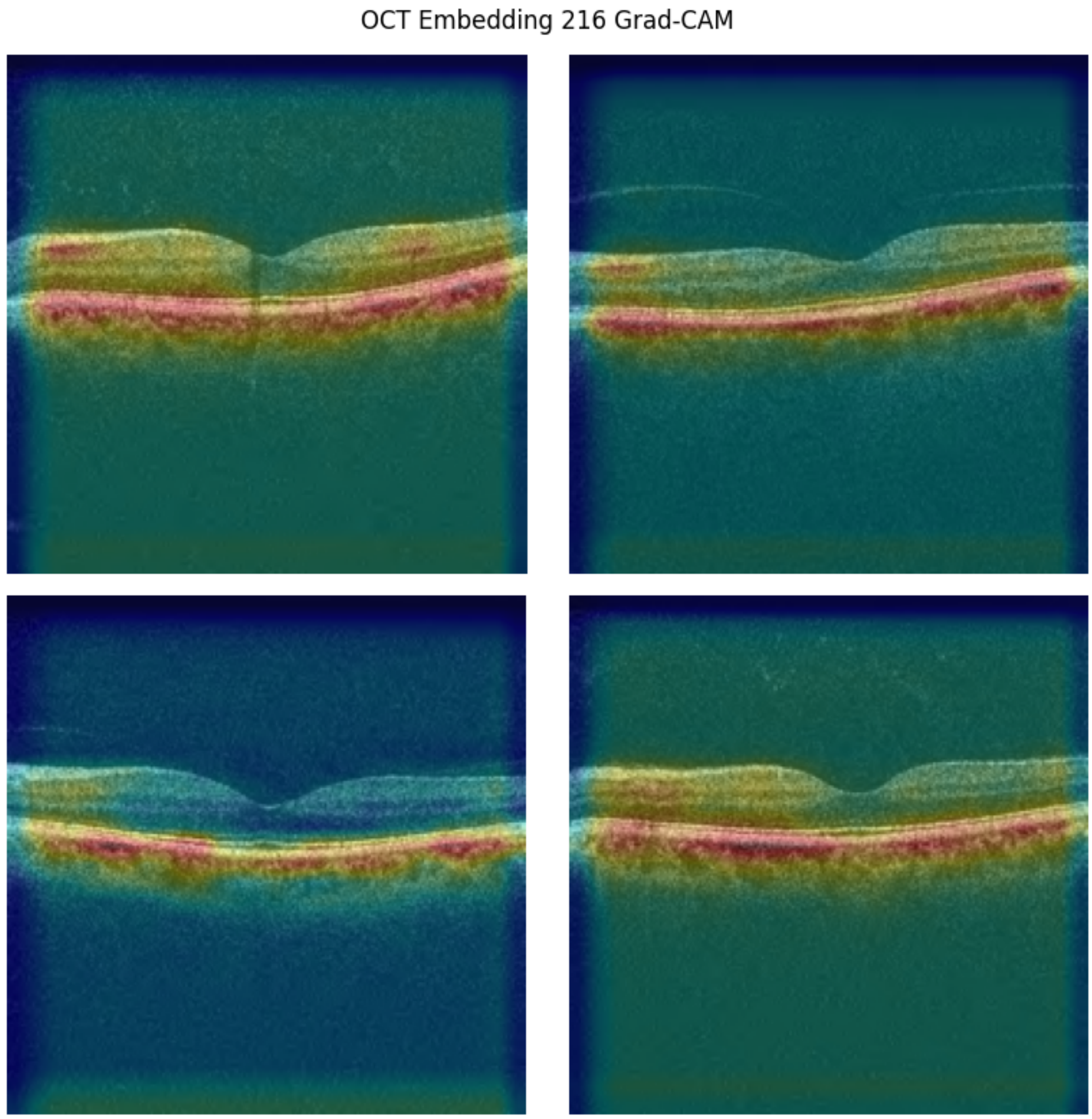

Supplementary Figure 65: Grad-CAM saliency map for OCT embedding 216. This was the leading embedding in the embedding-metabolome CCA analysis (i.e., the strongest weighted in relation to lipid measures). The second convolutional layer was targeted due to poor localisation in the third convolution. The feature appears to localise to the choroid and retinal nerve fibre layer (RNFL). Reproduced with the permission of UK Biobank.

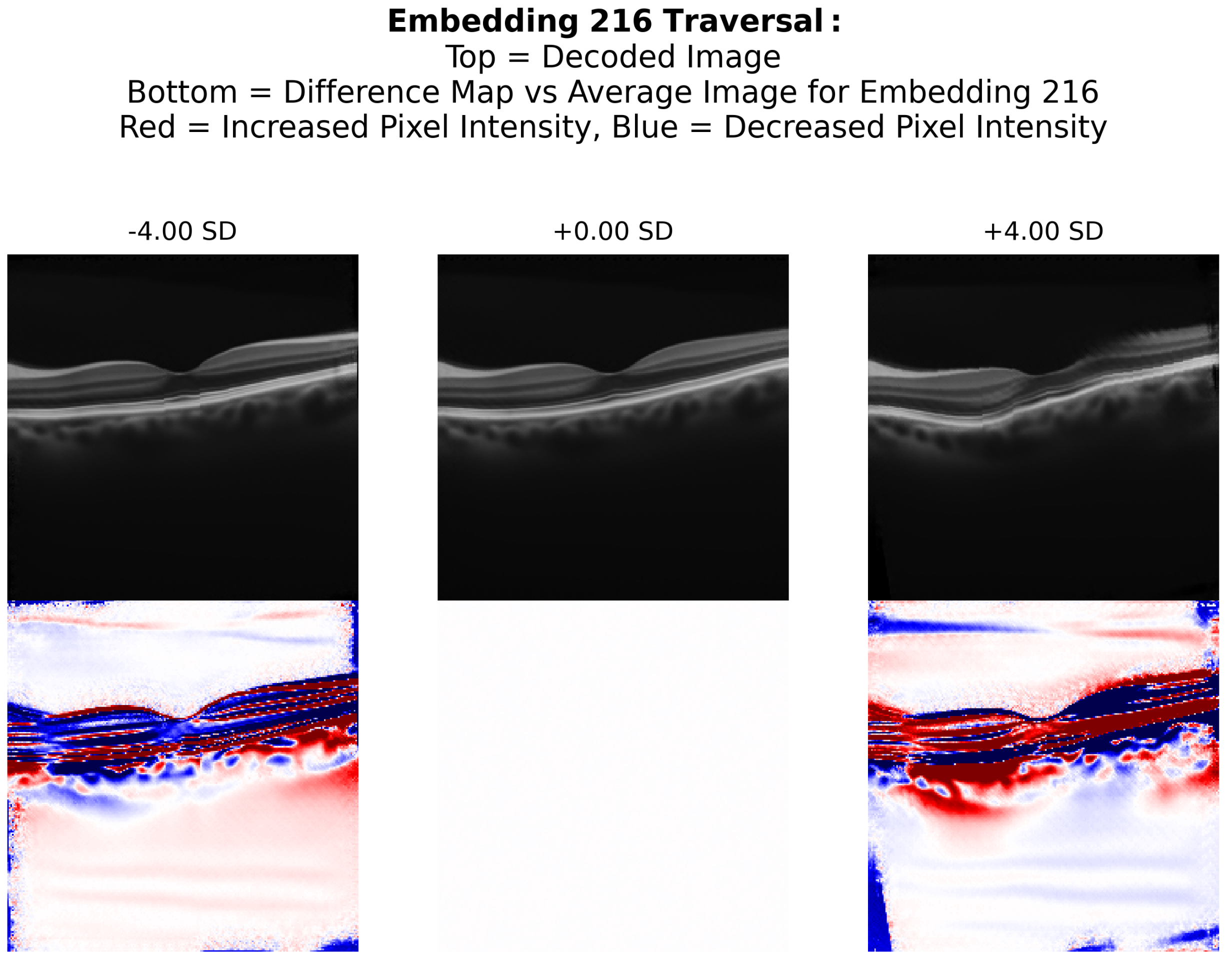

Supplementary Figure 66: Top row - reconstructions obtained by perturbing latent dimension 216 by ±4 standard deviations (SD) around its mean, while holding all other latent dimensions constant. This was the leading embedding in the embedding-metabolome CCA analysis (i.e., the strongest weighted in relation to lipid measures). The central image corresponds to the reference latent vector. Bottom row - difference maps showing the per-pixel change in intensity relative to the reference reconstruction. Red denotes increased pixel intensity, and blue denotes decreased intensity.

Perturbation of this latent dimension appears to affect the thickness of multiple retinal layers when comparing -4.00SD to the reference image (0.00 SD). The +4.00 image appears to be beyond the manifold and is morphologically implausible. Reproduced with the permission of UK Biobank.

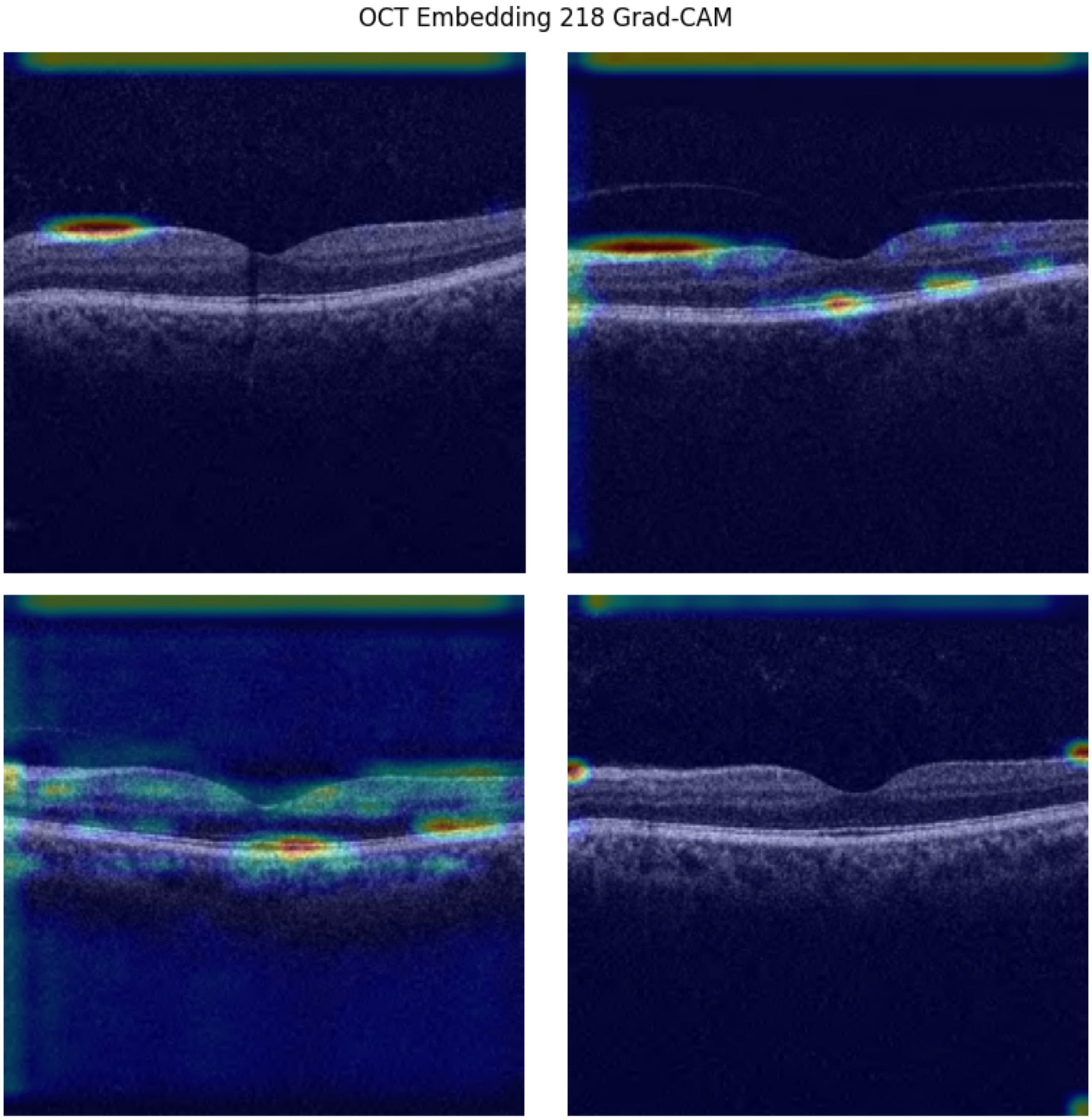

Supplementary Figure 67: Grad-CAM saliency map for OCT embedding 218. This embedding was the most strongly associated with baseline angina, baseline heart failure, future heart failure, and many cerebral radiomic features in our Pearson correlation analysis. The second convolutional layer was targeted due to poor localisation in the third convolution. The embedding appears to localise to the retinal nerve fibre layer. Reproduced with the permission of UK Biobank.

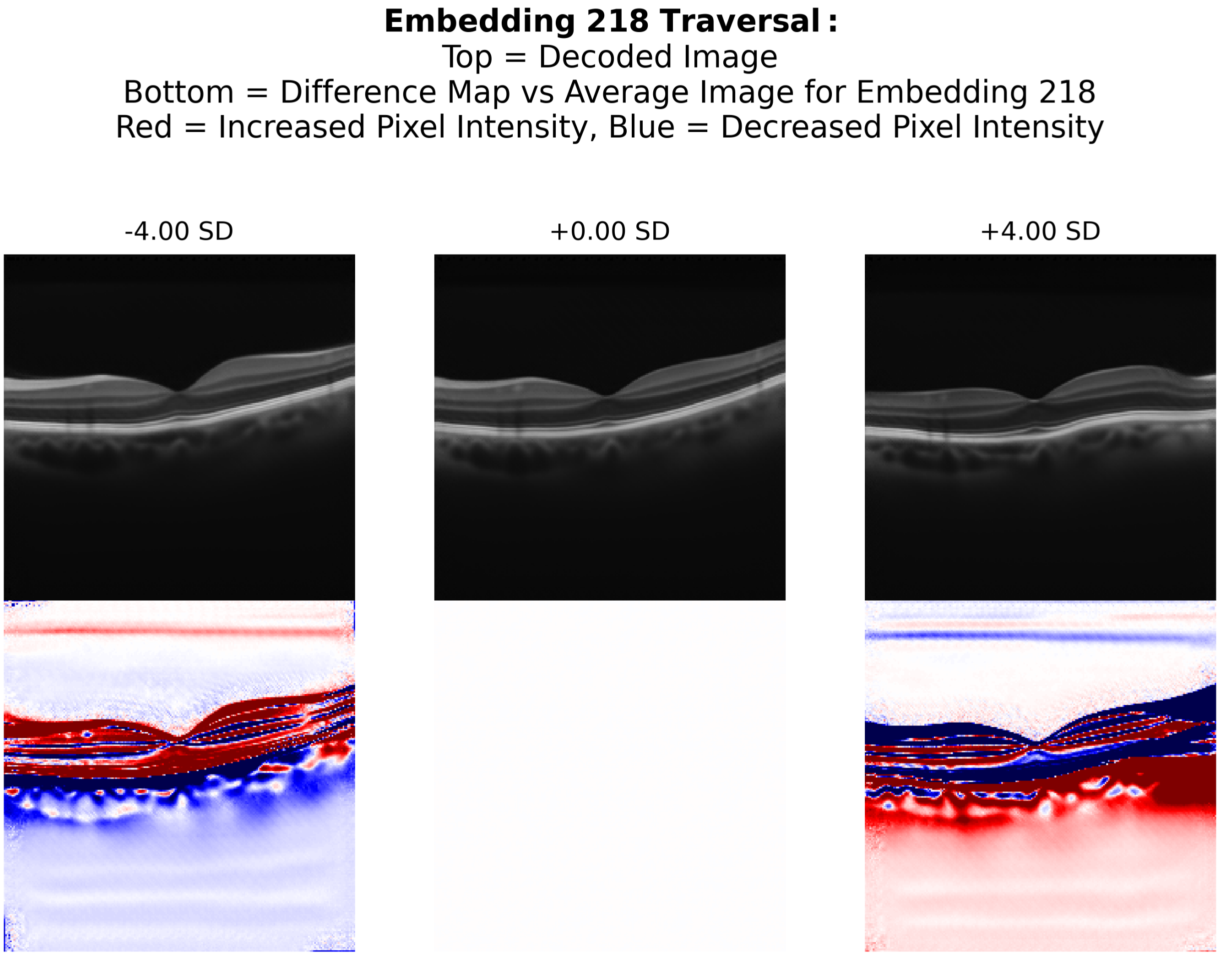

Supplementary Figure 68: Top row - reconstructions obtained by perturbing latent dimension 218 by ±4 standard deviations (SD) around its mean, while holding all other latent dimensions constant. This embedding was the most strongly associated with baseline angina, baseline heart failure, future heart failure, and many cerebral radiomic features in our Pearson correlation analysis. The central image corresponds to the reference latent vector. Bottom row - difference maps showing the per-pixel change in intensity relative to the reference reconstruction. Red denotes increased pixel intensity, and blue denotes decreased intensity.

Perturbation of this latent dimension affects the appearance of multiple retinal layers both in terms of thickness and intensity, the difference maps suggest the pixel intensity changes throughout the retina. Visually, the clearest changes are to the contour of the retina, the thickness and intensity of the retinal nerve fibre layer and inner nuclear layer, and the intensity of choroidal vessel walls. Reproduced with the permission of UK Biobank.

Cardiovascular Trait Associations

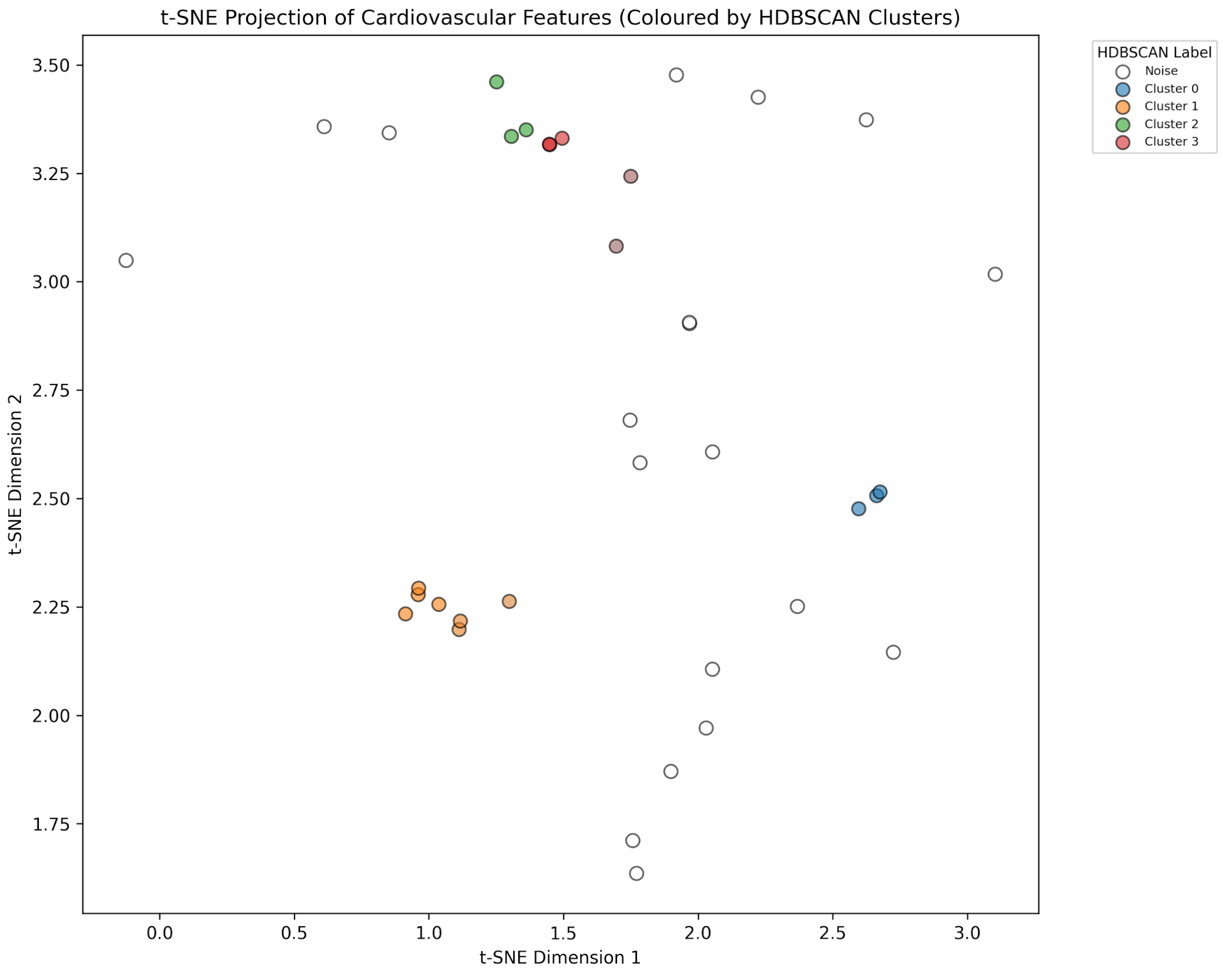

Supplementary Figure 69: A t-SNE projection of cardiovascular features clustered using HDBSCAN. The clusters have been colour coded according to the HDBSCAN cluster

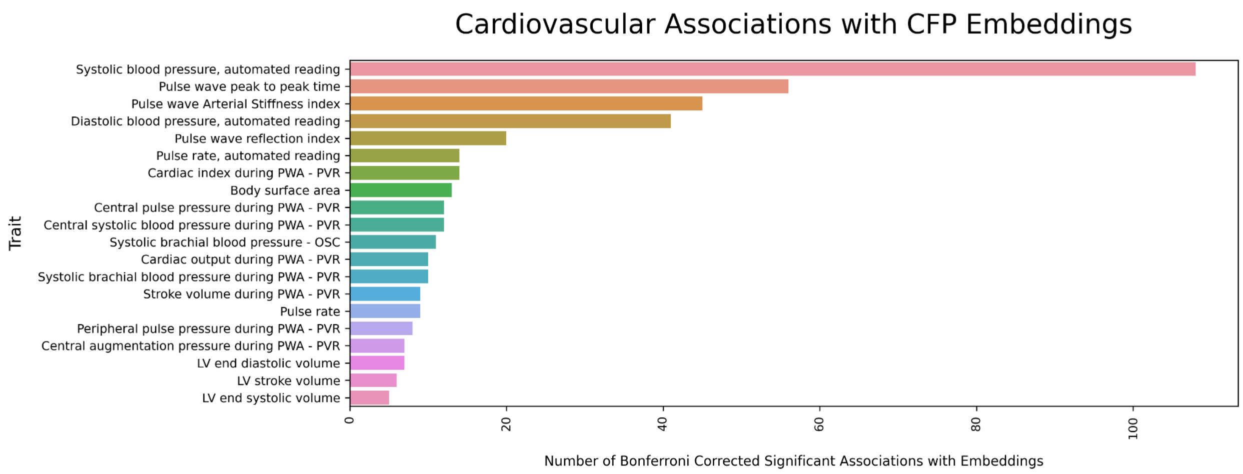

Supplementary Figure 70: A bar plot showing the top 20 cardiovascular traits in terms of the number of multiple testing corrective significant associations with CFP embeddings.

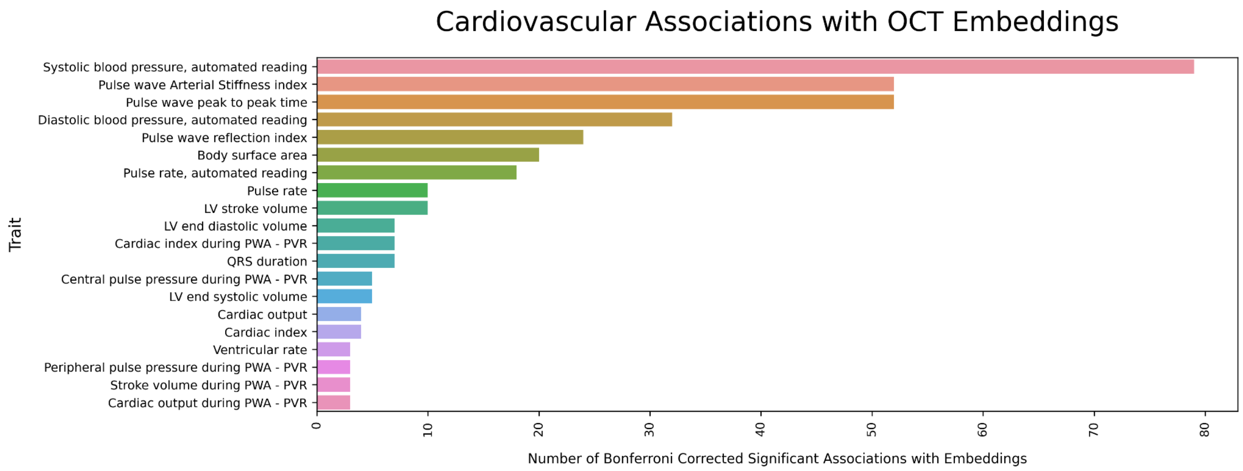

Supplementary Figure 71: A bar plot showing the top 20 cardiovascular traits in terms of the number of multiple testing corrective significant associations with OCT embeddings.

Metabolomic Analyses

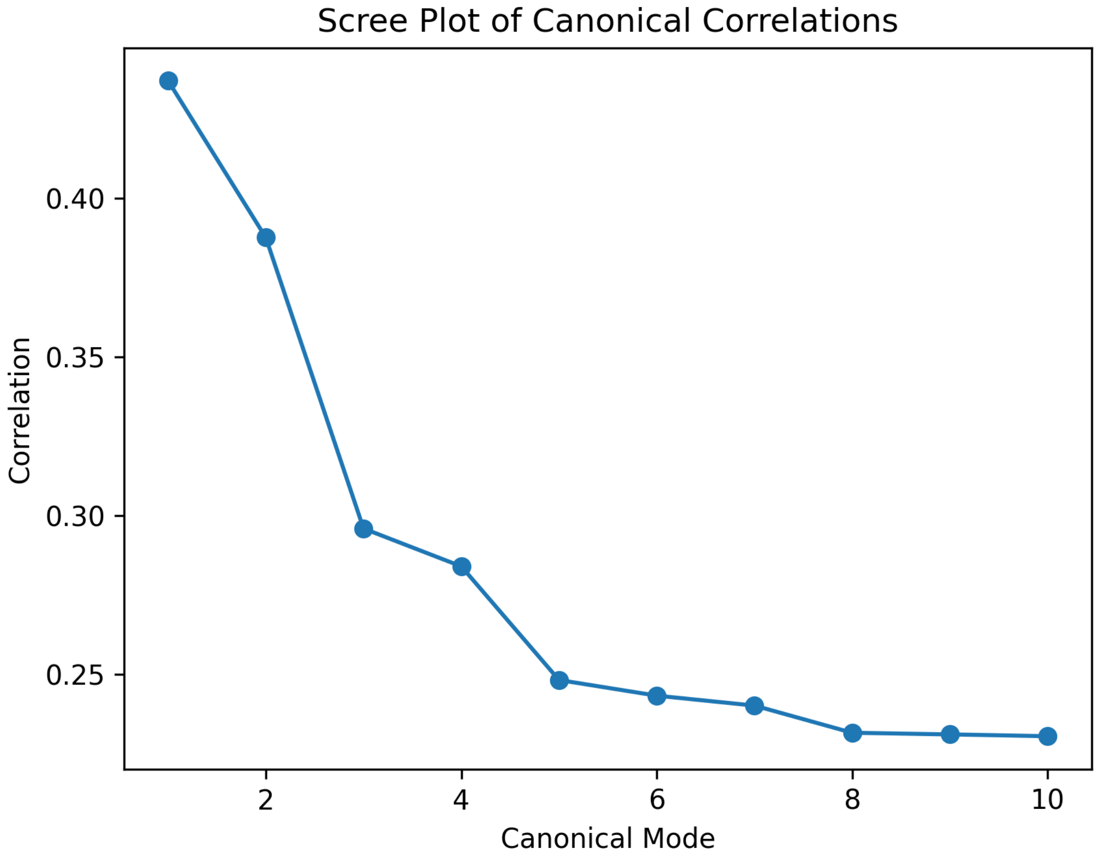

Supplementary Figure 72: A scree plot of the first 10 canonical modes in the CFP canonical correlation analysis.

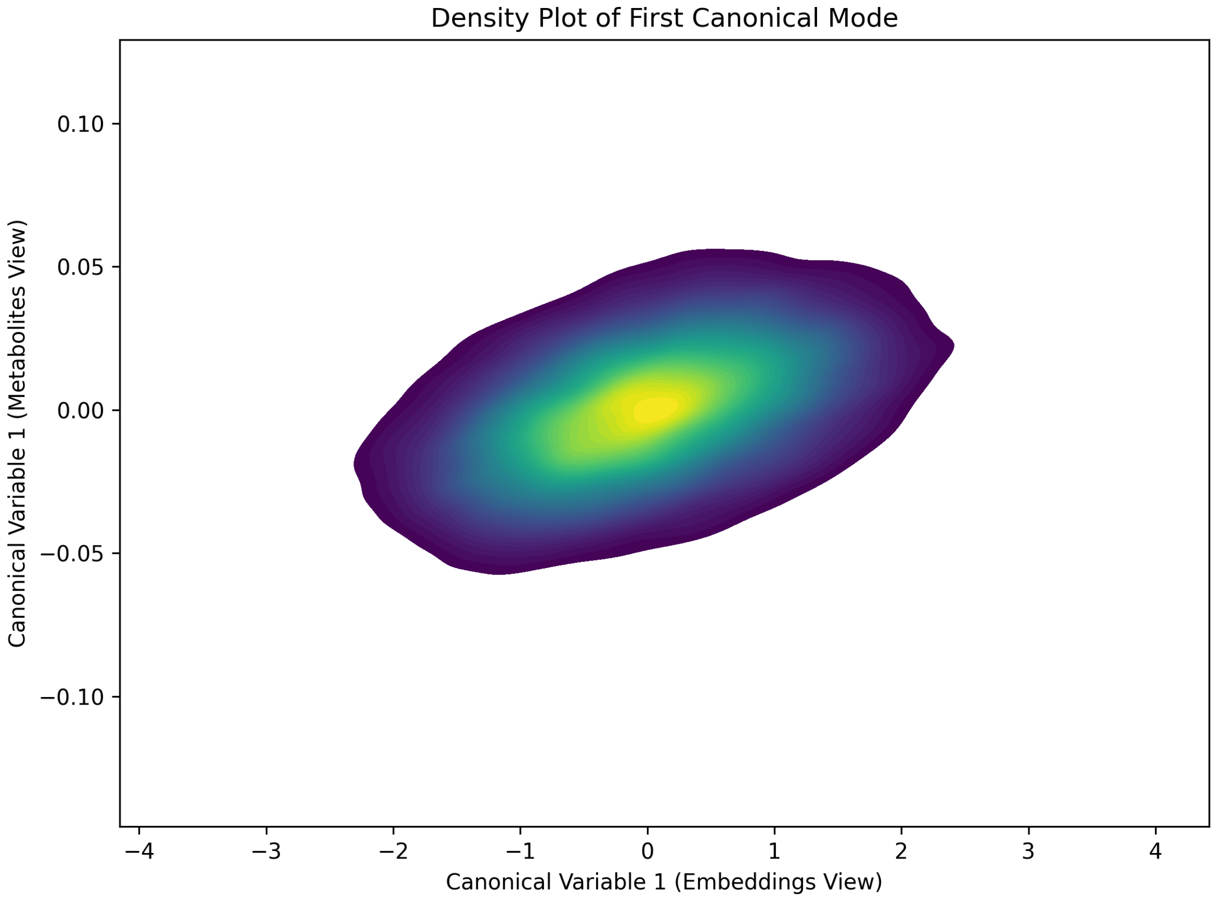

Supplementary Figure 73: A density plot of the first canonical mode in the CFP canonical correlation analysis, which is used to visualise the correlation between the canonical variables.

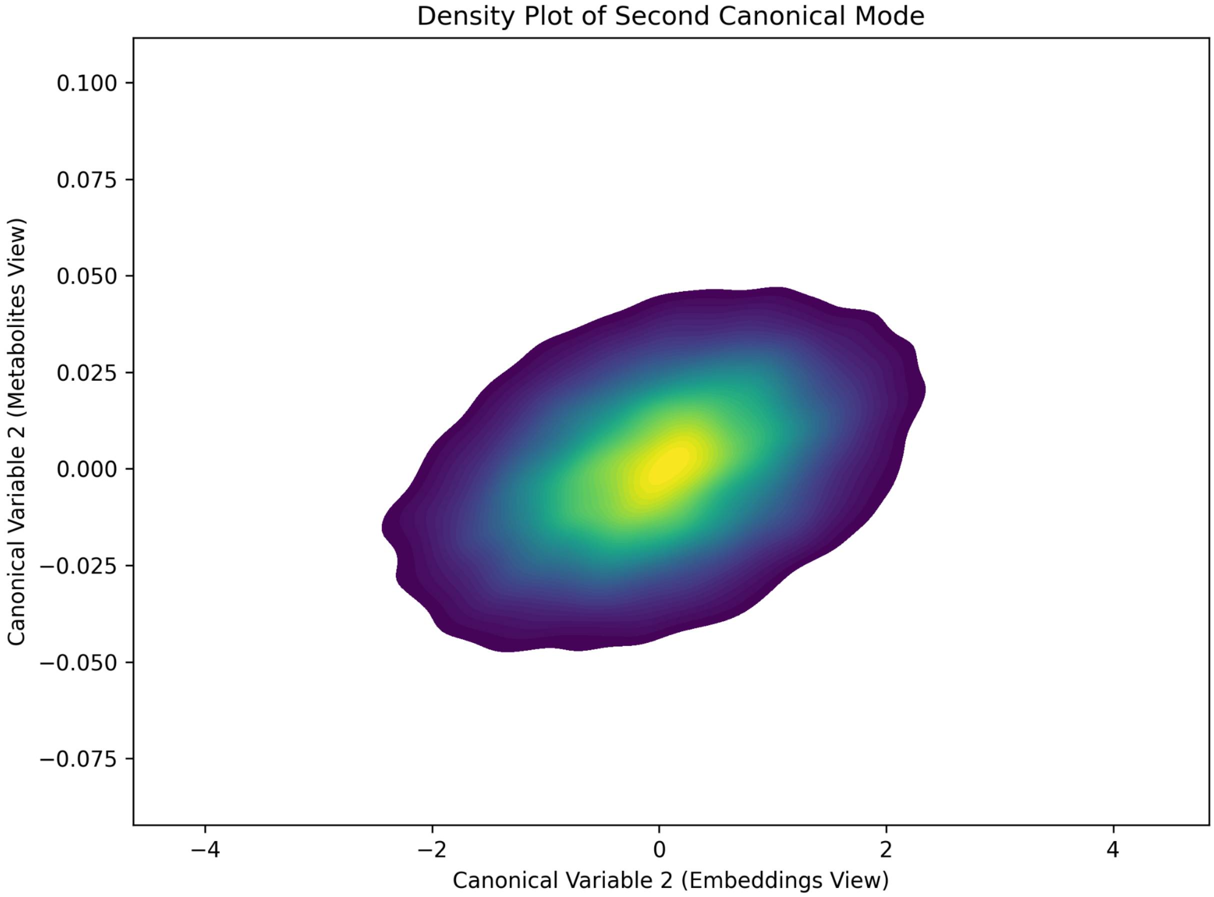

Supplementary Figure 74: A density plot of the second canonical mode in the CFP canonical correlation analysis, which is used to visualise the correlation between the canonical variables.

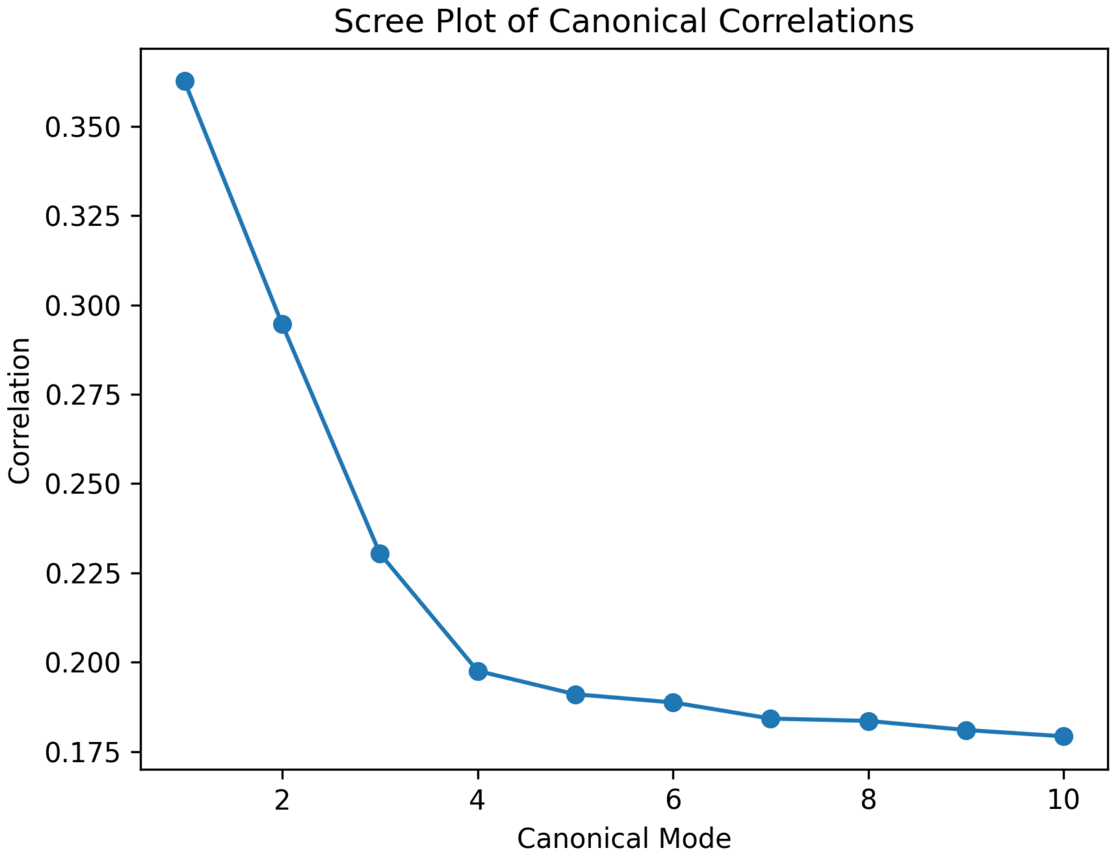

Supplementary Figure 75: A scree plot of the first 10 canonical modes in the OCT canonical correlation analysis.

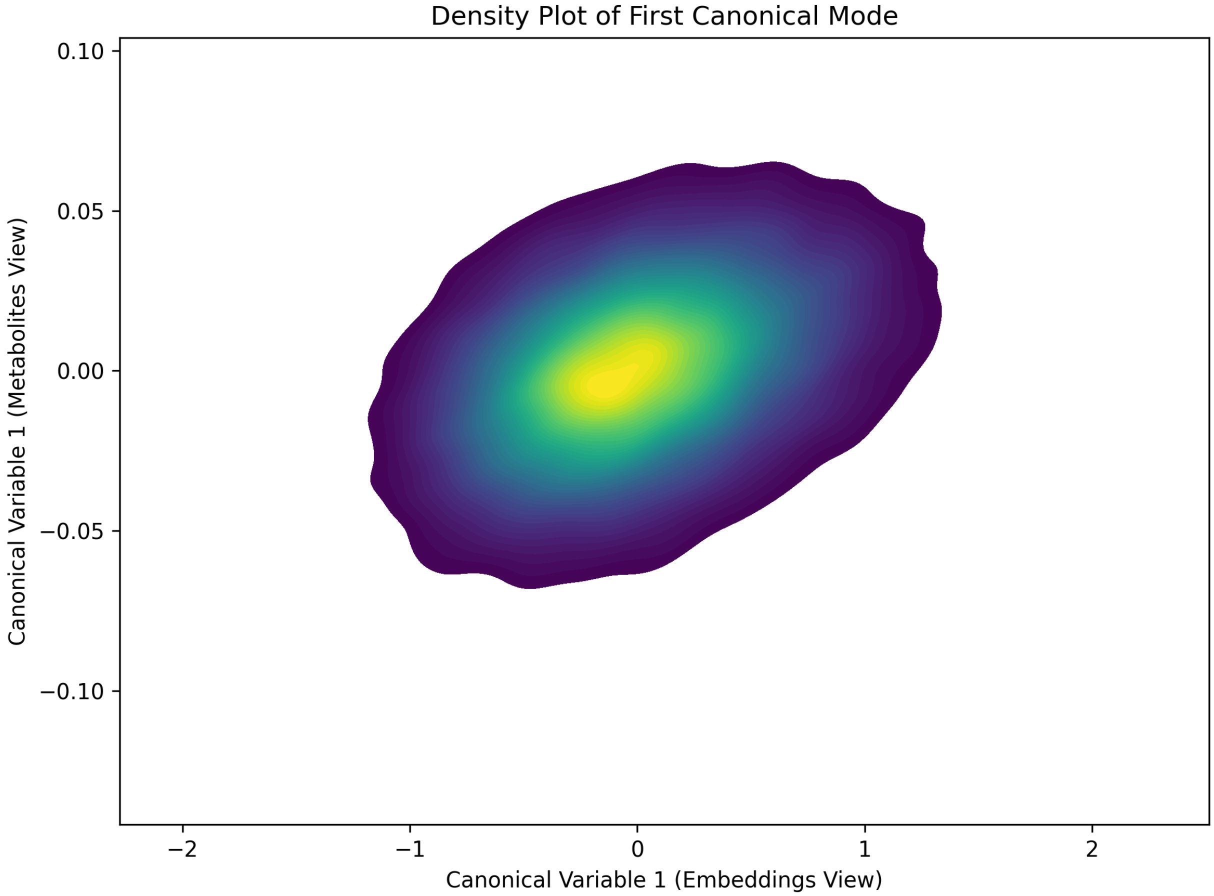

Supplementary Figure 76: A density plot of the second canonical mode in the OCT canonical correlation analysis, which is used to visualise the correlation between the canonical variables.

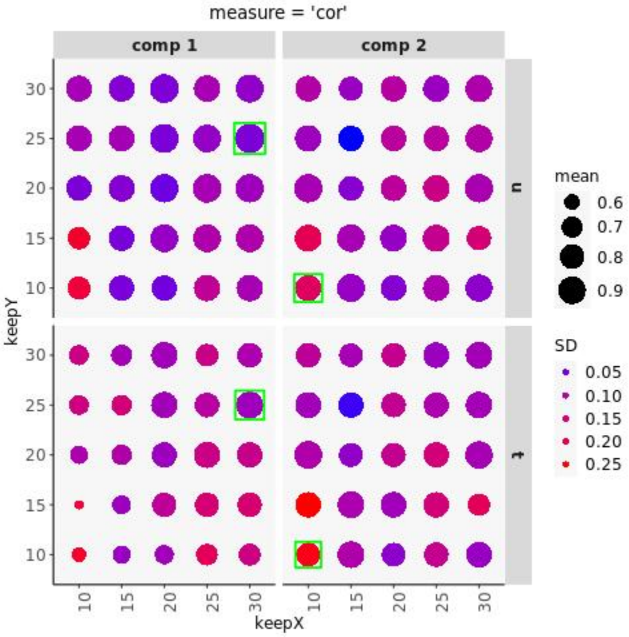

Supplementary Figure 77: The results of the colour fundus photograph – metabolite component number search. The green squares indicate the optimal number of components of to use for metabolites and embeddings in the component 1 and component 2 analysis.

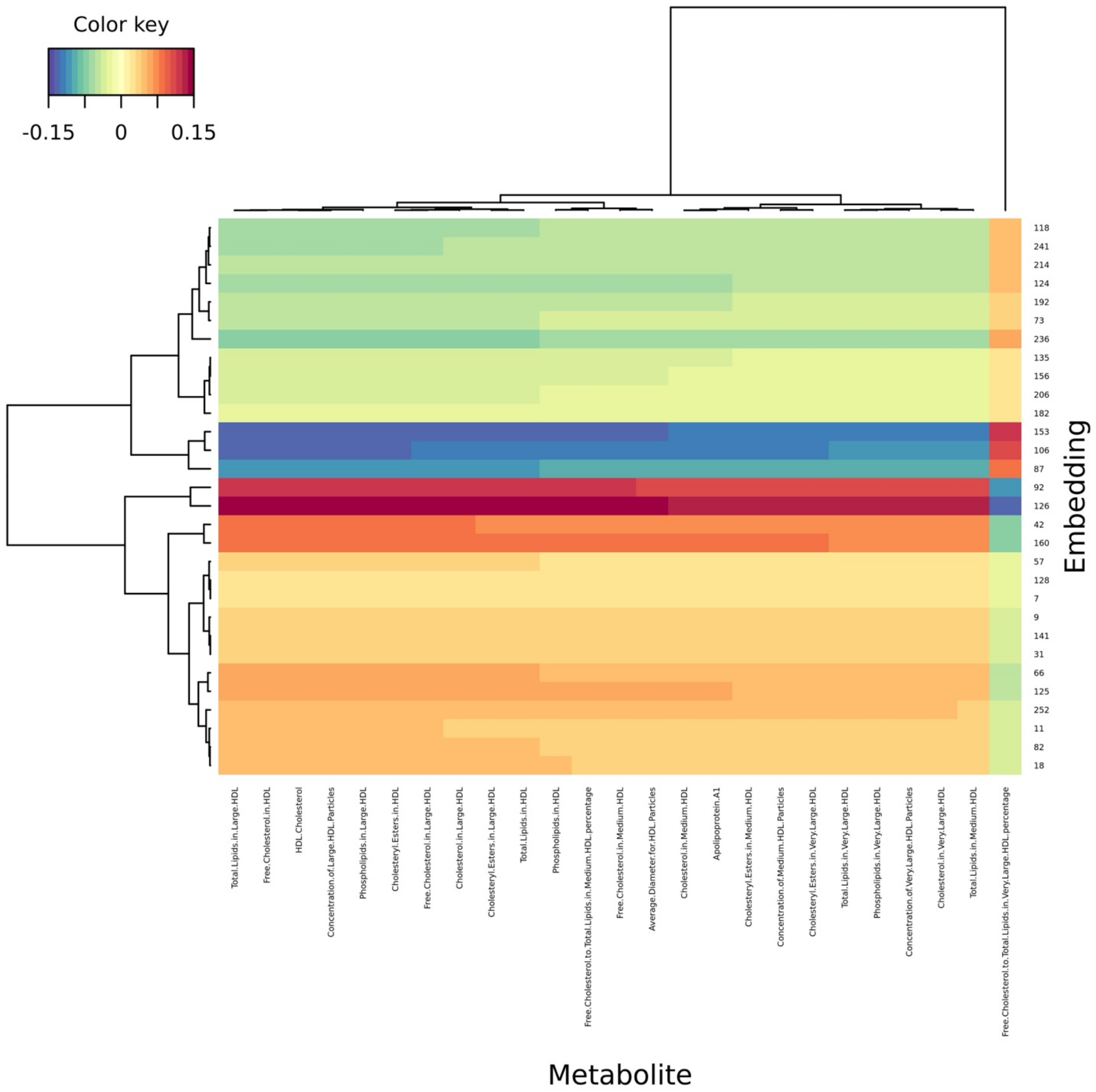

Supplementary Figure 78: A clustered heat map of the colour fundus photograph – metabolite sparse partial least squares analysis (SPLS). The plot features component one of the SPLS analysis and illustrates the correlation between features.

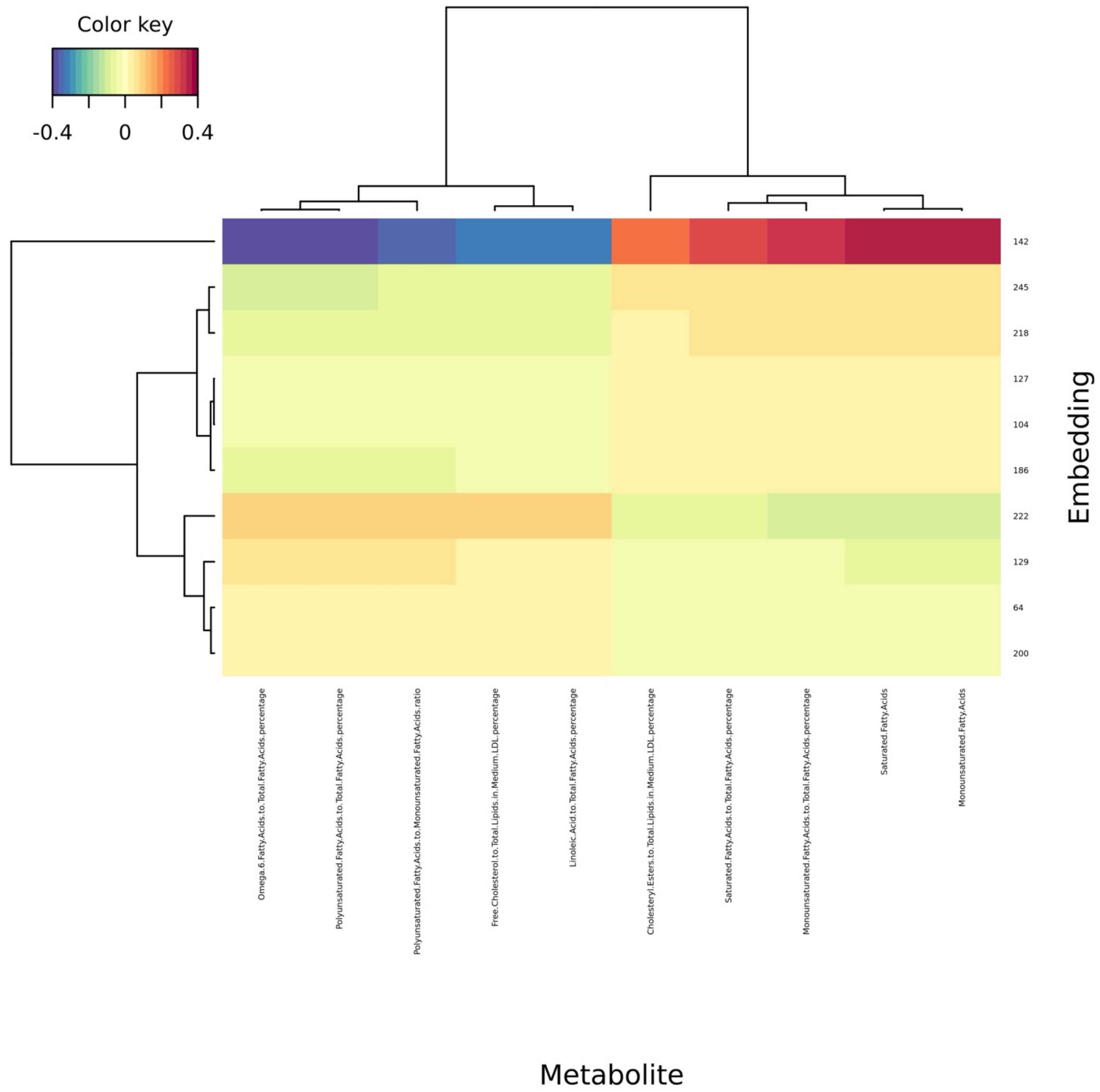

Supplementary Figure 79: A clustered heat map of the colour fundus photograph – metabolite sparse partial least squares analysis (SPLS). The plot features component two of the SPLS

analysis and illustrates the correlation between features.

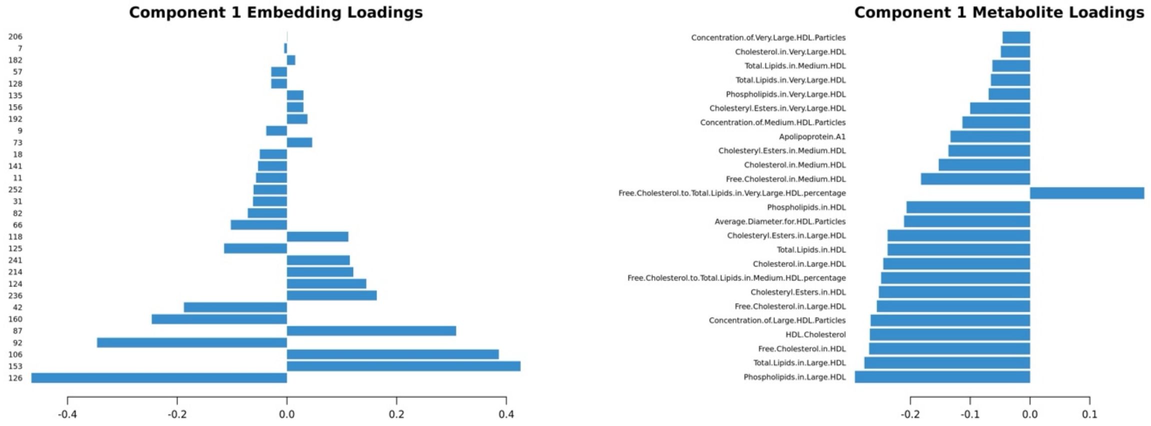

Supplementary Figure 80: A plot of each metabolite/embedding contribution to the latent components of the colour fundus photograph – metabolite sparse partial least squares analysis (SPLS). The plot shows which features drive embedding-metabolite associations in component 1.

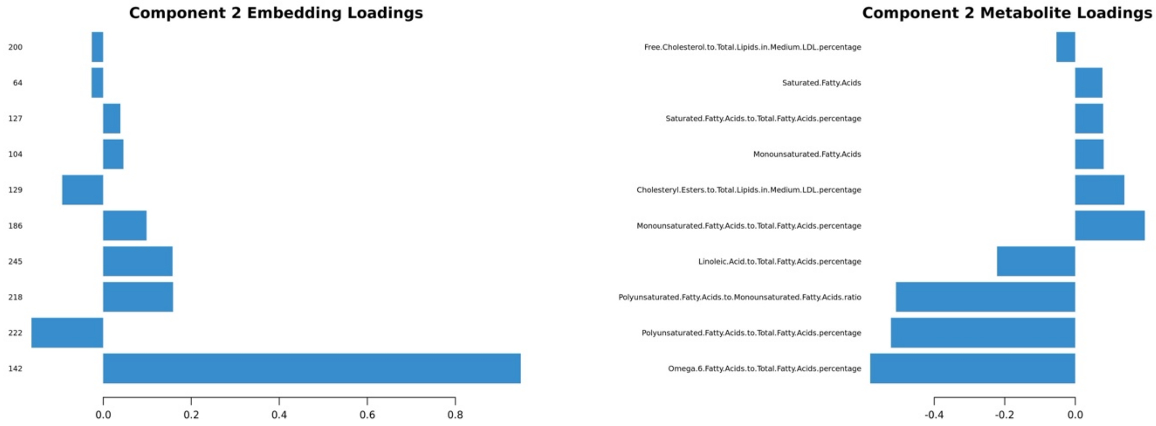

Supplementary Figure 81: A plot of each metabolite/embedding contribution to the latent components of the colour fundus photograph – metabolite sparse partial least squares analysis (SPLS). The plot shows which features drive embedding-metabolite associations in component 1.

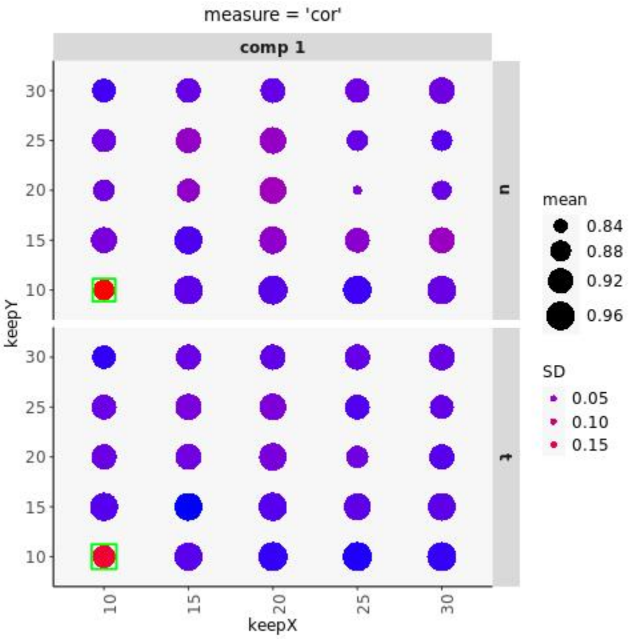

Supplementary Figure 82: The results of the optical coherence tomography – metabolite component number search. The green squares indicate the optimal number of components of to use for metabolites and embeddings in the component 1 analysis.

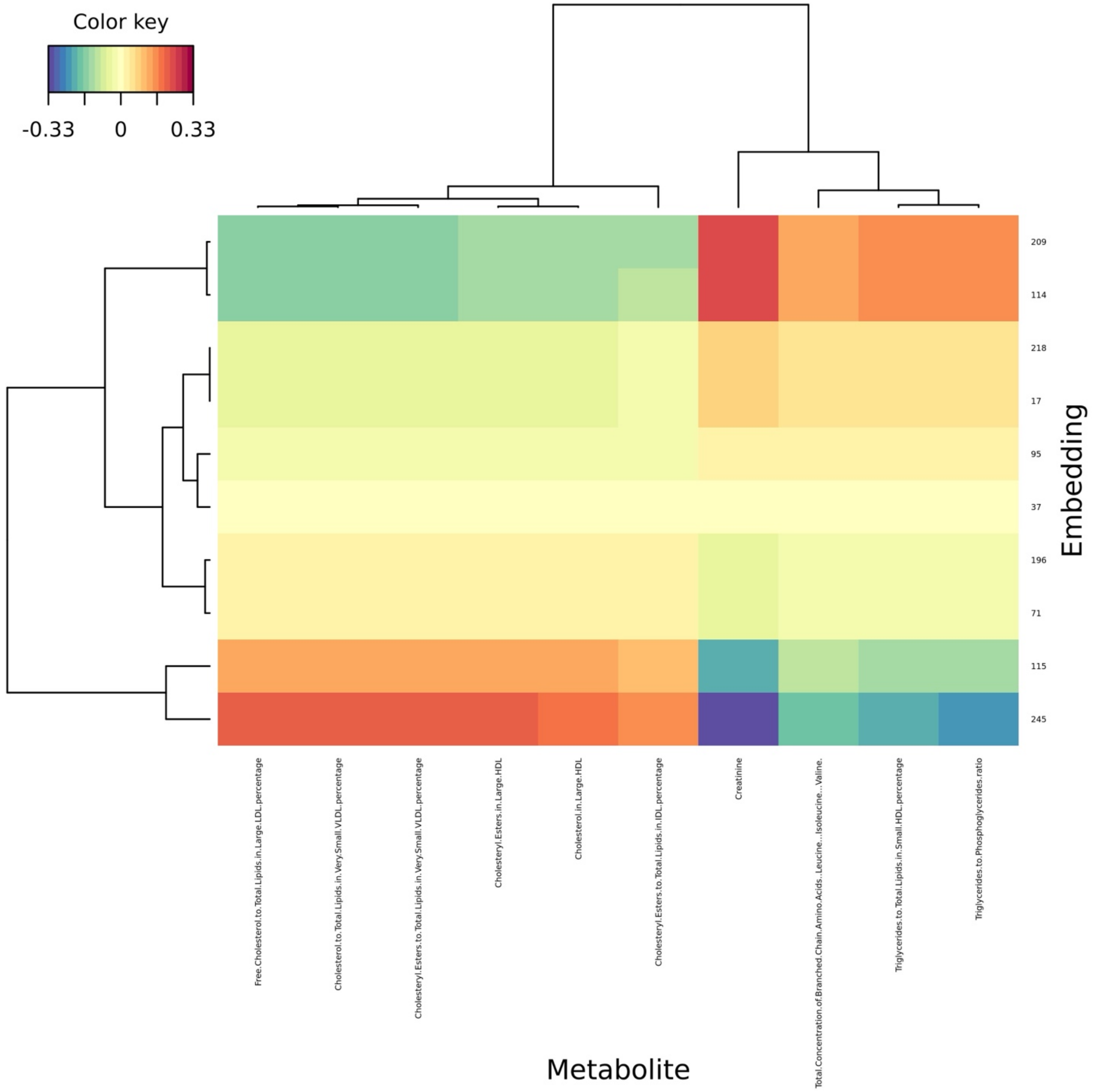

Supplementary Figure 83: A clustered heat map of the optical coherence tomography – metabolite sparse partial least squares analysis (SPLS). The plot features component one of the SPLS analysis and illustrates the correlation between features.

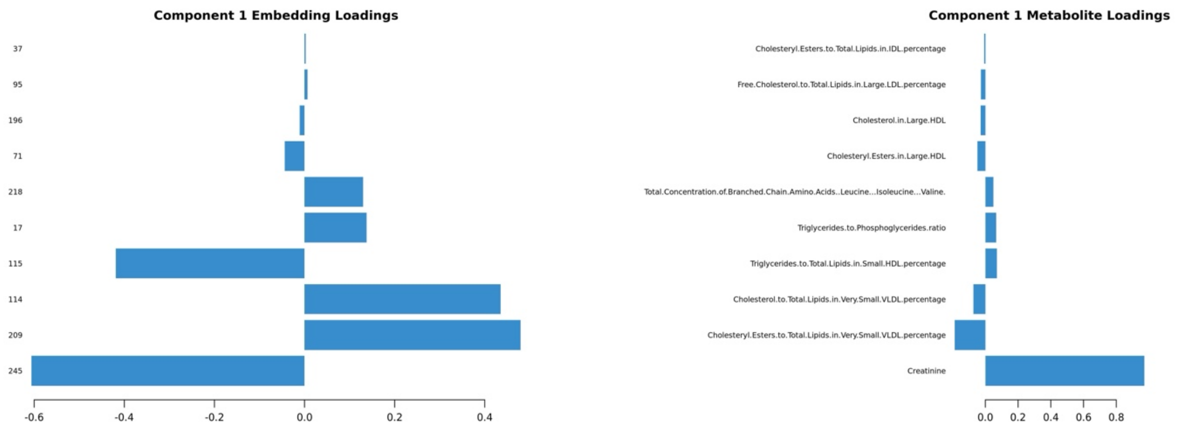

Supplementary Figure 84: A plot of each metabolite/embedding contribution to the latent components of the optical coherence tomography – metabolite sparse partial least squares analysis (SPLS). The plot shows which features drive embedding-metabolite associations in component 1.

Sensitivity Analyses

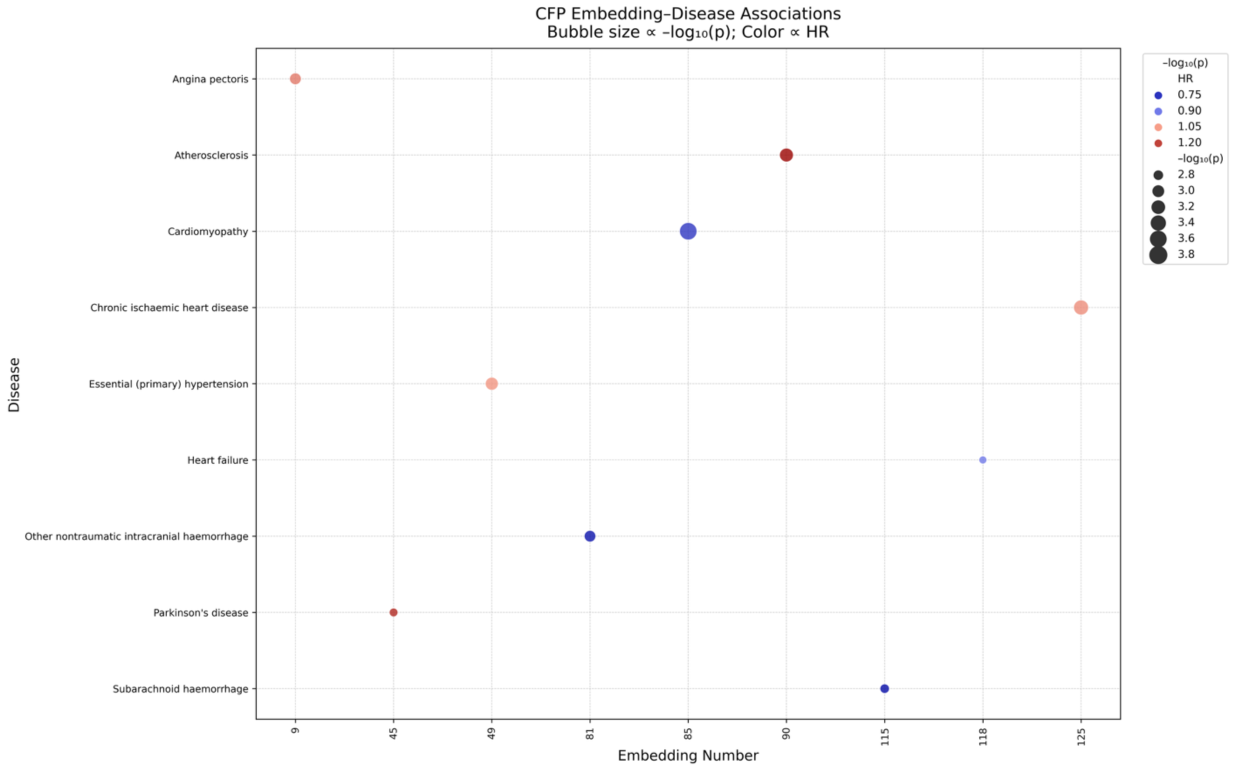

Supplementary Figure 85: A bubble plot illustrating the results of our **128-dimensional** CFP derived embedding Cox proportional hazards analysis. Only embeddings and disorders with at least one multiple testing corrected significant result are plotted here. The size of the bubbles indicates the level of significance (larger bubbles = greater significance, unadjusted). The bubbles are colour coded according to the hazards ratio (HR). The purpose of this analysis is to test the sensitivity of our results to selection of latent dimensionality.

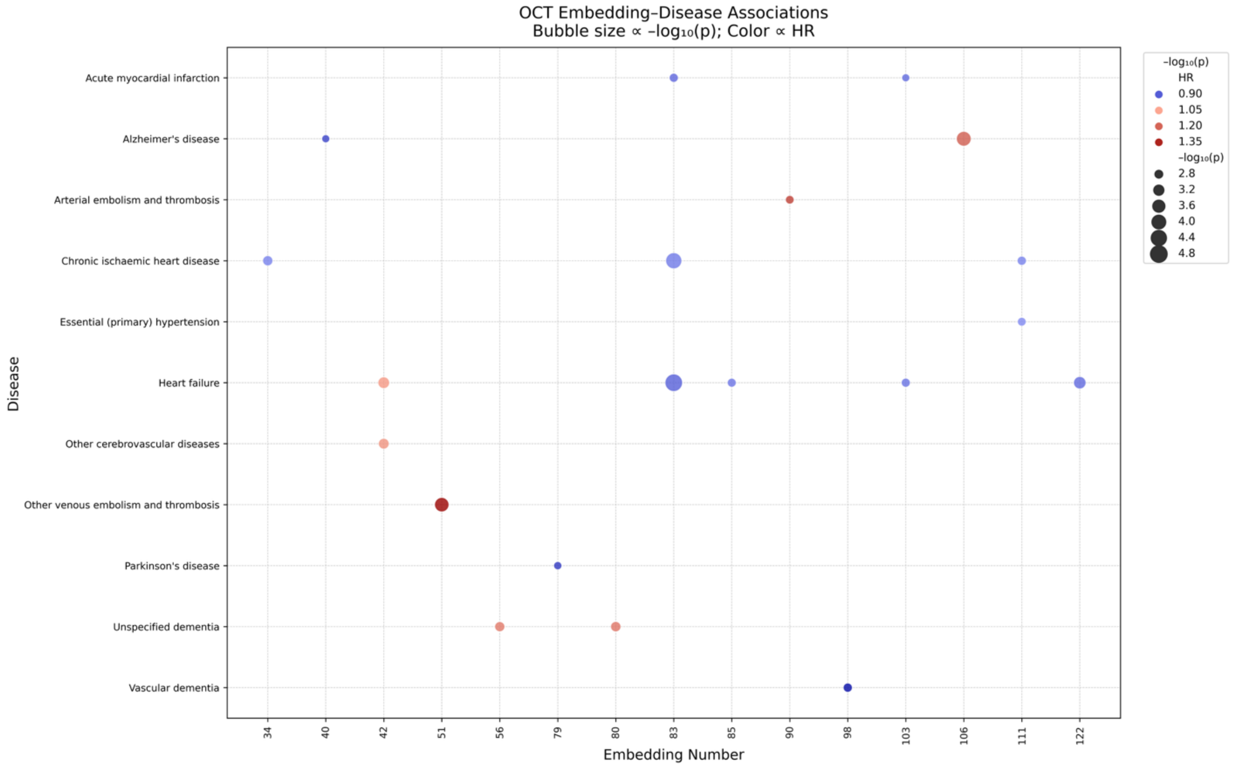

Supplementary Figure 86: A bubble plot illustrating the results of our **128-dimensional** OCT derived embedding Cox proportional hazards analysis. Only embeddings and disorders with at least one multiple testing corrected significant result are plotted here. The size of the bubbles indicates the level of significance (larger bubbles = greater significance, unadjusted). The bubbles are colour coded according to the hazards ratio (HR). The purpose of this analysis is to test the sensitivity of our results to selection of latent dimensionality.

Neurological Trait Analyses

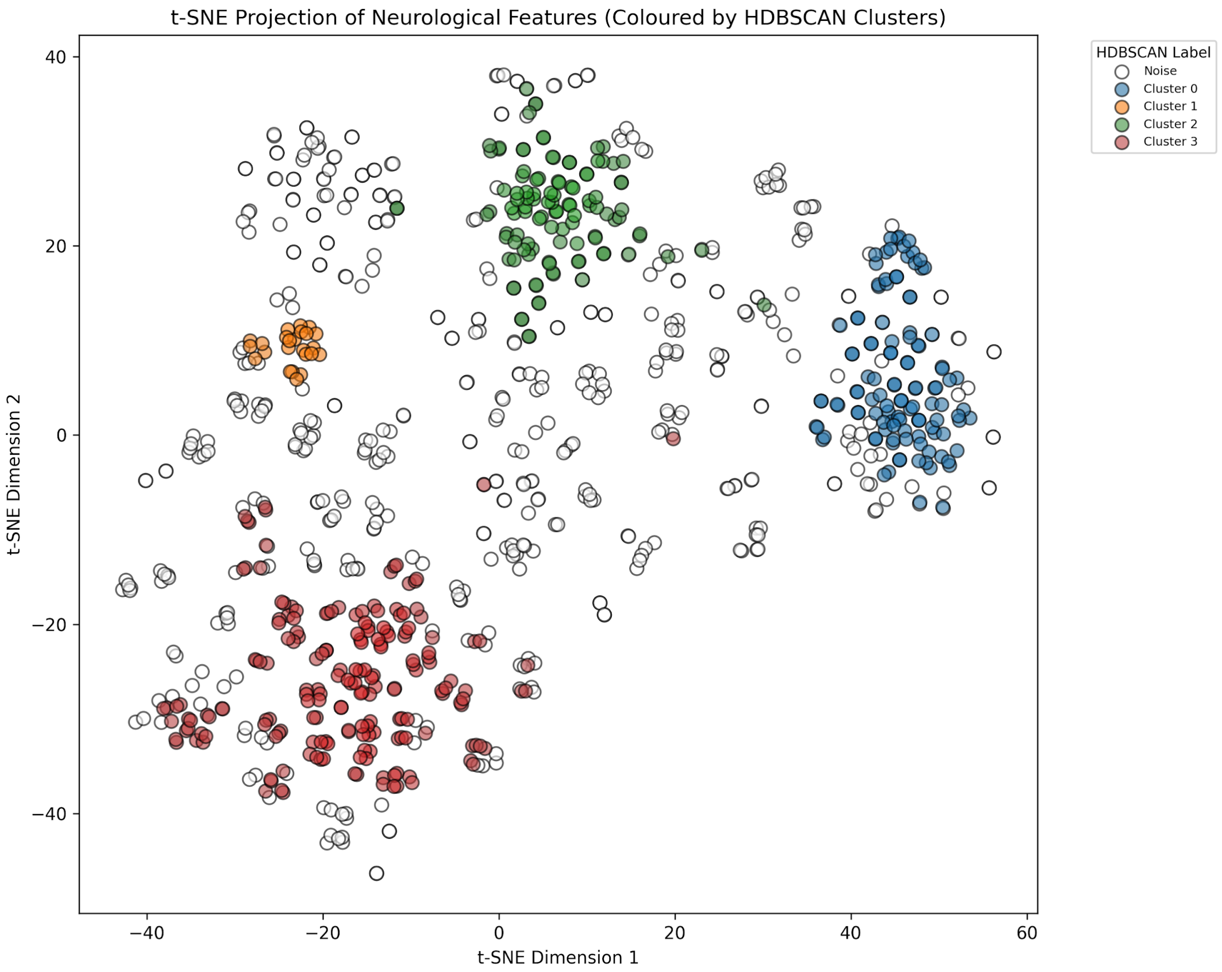

Supplementary Figure 87: A t-SNE projection of neurological features clustered using HDBSCAN. The clusters have been colour coded according to the HDBSCAN cluster.

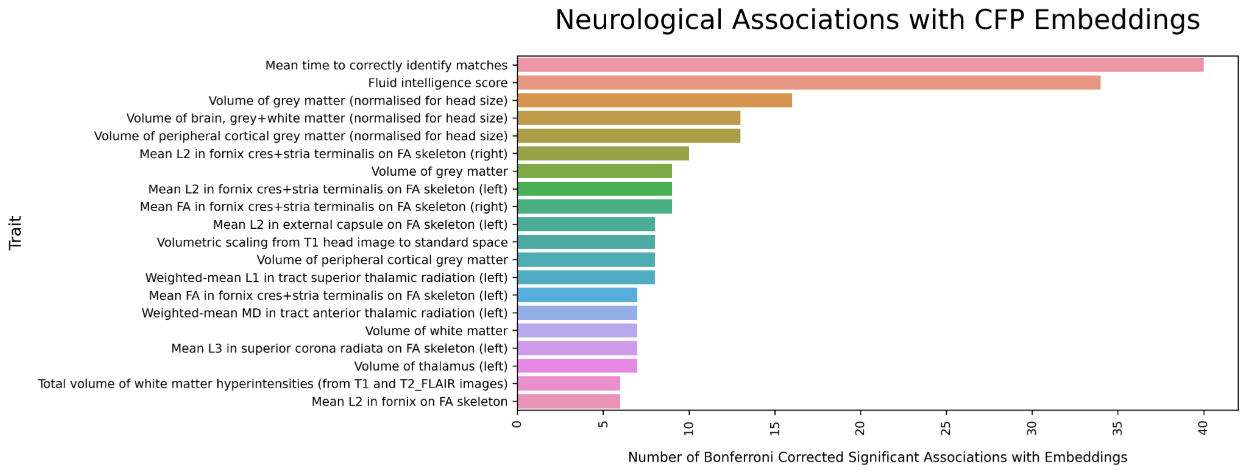

Supplementary Figure 88: A bar plot showing the top 20 neurological traits in terms of the number of multiple testing corrective significant associations with CFP embeddings.

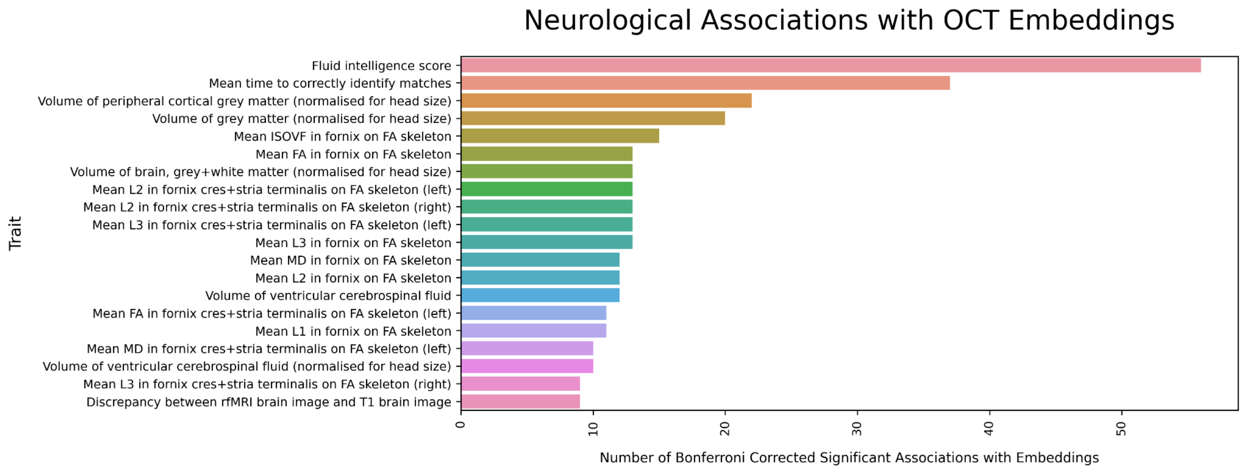

Supplementary Figure 89: A bar plot showing the top 20 neurological traits in terms of the number of multiple testing corrective significant associations with OCT embeddings.

Early Reconstruction Example

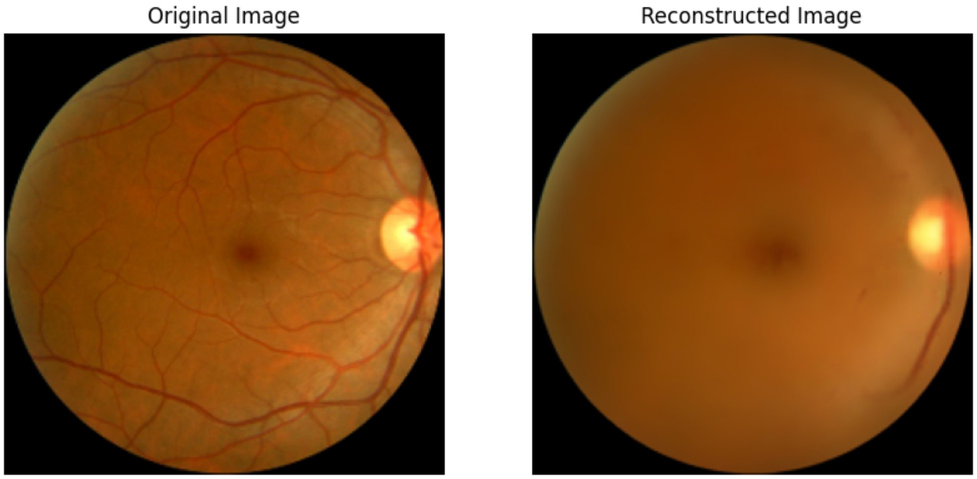

Supplementary Figure 90: An example of a CFP reconstruction prior to weighting of the vascular tree in our loss function. As can be seen, the model prioritised the background colour over reconstruction of the vasculature, which is amongst the most biologically meaningful features. Reproduced with the permission of UK Biobank.

Embedding-Embedding Correlations

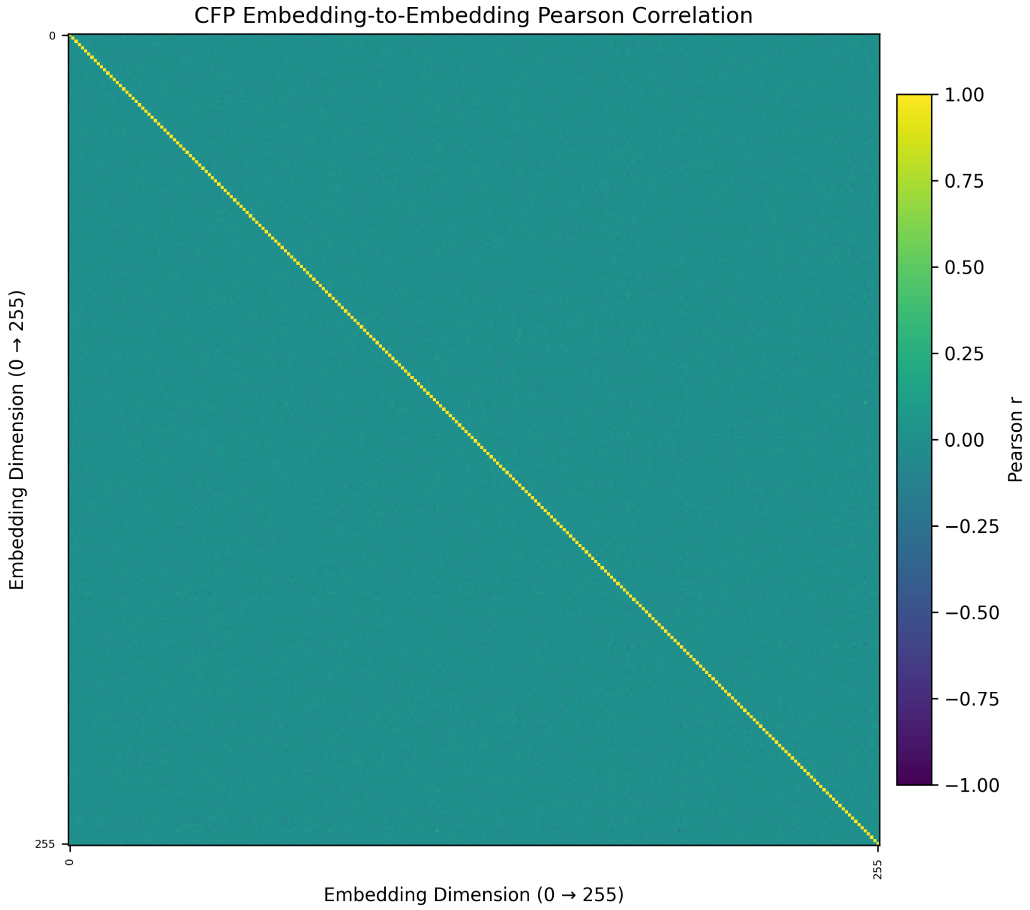

Supplementary Figure 91: A heat map of the correlations between CFP embeddings. As can be seen, there is perishingly little correlation between embeddings. This is an expected property given the distributional prior utilised.

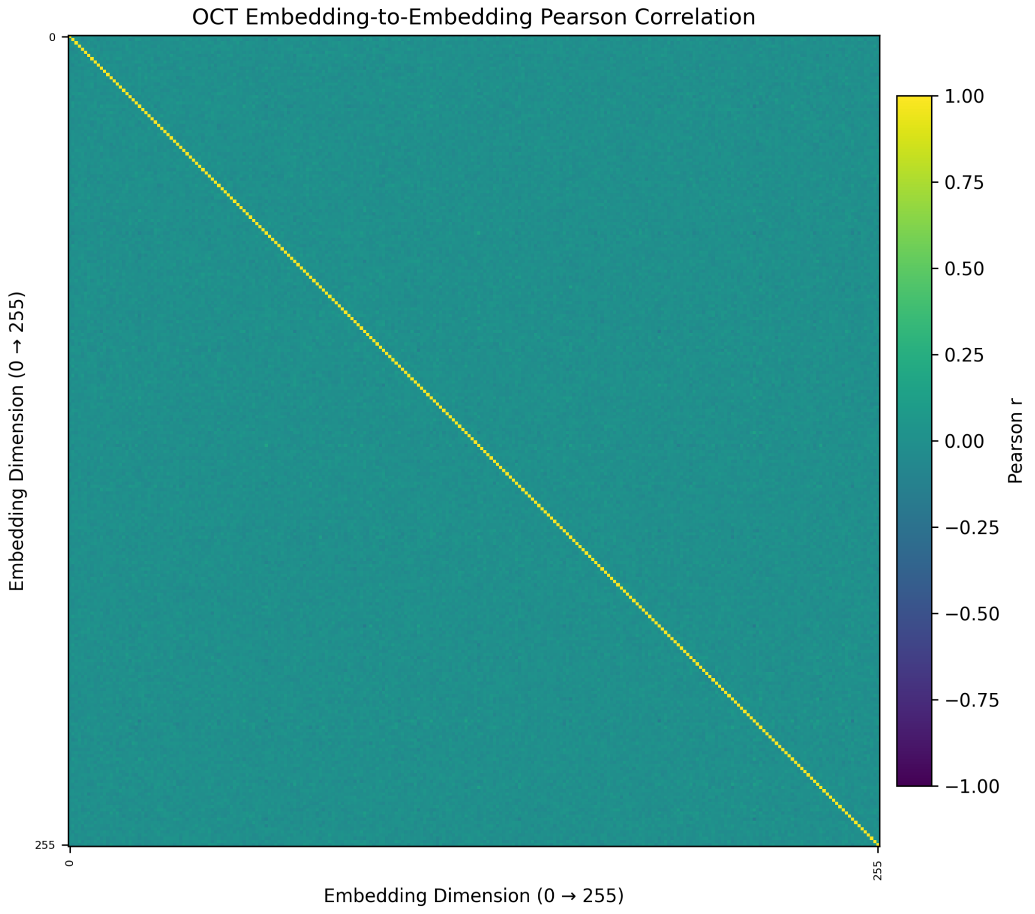

Supplementary Figure 92: A heat map of the correlations between OCT embeddings. As can be seen, there is perishingly little correlation between embeddings. This is an expected property given the distributional prior utilised.

UK Biobank Eye and Vision Consortium Membership:

Naomi Allen, University of Oxford, Oxford, United Kingdom

Tariq Aslam, The University of Manchester, Manchester, United Kingdom

Denize Atan, University of Bristol, Bristol, United Kingdom

Konstantinos Balaskas, Moorfields Eye Hospital, London, United Kingdom

Sarah Barman, Kingston University, London, United Kingdom

Jenny Barrett, University of Leeds, Leeds, United Kingdom

Paul Bishop, The University of Manchester, Manchester, United Kingdom

Graeme Black, The University of Manchester, Manchester, United Kingdom

Tasanee Braithwaite, St Thomas' Hospital, London, United Kingdom

Roxana Carare, University of Southampton, Southampton, United Kingdom

Usha Chakravarthy, Queen's University Belfast, Belfast, United Kingdom

Michelle Chan, Moorfields Eye Hospital, London, United Kingdom

Sharon Chua, UCL Institute of Ophthalmology, London, United Kingdom

Alexander Day, Moorfields Eye Hospital, London, United Kingdom

Parul Desai, Moorfields Eye Hospital, London, United Kingdom

Bal Dhillon, University of Edinburgh, Edinburgh, United Kingdom

Andrew Dick, University of Bristol, Bristol, United Kingdom

Alexander Doney, University of Dundee, Dundee, United Kingdom

Cathy Egan, Moorfields Eye Hospital, London, United Kingdom

Sarah Ennis, University of Southampton, Southampton, United Kingdom

Paul Foster, UCL Institute of Ophthalmology, London, United Kingdom

Marcus Fruttiger, UCL Institute of Ophthalmology, London, United Kingdom

John Gallacher, University of Oxford, Oxford, United Kingdom

David Garway-Heath, UCL Institute of Ophthalmology, London, United Kingdom

Jane Gibson, University of Southampton, Southampton, United Kingdom

Jeremy Guggenheim, Cardiff University, Cardiff, United Kingdom

Chris Hammond, King's College London, London, United Kingdom

Alison Hardcastle, UCL Institute of Ophthalmology, London, United Kingdom

Simon Harding, University of Liverpool, Liverpool, United Kingdom

Ruth Hogg, Queen's University Belfast, Belfast, United Kingdom

Pirro Hysi, King's College London, London, England, United Kingdom

Peng Tee Khaw, UCL Institute of Ophthalmology, London, United Kingdom
Anthony Khawaja, Moorfields Eye Hospital, London, United Kingdom
Gerassimos Lascaratos, Moorfields Eye Hospital, London, United Kingdom
Adam Lewandowski, University of Oxford, Oxford, United Kingdom
Thomas Littlejohns, University of Oxford, Oxford, United Kingdom
Andrew Lotery, University of Southampton, Southampton, England, United Kingdom
Robert Luben, UCL Institute of Ophthalmology, London, United Kingdom
Phil Luthert, UCL Institute of Ophthalmology, London, England, United Kingdom
Tom Macgillivray, University of Edinburgh, Edinburgh, United Kingdom
Sarah Mackie, University of Leeds, Leeds, United Kingdom
Savita Madhusudhan, Royal Liverpool University Hospital, Liverpool, United Kingdom
Bernadette Mcguinness, Queen's University Belfast, Belfast, United Kingdom
Gareth Mckay, Queen's University Belfast, Belfast, United Kingdom
Martin Mckibbin, Leeds Teaching Hospitals NHS Trust, Leeds, United Kingdom
Tony Moore, UCL Institute of Ophthalmology, London, United Kingdom
James Morgan, Cardiff University, Cardiff, United Kingdom
Eoin O'Sullivan, King's College Hospital, London, United Kingdom
Richard Oram, University of Exeter, Exeter, United Kingdom
Chris Owen, St George's, University of London, London, United Kingdom
Praveen Patel, Moorfields Eye Hospital, London, United Kingdom
Euan Paterson, Queen's University Belfast, Belfast, United Kingdom
Tunde Peto, Queen's University Belfast, Belfast, United Kingdom
Axel Petzold, UCL Institute of Neurology, London, United Kingdom
Nikolas Pontikos, UCL Institute of Ophthalmology, London, United Kingdom
Jugnoo Rahi, UCL Institute of Child Health, London, United Kingdom
Alicja Rudnicka, St George's, University of London, London, United Kingdom
Naveed Sattar, University of Glasgow, Glasgow, United Kingdom
Jay Self, University of Southampton, Southampton, United Kingdom
Sobha Sivaprasad, Moorfields Eye Hospital, London, United Kingdom
David Steel, Newcastle University, Newcastle, United Kingdom
Irene Stratton, Gloucestershire Hospitals NHS Foundation Trust, Gloucester, United Kingdom
Nicholas Strouthidis, Moorfields Eye Hospital, London, United Kingdom
Cathie Sudlow, University of Edinburgh, Edinburgh, United Kingdom
Zihan Sun, UCL Institute of Ophthalmology, London, United Kingdom

Robyn Tapp, St George's, University of London, London, United Kingdom
Dhanes Thomas, Moorfields Eye Hospital, London, United Kingdom
Emanuele Trucco, University of Dundee, Dundee, United Kingdom
Adnan Tufail, Moorfields Eye Hospital, London, United Kingdom
Ananth Viswanathan, Moorfields Eye Hospital, London, United Kingdom
Veronique Vitart, University of Edinburgh, Edinburgh, United Kingdom
Mike Weedon, University of Exeter, Exeter, United Kingdom
Katie Williams, King's College London, London, United Kingdom
Cathy Williams, University of Bristol, Bristol, United Kingdom
Jayne Woodside, Queen's University Belfast, Belfast, United Kingdom
Max Yates, University of East Anglia, Norwich, United Kingdom
Yalin Zheng, University of Liverpool, Liverpool, United Kingdom
Mervyn Thomas, University of Leicester, Leicester, United Kingdom
Annegret Dahlmann-Noor, Moorfields Eye Hospital, London, United Kingdom
