# Supplementary material for: Multi-omic analysis of deep learning-derived phenotypes links ophthalmic imaging to cardiovascular and neurological traits
Source: Nat Cardiovasc Res. 2026 Jun 16;5(6):541–54. doi: 10.1038/s44161-026-00815-5 (PMC13271892; doi:10.1038/s44161-026-00815-5)
Supplement: Supplementary file 2 — Reporting Summary [file 44161_2026_815_MOESM2_ESM.pdf]

nature portfolio

Corresponding author(s): Thomas Henry Julian

Last updated by author(s): Mar 16, 2026

Reporting Summary

Nature Portfolio wishes to improve the reproducibility of the work that we publish. This form provides structure for consistency and transparency in reporting. For further information on Nature Portfolio policies, see our Editorial Policies and the Editorial Policy Checklist.

Statistics

For all statistical analyses, confirm that the following items are present in the figure legend, table legend, main text, or Methods section.

n/a	Confirmed	
☐	☒	The exact sample size (*n*) for each experimental group/condition, given as a discrete number and unit of measurement
☐	☒	A statement on whether measurements were taken from distinct samples or whether the same sample was measured repeatedly
☐	☒	The statistical test(s) used AND whether they are one- or two-sided<br>*Only common tests should be described solely by name; describe more complex techniques in the Methods section.*
☐	☒	A description of all covariates tested
☐	☒	A description of any assumptions or corrections, such as tests of normality and adjustment for multiple comparisons
☐	☒	A full description of the statistical parameters including central tendency (e.g. means) or other basic estimates (e.g. regression coefficient) AND variation (e.g. standard deviation) or associated estimates of uncertainty (e.g. confidence intervals)
☐	☒	For null hypothesis testing, the test statistic (e.g. *F*, *t*, *r*) with confidence intervals, effect sizes, degrees of freedom and *P* value noted<br>*Give P values as exact values whenever suitable.*
☒	☐	For Bayesian analysis, information on the choice of priors and Markov chain Monte Carlo settings
☐	☒	For hierarchical and complex designs, identification of the appropriate level for tests and full reporting of outcomes
☐	☒	Estimates of effect sizes (e.g. Cohen's *d*, Pearson's *r*), indicating how they were calculated

Our web collection on statistics for biologists contains articles on many of the points above.

Software and code

Policy information about availability of computer code

Data collection	The data is available via UK Biobank upon application, approval, and payment of the relevant fees. We did not author the code which was implemented to develop the dataset.
Data analysis	We have presented our Ret-AAE code in a public GitHub repo which was included in our peer review: https://github.com/TomJulian/Ret-AAE.git

For manuscripts utilizing custom algorithms or software that are central to the research but not yet described in published literature, software must be made available to editors and reviewers. We strongly encourage code deposition in a community repository (e.g. GitHub). See the Nature Portfolio guidelines for submitting code & software for further information.

Data

Policy information about availability of data

All manuscripts must include a data availability statement. This statement should provide the following information, where applicable:

- Accession codes, unique identifiers, or web links for publicly available datasets
- A description of any restrictions on data availability
- For clinical datasets or third party data, please ensure that the statement adheres to our policy

UK Biobank data are available under restricted access through a procedure described at http://www.ukbiobank.ac.uk/using-the-resource/.

Research involving human participants, their data, or biological material

Policy information about studies with human participants or human data. See also policy information about sex, gender (identity/presentation), and sexual orientation and race, ethnicity and racism.

Reporting on sex and gender	In this study, we elected to utilise genetically determined sex (ie XY, XX) rather than gender or self-reported sex when describing population characteristics and when considering population characteristics as a covariate in analyses.
Reporting on race, ethnicity, or other socially relevant groupings	There are two uses of such data in this work:<br>(1) Genetically determined ancestry (ascertained using principal components) was used to identify European subjects, who were subsequently considered for genetic analysis.<br>(2) Self-reported ethnicity was reported for the purpose of population characteristic description and was used to explore how those who did and did not undergo ophthalmic imaging identified. We elected to use ethnic groups as defined in the most recent UK National Census.
Population characteristics	We have dedicated a section in the results 'Population characteristics of those with sufficiently high-quality images for phenotypic and genotypic analysis' to describing the characteristics of our study population. Here we described self reported ancestry, age, genetic sex, and economic status (Townsend score).
Recruitment	The recruitment strategy in the UK Biobank Eye and Vision Study has been described in detail in this publication: https://bmjopen.bmj.com/content/9/2/e025077
Ethics oversight	UK Biobank has approval from the North West Multi-centre Research Ethics Committee. The ethical approval processes surrounding UK Biobank are described here: https://www.ukbiobank.ac.uk/about-us/how-we-work/ethics/

Note that full information on the approval of the study protocol must also be provided in the manuscript.

Field-specific reporting

Please select the one below that is the best fit for your research. If you are not sure, read the appropriate sections before making your selection.

☒ Life sciences ☐ Behavioural & social sciences ☐ Ecological, evolutionary & environmental sciences

For a reference copy of the document with all sections, see nature.com/documents/nr-reporting-summary-flat.pdf

Life sciences study design

All studies must disclose on these points even when the disclosure is negative.

Sample size	We utlised all high-quality data available in the UK Biobank study. We utilised several positive control studies in order to demonstrate sufficient power for biological discovery. The exact sample size varies from analysis to analysis given that data availability is not consistent across all omics datasets available in UK Biobank. The sample size for every analysis is therefore explicitly reported throughout our study.
Data exclusions	Low quality OCT scans were excluded on the basis of an image quality score below 40. The quality score utilised was that which is native to the Topcon OCT device. CFPs were excluded if they were of 'reject' quality grading assessed using a deep learning method, 'Automorph' ('usable' and 'good' images were retained). Automorph was trained using the EyePACS-Q dataset, using grading provided by two experts according to image illumination, artifacts, and diagnosability of ocular diseases; the full method is documented in the referenced publication.<br><br>For phenotypic and omics datasets, subjects were omitted if there was missing data. The omissions are described comprehensively in text.<br><br>There was additional quality control for genetic analysis. Subject-level genetic quality control metrics were applied, including: exclusion of subjects whose genetic sex (Field 22001) does not match their reported sex (Field 31); exclusion of subjects with sex chromosome aneuploidy (Field 22019); exclusion of subjects with genetic kinship to other UKB participants (Field 2202); exclusion of subjects who were outliers for heterozygosity or missing rate (Field 22027), and removal of individuals with more than 10% missing genotype data.
Replication	The genetic analyses in the discovery cohort were conducted in subjects in instance 0 (initial assessment visit, 2006-2010). The genetic analyses in the replication cohort were conducted in subjects who attended within instance 1 (first repeat assessment visit, 2012-13).<br><br>Following reviewer feedback, we replicated disease association analysis using a lower dimensional autoencoder in order to ensure that our results were robust to selection of dimensionality.<br><br>It was not possible to externally replicate results. This is because there is not, to our knowledge, a multi-omic dataset available to us in which optical coherence tomography and colour fundus photographs have been captured which would allow replication.
Randomization	Randomization was not relevant to this study. There was no intervention being explored in this work.
Blinding	Blinding is not relevant to this study.

Reporting for specific materials, systems and methods

We require information from authors about some types of materials, experimental systems and methods used in many studies. Here, indicate whether each material, system or method listed is relevant to your study. If you are not sure if a list item applies to your research, read the appropriate section before selecting a response.

Materials & experimental systems

n/a	Involved in the study
☒	☐ Antibodies
☒	☐ Eukaryotic cell lines
☒	☐ Palaeontology and archaeology
☒	☐ Animals and other organisms
☐	☒ Clinical data
☒	☐ Dual use research of concern
☒	☐ Plants

Methods

n/a	Involved in the study
☒	☐ ChIP-seq
☒	☐ Flow cytometry
☒	☐ MRI-based neuroimaging

Clinical data

Policy information about clinical studies

All manuscripts should comply with the ICMJE guidelines for publication of clinical research and a completed CONSORT checklist must be included with all submissions.

Clinical trial registration: NA

Study protocol: The UK Biobank is not a clinical study, but benefits from health record linkage. UK Biobank describe their health record linkage on their webpage: https://www.ukbiobank.ac.uk/about-our-data/types-of-data/healthcare-records/

Data collection: Healthcare outcomes have been ascertained using community and hospital health records. The process has been described in the UK Biobank webpage: https://www.ukbiobank.ac.uk/about-our-data/types-of-data/healthcare-records/

Outcomes: The UKB first disease occurrences were generated by mapping read code information in the 'Primary Care data' (Category 3000), ICD-9 and ICD-10 codes in the 'Hospital inpatient data' (Category 2000), ICD-10 codes in 'Death Register records' (Field 40001, Field 40002), and 'self-reported medical condition codes' (Field 20002) reported at the baseline or subsequent UKB assessment centre visit. The UKB estimates that hospital inpatient data and death are mostly complete up to 31.05.2022, after which data may be incomplete. Accordingly, we utilised 31.05.2022 as the censoring date for our time-to-event analysis.

Plants

Seed stocks: *Report on the source of all seed stocks or other plant material used. If applicable, state the seed stock centre and catalogue number. If plant specimens were collected from the field, describe the collection location, date and sampling procedures.*

Novel plant genotypes: *Describe the methods by which all novel plant genotypes were produced. This includes those generated by transgenic approaches, gene editing, chemical/radiation-based mutagenesis and hybridization. For transgenic lines, describe the transformation method, the number of independent lines analyzed and the generation upon which experiments were performed. For gene-edited lines, describe the editor used, the endogenous sequence targeted for editing, the targeting guide RNA sequence (if applicable) and how the editor was applied.*

Authentication: *Describe any authentication procedures for each seed stock used or novel genotype generated. Describe any experiments used to assess the effect of a mutation and, where applicable, how potential secondary effects (e.g. second site T-DNA insertions, mosiacism, off-target gene editing) were examined.*
