# Supplementary material for: Multi-omic analysis of deep learning-derived phenotypes links ophthalmic imaging to cardiovascular and neurological traits
Source: Nat Cardiovasc Res. 2026 Jun 16;5(6):541–54. doi: 10.1038/s44161-026-00815-5 (PMC13271892; doi:10.1038/s44161-026-00815-5)
Supplement: Supplementary file 3 — Peer Review File [file 44161_2026_815_MOESM3_ESM.pdf]

nature portfolio

Peer Review File

Multi-omic analysis of deep learning-derived phenotypes links ophthalmic imaging to cardiovascular and neurological traits

Corresponding Author: Dr Thomas Julian

This file contains all reviewer reports in order by version, followed by all author rebuttals in order by version.

Version 0:

Reviewer comments:

Reviewer #1

(Remarks to the Author)
The submitted paper presents a large-scale analysis of the UK Biobank population study, aimed at discovering biomarkers of systemic disease in retinal images. To this end, the authors use self-supervised deep learning to represent color fundus photographs and optical coherence tomography images as compact 256-dimensional embeddings. The obtained embeddings are subsequently processed by a series of statistical analyses. First, the authors measure the correlation between each embedding and ocular, cardiovascular, and neurological disease traits as well metabolic biomarkers. Second, they associate the embeddings with systemic diseases diagnoses by fitting Cox proportional hazards models. Third, they conduct a genome-wide association study to match genetic variants and entire gene sets to the retinal image embeddings. Through the use of class activation maps each statistically significant finding is associated to regions in the encoded retinal images. The authors find that their retinal image embeddings are associated with several cardiovascular and neurological traits and diseases. Similarly, the embeddings are linked to several genes and gene sets in pathways that have been associated with the physiology of the vasculature and central nervous system as well as cardiovascular and neurological disease phenotypes.

The manuscript is clearly structured, very well written and accompanied by a large number of informative figures and tables. The relationship between retinal biomarkers and systemic disease is a topic of ongoing scientific interest. However, I slightly struggle to see the added value of the proposed method over the numerous previous studies in this increasingly popular field of oculomics. Additionally, I have several substantial concerns regarding the deployed methodology. I am listing these issues below on a point-by-point basis:

- Many studies have established a link between the retina, heart, and central nervous system (Wong et al. (2001). Retinal microvascular abnormalities and their relationship with hypertension, cardiovascular disease, and mortality. Survey of ophthalmology, 46(1), 59-80; London et al. (2013). The retina as a window to the brain - from eye research to CNS disorders. Nature Reviews Neurology, 9(1), 44-53 and many others) with more recent studies deploying deep learning (Poplin et al. (2018). Prediction of cardiovascular risk factors from retinal fundus photographs via deep learning. Nature biomedical engineering, 2(3), 158-164). While the specific strategy to incorporate deep learning may differ from previous studies, the presented method only seems to yield very limited new findings. Ultimately, the proposed method is restricted by the limited ability of class activation maps to link associated embeddings back to human-interpretable concepts, just as previous works based on supervised deep learning. While I found the GWAS results the most interesting, linking retinal image encodings to genotyping results has been also previously been explored (Taleb et al. (2022). Contig: Self-supervised multimodal contrastive learning for medical imaging with genetics. In Proceedings of the IEEE/CVF conference on computer vision and pattern recognition (pp. 20908-20921)). While the presented study may still be relevant due to its scale and width, I believe that the authors have to more clearly highlight how they differ from the rich body of previous works.

- The neural network architecture applies an adversarial loss to the latent embeddings, aiming to structure them according to a Gaussian distribution. As the seminal variational autoencoders (Kingma, and Welling. "Auto-encoding variational bayes." ICLR, 2013) have established using the Kullback-Leibler divergence for this purpose, the authors' decision to employ an adversarial loss warrants further rationale. Ideally, this should be supported by references to prior work utilizing a similar approach.

- I do not think that a correlation matrix based on Pearson's correlation coefficient is sufficient proof that the individual embeddings are independently distributed. Considering that all embeddings are processed by a highly non-linear neural network, individual embeddings may very well correspond to similar image concepts, even if they are not linearly correlated. As such, I believe that subsequent statistical analyses should not rely on the assumption that the embeddings are independently distributed.

- I am doubtful whether the implemented correction for multiple testing, which views each embedding as a "family" and only using a family-wise error rate control, is methodologically sound. In my understanding, correcting for multiple testing aims to guard against false discoveries (Farcomeni, Alessio. "A review of modern multiple hypothesis testing, with particular attention to the false discovery proportion." Statistical methods in medical research 17.4 (2008): 347-388). Using the currently employed correction for multiple testing, one could simply increase the dimensionality of the embedding to 512 to double the number of associations. I strongly believe that all results have to correct for the number of individually processed embeddings.

- Closely related to the previous two points, I am not sure whether the number of embedding dimensions correlated with a trait is a good metric. Even if all the embeddings were truly uncorrelated and associated with a phenotype, a single embedding may be just as important as 50 others considering that they are subsequently processed by a high-dimensional neural network.

- The authors use HDBSCAN to cluster an array of ocular, cardiovascular and neurological traits, before associating the clusters with the extracted retinal image embeddings. They motivate this decision as it purposedly "enhances interpretability." However, it is not clear to me how the clusters offer increased interpretability compared to the clearly defined UK Biobank data fields. Without appropriate rationale, I believe this clustering step should be replaced with a more transparent strategy or omitted entirely.

- Many cardiovascular and neurological diseases are notoriously underdiagnosed (Soljak et al. (2011). Variations in cardiovascular disease under-diagnosis in England: national cross-sectional spatial analysis. BMC cardiovascular disorders, 11(1), 12), which most likely affects the UK Biobank health report labels. I believe the authors should clearly discuss this issue and how it affects their study.

- While initially reading the paper, I was wondering whether the authors correct for age and sex. Only far in the methods section, I realized that the authors actually correct for this. I suggest already briefly mentioning this earlier than page 20.

- As discussed in the paper, ethnicity affects retinal pigmentation and has been linked to an increased prevalence of systemic disease. I believe the authors have to further explain their decision to not correct for these potential confounders as they have done with age and sex.

Reviewer #2

(Remarks to the Author)
Julian et al. explored the eye as a window to cardiovascular and neurodegenerative disease using a comprehensive multi-omics pipeline that integrated physiological, radiomic, metabolomic, and genomic information. The study was well designed to investigate the biological processes linking ocular features with systemic health, and it was clearly written and presented. I have two main suggestions for the authors to consider:

Clarification of Ret-AAE embeddings
The rationale for using the trained Ret-AAE to represent OCT images and CFP as 256-dimensional embeddings requires further explanation. A key issue lies in how these abstract embeddings associate with systemic diseases such as cardiovascular and neurodegenerative conditions, and more importantly, how they can be interpreted in a clinically meaningful way. AI-derived features such as retinal vascular parameters from Automorph (e.g., tortuosity) or retinal ageing biomarkers (e.g., retinal age, reti-Age, reti-phenoAge) are more intuitively interpretable by clinicians and patients. In contrast, the clinical relevance of 256-dimensional embeddings is not immediately clear. Providing additional explanation and context on how these embeddings can be understood and applied in a clinical setting would strengthen the manuscript.

Imaging quality control
Image quality control is crucial for ensuring robust analyses. Could the authors clarify how Automorph was applied to determine image quality and whether any thresholds or exclusion criteria were used? Clear reporting of these methods would improve reproducibility and interpretation.

Reviewer #3

(Remarks to the Author)
Julian and co-workers have performed a comprehensive analysis of ophthalmic imaging as a measure of cardiovasular and neurological health. While they create an impressive pipeline of analyses, the compiled results are described in very broad terms. I would recommend the authors to dive into the most convincing pathways, and perhaps add ekstra targeted analyses to exploit possible functionality further.

One lacking area is the importance of the pivotal APOE gene and its common functional variants - the e2/e3/e3 polymorphism - fundamental in peripheral and local lipid metabolism, risk of cardiovascular disease, age-related macular degeneration, and Alzheimer's disease, vascular dementia, and dementia in general. See PMID:36394841, PMID:31236578, PMID:32808727, PMID:38054895, and PMID:40385391 for inspiration.
I suggest you make APOE stratified analyses (e2 carrier, e33, and e4 carrier (excluding e42)

GWAS pipeline.
A stingent GWAS pipeline has been used. REGENIE 4.1 was selected, and all QC work seems appropriate. Please mention how you dealt with palindromic variants. Sufficient details are given to enable validation from external scientists.

Reviewer #4

(Remarks to the Author)
Summary: This is an ambitious and extensive study integrating deep-learning-derived retinal image embeddings with multiple omic data layers (genomic, metabolomic, radiomic) and clinical data from UK Biobank. The scale of analysis and the multi-omic integration are impressive. The general concept of using retinal imaging as a non-invasive window into systemic health strikes me as both sensible and potentially highly impactful, hence I believe this work to be both timely and relevant. Overall, I felt that the methodological foundation of the manuscript was strong, but I would be keen to see some additional clarifying details relating to reproducibility and statistical/machine learning analyses.

1. Deep-learning model development: The adversarial autoencoder (AAE) framework seems well suited for unsupervised representation learning, and the use of perceptual and weighted L1 losses sound. However, it would be great if the authors could address the following points: (a) With apologies if I missed it, the manuscript seems not to provide access to code, model weights, or configuration files. I would appreciate the opportunity to review these, and releasing these (e.g. via GitHub + Zenodo DOI) would seem essential for reproducibility. (b) Some further details of training the model (beyond the 35 epochs per configuration in the hyperparameter sweep) would be appreciated (the total number of epochs, convergence criteria, random-seed settings, …). Moreover, given the instability of adversarial training, I think that evidence of convergence (loss curves or reconstruction trajectories) should be provided. (c) Regarding the Bayesian hyperparameter sweep using the Weights & Biases platform, could the W&B version, as well as the hyperparameter search space, and acquisition function for the BayesOpt be provided? (d) I understood the choice of a 256-dimensional latent space to provide a balance between reconstruction quality (SSIM improvement compared to 128 dimensions) and computational/statistical feasibility. This seems reasonable; however, I couldn't help but wonder if the downstream analyses were robust to this choice of dimensionality? Could the authors discuss the robustness of the downstream findings to the choice of latent dimensionality (e.g. whether key associations are expected to be similar at 128 vs 256 dimensions), even if a full re-analysis is not feasible? (e) The OCT dataset (~89 k images) is quite a lot larger than the CFP dataset (~64 k) – should this be discussed in the manuscript, in terms of the potential impact on reconstruction quality and embedding stability (as well as on downstream analyses)?

2. GWAS and heritability analyses: (a) Running 512 separate GWAS (256 CFP + 256 OCT embeddings) would seem to introduce a substantial multiple-testing burden. Unless I have misunderstood, the authors are applying a standard genome-wide threshold, which provides appropriate control of false positives within each GWAS, but not across all 512 analyses taken together? If this is correct, the authors could consider either applying a cross-trait correction (e.g. Bonferroni, FDR, ..), or the analyses could be framed as exploratory (although I acknowledge that the instance-1 replication mitigates false positives to some extent). [Relatedly, I think the reported genetic correlations derived from LD score regression should be interpreted cautiously, given the small number of embeddings with reliably measurable heritability -- also, it was not entirely clear to me how multiple-testing correction was handled within these correlation analyses.] (b) Only a small fraction of embeddings (≈3 % OCT, 9 % CFP) show measurable heritability. This low polygenicity would seem to suggest that most embedding variance is environmental or technical – could the authors discuss this?

3. Phenotypic association analyses: (a) Associations with cardiovascular and neurological traits vary in sample size (≈11k–63k). If I have understood correctly, the authors restricted each analysis to participants with available data for the relevant modality or trait. It would be very helpful to include a supplementary summary table characterising the extent and pattern of missingness across data modalities, and ideally comparing participant characteristics (e.g. age, sex, health indicators) between those with and without each data type. This would provide readers with a sense of whether missingness might be non-random and therefore a potential source of bias. (b) For the baseline disease associations, my understanding is that participants with existing disease at the time of imaging were included. This makes it difficult to disentangle underlying risk from the effects of established disease or treatment. The manuscript also reports prospective (incident) disease analyses using Cox models; could the authors provide a direct comparison of these results with the baseline (prevalent) analyses for key outcomes, to help clarify how embedding differences relate to risk versus existing disease?

4. Survival modelling: Cox proportional hazards models seem sensible for the analyses performed. To strengthen the results, the authors might consider explicitly stating how the proportional-hazards assumption was assessed. Reporting diagnostics - such as those based on Schoenfeld residuals or graphical methods - for key outcomes (e.g. hypertension, heart failure, and dementia) may give readers greater confidence in the validity of the reported hazard ratios.

5. Canonical correlation analysis (CCA): (a) CCA seems an appropriate and sensible analysis approach here, but it can be prone to overfitting in high-dimensional settings. To mitigate this, the authors could consider using a cross-validated and/or

regularised (e.g. sparse or penalised) CCA. (b) The manuscript provides a qualitative summary of the dominant metabolites (e.g. HDL, LDL, VLDL) and lists the top contributing metabolites and embeddings in the supplementary tables. However, interpretability might be further enhanced by visualising the broader loading structure (e.g. clustered heatmaps or correlation maps).

6. General comment re: effect sizes: Effect sizes across analyses are typically small ($|r| < 0.2$, modest hazard ratios). The manuscript might benefit from explicit discussion of practical significance versus statistical significance. Even with large samples, very small effects may lack biological or clinical meaning. For Cox models, discrimination metrics could be reported to quantify predictive value.

7. General comment re: instance 0/1 split: The instance 0 / instance 1 split is clearly used for GWAS discovery and replication, and I can see that the CCA analysis restricts to instance 0 due to participant overlap. However, it was less clear to me whether this separation was applied consistently across all other analyses – could this be clarified?

Version 1:

Reviewer comments:

Reviewer #1

(Remarks to the Author)
The authors have presented an extensive and well written rebuttal to the four reviewers' comments. They have fully answered my questions regarding the deployed deep learning architecture, label quality in the UK Biobank dataset, and accounting for subjects age, sex, and ethnicity. Crucially, they have clarified the envisioned scope of their study, explaining their aim to "identify associations between ocular [deep-learning derived image] features and cardiovascular/ neurological disease" and "systematically explore potential mechanistic explanations for these associations". I believe that this clarification has helped to distinguish this study from previous works and positions it in a very interesting field of research, machine-learning-driven biomarker discovery. However, viewing the work through this lens amplifies two concerns that were already raised in the previous round of reviews: a lack of explainability of the deep-learning-derived imaging features and uncertainty regarding the robustness of any discovered associations.

I agree with the authors' decision to avoid already established ocular biomarkers, such as retinal layer thicknesses or vessel diameters. However, the proposed deep-learning-derived biomarkers are inherently difficult to interpret. As already mentioned in my initial review, the proposed method is restricted by the limited ability of class activation maps to link embeddings back to human-interpretable concepts. Additionally, I believe that the data-driven clustering of the traits adds another level of abstraction to the entire pipeline, even if the clusters are deemed plausible by experts and I understand the practical rationale for this step.

The concerns regarding the robustness of the discovered associations is directly linked to the previous comments regarding the correction for multiple testing by Reviewer #4 and me. The authors have attempted to alleviate these concerns by adding a section on the type I error risk. While I understand the difficulty balancing type I and type II errors and issues surrounding correcting for multiple testing, I still think that the definition of individual embeddings as a family is arbitrary. Importantly, the authors write that "key study findings will benefit from external experimental validation." Indeed, I believe that replicating (parts of) the results on a separate dataset would convincingly demonstrate the robustness and practical value of the proposed method.

I understand that addressing both these issues is very difficult, as linking deep learning embeddings to human-interpretable concepts is a complicated field of active research and large multiomic datasets are rare. However, I believe that addressing at least one of these fundamental issues is required demonstrate the practical value of the work and necessary for me to fully support acceptance of this work.

On a lesser note, I remain not fully convinced regarding the clustering of the cardiovascular and neurological disease traits and the use of number of correlated embeddings as a quantitative metric.

(Remarks on code availability)

Reviewer #2

(Remarks to the Author)
Authors have addressed all my concerns.

(Remarks on code availability)

Reviewer #3

(Remarks to the Author)

(Remarks on code availability)

Reviewer #4

(Remarks to the Author)
I thank the authors for their revisions and thoughtful responses, and am particularly grateful for the GitHub link. Almost all of my previous points have been comprehensively addressed.

I have one remaining query regarding latent dimensionality, although I may just be missing the authors' point (comment below).

Remaining point: Latent dimensionality justification

I appreciate the authors' response to my previous comment regarding the robustness of findings to the choice of latent dimensionality. However, I am not yet wholly convinced that this concern has been thoroughly addressed.

The authors argue that "the same overall ocular features will necessarily be encoded" regardless of dimensionality. While I accept that a reasonable autoencoder should capture the major anatomical structures to achieve good reconstruction, my concern is less about which features are encoded, and more about the granularity at which they are represented.

Specifically, at lower dimensionalities, the model may be forced to compress more aggressively, potentially merging features that are biologically distinct. At higher dimensionalities, these features might be separated. Reconstruction error alone does not necessarily uniquely determine how features are merged or separated -- and it seems to me plausible that different combinations could yield different downstream associations (not because different anatomy is captured, but because related features are combined or split differently).

I do not think this concern is fully addressed by the convergence of evidence across analyses, since all analyses were conducted using the same 256-dimensional embeddings.

I therefore wonder if the authors could consider either: (i) A limited sensitivity analysis, checking whether some of the top disease associations per modality remain qualitatively consistent when using the 128-dimensional model; or (ii) An acknowledgement in the limitations that the choice of 256 dimensions was pragmatic, that reconstruction quality does not guarantee identical feature organisation across dimensionalities, and that downstream findings might be sensitive to this choice.

(Remarks on code availability)
I have briefly reviewed the repo. The code is well-organised and includes a clear README with installation instructions, prerequisites, and usage guidance. The architecture is disclosed in models.py, and the scripts for generating embeddings (CFP_AAE.py, OCT_AAE.py) are straightforward.
There are, however, unavoidable limits to the extent to which the code can be tested: as explained by the authors in their response, the decoder weights cannot be shared due to UK Biobank policies on generative models, and running the encoder requires access to appropriately formatted ophthalmic images. These constraints are not the authors' fault and are clearly documented. Recognising these limits, the code nevertheless appears functional and should be a usable resource for researchers with access to compatible imaging data.

Version 2:

Reviewer comments:

Reviewer #1

(Remarks to the Author)
Although I appreciate the authors' detailed response and efforts to include two additional experiments, my fundamental concerns remains: the study is not able to conclusively demonstrate the robustness of any found links between ophthalmological image features and biological processes and systemic diseases, respectively.

I would argue that the reported "oculo-systemic associations" are a sort of imaging biomarker. Professional medical societies have demanded an increased level of proof for novel imaging biomarkers (e.g. O'connor, James PB, et al. "Imaging biomarker roadmap for cancer studies." Nature reviews Clinical oncology 14.3 (2017): 169-186). I acknowledge that the presented study is positioned at an early stage of the biomarker discovery pipeline and that finding a validation dataset of the scale of the UK Biobank is difficult. Still, I believe that establishing the proposed deep learning pipeline for biomarker discovery and validating any found associations requires proof beyond the level of evidence provided by the study.

Any concerns regarding the unclear robustness are amplified by the limited interpretability of the found imaging features. The provided experiments showcasing the effect of perturbing individual entries of the latent embedding on the decoded

images are interesting, but far from comprehensive and conclusive. In particular, it is unclear if the found deep-learning-derived image features are not simply a decomposition of already known physiology-based image features.

At the same time, I would like to explicitly acknowledge the many strengths of the study: its innovative and interesting research question, clear description of methods and results, large experimental scale, and high-quality writing and figures. My sole major concern is that the level of evidence falls slightly short of the bar I would have personally liked to see. Crucially, I feel that further revisions will not be able to close the gap between the authors' and my position, so that I am happy to leave the final decision to the editor.

(Remarks on code availability)

Reviewer #4

(Remarks to the Author)
I thank the authors for their revisions and for undertaking the sensitivity analysis.

The authors have addressed my one previous point, and I have no further comments.

(Remarks on code availability)
I have checked that the above link still works. The repo does not appear to have changed since the previous round of reviews.

Open Access This Peer Review File is licensed under a Creative Commons Attribution 4.0 International License, which permits use, sharing, adaptation, distribution and reproduction in any medium or format, as long as you give appropriate credit to the original author(s) and the source, provide a link to the Creative Commons license, and indicate if changes were made.
In cases where reviewers are anonymous, credit should be given to 'Anonymous Referee' and the source.
The images or other third party material in this Peer Review File are included in the article's Creative Commons license, unless indicated otherwise in a credit line to the material. If material is not included in the article's Creative Commons license and your intended use is not permitted by statutory regulation or exceeds the permitted use, you will need to obtain permission directly from the copyright holder.
To view a copy of this license, visit https://creativecommons.org/licenses/by/4.0/

4th December 2025

Dr Thomas H. Julian
Division of Evolution, Infection and Genomics,
School of Biological Sciences,
Faculty of Biology, Medicine, and Health,
University of Manchester,
Manchester, UK.
Email: thomas.julian@manchester.ac.uk

Dear Reviewers,

Thank you for considering our manuscript titled "Ophthalmic imaging as a measure of cardiovascular and neurological health: a multi-omic analysis of deep-learning derived phenotypes" for publication in *Nature Cardiovascular Research*.

We are grateful for your thoughtful and comprehensive feedback. We have carefully evaluated all your suggestions and critiques, undertaking several new analyses to address your comments empirically wherever possible.

In response to your feedback, we have revised the manuscript content to improve the clarity of writing, undertaken new experiments (e.g. sparse partial least squares analysis, comparison of imaged and non-imaged populations, and new Cox-related analyses), and produced >200 new requested supplementary visualisations/figures. We believe the revisions have substantially strengthened the manuscript.

Please find our responses to your comments below. Thank you for your time and consideration.

Kind regards,
Dr Tom Julian, on behalf of the authors

4th December 2025

Response to editors' comments:

Comment 1

Provide access to the code for our referees and submit a filled-in reporting summary.

Response 1

We apologise for this omission. We have now provided access to the code and model weights via a public repository on GitHub. We have directed readers to this repository in the manuscript to allow users to use Ret-AAE in their own work. The repository is available here: https://github.com/TomJulian/Ret-AAE. We have completed the required reporting summary and model card, and submitted the document alongside our revisions. We would like to note that all details requested in the model card are additionally detailed in the manuscript and/or the GitHub repository.

Comment 2

Improve the statistical analysis according to the suggestions by referees #1 and #4, clarify how HDBSCAN-derived clusters enhance the interpretability of the findings (referee #1), and compare the incidence and prevalence analysis for key outcomes (referee #4).

Response 2

Thank you for the opportunity to improve our analysis. In response to the highlighted reviewer comments, we have made the following changes:

- We have conducted **sparse partial least squares** analysis in response to Reviewer 1 – Comment 3 and Reviewer 4 – Comment 11.
- We have presented **clustered heat maps** for our metabolomic analysis in response to Reviewer 4 – Comment 12.
- We have produced **residual plots** and **partial effects plots** in response to Reviewer 4 – Comment 13.
- We have **compared population characteristics** graphically and within the manuscript itself in response to Reviewer 4 – Comment 8.
- We have **clarified the role of HDBSCAN clusters** in our response to Review 1 – Comment 6.
- Regarding the relationship between incident and prevalent disease, we have clarified the reason that this comparison has been omitted in response to Reviewer 4 – Comment 9.

Comment 3

Associate the embeddings with more human-interpretable concepts, ideally retinal phenotypic features (as recommended by referees #1 and #2), and highlight the advantages of this work over the established literature.

Response 3

The reviewer comments indicated that we needed to clarify the advantages of using deep learning derived imaging features over human defined imaging labels. Accordingly, **we have altered our introduction to frame this more clearly for readers**. We have also provided an extended explanation of the advantages of our approach in response to Reviewer 1 – Comment 1. Deep learning derived features do not strictly align with individual human labels, and we do not believe it would be scientifically sound for us to claim that specific embeddings represent conventional ophthalmic imaging labels (e.g. venular or arteriolar widths). With that said, we have aligned the embeddings to human interpretable concepts to the extent that it is possible using saliency maps (GradCAM), and we have noted the ocular features embeddings localise to in text when presenting our results, as well as in the discussion. We have presented saliency maps for the most important embeddings in the supplementary figures. The use of deep learning derived features liberates our analysis of human biases and is a key strength of our approach, as we have now made clearer in the manuscript – please kindly see a detailed explanation of our rationale for this in Reviewer 1 – Response 1.

Comment 4

Stratify the GWAS analysis based on APOE variants (referee #3).

Response 4

APOE is located on **chromosome nine**, which although implicated in several diseases, has not been identified in ophthalmic imaging GWAS. As we explain in our response to Reviewer 3 – Comment 1, **our results feature no genome-wide significant, independent variants on chromosome nine** for which we can adjust our results. This is in keeping with previous GWAS by our group and others, which have very rarely identified significant associations with ophthalmic imaging features in chromosome nine (and never in *APOE* – see kindly see the references provided in our response). Please kindly refer to our detailed response to this point provided in Reviewer 3 – Response 1. As we document in our letter to the editor, we are open to discussing completion of this analysis should the editor regard it essential for publication despite the evidence we have presented.

4th December 2025

Reviewers' comments:

Reviewer #1

Comment 1

Many studies have established a link between the retina, heart, and central nervous system (Wong et al. (2001). Retinal microvascular abnormalities and their relationship with hypertension, cardiovascular disease, and mortality. Survey of ophthalmology, 46(1), 59-80; London et al. (2013). The retina as a window to the brain - from eye research to CNS disorders. Nature Reviews Neurology, 9(1), 44-53 and many others) with more recent studies deploying deep learning (Poplin et al. (2018). Prediction of cardiovascular risk factors from retinal fundus photographs via deep learning. Nature biomedical engineering, 2(3), 158-164). While the specific strategy to incorporate deep learning may differ from previous studies, the presented method only seems to yield very limited new findings. Ultimately, the proposed method is restricted by the limited ability of class activation maps to link associated embeddings back to human-interpretable concepts, just as previous works based on supervised deep learning. While I found the GWAS results the most interesting, linking retinal image encodings to genotyping results has been also previously been explored (Taleb et al. (2022). Contig: Self-supervised multimodal contrastive learning for medical imaging with genetics. In Proceedings of the IEEE/CVF conference on computer vision and pattern recognition (pp. 20908-20921)). While the presented study may still be relevant due to its scale and width, I believe that the authors have to more clearly highlight how they differ from the rich body of previous works.

Response 1

We thank the reviewer for their comment. **In response, we have altered our introduction to address any ambiguity surrounding the role of deep learning-derived phenotypes and how our work builds on the existing literature.**

We note that some of the studies highlighted in this comment are disease prediction papers (e.g. Poplin et al.) , and emphasize that this is not the purpose of our study. Other studies highlighted utilise human labelled ophthalmic imaging measures (e.g. Wong et al. & London et al.), which do not necessarily identify the most relevant

features, as we expand upon below. Furthermore, multi-omics approaches have received little attention thus far, leaving mechanistic hypotheses underexplored.

The manuscript presented has two key objectives: (1) to identify associations between ocular features and cardiovascular / neurological disease, (2) to explore the diversity of disease associated with ophthalmic imaging; and (3) to systematically explore potential mechanistic explanations for these associations.

We believe our key contribution is objective (3). Mechanistically, our work builds on the existing literature by (a) using deep learning derived phenotypes to represent ophthalmic images, rather than human-derived features; and (b) exploring mechanistic ocular-systemic links in a systematic manner across the multi-omics space using a range of methods, producing converging results that illustrate the role of the eye as a composite measure of systemic health.

The use of deep learning derived features over human derived features is critical. The literature thus far has focused on human defined features and consequently has converged upon a narrow range of ocular features deemed to be associated with systemic disease. This approach leads to repeated study of features we ***already know*** are important and may neglect more complex or abstract structural features that are challenging to define using human labels. Published work is therefore currently dominated by conventional vessel measures such as venular and arteriolar widths, tortuosity measures, and OCT layer thicknesses (see reviews doi: 10.1007/s00415-023-12171-6, 10.1136/jnnp-2020-325347 and 10.3390/jcdd12060230). Whilst these findings are interesting and immediately interpretable, their clarity of interpretation does not necessarily equate to them being of the upmost importance. This is perhaps brought into focus by our study's conclusion that choroidal features represent a key structure driving associations between OCT and systemic health – contrasting our 2024 systematic review which identified that not a single study on the prediction of stroke had considered the choroid in its analysis. We believe our hypothesis-free, deep learning derived ophthalmic features enable an unbiased approach and for a richer range of potential mechanistic factors to be identified. Recognising we should make this clearer in our manuscript, we have altered our introduction to frame the value of representing features as deep learning embeddings over hand crafted features.

Regarding the reviewers second point, we acknowledge that embeddings have previously been used in GWAS, including with respect to ophthalmic features. We are

strong proponents of this approach, and have previously undertaken GWAS studies using variational autoencoder (doi: 10.1038/s42256-024-00801-1) and conventional autoencoder embeddings (doi: 10.1093/bioinformatics/btae732).

The current GWAS differs significantly to our group's ophthalmic embedding GWAS, and the study highlighted by the reviewer (Taleb et al., 2022 /the 'ContIG' paper) with respect to methodological rigor and the nature of images studied. First, this is the first GWAS of OCT B scan embedding features (previously, only explored in the format of a thickness map designed to simplify the representation task). Second, neither of the previous studies enforced normality of the embeddings, which is an assumption of GWAS. Third, the work by Taleb et al., although ahead of its time as a imaging genetics paper, falls short on GWAS methodological standards: the authors did not perform fine mapping to ensure independence of SNPs, did not perform conventional quality control for genetic analyses (or at least did not report it if it was performed), did not adjust for conventional ophthalmic GWAS covariates (e.g. refractive error), did not follow their analysis with any of the conventional downstream analyses used by statistical geneticists (e.g. MAGMA, LDSC), and failed to validate their discovery GWAS findings.

The work presented in our manuscript is therefore more rigorous than previously presented GWAS in this space and represents a state-of-the-art approach for the genetic analysis of deep learning feature GWAS.

Comment 2

The neural network architecture applies an adversarial loss to the latent embeddings, aiming to structure them according to a Gaussian distribution. As the seminal variational autoencoders (Kingma, and Welling. "Auto-encoding variational bayes." ICLR, 2013) have established using the Kullback-Leibler divergence for this purpose, the authors' decision to employ an adversarial loss warrants further rationale. Ideally, this should be supported by references to prior work utilizing a similar approach.

Response 2

We agree with the reviewer that variational autoencoders (VAE) are a plausible alternative method, **and we have added a comment to this effect in the discussion section referencing the above VAE study.** Our group have previously used VAE representations of cardiac imaging features for GWAS (doi:

10.1038/s42256-024-00801-1) and VAEs of OCT imaging for prediction and genetic studies (e.g. doi: 10.3389/frai.2025.1624550 & 10.1093/eurheartj/ehaf784.4363). In our experience, the challenge with VAE architectures with substantial regularization (through the Kullback-Leibler divergence) is that the reconstruction quality through a narrow bottleneck is relatively poor/lossy (for example, in our previous CFP multi-channel VAE myocardial infarction prediction work, we had to use unsharp masking to ensure that the vasculature was reconstructed – an undesirable approach here as we wanted to preserve colour). Therefore, in this study, we elected to pursue the adversarial autoencoder (AAE) methodology described to balance reconstruction quality/feature preservation against a normalized latent space. The AAE has been described by Makzani et al. (doi: 10.48550/arXiv.1511.05644), and this work is referenced in our manuscript.

Future work comparing the genetic discovery power and reproducibility of VAE/AAE/contrastive methods for imaging genetics would be very interesting but are outside the remit of the present work.

Comment 3

I do not think that a correlation matrix based on Pearson's correlation coefficient is sufficient proof that the individual embeddings are independently distributed. Considering that all embeddings are processed by a highly non-linear neural network, individual embeddings may very well correspond to similar image concepts, even if they are not linearly correlated. As such, I believe that subsequent statistical analyses should not rely on the assumption that the embeddings are independently distributed.

Response 3

Our AAE enforces independently distributed latent vectors by matching the encoder's output to a chosen prior distribution, which is a multivariate Gaussian with zero mean and identity covariance, where all dimensions are independent (described in the section "model architecture" in our manuscript). A discriminator distinguishes true samples from this prior and the encoder's outputs. To fool it, the encoder learns to produce latent vectors with the same independent structure, removing any correlations between dimensions. Therefore, it is reasonable to state (1) based on the presented correlation analysis there is no evidence of substantial dependence, and (2) that the distributional properties enforced by our loss functions ought to promote independence. Of course, embeddings will relate to shared semantic concepts (and

this was demonstrated by GradCAM, and presented in the supplementary figures), but that is not equivalent to statistical dependence. Of the analyses presented, the method which appears to be at-risk of spurious results in the context of shared semantic meaning is the canonical correlation analysis. **Recognising the reviewers concern (and the concern surrounding CCA in a similar theme highlighted by "Reviewer 4 – Comment 11") , we have implemented canonical sparse partial least squares as a means of exploring metabolomic associations in the context of shared semantic concepts amongst embeddings / collinearity amongst metabolites**. The results of sparse partial least squares are presented both in the main text and the supplementary content.

Comment 4

I am doubtful whether the implemented correction for multiple testing, which views each embedding as a "family" and only using a family-wise error rate control, is methodologically sound. In my understanding, correcting for multiple testing aims to guard against false discoveries (Farcomeni, Alessio. "A review of modern multiple hypothesis testing, with particular attention to the false discovery proportion." Statistical methods in medical research 17.4 (2008): 347-388). Using the currently employed correction for multiple testing, one could simply increase the dimensionality of the embedding to 512 to double the number of associations. I strongly believe that all results have to correct for the number of individually processed embeddings.

Response 4

We acknowledge the reviewers concerns regarding type one error risk. The question of how best to approach multiple testing in the age of multi-omic, high dimensional, hypothesis free analyses is nuanced. **In response to the reviewer comment, we have added a section titled "Controlling for type one error" where we discuss our approach and its limitations. We have now also highlighted the hypothesis free nature of the work and/or the approach to multiple testing correction taken in the introduction, results, methods and limitations to ensure honest and transparent reporting throughout the manuscript.**

When undertaking many tests, the risk of type one error is increased, but when applying a strict multiple testing criteria, the risk of type two error is increased. For instance, taking our CFP-feature GWAS studies, if we correct for total embeddings (512) ***and*** total tests in our analysis, classic findings (positive controls) such as

4th December 2025

OCA2, *TSPAN10*, *RDH5* and *VSX2* are not replicated. The failure to replicate well-established associations under these conditions is supportive of over-correction. Applying such a threshold also assumes that all traits should receive the same level of scrutiny, which is untrue given that each analysis has a different level of prior plausibility. With this in mind, it has been argued that multiple-testing correction approaches represent an over-simplification of a highly nuanced issue – particularly in the case of hypothesis free studies such as ours (doi: 10.1016/j.athoracsur.2015.11.024). Some have argued this particularly strongly, asserting that multiple-testing correction should not be carried out whatsoever in large-scale multi-omic studies (doi: 0.1016/S0895-4356(00)00314-0).

The problems with mandating strict multiple-testing corrections in multi-omics studies include:

(1) It increases the probability of type 2 error.
(2) Promotes biased reporting, encouraging academics to omit non-significant results which deflate their p values.
(3) Encouraging authors to 'slice' their work into multiple papers.
(4) Ignoring the reality that some results are more/less plausible a priori based on the existing literature and /or other evidence presented orthogonally.

Instead, we aim to judge associations on their own merits and report outcomes openly. We feel that the application of a study-wide/embedding-wide multiple testing correction in this manuscript would be punitive for endeavouring to systematically assess eye-health relationships in what is the most comprehensive study exploring oculo-systemic health associations to date.

Intending to balance the risks of type 1 and type 2 error, our approach to addressing concerns regarding type 1 error with omics scale data is as follows:

(1) To apply a family wise error rate correction a per embedding basis – which we believe is justified given that embeddings demonstrated little correlation and follow a multivariate Gaussian distribution.
(2) To consider the biological plausibility of findings, and report if there is concern.
(3) To report raw, unadjusted p values alongside any assertions of significance wherever possible – allowing readers to draw their own conclusions surrounding the results.
(4) To implement orthogonal analyses as a form of methodological validation of the results.

(5) To assess the convergence of evidence both within the project and the broader literature.
(6) To validate results where feasible (as we have with our genetic analyses) and triangulate results across analyses.

Notably, several publications in *Nature* family journals have published similar approaches in the context of hypothesis free analyses, including doi: 10.1038/s41467-023-44551-x, 10.1038/s41591-025-04106-7, 10.1038/s44161-022-00091-z, 10.1038/s41598-024-57192-x, and 10.1038/s41598-020-79964-x.

Comment 5

Closely related to the previous two points, I am not sure whether the number of embedding dimensions correlated with a trait is a good metric. Even if all the embeddings were truly uncorrelated and associated with a phenotype, a single embedding may be just as important as 50 others considering that they are subsequently processed by a high-dimensional neural network.

Response 5

We thank the reviewer for their comment. We report individual results both in text and in the supplementary tables, but the high-dimensional nature of the results warrants some simplification in figures, to ensure visualisations are digestible for readers. We recognize the limitations of this approach, and so **we have now added notes to figure legends to highlight that individual significance levels are important and that they are comprehensively presented in the supplementary content.**

Comment 6

The authors use HDBSCAN to cluster an array of ocular, cardiovascular and neurological traits, before associating the clusters with the extracted retinal image embeddings. They motivate this decision as it purposedly "enhances interpretability." However, it is not clear to me how the clusters offer increased interpretability compared to the clearly defined UK Biobank data fields. Without appropriate rationale, I believe this clustering step should be replaced with a more transparent strategy or omitted entirely.

4th December 2025

Response 6

We understand that our rationale for this may benefit from some expansion in the manuscript, and **we have now added content to the relevant figure legend to explain the role of clustering.** The trait-wise association studies in which HDBSCAN was utilised contain a very large number of traits, and the full results are presented according to UK Biobank fields in the supplementary content. However, we recognize that in such a large omics study, we cannot expect readers to be **required** to refer to the supplementary content to get an overview of the findings. Therefore, a means of summarising the results is required.

Our aim was to give readers an impression of the diversity of eye-systemic trait associations, and for it to be made feasible for readers to compare the diversity of associations in a plot. As it's not possible to plot the associations between >1000 traits and 512 ophthalmic features, we sought a means to summarise the traits into groups for presentation. In our initial iteration of this manuscript, we achieved this with a clinician categorising traits according to their clinical expertise/opinion. However, this is highly subjective, and another author/clinician would certainly come to different conclusions regarding the groups of traits available and what ought to belong to each. Similarly, the UK Biobank categories are coarse and as far as we are aware do not follow any specific methodology to assign labels (i.e. they are entirely arbitrary and subjective).

Accordingly, we sought a more scientific, unbiased, and reproducible means to classify traits into related groups. Following a review of the methods available, we determined that a hierarchical, density based clustering model was most appropriate given (1) it allows for clusters of different densities, (2) it allows for traits that don't fit into clusters to be identified as 'noise' rather than forcing traits into categories to which they do not belong, and (3) it scales to large datasets. We were pleased to see that upon appraisal by two clinicians leading this study (THJ and PIS), the statistical solution led to clustering that is biologically / clinically plausible – which we believe the reviewers are likely to agree with should they wish to review results of HDBSCAN clustering in Supplementary Tables 1.22 and 1.28. Whilst other means are available , we would argue strongly that a reproducible and unbiased statistical solution is preferable, and readers who want to explore traits according to individual labels can still do so if they wish to peruse the supplementary content, where the results are available in their entirety.

4th December 2025

Comment 7

Many cardiovascular and neurological diseases are notoriously underdiagnosed (Soljak et al. (2011). Variations in cardiovascular disease under-diagnosis in England: national cross-sectional spatial analysis. BMC cardiovascular disorders, 11(1), 12), which most likely affects the UK Biobank health report labels. I believe the authors should clearly discuss this issue and how it affects their study.

Response 7

We agree with the reviewer's comment - in all studies relying on health data linkage, labels are imperfect for a variety of reasons, some of which are discussed in the highlighted study. **We have now added a mention of data missingness to the limitations paragraph of the discussion.**

We would like to note, that we have controlled for missed labels to the extent it is possible in an observational study by considering UK Biobank censoring dates in our analyses as described in the methods. This is often forgotten best practice for managing disease association studies in UK Biobank and is a methodological strength of the analysis presented, the definition of censoring dates in UK Biobank is:

"The date up to when UK Biobank estimate that data received from that provider is mostly complete.[…] The censoring date is the last day of the month for which the number of records is greater than 90% of the mean of the number of records for the previous three months, except where the data for that month is known to be incomplete in which case the censoring date is the last day of the previous month."

Comment 8

While initially reading the paper, I was wondering whether the authors correct for age and sex. Only far in the methods section, I realized that the authors actually correct for this. I suggest already briefly mentioning this earlier than page 20.

Response 8

We apologise for this omission and have now clearly stated that the correction has been made in the results section.

4th December 2025

Comment 9

As discussed in the paper, ethnicity affects retinal pigmentation and has been linked to an increased prevalence of systemic disease. I believe the authors have to further explain their decision to not correct for these potential confounders as they have done with age and sex.

Response 9

Unlike age and genetic sex, which are objective and directly biologically relevant - self-reported ethnicity and race are social constructs (see doi: 10.1113/EP091491 & 10.1001/jama.2021.13304) defined by a combination of culture, personal and family narratives, and ancestry. In an admixed population such as in the UK, the rationale for ethnicity as a biological trait is even less sound (e.g. see the Response Figure 1 below, sampled from doi: 10.1186/s40246-022-00380-5, which shows the ancestral distribution of subjects based on principal components; and Response Figure 2 which shows principal components plotted and colour coded according to ethnic group – which illustrates how self-reported ethnic group does not necessarily match ancestral group, taken from doi 10.1038/s41586-018-0579-z). There is controversy regarding when ethnicity ought to be used in biological research for this very reason with a range of conflicting guidelines identified in a scoping review (doi: 10.1016/j.ajhg.2022.11.001). Whilst ethnicity has an important role to play in the validation of clinical decision-making tools and for studying social determinants of health outcomes, it should not have a central role in biological studies, and the role of ethnicity in biology is grossly over-emphasised – leading to wide-ranging misconceptions on the differences between ethnic groups amongst the public, scientists and clinicians. Therefore, in our work, we have elected to utilise only genetic ancestry (not ethnicity) as a factor in our analysis, and only in the GWAS methodology, where it is rather uncontroversially biologically relevant.

4th December 2025

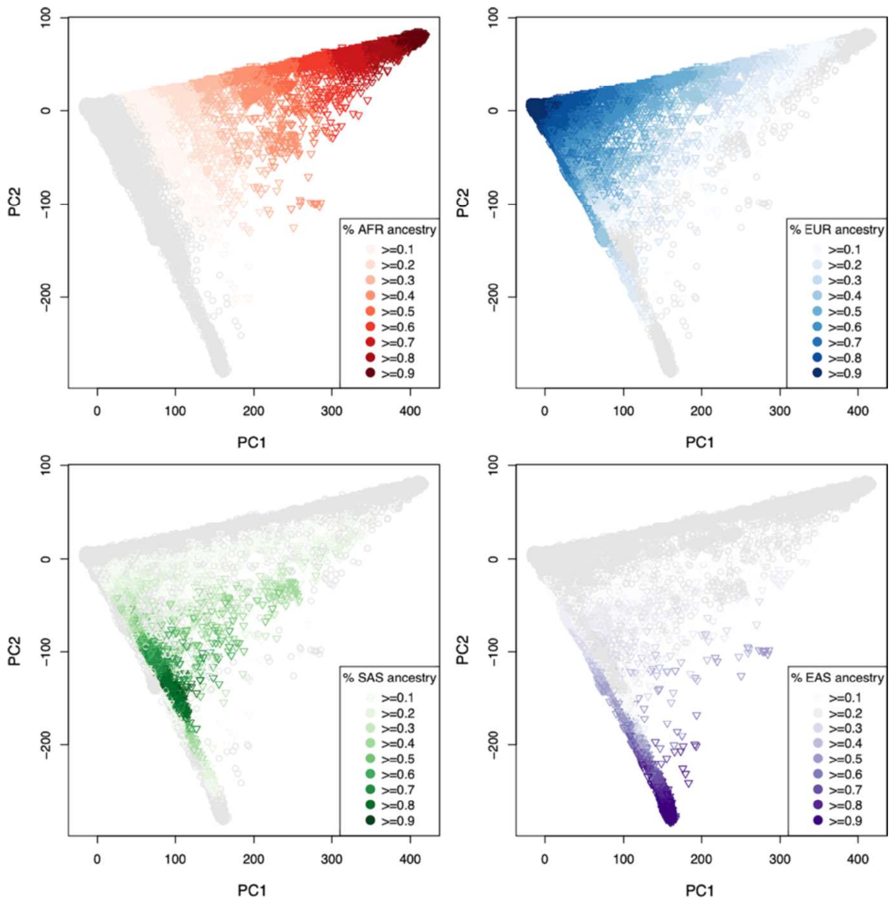

Response Figure 1: Ancestry proportions on UKBB PCs: Continental **A** African, **B** European, **C** South Asian, and **D** East Asian ancestry proportions placed on principal components one and two, as supplied by the UK Biobank. Credit: https://doi.org/10.1186/s40246-022-00380-5

4th December 2025

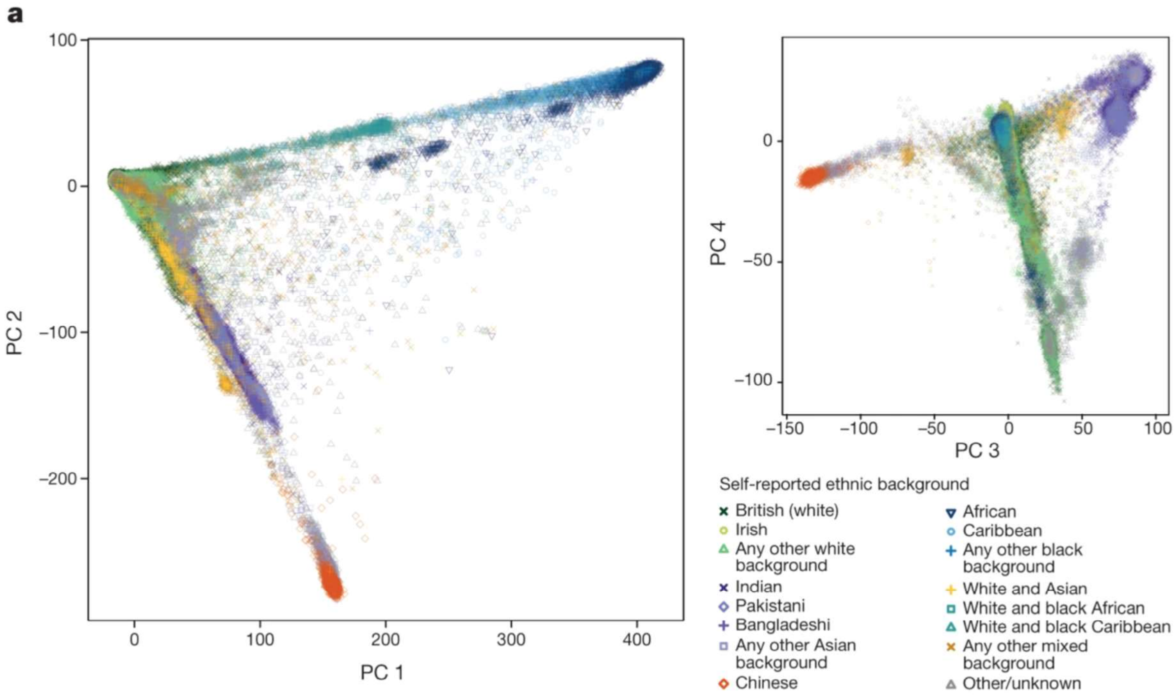

Response Figure 2: Each point represents a UK Biobank participant (n = 488,377 samples) and is placed according to their principal component (PC) scores in each of the top four principal components. Colours and shapes indicate the self-reported ethnic background of each individual. Credit: https://doi.org/10.1038/s41586-018-0579-z

Reviewer #2

Comment 1

The rationale for using the trained Ret-AAE to represent OCT images and CFP as 256-dimensional embeddings requires further explanation. A key issue lies in how these abstract embeddings associate with systemic diseases such as cardiovascular and neurodegenerative conditions, and more importantly, how they can be interpreted in a clinically meaningful way. AI-derived features such as retinal vascular parameters from Automorph (e.g., tortuosity) or retinal ageing biomarkers (e.g., retinal age, reti-Age, reti-phenoAge) are more intuitively interpretable by clinicians and patients. In contrast, the clinical relevance of 256-dimensional embeddings is not immediately clear. Providing additional explanation and context on how these

embeddings can be understood and applied in a clinical setting would strengthen the manuscript.

Response 1

We thank the reviewer for their comment, which highlights ambiguity in the rationale for our autoencoding methodology. **In response to this comment, we have altered our introduction to clarify the value of autoencoder embeddings over human labelled features.**

It is perhaps worth noting that we do not intend for embeddings to be used in a clinical context, as the principal role of this study is to explore mechanistic links between ophthalmic features and systemic health and to scope the breadth of neurological and cardiovascular disease associated with OCT/CFP images. **This is now clearly stated in the discussion.**

Comment 2

Image quality control is crucial for ensuring robust analyses. Could the authors clarify how Automorph was applied to determine image quality and whether any thresholds or exclusion criteria were used? Clear reporting of these methods would improve reproducibility and interpretation.

Response 2

Automorph categorises images according to three categories, "bad", "usable" and "good". We retained "usable" and "good" images. Our selection is detailed in our methods: "*CFPs were excluded if they were of 'reject' quality grading assessed using a deep learning method, 'Automorph' ('usable' and 'good' images were retained)"*. **Following the reviewer comment, we have expanded the relevant description to explain that "Automorph was trained using the EyePACS-Q dataset, using grading provided by two experts according to image illumination, artifacts, and diagnosability of ocular diseases".** The full training scheme for the Automorph model has been described in the model publication "Zhou, Y. *et al.* AutoMorph: Automated retinal vascular morphology quantification via a deep learning pipeline. *Transl. Vis. Sci. Technol.* **11**, 12 (2022)."; this paper is referenced in our manuscript.

4th December 2025

Reviewer #3

Comment 1

One lacking area is the importance of the pivotal APOE gene and its common functional variants - the e2/e3/e3 polymorphism - fundamental in peripheral and local lipid metabolism, risk of cardiovascular disease, age-related macular degeneration, and Alzheimer's disease, vascular dementia, and dementia in general. See PMID:36394841, PMID:31236578, PMID:32808727, PMID:38054895, and PMID:40385391 for inspiration. I suggest you make APOE stratified analyses (e2 carrier, e33, and e4 carrier (excluding e42)

Response 1

We thank the reviewer for their comment and acknowledge the importance of *APOE* in several human diseases and traits.

Having studied the literature further, we understand that the three main *APOE* alleles (ε4, ε3, and ε2) are defined by the combination of two single nucleotide polymorphisms (rs429358 and rs7412). **These variants reside on chromosome 19.**

Having returned to re-assess our GWAS result, we have found that we do not report any variants in association with OCT or CFP features on the entirety of chromosome 19 (please see the manuscript text and GCTA COJO results presented in supplementary tables 1.8 and 1.9). This finding is consistent with work across ophthalmic-imaging GWAS in general, which rarely identify significant, independent variants in chromosome 19 (doi: 10.1111/pcmr.70038, 10.1371/journal.pgen.1009497, 10.1093/bioinformatics/btae732, 10.1038/s42003-023-04836-9, and 10.1371/journal.pgen.1010587) . Given the above, we believe it is exceedingly unlikely that *APOE* is responsible for any of the GWAS hits identified in our study and strongly suspect that using REGENIE's chromosome-wise variant conditioning function ('*--condition-list*') will not alter our results.

If this point remains a significant editorial and/or reviewer concern despite the scientific rationale provided, we are willing to discuss it further and/or re-run the chromosome 19 GWAS with *APOE* conditioning. However, in the absence of any independently significant variants on chromosome 19, it is unclear what benefit this

would provide, and repeating 512 GWAS analyses will expend substantial computational resources unnecessarily.

Comment 2

A stringent GWAS pipeline has been used. REGENIE 4.1 was selected, and all QC work seems appropriate. Please mention how you dealt with palindromic variants. Sufficient details are given to enable validation from external scientists.

Response 2

Regarding palindromic variants, this study does not feature any GWAS meta-analysis, and the method is implemented on the raw imputed genotype data from a single cohort (UK Biobank). The alleles are therefore internally consistent, and it is not necessary to remove palindromic variants. Supporting this assertion, removal of palindromic variants is not part of the REGENIE team's recommended UK Biobank GWAS pipeline: https://rgcgithub.github.io/regenie/recommendations/ .

Reviewer #4

Comment 1

With apologies if I missed it, the manuscript seems not to provide access to code, model weights, or configuration files. I would appreciate the opportunity to review these, and releasing these (e.g. via GitHub + Zenodo DOI) would seem essential for reproducibility.

Response 1

Thank you for your comment. We are very happy to share the model, within the constraints placed by UK Biobank. **We have now shared the model in a public repository which is entirely compliant with UK Biobank policies (https://github.com/TomJulian/Ret-AAE).** The repository features an explanation of the pre-processing required to use the model, and an explanation of the types of images that Ret-AAE can be used with. The model architecture is available for both the encoder and decoder, and weights for the CFP/OCT Ret-AAE encoders are publicly available. We have added the repository link to the manuscript file. We are

unable to share UK Biobank data, and therefore a Zenodo page has not been created (it would be a duplication of the GitHub repository only, and a doi will be available for our work once our work is published). The provided GitHub repository is sufficient to assess the reproducibility of the study by researchers with UK Biobank access.

Please note that we are unable to share the decoder weights (although we have shared the model itself) due to the UK Biobanks policy on generative artificial intelligence, pasted below:

3.1 These restrictions are all contained within UK Biobank's standard MTA, but to be explicit, UK Biobank does not permit any researcher:

- *to (directly or indirectly) feed or enable UK Biobank participant level data to be incorporated into a publicly available Generative AI or similar model; and/or*
- *to enable (however inadvertently) UK Biobank participant level data to be incorporated into a publicly available Generative AI or similar model. This includes making it publicly available on any searchable website by (for example) posting it to GitHub or similar repository.*

Comment 2

Some further details of training the model (beyond the 35 epochs per configuration in the hyperparameter sweep) would be appreciated (the total number of epochs, convergence criteria, random-seed settings, …). Moreover, given the instability of adversarial training, I think that evidence of convergence (loss curves or reconstruction trajectories) should be provided.

Response 2

We have now provided loss curves; they are presented in the "Extended Supplementary Figures". We have also added additional information regarding the training schedule to "Additional AAE experimental details" for reproducibility purposes.

Comment 3

Regarding the Bayesian hyperparameter sweep using the Weights & Biases platform, could the W&B version, as well as the hyperparameter search space, and acquisition function for the BayesOpt be provided?

4th December 2025

Response 3

We added the W&B version to our methods. Regarding the Bayesian optimisation, the W&B platform only have a single BayesOpt function which isn't customizable – explaining this isn't expanded on. The sweep function is described in this repo: https://github.com/wandb/sweeps/blob/master/src/sweeps/bayes_search.py . **With regards to the search space, we have added a figure to the 'Extended Supplementary Figures" which highlights the parameters.**

Comment 4

I understood the choice of a 256-dimensional latent space to provide a balance between reconstruction quality (SSIM improvement compared to 128 dimensions) and computational/statistical feasibility. This seems reasonable; however, I couldn't help but wonder if the downstream analyses were robust to this choice of dimensionality? Could the authors discuss the robustness of the downstream findings to the choice of latent dimensionality (e.g. whether key associations are expected to be similar at 128 vs 256 dimensions), even if a full re-analysis is not feasible?

Response 4

Thank you for your comment. Increasing/decreasing the dimensionality of the model would necessarily alter the results for **specific embeddings** given that the features would be represented across a broader/narrower range of features. However, it is highly unlikely that the cardiovascular and neurological themes identified are a product of vector dimensionality, and we have presented convergence of evidence across disease association, physiological measures, radiomic, metabolic, and genomic tests – offering strong protection against type 1 error. Secondly, although altering the embedding dimensionality will impact the values of individual embeddings, the same overall ocular features will necessarily be encoded, and there is therefore no reason to believe that the overall themes would change with increased/decreased dimensionality (i.e. the overall finding of cardiovascular and neurological associations). The range of positive control studies conducted, strong reconstruction performance, and GradCAM visualisations indicate that the encoder is successfully representing the expected ocular features, rather than encoding irrelevant abstractions that might lead to spurious results. In the absence of a rationale for why the cardiovascular and neurodegenerative themes identified might change with altered embedding dimensions, we do not feel that there is sufficient

justification to repeat the extensive analyses conducted to assess the impact of dimensionality. We are willing to discuss this further if the editor deems it necessary.

Comment 5

The OCT dataset (~89 k images) is quite a lot larger than the CFP dataset (~64 k) – should this be discussed in the manuscript, in terms of the potential impact on reconstruction quality and embedding stability (as well as on downstream analyses)?

Response 5

We thank the reviewer for their comment. We recognise the asymmetry, but our results indicate that the CFP sample size did not pose any problems. The sample size in both cases is sufficiently large for training, as demonstrated in our reconstruction metrics and example reconstructions presented. The asymmetry of sample size is a product of the quality control metrics available for each image modality and conventional thresholds utilized within these metrics. We do not find any evidence to suggest that this has proven problematic (especially given that our reconstruction metrics were superior for the CFPs despite the smaller sample size, and that, for multiple analyses, there were a larger number of associations for CFP features than OCT). **Therefore, to the best of our knowledge, the asymmetric sample sizes have therefore not created any systematic bias in the CFP-related results that ought to be referenced in the manuscript.**

Comment 6

Running 512 separate GWAS (256 CFP + 256 OCT embeddings) would seem to introduce a substantial multiple-testing burden. Unless I have misunderstood, the authors are applying a standard genome-wide threshold, which provides appropriate control of false positives within each GWAS, but not across all 512 analyses taken together? If this is correct, the authors could consider either applying a cross-trait correction (e.g. Bonferroni, FDR, ..), or the analyses could be framed as exploratory (although I acknowledge that the instance-1 replication mitigates false positives to some extent). [Relatedly, I think the reported genetic correlations derived from LD score regression should be interpreted cautiously, given the small number of embeddings with reliably measurable heritability -- also, it was not entirely clear to me how multiple-testing correction was handled within these correlation analyses.]

4th December 2025

Response 6

Please kindly see our response to "Reviewer 1 , Response 4" where we have responded to a similar comment in detail. In response to the comments surrounding multiple comparisons **we have added a section titled "Controlling for type one error" where we discuss our approach and its limitations. We have now also highlighted the hypothesis free nature of the work and/or the approach to multiple testing correction taken in the introduction, results, methods and limitations to ensure honest and transparent reporting throughout the manuscript.** With respect the heritability analysis, LDSC does not output p values for these analyses. Instead, it outputs an estimate of polygenicity, and if the trait is polygenic then it outputs the h^2. The details of the LDSC outputs for heritability analyses are documented here: https://github.com/bulik/ldsc/wiki/Heritability-and-Genetic-Correlation. **To avoid confusion, we have now noted this in the LDSC section of our methods.**

Comment 7

Only a small fraction of embeddings (≈3 % OCT, 9 % CFP) show measurable heritability. This low polygenicity would seem to suggest that most embedding variance is environmental or technical – could the authors discuss this?

Response 7

This is an excellent point, and **we have added a sentence to the results to expand on this.** Our results may well suggest that some embeddings represent technical factors or features which are more environmental than genetic. Whilst almost all non-technical traits will likely be determined by a combination of genetic and environmental factors, this does not necessarily translate to all traits being heritable to a statistically significant degree. It does not however indicate that there is no genetic component to that trait (not least, because a trait can be genetically influenced but not polygenic). Secondly, it should be noted that we trained the model on both left and right eye images in order to ensure that the encoder would be useful to the academic community regardless of whether academics had laterality labels, but the genetic analysis is conducted on the left eye only (in keeping with convention in the field, see doi: 10.1093/bioinformatics/btae732, 10.1371/journal.pgen.1009497, 10.1371/journal.pgen.1010587). It is therefore also possible that some embeddings associate more strongly with right eye features than left eye features. Therefore, in

summary, there are three likely sources of non-heritable embeddings: (1) technical factors, (2) embeddings associated with right eye features, (3) environmentally determined features, (4) embeddings that may well have genetic components, but which do not demonstrate a sufficiently polygenic architecture to demonstrate statistically significant heritability (e.g., multifactorial inheritance).

Comment 8

Associations with cardiovascular and neurological traits vary in sample size (≈11k–63k). If I have understood correctly, the authors restricted each analysis to participants with available data for the relevant modality or trait. It would be very helpful to include a supplementary summary table characterising the extent and pattern of missingness across data modalities, and ideally comparing participant characteristics (e.g. age, sex, health indicators) between those with and without each data type. This would provide readers with a sense of whether missingness might be non-random and therefore a potential source of bias.

Response 8

Thank you for your comment. We recognize the risk of bias in UK Biobank, and that population characteristics may vary according to missingness. **We have added a section to our results to highlight this ("Population characteristics of those included in the phenotypic and genotypic analyses").** It is feasible for us to present the population characteristics according to whether a subject underwent ophthalmic imaging or not, and so we have now included figures comparing age, sex, ethnicity and deprivation indices in the supplementary content, and presented the values numerically in text. **We have also acknowledged that missingness may be a UK Biobank limitation in the discussion.** However, given that this study tests several thousand associations, it less feasible to present figures or tables to comprehensively delineate the pattern of missingness across all tests.

Comment 9

For the baseline disease associations, my understanding is that participants with existing disease at the time of imaging were included. This makes it difficult to disentangle underlying risk from the effects of established disease or treatment. The manuscript also reports prospective (incident) disease analyses using Cox models;

could the authors provide a direct comparison of these results with the baseline (prevalent) analyses for key outcomes, to help clarify how embedding differences relate to risk versus existing disease?

Response 9

The ‘baseline associations’ are the disease associations between OCT/CFP imaging features and diseases present at the time of imaging (i.e., those already diagnosed). The subjects therefore necessarily have their existing diagnoses included. The above comment highlights that this was not clear in our original submission, and so **we have now stated the definition of baseline explicitly in the results section. We acknowledge that it is possible that some associations may be pharmacologically mediated, and we have added this consideration to the limitations section of our discussion**. With regards to comparing the embedding-disease associations at baseline and for disease prediction, the abstract nature of the embedding features means that comparisons of how embeddings differ in their disease prediction profiles does not offer a great deal of enhanced interpretability for readers and therefore we have not presently included such a discussion.

Comment 10

Cox proportional hazards models seem sensible for the analyses performed. To strengthen the results, the authors might consider explicitly stating how the proportional-hazards assumption was assessed. Reporting diagnostics - such as those based on Schoenfeld residuals or graphical methods - for key outcomes (e.g. hypertension, heart failure, and dementia) may give readers greater confidence in the validity of the reported hazard ratios.

Response 10

Thank you for your comment, **we have now included the Schoenfeld residual plots (and associated *p*-values) in the “Extended Supplementary Figures”.** However, we note that it has been argued that formally testing the proportional hazards assumption is often unnecessary (doi: 10.1093/aje/kwae002 , and as advised in the proportional hazards library). It is therefore unclear whether the large amount of data/figures this assumption analysis generates adds significant value for the readership.

4th December 2025

Comment 11

CCA seems an appropriate and sensible analysis approach here, but it can be prone to overfitting in high-dimensional settings. To mitigate this, the authors could consider using a cross-validated and/or regularised (e.g. sparse or penalised) CCA.

Response 11

We thank the reviewer for their excellent suggestion. **Following this feedback, we have validated our analysis with canonical sparse partial least squares**, finding similar HDL, LDL and VLDL compositional associations with embedding features. The results are presented in the metabolomic section of the manuscript alongside conventional CCA.

Comment 12

The manuscript provides a qualitative summary of the dominant metabolites (e.g. HDL, LDL, VLDL) and lists the top contributing metabolites and embeddings in the supplementary tables. However, interpretability might be further enhanced by visualising the broader loading structure (e.g. clustered heatmaps or correlation maps).

Response 12

Thank you for your suggestion. **Following your feedback, we have presented clustered heat maps** (using SPLS, mixOmics) and these are presented in the Supplementary Figures.

Comment 13

General comment re: effect sizes: Effect sizes across analyses are typically small ($|r| < 0.2$, modest hazard ratios). The manuscript might benefit from explicit discussion of practical significance versus statistical significance. Even with large samples, very small effects may lack biological or clinical meaning. For Cox models, discrimination metrics could be reported to quantify predictive value.

4th December 2025

Response 13

We are conscious that extended reporting of discrimination and clinical significance may cause confusion with readers. Our methodology is designed to explore the breadth of oculo-systemic associations, provide mechanistic hypothesis, and align findings with human interpretable concepts to the extent that this is possible using GradCAM. If we were to wish to develop a predictive model, we would do so using all 256 embeddings and using a machine learning classifier rather than a Cox model. We do not want to give readers the impression that Ret-AAE has been designed for clinical implementation. With that said, **we have made survival curves for the partial effects of percentile changes in the embedding values for key diseases and presented these in the Extended Supplementary Figures** for any readers who might wish to get a better understanding of the discriminative value of individual embeddings. With regards to clinical vs statistical significance**, we have now added a note in the limitations section to clarify that this model has not been designed for clinical purposes / that clinical and statistical significance are not the same.**

Comment 14

General comment re: instance 0/1 split: The instance 0 / instance 1 split is clearly used for GWAS discovery and replication, and I can see that the CCA analysis restricts to instance 0 due to participant overlap. However, it was less clear to me whether this separation was applied consistently across all other analyses – could this be clarified?

Response 14

Thank you for your comment, and our apologies for this omission**. We have now made this clear in the methods.**
To summarise here additionally:

- **AAE training:** instance 0 and 1 images were used.
- **Genetic analyses:** instance 0 for discovery, instance 1 for validation (overlapping subjects were excluded from instance 1).
- **Metabolomic analyses:** There is substantial overlap in the instances, and therefore only instance 0 was used.
- **Cox proportional hazards, t-test, and correlation analyses:** instances 0 and 1 were used, but where a subject was imaged at both instances, only the instance 0 image was considered.

6th February 2026

Dr Thomas H. Julian
Division of Evolution, Infection and Genomics,
School of Biological Sciences,
Faculty of Biology, Medicine, and Health,
University of Manchester,
Manchester, UK.
Email: thomas.julian@manchester.ac.uk

Dear Reviewers and Nature Cardiovascular Research editorial team,

Thank you for taking the time to re-review our manuscript titled "*Ophthalmic imaging as a measure of cardiovascular and neurological health: a multi-omic analysis of deep-learning derived phenotypes*".

We are grateful for your thoughtful and comprehensive feedback. Having reviewed the comments, we have conducted additional analyses to strengthen our manuscript.

Please find our responses to your comments below. Thank you for your time and consideration.

Kind regards,
Tom Julian and Panos Sergouniotls on behalf of the authors

6th February 2026

Response to editorial comments:

Comment 1

In general, we agree with the reviewers that the work has substantially improved after the revision, and you will see that our referees #2 and 3 now support its publication. At the same time, our referees #1 and 4 have raised some additional points, and we feel that addressing these remarks would strengthen the manuscript further. We would be happy to look at a revised manuscript that has been revised accordingly (unless something similar has been accepted at Nature Cardiovascular Research or appeared elsewhere in the meantime).

Response 1

We are pleased to see that reviewers #2 and #3 support publication of our manuscript, and we are grateful for the opportunity to revise our work further in response to the comments of reviewer #1 and #4.

Comment 2

You will see that our referee #1 asks for either some external validation of the findings or for a more straightforward link between the machine learning embeddings and human-interpretable concepts.

Response 2

Thank you for your comment. Whilst , to the best of our knowledge, an appropriate external CFP/OCT dataset with multi-omic data does not exist, we are able to strengthen our work through the suggested increased alignment with human concepts.

The strength of our approach is that it does not rely on human interpretable concepts to align ophthalmic imaging phenotypes with systemic health. We consider this a feature of our work, rather than a limitation. With that said, we appreciate that it would be informative for some readers to discuss relevant concepts in conventional ophthalmic terms, and we want our work to be useful and interpretable to a wide audience. With a view to providing further interpretability, we have conducted a series

of latent traversals. Please see our response to "Reviewer 1, Response 1" for detailed description of this work.

Comment 3

It is our editorial recommendation that you address referee #4's requests by including a sensitivity analysis to investigate the performance of the model when using a 128-dimensional model.

Response 3

In response to the reviewer feedback, we have now trained 128-dimensional models for CFP and OCT images. Using these autoencoders, we have explored the sensitivity of our disease association studies to selection of dimensionality. Reassuringly, our sensitivity analysis recapitulated the findings of our 256-dimensional encoders. Please see our response to "Reviewer 4, Response 2" for a detailed response.

Reviewers' comments:

Reviewer #1

Comment 1

I agree with the authors' decision to avoid already established ocular biomarkers, such as retinal layer thicknesses or vessel diameters. However, the proposed deep-learning-derived biomarkers are inherently difficult to interpret. As already mentioned in my initial review, the proposed method is restricted by the limited ability of class activation maps to link embeddings back to human-interpretable concepts. Additionally, I believe that the data-driven clustering of the traits adds another level of abstraction to the entire pipeline, even if the clusters are deemed plausible by experts and I understand the practical rationale for this step.

6th February 2026

Response 1

We thank the reviewer for raising this issue. Our use of abstract image representations is deliberate and central to the aims of this work. Unlike supervised approaches that are explicitly constrained to reproduce predefined human annotations, autoencoders learn compact representations optimised for faithful reconstruction. Therefore, the resulting latent dimensions are not expected to correspond one-to-one with human-defined traits.

Although direct alignment of individual latent dimensions with scalar human measurements is not possible, it is feasible to probe the *type* of image information encoded by each dimension. In response to this comment, we have therefore undertaken additional analyses aimed at characterising and contextualising the learned embeddings using complementary visualisation strategies.

Specifically, we performed systematic latent traversals to examine the effect of isolated variation along each latent dimension. For each latent variable z_i, we first identified a representative reference image whose coordinate along that dimension lay closest to the population mean (approximately zero). Holding all other latent variables fixed, we independently perturbed z_i by ±4 standard deviations and decoded the resulting latent vectors using the trained adversarial autoencoder decoder. This procedure allows us to visualise the marginal effect of each latent dimension on the reconstructed image (as per doi: 10.48550/arXiv.1807.10267).

Because the induced changes are often subtle, qualitative inspection alone is challenging. We therefore complemented decoded images with quantitative difference maps. For colour fundus photographs (three-channel RGB), we computed per-pixel mean changes across colour channels relative to the reference reconstruction. For OCT images (single-channel), we computed absolute per-pixel intensity differences. These maps highlight anatomical regions and image structures most sensitive to variation in each latent dimension and provide a more objective basis for interpretation.

To ground these observations in clinical context, all latent traversals were independently reviewed by an ophthalmology registrar (T. Julian) and an ophthalmology consultant (P. Sergouniotis), who jointly assessed the anatomical patterns emerging across traversals and related them, where possible, to established

ophthalmic descriptors. Interpretations are reported conservatively alongside the previously reported saliency maps in the legends of **Supplementary Figures 19-73.**

In several cases, perturbations produced clear and anatomically localised effects. For example, CFP embedding 148 (genetically associated with melanin biosynthesis) showed pronounced changes in fundal pigmentation and overall illumination (**Figure 1** below), while OCT embedding 15 was associated with variation in nasal retinal nerve fibre layer thickness (**Figure 2** below). In other cases, effects were diffuse or subtle. For instance, OCT embedding 153 was associated with global shifts in pixel intensity and enhanced definition of choroidal vasculature at −4 SD relative to +4 SD (**Figure 3** below). Such patterns are unlikely to be annotated reliably by human graders, yet are biologically plausible, illustrating the capacity of autoencoders to capture sub-perceptual image variation.

Taken together, these analyses provide evidence that the learned embeddings encode meaningful and spatially structured image information that can, in some cases, be related to known anatomical or biological features. By adding latent traversals, difference maps, and explicit clinical annotation in addition to our existing saliency mapping, we believe we have addressed the reviewer's concern to the extent that is methodologically justifiable for unsupervised representation learning. The latent traversals are presented in the **Supplementary Figures**.

6th February 2026

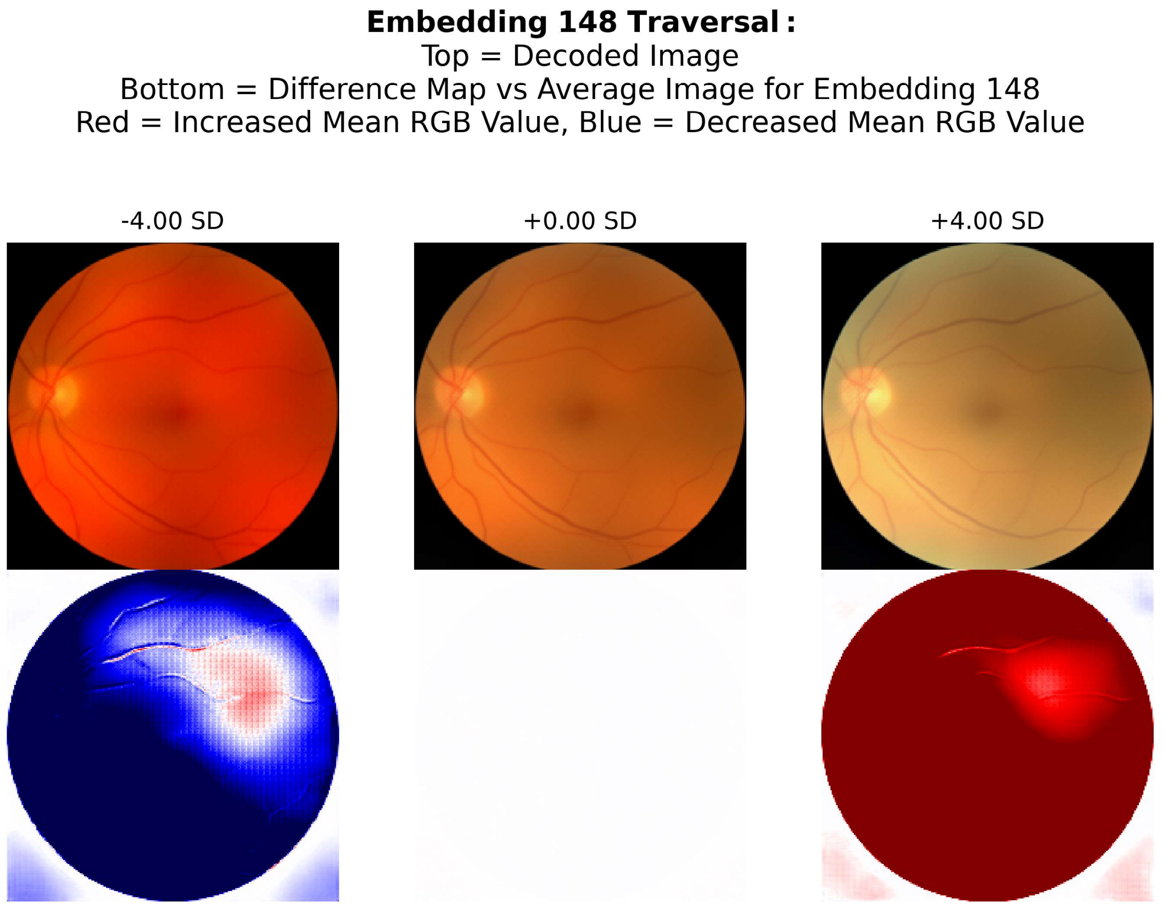

Figure 1: Top row - reconstructions obtained by perturbing latent dimension 148 by ±4 standard deviations (SD) around its mean, while holding all other latent dimensions constant. Genetic analyses revealed this embedding was associated with melanin biosynthesis. The central image corresponds to the reference latent vector. Bottom row - difference maps showing the mean per-pixel change in RGB intensity relative to the reference reconstruction. Red denotes increased mean RGB intensity, and blue denotes decreased intensity. Perturbation of this latent dimension has a clear effect on fundal pigmentation. Reproduced with the permission of UK Biobank.

6th February 2026

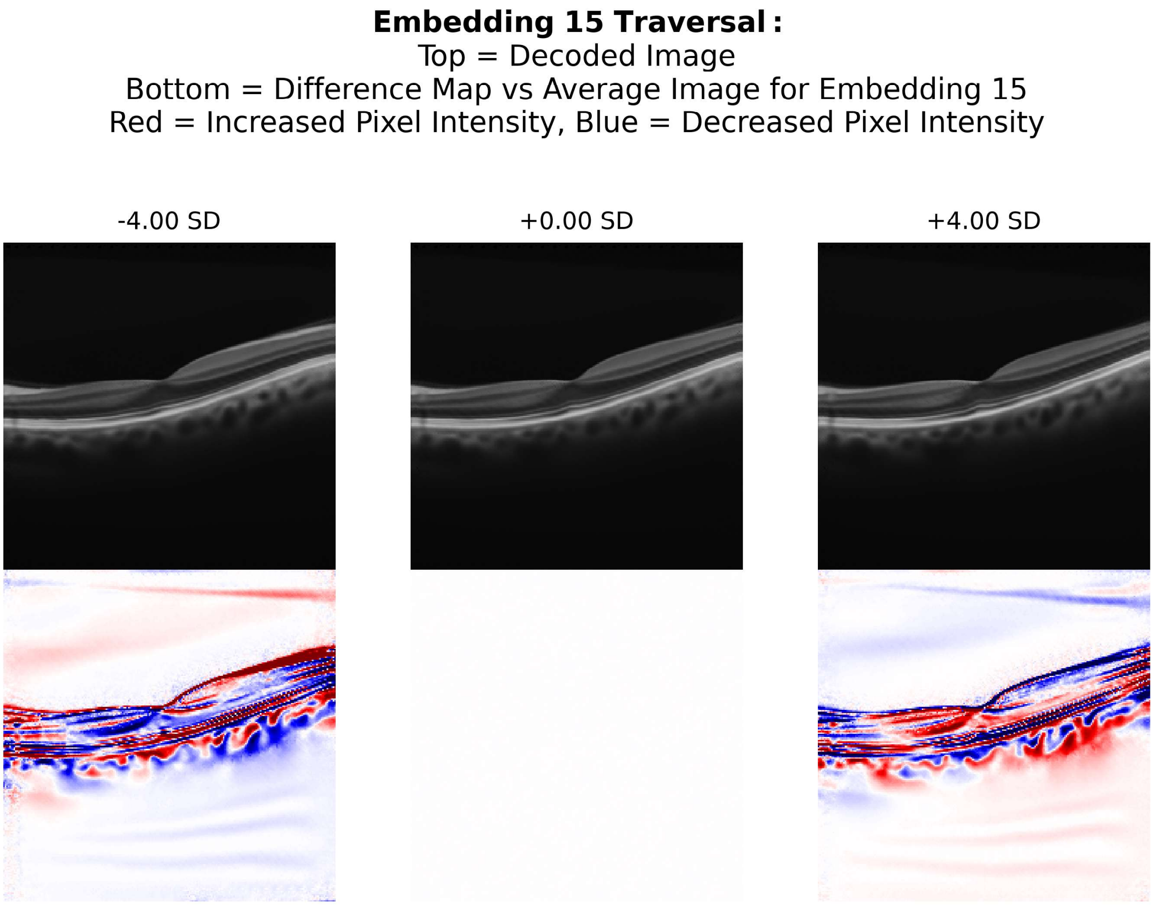

Figure 2: Top row - reconstructions obtained by perturbing latent dimension 15 by ±4 standard deviations (SD) around its mean, while holding all other latent dimensions constant. This embedding was the OCT embedding most strongly associated with baseline Parkinson's disease. The central image corresponds to the reference latent vector. Bottom row - difference maps showing the per-pixel change in intensity relative to the reference reconstruction. Red denotes increased intensity, and blue denotes decreased intensity. The most visually obvious impact of perturbation of this latent dimension is thinning of the temporal retinal nerve fibre layer (the superior, bright layer on the right side of the image) from -4.00SD to +4.00 SD. However, the difference map suggests there are broader pixel intensity differences across all layers that are more challenging to identify with the human eye. Reproduced with the permission of UK Biobank.

6th February 2026

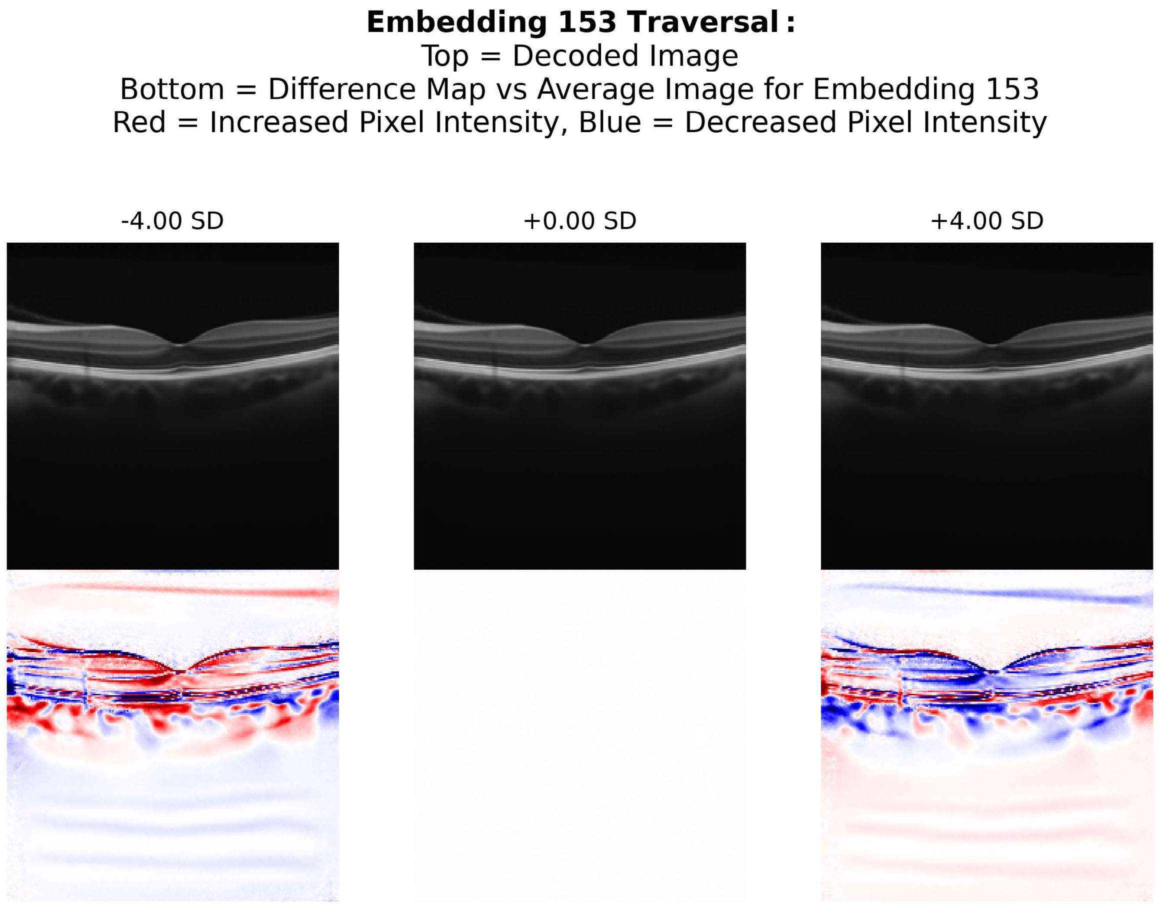

Figure 3: Top row - reconstructions obtained by perturbing latent dimension 153 by ±4 standard deviations (SD) around its mean, while holding all other latent dimensions constant. This was the OCT feature most strongly associated with the gene set 'KEGG medicus variant duplication or mutation activated *FLT3* to Jak-STAT signaling pathway'. The central image corresponds to the reference latent vector. Bottom row - difference maps showing the per-pixel change in intensity relative to the reference reconstruction. Red denotes increased intensity, and blue denotes decreased intensity. It is noted that the choroidal vessels are more well defined at -4.00SD than +4.00SD, and that there are subtle changes to pixel intensity across the image. Reproduced with the permission of UK Biobank.

Comment 2

The concerns regarding the robustness of the discovered associations is directly linked to the previous comments regarding the correction for multiple testing by

Reviewer #4 and me. The authors have attempted to alleviate these concerns by adding a section on the type I error risk. While I understand the difficulty balancing type I and type II errors and issues surrounding correcting for multiple testing, I still think that the definition of individual embeddings as a family is arbitrary. Importantly, the authors write that "key study findings will benefit from external experimental validation." Indeed, I believe that replicating (parts of) the results on a separate dataset would convincingly demonstrate the robustness and practical value of the proposed method.

Response 2

We agree with the reviewer that independent replication would be the most direct way to demonstrate robustness. In an ideal setting, this would involve re-analysing a fully independent cohort with paired OCT, CFP, and matched multi-omic data (i.e. in a similar manner to the UK Biobank protocol). At present, no such dataset exists to our knowledge, which makes external replication infeasible rather than omitted by choice. We have therefore been explicit in the manuscript about this limitation and the resulting constraints on interpretation.

Notably, our primary conclusions do not rely on isolated associations near a significance threshold. Instead, they are supported by convergent evidence across multiple analyses, including consistent effect directions, biologically coherent patterns, and agreement with established retinal and systemic pathways. Additionally, during this second round of review, we have undertaken sensitivity analysis using 128-dimensional models as requested by Reviewer #4 (Comment #2), validating our findings under a more compressed latent dimensionality. While this does not substitute for external replication, it does argue against the findings being driven solely by chance fluctuations.

Taken together, we believe the analytical framework and reporting adopted here appropriately balance sensitivity and statistical caution in a high-dimensional setting. By explicitly describing error trade-offs and clearly framing the findings in context, we aim to enable informed interpretation while avoiding overstatement of robustness in the absence of external replication.

6th February 2026

Reviewer #2

Comment 1

Authors have addressed all my concerns.

Response 1

We are pleased to have addressed the reviewers' comments. We thank the reviewer for taking the time to review our study.

Reviewer #4

Comment 1

I thank the authors for their revisions and thoughtful responses, and am particularly grateful for the GitHub link. Almost all of my previous points have been comprehensively addressed.

Response 1

We are grateful to the reviewer for taking the time to review our study a second time, and we are pleased to have addressed most of their comments through the previous revision.

Comment 2

I wonder if the authors could consider either: (i) A limited sensitivity analysis, checking whether some of the top disease associations per modality remain qualitatively consistent when using the 128-dimensional model; or (ii) An acknowledgement in the limitations that the choice of 256 dimensions was pragmatic, that reconstruction quality does not guarantee identical feature organisation across dimensionalities, and that downstream findings might be sensitive to this choice.

6th February 2026

Response 2

We acknowledge the reviewers concern, and we appreciate the reviewer's pragmatic suggestions.

Following this comment, we trained 128-dimensional models to represent CFP and OCT images. As suggested, we subsequently utilised the embeddings to undertake a sensitivity analysis in relation to future disease risk (Cox proportional hazards). The results of this analysis are presented **in Supplementary Figures 90 & 91.** As can be seen in the figures, the leading disease associations were verified in this analysis. These results are now discussed in the cox proportional hazards section of the manuscript (**page 12**).
